# Chlorophyll-Inspired Red-Region Fluorophores: Building Block Synthesis and Studies in Aqueous Media

**DOI:** 10.3390/molecules23010130

**Published:** 2018-01-10

**Authors:** Rui Liu, Mengran Liu, Don Hood, Chih-Yuan Chen, Christopher J. MacNevin, Dewey Holten, Jonathan S. Lindsey

**Affiliations:** 1 Department of Chemistry, North Carolina State University, Raleigh, NC 27695-8204, USA; rliu8@ncsu.edu (R.L.); mliu5@ncsu.edu (M.L.); 2 Department of Chemistry, Washington University, St. Louis, MO 63130-4889, USA; hoodlaw@sbcglobal.net; 3 NIRvana Sciences, Inc., Research Triangle Park, NC 27709, USA; chen@nirvanasciences.com (C.-Y.C.); chris@nirvanasciences.com (C.J.M.)

**Keywords:** amphiphilic, bioconjugation, chlorin, fluorescence, micelle, Poisson, PEG, protein

## Abstract

Fluorophores that absorb and emit in the red spectral region (600–700 nm) are of great interest in photochemistry and photomedicine. Eight new target chlorins (and 19 new chlorins altogether)—analogues of chlorophyll—of different polarities have been designed and synthesized for various applications; seven of the chlorins are equipped with a bioconjugatable tether. Hydrophobic or amphiphilic chlorins in a non-polar organic solvent (toluene), polar organic solvent (DMF), and aqueous or aqueous micellar media show a sharp emission band in the red region and modest fluorescence quantum yield (Φ_f_ = 0.2–0.3). A Poisson analysis implies most micelles are empty and few contain >1 chlorin. Water-soluble chlorins each bearing three PEG (oligoethyleneglycol) groups exhibit narrow emission bands (full-width-at-half maximum <25 nm). The lifetime of the lowest singlet excited state and the corresponding yields and rate constants for depopulation pathways (fluorescence, intersystem crossing, internal conversion) are generally little affected by the PEG groups or dissolution in aqueous or organic media. A set of chlorin–avidin conjugates revealed a 2-fold increase in Φ_f_ with increased average chlorin/avidin ratio (2.3–12). In summary, the chlorins of various polarities described herein are well suited as red-emitting fluorophores for applications in aqueous or organic media.

## 1. Introduction

**Figure 1:**
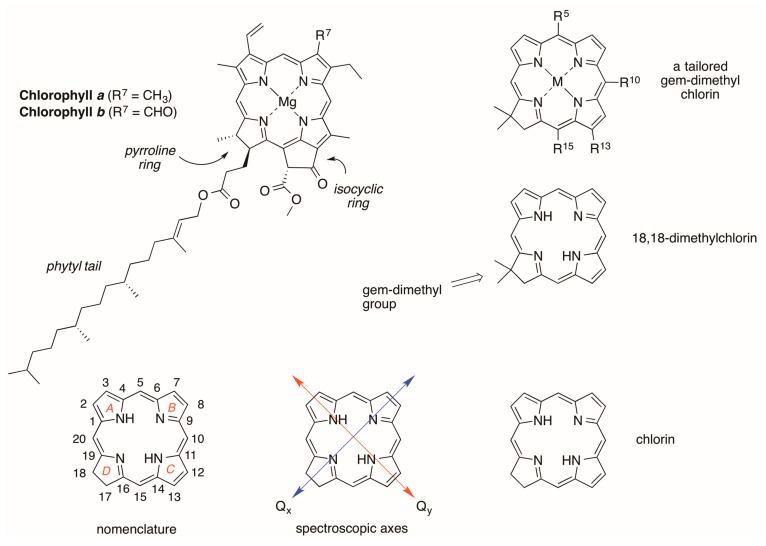
Natural chlorophylls, terminology, and synthetic chlorins.

Nature’s “advanced functional dyes”—chosen by the fine comb of evolution [1,2,3,4,5,6]—are the chlorophylls and their analogues. The structures of chlorophyll *a* and chlorophyll *b* are shown in Figure 1 [7]. Chlorophylls *a* and *b* differ only in the nature of a single substituent at the 7-position but otherwise contain common features: (1) an 18π-aromatic macrocycle; (2) three pyrrole rings and one reduced, “pyrroline” ring; (3) a fifth, “isocyclic” ring spanning positions 13 and 15 and containing a β-ketoester unit; (4) a full complement of substituents at the β-sites of the pyrrole or pyrroline rings; (5) a centrally coordinated divalent magnesium ion; (6) a phytyl tail; (7) *trans*-configuration of the vicinal alkyl substituents at the β-sites of the pyrroline ring; and (8) vinyl and keto groups in conjugation with the π-system and disposed along the Q_y_ axis. Chlorophylls *a* and *b* absorb strongly in the blue (and near-ultraviolet) and in the red spectral regions; green light is absorbed with weaker intensity, hence the characteristic color of the compounds in thin films (e.g., a leaf) or dilute solutions, and the verdant landscapes of Earth [8,9].

A longstanding objective in photochemistry, artificial photosynthesis and allied disciplines has been to create synthetic chlorophyll-like molecules as a means to examine the essential structural features in the native macrocycles and to tailor the molecules for diverse applications. In this regard, the phytyl group, which constitutes about 1/3 of the overall mass [8], does not contribute to the core photophysical features of chlorophylls. Chlorins lacking the 3-vinyl and 13-keto substituents still exhibit chlorin-like spectral features; the red-region absorption is certainly diminished versus that of chlorophylls but still enhanced by at least 5–10-fold versus that of a porphyrin [9]. The central magnesium affords a more reducing macrocycle versus the zinc chelate and even more so than the free base macrocycle, yet in broad perspective, the magnesium, zinc and free base systems have similar photophysical features. Hence, the phytyl tail, isocyclic ring, and central magnesium can often be elided in creating synthetic chlorins without loss of photochemical function [9].

The chief methods of synthesis of chlorins include derivatization of porphyrins [9,10], semisynthetic tailoring of chlorophylls [11], and de novo routes [8,12]. We have been working over the years to learn to create chlorins in de novo fashion, inspired by the roles of chlorophyll in photosynthesis, and with an eye toward exploiting the resulting synthetic chlorins in fundamental photophysical studies and diverse applications ranging from the materials sciences to biomedicine [8]. A key design element is the inclusion of a geminal-dialkyl (typically a gem-dimethyl) group in the pyrroline ring (Figure 1), thereby securing the chlorin π-system from adventitious dehydrogenation upon routine handling in aerobic environments; chlorin itself (lacking any substituents) is susceptible in this regard, the consequence of which is formation of the corresponding porphyrin and loss of the desired red-region absorption intensity. In this paper, we describe the de novo synthesis of a set of gem-dimethyl-substituted chlorins aimed primarily toward studies in the life sciences.

**Figure 2:**
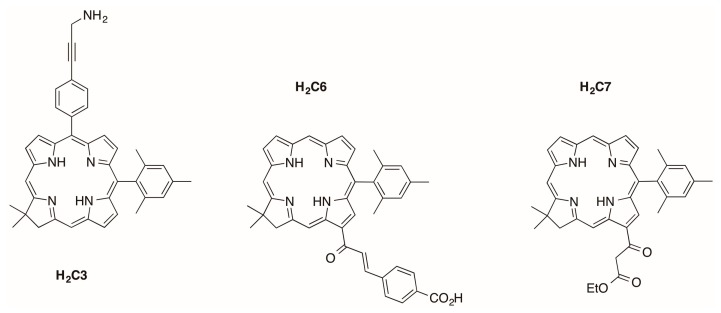
Target synthetic chlorins.

The first three target synthetic chlorins are shown in Figure 2. Chlorins **H_2_C3**, **H_2_C6** and **H_2_C7** each contain a 10-mesityl group and one appended group including a 1° amine, a β-ketoester and a carboxylic acid-substituted chalcone, respectively. Chlorins **H_2_C3** and **H_2_C6** are amphiphilic given the presence of a hydrophobic core and an ionizable terminal functional group, whereas chlorin **H_2_C7** is relatively hydrophobic and lacks an ionizable functionality. This work extends our prior research toward amphiphilic chlorins [13] and draws on prior routes for installation of the β-ketoester unit [14]. All three chlorins can be regarded as building blocks given the presence of a single derivatizable group attached to the macrocycle. The photophysical properties of all three chlorins have been examined in toluene, whereas chlorins **H_2_C3** and **H_2_C6** have been further examined in DMF and in aqueous micellar media.

**Figure 3:**
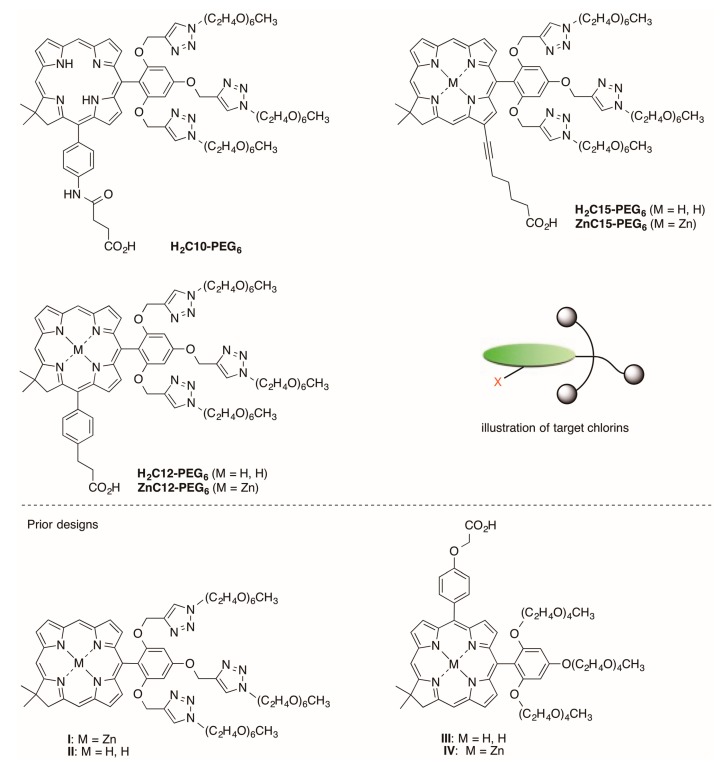
Target synthetic PEgylated chlorins (**upper**) with illustration, and prior designs (**lower**).

Five other target synthetic chlorins are shown in Figure 3. Free base chlorins **H_2_C10-PEG_6_**, **H_2_C12-PEG_6_**, and **H_2_C15-PEG_6_** as well as zinc chlorins **ZnC12-PEG_6_** and **ZnC15-PEG_6_** each bear a single carboxylic acid and a tri-PEGylated aryl group attached to the chlorin 10-position. This work extends prior research in preparing PEGylated chlorins [15]; alternative approaches for installation of PEG groups in hydroporphyrins-containing arrays have been described recently by Meares et al. [16]. Each PEG group consists of six ethyleneoxy units and is terminated with a methyl group. The PEG moieties are introduced via copper(I)-mediated azide–alkyne click chemistry [17] and hence are linked via a triazole unit. Two of the PEG groups project above/below the face of the macrocycle. Such facial encumbrance (illustrated in Figure 3) is a viable strategy for suppressing π–π aggregation of tetrapyrrole macrocycles [18]. The photophysical properties of the five chlorins have been examined in aqueous solution. One chlorin (**H_2_C12-PEG_6_**) has been converted to the *N*-hydroxysuccinimidyl ester for potential use in bioconjugation processes. Taken together, the molecular design, syntheses, and photophysical studies illustrate the ability to create analogues of chlorophyll tailored for specific applications.

## 2. Results and Discussion

### 2.1. Reconnaissance

We previously prepared a set of chlorins containing PEG groups for water-solubilization (Figure 3). Chlorins **I** and **II** each contain three PEG groups, which were installed by click chemistry with a trialkynyl-bearing chlorin. This design was attractive to us, and our goal here was to incorporate a tether containing a carboxylic-acid terminus for bioconjugation. In prior studies, chlorin **III** was converted to the *N*-hydroxysuccinimidyl (NHS) ester, but the latter was found to undergo rapid hydrolysis, so fast as to prohibit the bioconjugation.

In this regard, PEG–O–CH_2_CO_2_–NHS is reported to have a very short half-time (0.75 min) under mild conditions in aqueous solution (pH 8, 25 °C) as measured by liberation of the NHS group, to be compared with 16.5 or 23.3 min for the structure with one or two additional methylene units (PEG–O–CH_2_CH_2_CO_2_–NHS or PEG–O–CH_2_CH_2_CH_2_CO_2_–NHS), respectively, where “PEG” lengths here are unspecified [19]. The poor results we encountered with the aryloxyacetic acid tether prompted exploration of other designs: we first examined a tether derived from succinic acid and a *p*-arylamine, and subsequently turned to an arylpropionic acid tether.

**Scheme 1:**
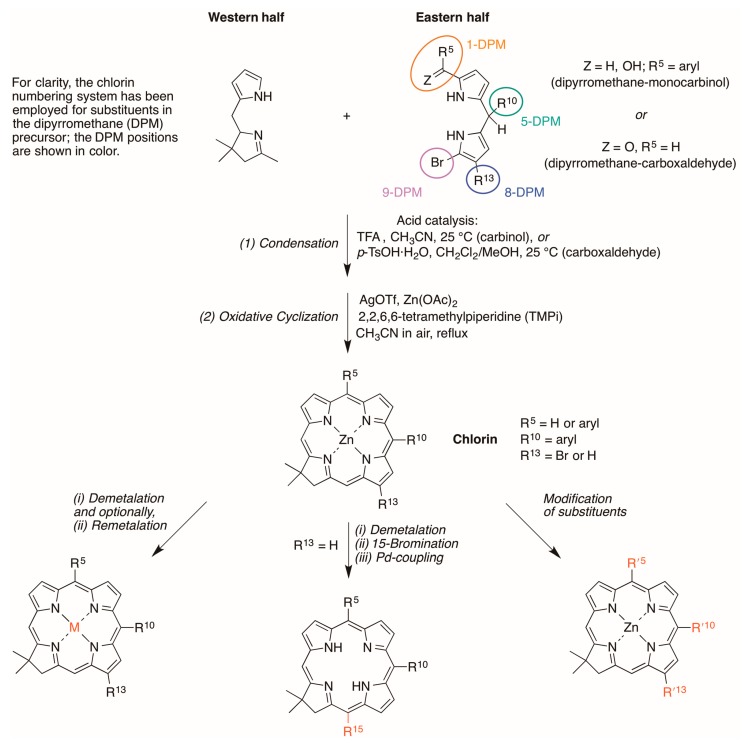
General routes for chlorin formation and derivatization.

The synthetic approach to chlorins relies on the reaction of a tetrahydrodipyrrin (Western half) and a 1,9-disubstituted dipyrromethane (Eastern half). The overall synthetic approach is shown in Scheme 1. Acid-catalyzed condensation followed by zinc(II)-mediated cyclization with accompanying dehydrogenation affords the corresponding zinc chlorin. Dipyrromethanes bearing 1-formyl-9-bromo substitution patterns afford the 5-unsubstituted chlorin, whereas the 1-aroyl substituted dipyrromethanes lead to the corresponding 5-arylchlorins. Each chlorin prepared herein bears a 10-aryl unit. Substitution at the 13-position is achieved by carrying through a bromo substituent in the Eastern half whereas substitution at the chlorin 15-position is accomplished by bromination of the free base 18,18-dimethylchlorin, which proceeds regioselectively at the 15-position [8]. In both cases, subsequent Pd-mediated substitution is employed to install the desired substituent at the perimeter of the macrocycle.

### 2.2. Synthesis of Chlorins

#### 2.2.1. Amino Substitution at the 5-Position

**Scheme 2:**
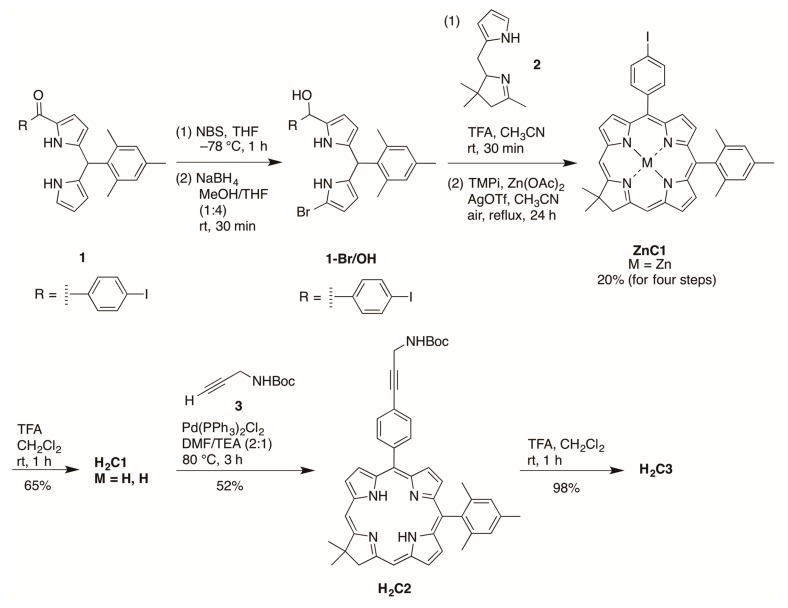
Synthesis of a chlorin equipped with a primary amine.

Zinc chlorin **ZnC1** was synthesized following an established route [20]. Bromination of the 9-position of the 1-acyldipyrromethane **1** [21] with NBS at −78 °C followed by reduction with NaBH_4_ in THF/MeOH afforded the corresponding 9-bromodipyrromethane-1-carbinol **1-Br/OH** (Scheme 2). Condensation of the resulting carbinol with the Western half **2** [22] in the presence of trifluoroacetic acid (TFA) followed by zinc-mediated cyclization gave the corresponding zinc chlorin **ZnC1** in 20% yield. Demetalation of **ZnC1** using TFA afforded the free-base chlorin **H_2_C1** in 65% yield. Copper-free palladium-mediated Sonogashira coupling [23,24] of **H_2_C1** and *N*-Boc-propargylamine afforded **H_2_C2** in 52% yield. Cleavage of the Boc protecting group using TFA gave the target bioconjugatable chlorin **H_2_C3** bearing a single primary amine in 98% yield.

#### 2.2.2. Chalcone or β-Ketoester Substitution at the 13-Position

**Scheme 3:**
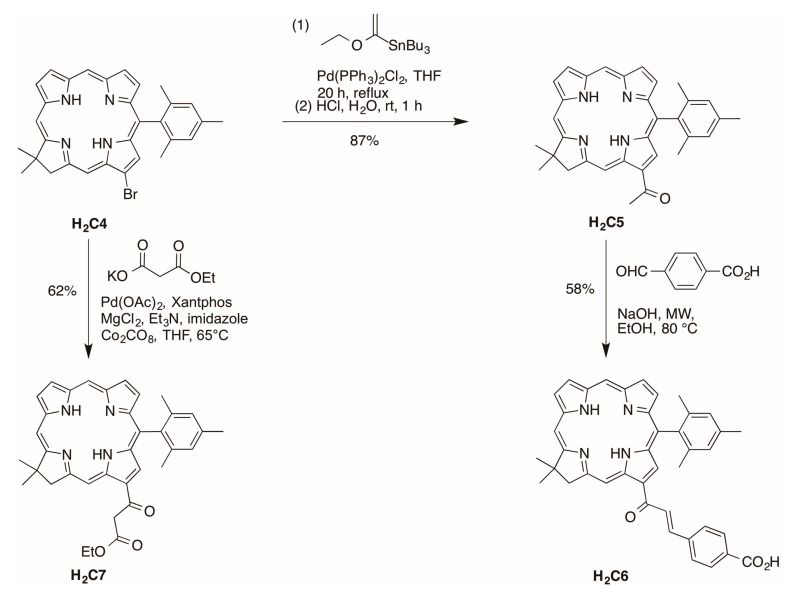
Synthesis of a chlorin bearing a β-ketoester or chalcone–carboxylic acid.

13-Bromochlorin **H_2_C4** has been prepared by de novo synthesis [25] and converted to the corresponding 13-acetylchlorin **H_2_C5** by Pd-mediated coupling in 87% yield [26] as shown in Scheme 3. Aldol condensation of **H_2_C5** and 4-formylbenzoic acid in ethanol under microwave condition [26] afforded the corresponding chlorin–chalcone **H_2_C6** bearing a bioconjugatable tether in 58% yield (Scheme 3).

The installation of a β-ketoester on a chlorin was conducted via Pd-mediated carbonylation [27]. Chlorin **H_2_C4** [25] reacted with potassium mono ethyl malonate, MgCl_2_, imidazole, Xantphos, Pd(OAc)_2_, Et_3_N, and Co_2_CO_8_ in THF at 65 °C over 16 h afforded **H_2_C7** in 62% yield (Scheme 3). The reaction conditions were modified compared with a reported procedure [27] in the following manner: (1) the amounts of both Pd(OAc)_2_ and Xantphos were increased from 0.1 equiv to 0.5 equiv, and (2) the reaction was carried out at 65 °C without microwave irradiation, as exploratory reactions illustrated that treatment of **H_2_C4** to 1 h of microwave irradiation under similar reaction conditions gave a cobalt derivative of **H_2_C4** rather than the desired **H_2_C7**.

#### 2.2.3. Carboxylic Acid (or NHS Ester) and Tris(PEGylation) at the Respective 15- and 10-Position

**Scheme 4:**
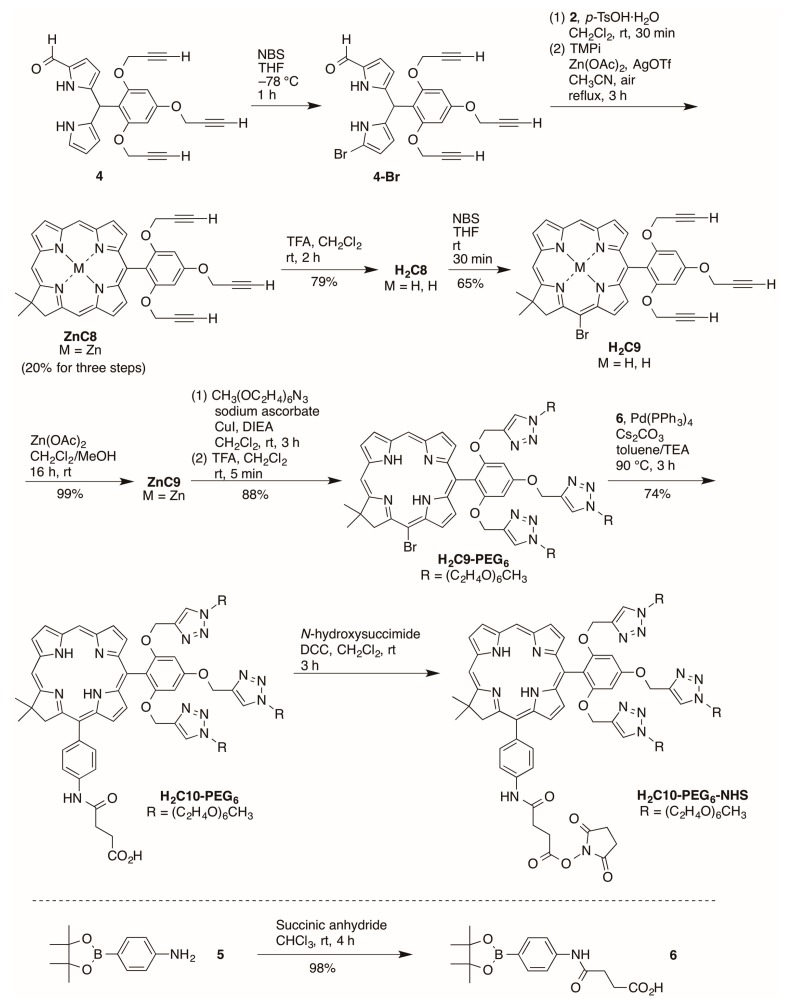
Synthesis of a PEGylated chlorin bearing a carboxylic acid.

A refined synthesis of known **ZnC8** [15] is shown in Scheme 4. 1-Formyldipyromethane **4** [15] was brominated selectively [28] at the 9-position with NBS at −78 °C in anhydrous THF for 1 h. The resulting crude 9-bromo-1-formyldipyrromethane **4-Br** was used directly in the condensation (without isolation as done previously) with Western half **2** in the presence of *p*-TsOH·H_2_O. Zinc-mediated cyclization in CH_3_CN under reflux in the presence of air for 3 h gave the target zinc chlorin **ZnC8** in 20% yield for the three steps. The yield was 20% here versus 10% previously.

Demetalation [29] with TFA afforded free base chlorin **H_2_C8** in 79% yield. Regioselective bromination at the 15-position [30] gave the 15-brominated chlorin **H_2_C9** in 65% yield. Zinc chelation [31] afforded **ZnC9** in 99% yield. Regioselective bromination was carried out on the free base chlorin instead of the zinc chelate due to challenges in purification of brominated zinc chlorins [30]. Zinc chelation is desired, however, to block copper insertion into the chlorin during the copper-catalyzed click reaction [15]. The copper(I)-catalyzed click reaction [32] of **ZnC9** and PEG-azide followed by demetalation using TFA gave the corresponding crude PEGylated free base chlorin. Purification by a 3-column chromatographic approach [33] including adsorption chromatography, size-exclusion chromatography (SEC), and adsorption chromatography again (to remove materials that leach from the SEC resin) afforded **H_2_C9-PEG_6_** in 88% yield for the two synthetic steps. Suzuki coupling partner **6** was prepared by reaction [34] of 2-(4-aminophenyl)-1,3-dioxa-4,4,5,5-tetramethylborolane and succinic anhydride. Pd-mediated Suzuki coupling [35,36] of **H_2_C9-PEG_6_** and **6** yielded target bioconjugatable PEGylated chlorin **H_2_C10-PEG_6_** in 74% yield.

**Scheme 5:**
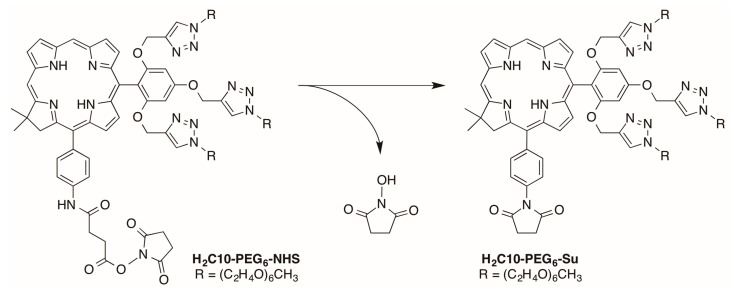
Undesired cyclization of **H_2_C10-PEG_6_-NHS**.

Attempted use of **H_2_C10-PEG_6_-NHS** in bioconjugation processes led to poor yields; subsequent LC-MS analysis showed a small peak due to **H_2_C10-PEG_6_-NHS** and a preceding peak of ~2.5 times greater intensity (Figure S1, panels A and B). Peak 1 gave *m*/*z* = 1716 whereas peak 2 gave the expected *m*/*z* = 1831 for **H_2_C10-PEG_6_-NHS** (Figure S1, panels C and D). The lower mass value is consistent with loss of *N*-hydroxysuccinimide (115 Da). The loss is attributed to cyclization of the activated carboxyl group (NHS-ester) to form a succinimidyl moiety at the *p*-aryl position (Scheme 5). Following this observation, we found literature reports of similar facile reactions with the methyl ester of simple anilide substrates (e.g., 3-(methoxycarbonyl)propionanilide) [37,38]. To avoid this side reaction, alternative designs for bioconjugation were considered such as use of an isothiocyanate as employed with tetrapyrroles by Sutton et al. [39]. Ultimately, because we wished to limit synthetic manipulations on the intact macrocycles, designs with a propionic acid directly attached to the aryl ring were pursued.

**Scheme 6:**
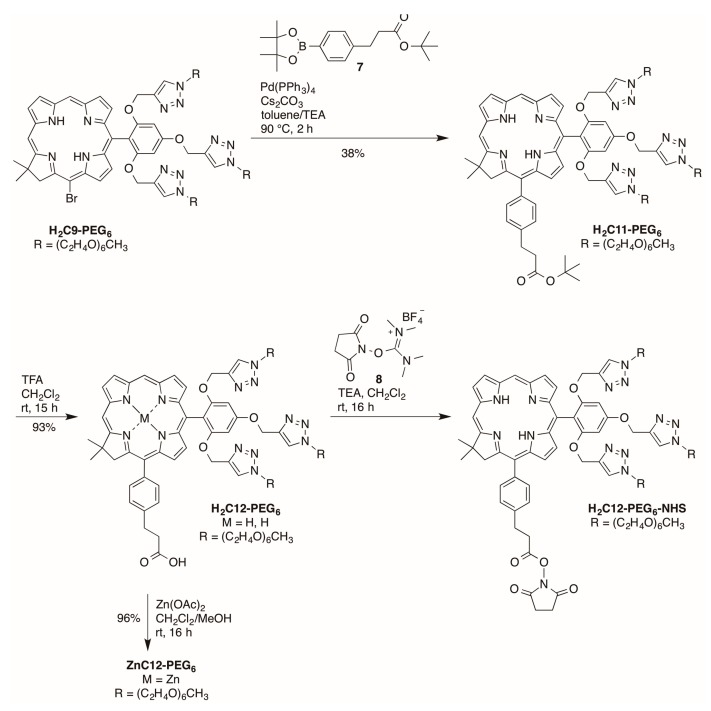
Synthesis of PEGylated chlorins bearing an arylpropionic acid.

Palladium-mediated Suzuki coupling [35,36] of PEGylated bromochlorin **H_2_C9-PEG_6_** with coupling partner **7** gave chlorin **H_2_C11-PEG_6_** with 38% yield (Scheme 6). Removal of the *tert*-butyl group with TFA afforded the corresponding chlorin **H_2_C12-PEG_6_** in 93% yield. Subsequently, **H_2_C12-PEG_6_** was converted to **ZnC12-PEG_6_** using zinc acetate in 96% yield. The carboxyl group on **H_2_C12-PEG_6_** was then converted to the *N*-succinimidyl ester upon use of **8** (2-succinimido-1,1,3,3-tetramethyluronium tetrafluoroborate, TSTU [40,41]) to afford the active ester **H_2_C12-PEG_6_-NHS** for protein conjugation. The reagent TSTU is an attractive, pre-activated entity for installation of the NHS ester and thus avoids use of an exogenous coupling agent such as DCC.

#### 2.2.4. Heptynoic Acid and Tris(PEGylation) at the Respective 13- and 10-Position

**Scheme 7:**
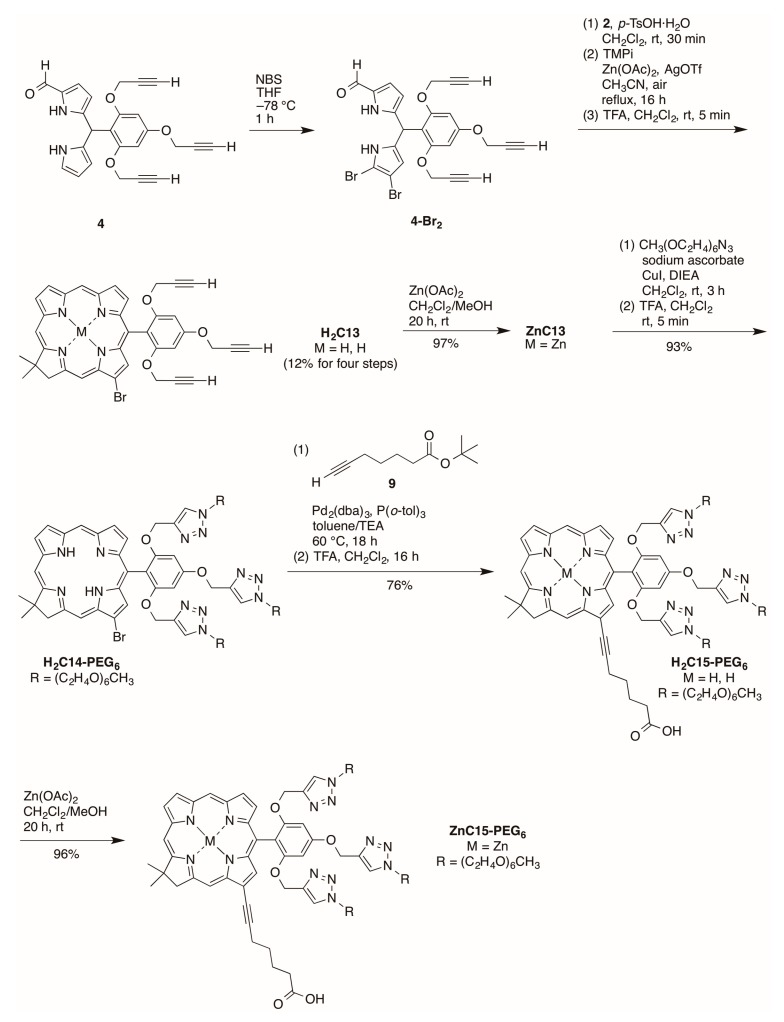
Synthesis of PEGylated chlorins bearing heptynoic acid at the 13-position.

Bromination of formyl dipyrromethane **4** [15] using 2 equivalents of NBS at −78 °C for 1 h afforded a mixture of 8,9-dibromo-1-formyldipyrromethane **4-Br_2_** and 9-bromo-1-formyldipyrro-methane **4-Br** [42] (Scheme 7). The mixture could not be separated given the instability [15] and similar polarity (upon analysis by TLC) of the two products. The resulting mixture was used directly in the condensation with Western half **2** for 30 min in the presence of *p*-TsOH·H_2_O. Zinc-mediated cyclization in CH_3_CN under reflux in the presence of air for 16 h gave a mixture of zinc chlorins with or without a bromo group at the 13-position of the macrocycle, as determined upon examination by matrix-assisted laser-desorption mass spectrometry (MALDI-MS) and ^1^H-NMR spectroscopy.

The ^1^H-NMR spectral properties of 13-substituted chlorins have been described previously [20]. The two zinc chlorins could not be separated using preparative chromatography. Therefore, the mixture was demetalated using TFA, and the resulting free base chlorins were separated by column chromatography. Chlorin **H_2_C13** bears a bromo substitution at the 13-position and was obtained in 12% overall yield for four steps. Zincation of **H_2_C13** gave zinc chlorin **ZnC13** in 97% yield. Copper(I)-catalyzed click reaction between **ZnC13** and PEG-azide followed by demetalation in the presence of TFA afforded the corresponding PEGylated chlorin **H_2_C14-PEG_6_** in 93% yield for the two steps. The palladium-mediated copper-free Sonogashira coupling reaction [43,44,45] of **H_2_C14-PEG_6_** and *tert*-butyl 6-heptynoate (**9**) [46] followed by removal of the *tert*-butyl group using TFA afford **H_2_C15-PEG_6_** in 76% yield. Zincation of **H_2_C15-PEG_6_** gave the corresponding zinc chlorin **ZnC15-PEG_6_** in 96% yield.

The new chlorins were characterized by absorption spectroscopy, ^1^H-NMR spectroscopy, ^13^C-NMR spectroscopy (where possible) and electrospray ionization mass spectrometery (ESI-MS). Chlorins **H_2_C2**, **H_2_C3**, **ZnC8**, **H_2_C9**, **ZnC9**, **H_2_C9-PEG_6_**, **H_2_C10-PEG_6_-NHS**, **H_2_C12-PEG_6_-NHS** and **ZnC13** were also characterized by MALDI-MS. Exceptions to the general characterization protocol included the following: (1) chlorins **H_2_C10-PEG_6_-NHS** and **H_2_C12-PEG_6_-NHS** were only characterized by MALDI-MS; and (2) ^13^C-NMR spectra were not obtained for **H_2_C6**, **H_2_C11-PEG_6_**, **H_2_C12-PEG_6_**, **ZnC12-PEG_6_**, **H_2_C15-PEG_6_** and **ZnC15-PEG_6_** owing to limited solubility. The synthetic chlorins also were subjected to photophysical examination, as described in the next section.

### 2.3. Photophysical Characterization

Photophysical studies were carried out with several chlorins. Chlorin **H_2_C10-PEG_6_** was examined for ground-state absorption and singlet-excited-state properties (fluorescence, intersystem-crossing and internal-conversion) in solvents of diverse polarity including toluene, DMF, and water. Amphiphilic chlorins **H_2_C_2_**, **H_2_C3** and **H_2_C6** were examined for absorption, fluorescence and excited-state characteristics in toluene, DMF and aqueous micellar solution. Finally, chlorin **H_2_C12-PEG_6_-NHS** was coupled with the protein avidin to make chlorin–avidin conjugates, which were examined for absorption and fluorescence properties in aqueous solution. The studies illustrate the utility of molecular design and synthesis in tailoring chlorins for compatibility with diverse environments.

#### 2.3.1. Spectral and Photophysical Properties of PEGylated Chlorins

**Figure 4:**
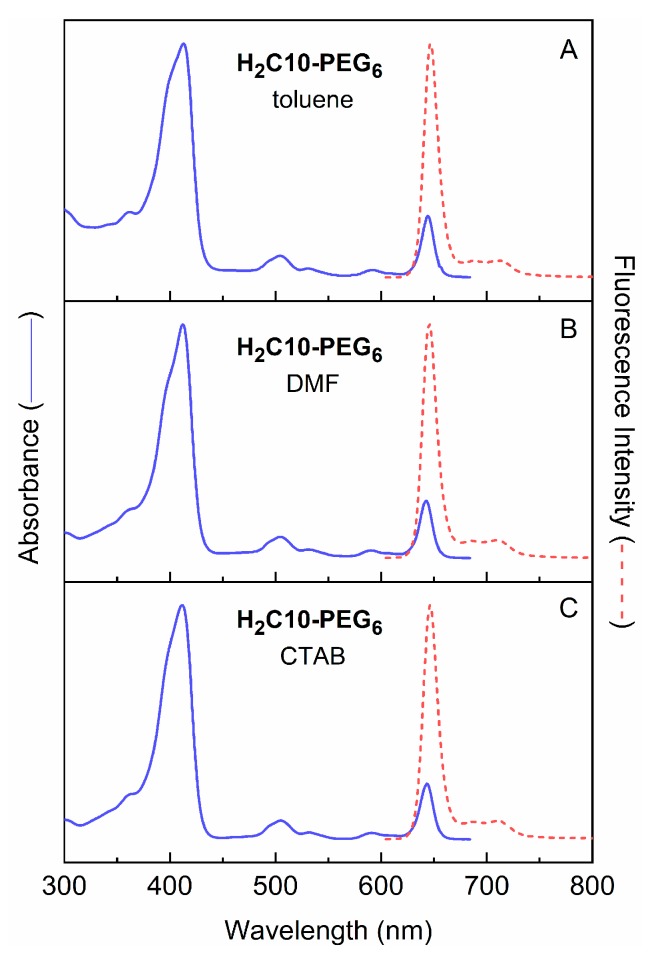
Absorption and emission spectra of **H_2_C10-PEG_6_** in toluene, DMF and water.

Chlorin **H_2_C10-PEG_6_** was characterized by static absorption and emission spectroscopy at room temperature in three media of diverse polarity: toluene, DMF and water. The absorption and fluorescence spectra are shown in Figure 4. The spectral characteristics are summarized in Table 1. The prominent fluorescence emission band in each case is sharp, as assessed by the full-width-at-half maximum (fwhm).

**Figure 5:**
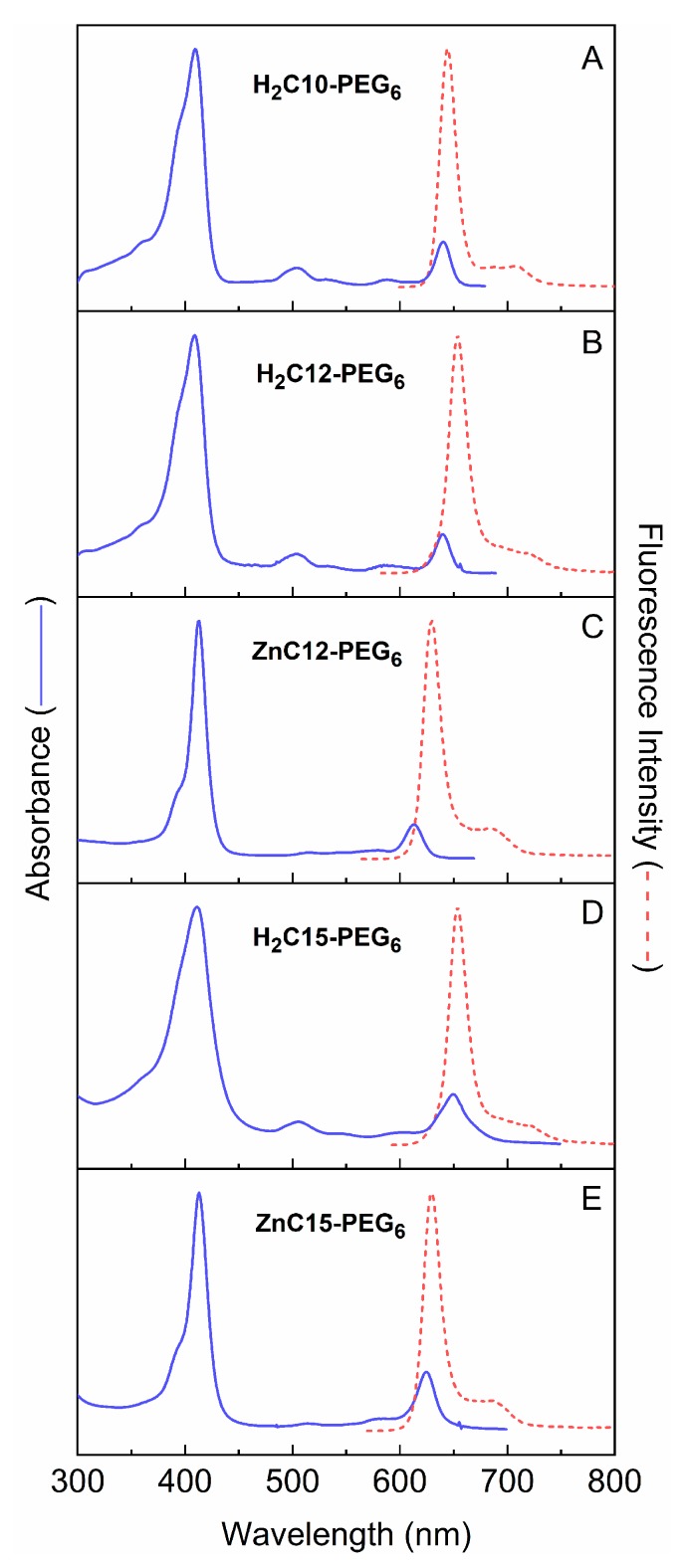
Absorption and emission spectra of the five PEGylated chlorins in water.

The absorption spectra of all five PEGylated chlorins in water are given in Figure 5. The absorption spectra show the Q_y_ (S_0_ → S_1_) absorption in the red region, the Q_x_ (S_0_ → S_2_) band in the green, and overlapping B_x_ and B_y_ (S_0_ → S_3_ and S_0_ → S_4_) features in the near-UV. Each of these bands has one or more vibronic satellite features to higher energy. The fluorescence spectrum is approximately mirror symmetric to the Q_y_ absorption manifold and contains the prominent (0, 0) band at ~650 nm and the weaker (1, 0) feature at ~710 nm.

**Figure 6:**
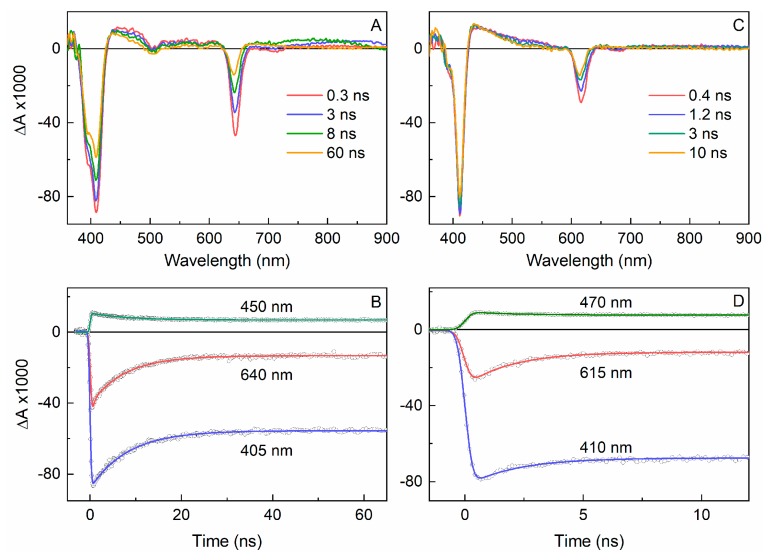
Transient absorption data for **H_2_C12-PEG_6_** (left, panels **A** and **B**) and **ZnC12-PEG_6_** (right, panels **C** and **D**) and in aqueous PBS solution obtained using 100 fs excitation flashes at 640 nm in the presence of ambient O_2_.

Table 1 also summarizes the spectral and excited-state properties for the two PEGylated chlorins **H_2_C12-PEG_6_** and **H_2_C15-PEG_6_** in water as well as the analogous zinc chelates **ZnC12-PEG_6_** and **ZnC15-PEG_6_** in water, toluene and DMF. Figure 6A shows transient absorption data for **H_2_C12-PEG_6_** in aqueous solution, illustrating the measurement of τ_S_ and Φ_isc_ (for all the chlorins). The absorption difference spectrum at 0.3 ns contains features expected for the first S_1_ excited state. These features include bleaching of the near-UV (Soret) ground-state absorption (combined S_0_ → S_3_ and S_0_ → S_4_) at 405 nm and a feature at 640 nm that contains bleaching of the S_0_ → S_1_ absorption along with S_1_ → S_0_ emission stimulated by the white-light probe pulse. The spectrum at 60 ns can be assigned to the lowest triplet excited state (T_1_). At this time, the stimulated emission contribution has disappeared and the magnitudes of S_0_ → S_n_ bleachings have decreased because some molecules have returned to the ground state (by S_1_ → S_0_ fluorescence and internal conversion) in parallel with formation of T_1_. The yield of S_1_ → T_1_ intersystem crossing is obtained by comparing the bleaching magnitudes (referenced to the relatively weak featureless excited-state absorption) at long times (due to T_1_) compared to immediately after the excitation flash (due to S_1_). The value for **H_2_C12-PEG_6_** in aqueous solution is Φ_isc_ = 0.70 (Table 1). Figure 6B shows the decay of the S_1_ excited state probed at three representative wavelengths, which together with global fitting of the entire date set afford τ_S_ = 7.6 ns.

Figure 6C,D give analogous transient absorption data for the analogous zinc chelate **ZnC12-PEG_6_**. The data differ from that for **H_2_C12-PEG_6_** in aqueous solution in several respects (Table 1): a shorter excited-state lifetime (τ_S_ = 1.9 ns vs. 7.6 ns), a larger triplet yield (Φ_isc_ = 0.80 vs. 0.70) and a smaller fluorescence yield (Φ_f_ = 0.060 vs. 0.23). These differences can be traced primarily to the heavy atom (zinc) effect on spin-orbit coupling and thus the rate constant for S_1_ → T_1_ intersystem crossing [k_isc_ = (2.5 ns)^−1^ vs. (11 ns)^−1^]. Similar comparisons can be made concerning the photophysical properties of **ZnC15-PEG_6_**. The data differ from that for **H_2_C15-PEG_6_** in water (Table 1). It is also interesting that the rate constants for S_1_ → S_0_ internal conversion for **ZnC12-PEG_6_** and **ZnC15-PEG_6_** are greater than those for the free base analogues **H_2_C12-PEG_6_** and **H_2_C15-PEG_6_**. Generally one expects the opposite to be true because of the involvement of N-H vibrations associated with the central free base protons to enhance the Franck-Condon factor for this non-radiative decay process. Here, internal conversion may be facilitated by structural effects (e.g., out-of-plane-distortions) or vibrational effects of water molecules coordinated to the central zinc ion. However, internal conversion has approximately the same rate and yield for the two zinc chelates in toluene or DMF as in water (Table 1). Perhaps axial-coordination effects occur but involve instead interaction of the central zinc of one chlorin with the PEG groups of an adjacent zinc chlorin.

#### 2.3.2. Amphiphilic Chlorins in Various Solvents Including Micellar Solution

**Figure 7:**
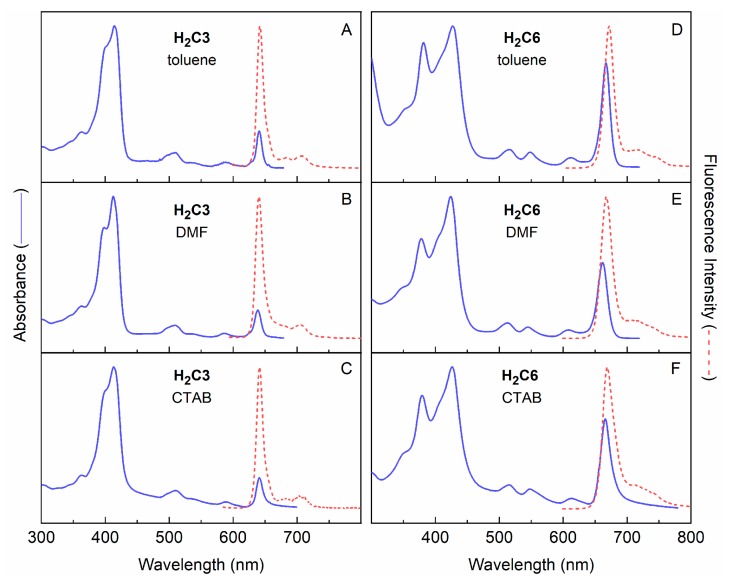
Absorption and emission spectra of **H_2_C3** and **H_2_C6** in toluene DMF and CTAB micelles.

Chlorins **H_2_C2**, **H_2_C3** and **H_2_C6** were characterized by static absorption and emission spectroscopy and transient absorption spectroscopy at room temperature in three media. The media included toluene, DMF and aqueous micellar solution; the latter entailed cetyl trimethylammonium bromide (CTAB) at 10 mM in aqueous sodium phosphate buffer (pH 7, 0.1 M). The absorption and fluorescence spectra of **H_2_C3** and **H_2_C6** in the three media are shown in Figure 4. The spectra of **H_2_C2** are nearly very similar to those for **H_2_C2**; the two molecules differ only in the nature of the terminal amine (-NHBOC vs. -NH_2_). The spectral properties of the three chlorins are summarized in Table 2 along with values for τ_S,_ Φ_f_, Φ_isc_ and Φ_ic_ in toluene, DMF and aqueous CTAB micelle solution. Noteworthy observations include the bathochromic and hyperchromic effects on the Q_y_ absorption of **H_2_C6** relative to **H_2_C3** (Figure 7). These coupled effects on wavelength and intensity of the S_0_ → S_1_ (Q_y_) transition are consistent with the four-orbital model, as we have described previously for the effects of auxochromic substituents on chlorin spectra [47]. The larger intensity of the Q_y_ absorption of **H_2_C6** versus **H_2_C3** is accompanied by an increase in radiative rate constant k_f_ for spontaneous emission (Table 1) because of the relationship of the Einstein coefficients for the two processes [48].

#### 2.3.3. Poisson Distribution of **H_2_C3** and **H_2_C6** in CTAB Micelle Solution

Understanding the properties of chromophores in aqueous micellar media requires estimation of the number of chromophores per micelle. If the distribution of chromophores is random and not affected by attraction (e.g., aggregation) or repulsion, the Poisson equation (Equation (1)) [49,50] provides an appropriate description for the distribution of molecular species in the micelles. Here, m is the mean occupancy number of chromophore per micelles, and k takes on integer values for the number of chromophores per micelle (k = 0, 1, 2…):

P(x = k) = m^k^ · e^−m^/k! (1)

The average aggregation number for 10 mM CTAB in aqueous solution at room temperature is about 95 [51]. The mean occupancy number varies depending on the concentration of micelles; given a critical micelle concentration (cmc) of 1 mM for CTAB [52] and chlorin concentration of 3.1 µM (**H_2_C3)** or 6.4 µM (**H_2_C6**), the mean occupancy of **H_2_C3** or **H_2_C6** per micelle is very low: 0.033 or 0.067, respectively.

**Figure 8:**
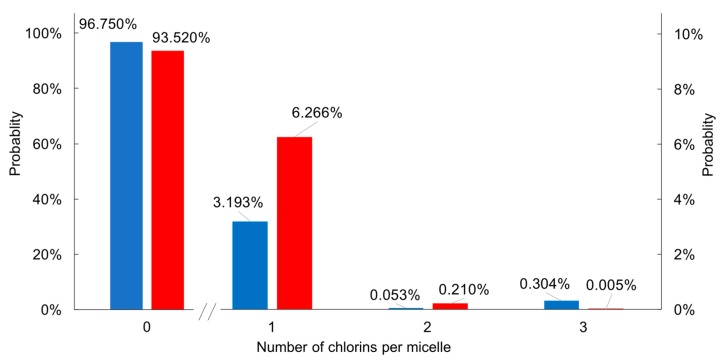
Poisson distribution of numbers of chlorins (**H_2_C3** in blue; **H_2_C6** in red) per micelle.

The Poisson distribution histograms of **H_2_C3** and **H_2_C6** in 10 mM CTAB micelle solution are shown in Figure 8. Inspection shows that most micelles are empty, and among those that contain a chlorin, relatively few (1.8% or 3.2% for **H_2_C3** or **H_2_C6**, respectively) contain more than one chlorin. Prior treatments of Poissonian distributions of tetrapyrroles in micelles include bacteriochlorophyll *a* [53] or chlorophyll *a* [54] in Triton-X100 micelles; octaalkylporphyrins in cetyl trimethylammonium chloride or sodium dodecyl sulfate (SDS) [55]; chlorophyll *a* (and derivatives thereof) [56] in Triton-X100, sodium bis(2-ethylhexyl)sufosuccinate (AOT) or SDS [57]; and synthetic chlorins and bacteriochlorins in Triton-X100, CTAB or SDS [13]. The topic of porphyrins in membranes has been reviewed [58,59]. The distributions of tetrapyrroles in micelles should not be regarded as static, given that hydrophobic solutes typically undergo extensive exchange among micelles; for example, substrates such as dodecylpyrene undergo extensive exchange in minutes–hours among SDS micelles [60]. The exchange of solutes between micelles [55,61,62,63,64,65] can entail exchange through the aqueous solution, fusion–fission of micelles, and/or micellar fission. The prevalence of a given mechanism depends on the micellar surface charge, aqueous ionic strength, and solute polarity.

#### 2.3.4. **H_2_C12-PEG_6_-NHS** Avidin Loading Experiment

**Figure 9:**
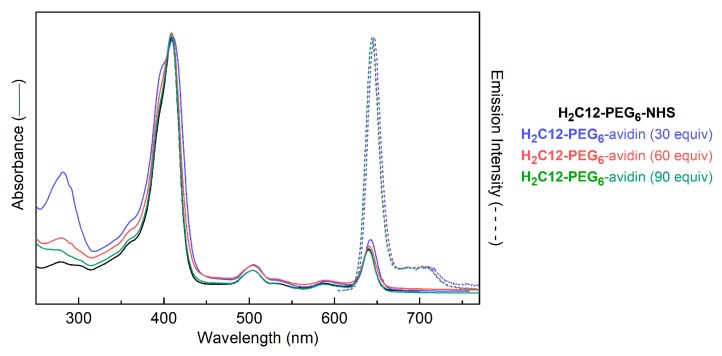
Absorption (solid lines) and emission (dashed lines) spectra of **H_2_C12-PEG_6_**-avidin conjugates (prepared with 30, 60 or 90 equiv of **H_2_C12-PEG_6_-NHS** per avidin) as well as chlorin **H_2_C12-PEG_6_-NHS** in aqueous solution at room temperature (λ_exc_ = 505 nm). The spectra are normalized at the maximum value.

To gain insight into how fluorescence quantum yields of chlorin–protein conjugates vary with the chlorin/protein ratio, we carried out exploratory studies of chlorin–avidin conjugates. The conjugates were prepared with increasing equivalents of chlorin/avidin. Avidin is a 68,000 Da tetrameric protein from egg white and has high binding affinity for biotin (K_D_ = 10^−15^ M) [66,67,68,69,70]. There are nine lysine residues in each subunit of avidin (36 lysines for tetrameric avidin) for possible bioconjugation with an activated carboxyl group [71]. All subsequent usage of “avidin” here refers to the tetramer. The conjugation was carried out by reaction of a solution of **H_2_C12-PEG_6_-NHS** (0.42 mg, 0.84 mg, or 1.26 mg for 30, 60 or 90 equiv.) with avidin (0.5 mg, 7.9 nmol) in 0.1 M sodium phosphate buffer (100 µL total volume, pH 7.6) for 16 h at room temperature. The chlorin–avidin conjugate in each case was purified by (1) passage through a gel permeation chromatography column (molecular weight cut-off at 5000 Da) to obtain the chlorin–avidin conjugate free from the excess chlorin (which remains bound to the top of the column); and (2) concentration of the chlorin–avidin conjugate and further liberation from any residual excess chlorin by mass-selective centrifugation through a filter (molecular weight cut-off at 30,000 Da) at 4000 rpm for 5 min. The resulting chlorin–avidin conjugate was analyzed by absorption spectroscopy and fluorescence spectroscopy (vide infra). Analysis of the absorption spectrum in each case revealed the number of chlorins attached per avidin. For the reactions of 30, 60, or 90 equiv. of **H_2_C12-PEG_6_-NHS** relative to avidin, the loading (number of chlorins attached per avidin) to be 2.3, 6.2, or 12, respectively (Table 3). The loading values are estimates, yet the trend of increased chlorin/avidin with increased equiv of chlorin–NHS ester reactants is unmistakable (see Figure 9), as observed in the relative ratio of the intensities of absorption in the Soret band (where the chlorin absorbs exclusively) versus that at 280 nm (where the molar absorption coefficient of avidin exceeds that of the chlorin). Finally, it warrants emphasis that the observed loadings are average values, and as in the case of bacteriochlorin–myoglobin conjugates [36], represent a mixture composed of conjugates wherein the number of chlorins/avidin spans a very broad range.

Attempts to use MALDI-MS to characterize the chlorin–avidin conjugates were not successful using various matrices including α-cyano-4-hydroxycinnamic acid (CHCA), sinapinic acid, and the recommended sinapinic acid with ammonium citrate at pH 7 [72]). We attribute the failure in this case to the large number of PEG groups in the sample, given that (1) native avidin was successfully analyzed by MALDI-MS [72]; and (2) MALDI-MS data were readily obtained of bacteriochlorin–myoglobin conjugates wherein each bacteriochlorin was substituted with four carboxylic acid groups [36]. We note that characterization of the products of dye–protein conjugation often is quite limited; regardless, given the available data, the identity of the chlorin–avidin conjugates prepared herein must be regarded as provisional.

The absorption and emission spectra of the conjugates are shown in Figure 9. The absorption spectra were normalized at the near-UV (Soret or B) maximum of the chlorin. As the chlorin loading increases, the normalized spectra show an apparent decrease in the UV protein feature near 290 nm (relative to the chlorin features). However, the chlorin Q_y_ absorption and fluorescence features do not change shape. The only change in the chlorin absorption is a small increase on the short-wavelength side of the Soret band, potentially reflecting some modest dipole–dipole interaction among nearby chlorins. With increased loading over the ~5-fold range explored here, the fluorescence quantum yield Φ_f_ increases by ~2-fold. Note that the 2-fold increase pertains to the Φ_f_ value, not the emission across constant protein concentrations; hence, the relative brightness per protein (given by the number of chlorins/avidin × Φ_f_ per chlorin) increases by ~12-fold. Further studies are required to explore the origin of this unexpected increase in brightness with increased loading.

#### 2.3.5. Comparison of Absorptance Spectra and Fluorescence Excitation Spectra

**Figure 10:**
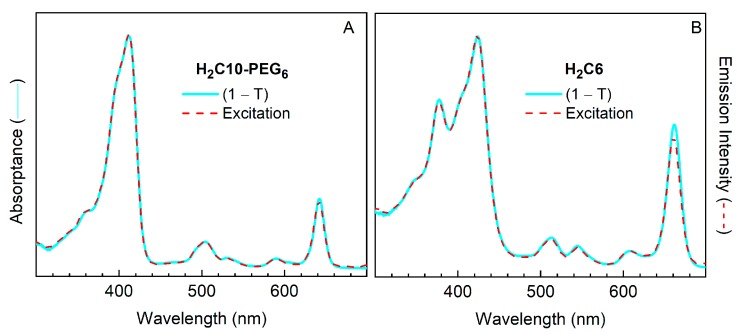
Comparison of absorptance (1 − T, where T is transmittance) spectra (solid cyan) and fluorescence excitation spectra (dashed red) for **H_2_C10-PEG_6_** (**A**) and **H_2_C6** (**B**) in DMF, normalized at the near-UV (Soret) maximum. A very small Rayleigh light-scattering correction was applied to the absorptance spectra. For each compound, the fluorescence was detected in the Q_y_(0,1) feature at 700–710 nm, although the same spectrum was obtained at other detection wavelengths.

Figure 10 compares the absorptance (1 − T, where T is transmittance) spectra and fluorescence excitation spectra for a PEGylated chlorin (**H_2_C10-PEG_6_**) and a non-PEGylated benchmark chlorin (**H_2_C_6_**) in DMF. There is a near-perfect match between the two types of spectra for each compound. These data reflect the high purity of the compounds, and indicate that the fluorescence originates from the target compound and is not affected by the presence of any highly fluorescent trace impurities. The absorptance (and absorption) spectra show no signs of broadening or peak shifts relative to the excitation spectrum that could arise from aggregation for either compound. Note that aggregates with strictly face-to-face interactions (e.g., H-type dimers) generally would not result in extra features in the fluorescence excitation not already present in the absorptance spectra. Such aggregates are typically non-fluorescent or weakly fluorescent because the exciton splitting for each state will have the lower-exciton component dipole-forbidden and the upper exciton component dipole-allowed. In this regard, the spectra in Figure 10 show no evidence for aggregates or other contaminants.

## 3. Materials and Methods

### 3.1. General Methods

All chemicals obtained commercially were used as received unless otherwise noted. Reagent-grade solvents (CH_2_Cl_2_, hexanes, methanol, toluene, ethyl acetate) and HPLC-grade solvents (toluene, CH_2_Cl_2_, hexanes) were used as received. THF was freshly distilled from sodium/benzophenone ketyl and used immediately. MALDI-MS was performed with the matrix 1,4-bis(5-phenyl-2-oxaxol-2-yl)benzene (POPOP) [73] unless noted otherwise. Accurate-mass (ESI-MS) data are reported for the molecular ion or cationized molecular ion. Noncommercial compounds **1** [21], **2** [22], **4** [15], **7** [36], **9** [46], **H_2_C4** [25] and **H_2_C5** [26] were prepared following literature procedures. Absorption spectra were measured with Agilent 8453 (Santa Clara, CA, USA) and Shimadzu UV1800 (Columbia, MD, USA) instruments using dilute (µmolar) solutions of the compound in UV transparent (e.g., quartz) cuvettes versus a solvent blank at room temperature.

### 3.2 Synthesis

*4-Oxo-4-[(4-(4,4,5,5-tetramethyl-1,3,2-dioxaborolan-2-yl)phenyl)amino]butanoic acid* (**6**). Following a reported procedure [34], a solution of **5** (100 mg, 0.456 mmol) in CHCl_3_ (18.7 mL) was treated with succinic anhydride (49.7 mg, 0.497 mmol), the resulting mixture was stirred at room temperature for 4 h. Then the mixture was diluted with ethyl acetate, washed with brine, dried (Na_2_SO_4_) and concentrated to afford a light yellow solid (142 mg, 98%): ^1^H-NMR (CDCl_3_, 300 MHz, the CO_2_H and amide NH proton peaks were not observed) δ 7.76 (d, *J* = 8.1 Hz, 2H), 7.50 (d, *J* = 8.1 Hz, 2H), 2.79–2.77 (m, 2H), 2.73–2.62 (m, 2H), 1.33 (s, 12H); ^13^C-NMR (CD_3_OD, 100 MHz) δ 175.0, 171.5, 141.4, 135.2, 118.5, 83.6, 48.1, 47.9, 47.7, 47.5, 47.3, 31.1, 28.6, 23.9, 23.7; ESI-MS obsd 320.1660 [M + H]^+^, calcd 320.1664 (M = C_16_H_22_BNO_5_).

*Zn(II)-5-(4-Iodophenyl)-10-mesityl-18,18-dimethylchlorin* (**ZnC1**). Following a reported procedure [20], a solution of **1** (150 mg, 0.30 mmol) in anhydrous THF (3 mL) was treated with NBS (54 mg, 0.30 mmol) at −78 °C. The reaction mixture was stirred at −78 °C for 1 h and then allowed to warm to 0 °C, whereupon water and hexanes was added to quench the reaction. The organic phase was diluted with CH_2_Cl_2_, washed with brine, dried (Na_2_SO_4_) and concentrated. The resulting product was dissolved in THF/MeOH (6 mL, 4:1) and treated with NaBH_4_ (113 mg, 2.99 mmol) in three portions at room temperature. The mixture was stirred at room temperature for 30 min, whereupon saturated NH_4_Cl aqueous solution was added to quench the reaction. The resulting product (**1-Br/OH**) was extracted with CH_2_Cl_2_, washed with water, dried (Na_2_SO_4_), and concentrated to a volume of 2.0 mL. Then CH_3_CN was added, the solution was concentrated to 2.0 mL and further diluted to 3.0 mL with CH_3_CN. A sample of **2** (56 mg, 0.29 mmol) was added followed by TFA (23 µL, 0.30 mmol). The mixture was stirred at room temperature for 30 min, whereupon CH_3_CN (27 mL) was added to the solution. Then samples of 2,2,6,6-tetramethylpiperidine (0.76 mL, 4.5 mmol), Zn(OAc)_2_ (0.83 g, 4.5 mmol) and AgOTf (231 mg, 0.899 mmol) were added in succession. The mixture was heated to reflux exposed to air for 24 h. After allowing to cool, the mixture was passed through a silica pad (CH_2_Cl_2_). The filtrated was concentrated and chromatographed [silica gel, hexanes/CH_2_Cl_2_ (2:1)] to afford a dark green solid (43.1 mg, 20%): ^1^H-NMR (CDCl_3_, 300 MHz) δ 8.65–6.64 (m, 1H), 8.62–8.60 (m, 2H), 8.57–8.55 (m, 2H), 8.50 (d, *J* = 4.5, 1H), 8.31 (d, *J* = 4.4, 1H), 8.24 (d, *J* = 4.4, 1H), 7.99 (d, *J* = 8.2 Hz, 2H), 7.80 (d, *J* = 8.2 Hz, 2H), 7.20 (s, 2H), 4.49 (s, 2H), 2.57 (s, 3H), 2.01 (s, 6H), 1.84 (s, 6H); ^13^C-NMR (CDCl_3_, 75 MHz) δ 171.2, 159.8, 154.3, 153.8, 147.5, 146.9, 146.2, 145.7, 142.4, 139.0, 138.8, 137.4, 136.02, 135.96, 135.5, 133.0, 132.5, 129.0, 127.82, 127.73, 127.62, 127.3, 122.76, 122.71, 96.9, 94.9, 93.8, 50.5, 45.5, 31.2, 21.7, 21.4; ESI-MS obsd 722.0861 [M]^+^, calcd 722.0879 (M = C_37_H_31_IN_4_Zn); λ_abs_ (toluene) 403, 504, 568, 613 nm.

*5-(4-Iodophenyl)-10-mesityl-18,18-dimethylchlorin* (**H_2_C1**). Following a standard procedure [29], a solution of **ZnC1** (15.7 mg, 217 µmol) in CH_2_Cl_2_ (10.9 mL) was treated with TFA (83 µL, 1.1 mmol). The mixture was stirred at room temperature for 1 h, whereupon triethylamine and water were slowly added to quench the reaction. The organic phase was washed with brine, dried (Na_2_SO_4_), concentrated and chromatographed [silica gel, hexanes/CH_2_Cl_2_ (2:1)] to afford a purple solid (9.3 mg, 65%): ^1^H-NMR (CDCl_3_, 300 MHz) δ 8.91 (s, 1H), 8.84 (s, 1H), 8.81 (d, *J* = 4.8 Hz, 1H), 8.73–8.70 (m, 2H), 8.59 (d, *J* = 4.8 Hz, 1H), 8.41 (d, *J* = 4.5 Hz, 1H), 8.35 (d, *J* = 4.4 Hz, 1H), 8.02 (d, *J* = 8.2 Hz, 2H), 7.85 (d, *J* = 8.2 Hz, 2H), 7.22 (s, 2H), 4.57 (s, 2H), 2.59 (s, 3H), 2.03 (s, 6H), 1.84 (s, 6H), −1.85 (br, 2H); ^13^C-NMR (CDCl_3_, 75 MHz) δ 175.2, 163.7, 152.4, 151.7, 141.9, 141.0, 140.5, 139.3, 138.4, 137.7, 136.1, 135.9, 134.7, 134.6, 132.3, 131.2, 128.3, 127.9, 123.8, 123.6, 120.63, 120.56, 96.8, 95.0, 94.1, 51.9, 46.7, 31.4, 21.7, 21.6; ESI-MS obsd 660.1728 [M]^+^, calcd 660.1744 (M = C_37_H_33_IN_4_); λ_abs_ (toluene) 414, 508, 534, 590, 641 nm.

*5-[4-(3-((tert-Butoxycarbonyl)amino)prop-1-ynyl)phenyl]-10-mesityl-18,18-dimethylchlorin* (**H_2_C2**). A reported procedure [23,24] was followed. A mixture of DMF/triethylamine (2:1, *v*/*v*) was deaerated with a continuous stream of argon for 1 h. Samples of **H_2_C1** (11 mg, 17 µmol), *N*-Boc-propargylamine (5.1 mg, 34 µmol) and Pd(PPh_3_)_2_Cl_2_ (1.2 mg, 1.7 µmol) were placed into a Schlenk flask, the contents of which were then degassed under high vacuum for 20 min. A solution of the deaerated DMF/triethylamine solution (5.3 mL, 2:1, *v*/*v*) was added to the flask. The mixture was degassed by three freeze-pump-thaw cycles. The resulting mixture was stirred at 80 °C for 3 h. The mixture was allowed to cool to room temperature. The cooled mixture was diluted with CH_2_Cl_2_, washed with water, dried (Na_2_SO_4_), concentrated and chromatographed [silica gel, hexanes/CH_2_Cl_2_ (1:2) to (1:3)] to afford a dark brown solid (6.1 mg, 52%): ^1^H-NMR (CDCl_3_, 400 MHz) δ 8.94 (s, 1H), 8.86 (s, 1H), 8.83 (d, *J* = 4.7 Hz, 1H), 8.75 (d, *J* = 4.7 Hz, 1H), 8.70 (d, *J* = 4.7 Hz, 1H), 8.60 (d, *J* = 4.7 Hz, 1H), 8.38 (d, *J* = 4.5 Hz, 1H), 8.33 (d, *J* = 4.4 Hz, 1H), 8.07 (d, *J* = 7.6 Hz, 1H), 7.78 (d, *J* = 7.9 Hz, 1H), 7.65–7.61 (m, 2H), 7.23 (s, 2H), 4.80 (br, 1H), 4.61 (s, 2H), 4.21–4.19 (m, 2H), 2.60 (s, 3H), 2.06 (s, 6H), 1.84 (s, 6H), 1.57 (s, 9H), −1.86 (br, 2H); ^13^C-NMR (CDCl_3_, 100 MHz) δ 175.2, 163.6, 140.4, 139.3, 138.4, 137.7, 137.0, 134.78, 134.66, 134.2, 132.4, 131.1, 127.9, 126.9, 123.7, 123.5, 121.4, 96.8, 95.0, 51.9, 46.7, 31.4, 28.6, 21.69, 21.55; MALDI-MS obsd 688.4; ESI-MS obsd 688.3627 [M + H]^+^, calcd 688.3646 (M = C_45_H_45_N_4_O_2_); λ_abs_ (toluene) 415, 509, 534, 590, 641 nm.

*5-[4-(3-Aminoprop-1-ynyl)phenyl]-10-mesityl-18,18-dimethylchlorin* (**H_2_C3**). Following a standard procedure [29], a solution of **H_2_C2** (6.0 mg, 8.7 µmol) in CH_2_Cl_2_ (1.2 mL) was treated with TFA (0.23 mL, 3.0 mmol). The mixture was stirred at room temperature for 1 h, whereupon triethylamine and water were slowly added to quench the reaction. The organic phase was washed with brine, dried (Na_2_SO_4_), concentrated and chromatographed (silica gel, CH_2_Cl_2_ with 1% triethylamine) to afford a brown solid (5.0 mg, 98%): ^1^H-NMR (CDCl_3_, 400 MHz) δ 8.94 (s, 1H), 8.87 (s, 1H), 8.84 (d, *J* = 4.8 Hz, 1H), 8.75 (d, *J* = 4.7 Hz, 1H), 8.72 (d, *J* = 4.8 Hz, 1H), 8.61 (d, *J* = 4.7 Hz, 1H), 8.39 (d, *J* = 4.4 Hz, 1H), 8.35 (d, *J* = 4.5 Hz, 1H), 8.20 (s, 1H), 8.08 (d, *J* = 7.6 Hz, 1H), 7.79 (d, *J* = 7.9 Hz, 1H), 7.64 (t, *J* = 7.7 Hz, 1H), 7.24 (s, 2H), 4.82 (br, 2H), 4.61 (s, 2H), 4.22–4.20 (m, 2H), 2.61 (s, 3H), 2.07 (s, 6H), 1.85 (s, 6H), −1.83 (br, 2H); ^13^C-NMR (CDCl_3_, 100 MHz) δ 163.6, 141.0, 140.4, 139.3, 137.7, 136.9, 134.8, 132.4, 131.1, 128.5, 128.97, 127.88, 127.0, 123.70, 123.56, 96.8, 95.0, 51.9, 46.7, 31.4, 21.66, 21.53; MALDI-MS obsd 589.3; ESI-MS obsd 588.3105 [M + H]^+^, calcd 588.3122 (M = C_40_H_37_N_5_); λ_abs_ (toluene) 415, 509, 590, 641 nm.

*13-[(E)-3-(4-Carboxyphenyl)prop-2-en-1-onyl]-10-mesityl-18,18-dimethylchlorin* (**H_2_C6**). A mixture of **H_2_C5** (30.0 mg, 0.060 mmol), terephthalaldehydic acid (90.0 mg, 0.600 mmol), and NaOH (48.0 mg, 1.20 mmol) in absolute ethanol (30 mL) was reacted under reflux in the open air via microwave irradiation at 60 W. The protocol [26] was as follows: (1) heat from room temperature to 100 °C (irradiate for 2 min); (2) hold at 100 °C (irradiate for 38 min); (3) allow to cool to room temperature. The reaction mixture was transferred to a round-bottom flask and concentrated. The resulting crude product was dissolved in CH_2_Cl_2_ and washed with a saturated aqueous solution of NH_4_Cl. The organic layer was separated, dried (Na_2_SO_4_), concentrated, and chromatographed [silica, CH_2_Cl_2_/CH_3_OH (49:1)] to afforded a grey solid (22 mg, 58%): ^1^H-NMR (THF–*d*_8_, 400 Hz) δ 10.27 (s, 1H), 9.64 (s, 1H), 9.27 (s, 1H), 9.20 (d, *J* = 4.4 Hz, 1H), 8.95 (d, *J* = 4.4 Hz, 1H), 8.91 (s, 1H), 8.75 (d, *J* = 4.4 Hz, 1H), 8.33 (d, *J* = 15.6 Hz, 1H), 8.24 (d, *J* = 4.4 Hz, 1H), 8.12 (d, *J* = 15.6 Hz, 1H), 8.10 (d, *J* = 8.0 Hz, 2H), 7.46 (d, *J* = 8.0 Hz, 2H), 7.30 (s, 2H), 4.63 (s, 2H), 2.61 (s, 3H), 2.04 (s, 6H), 1.88 (s, 6H), −0.98 (s, 1H), −1.33 (s, 1H), the –CO_2_H proton was not detected; ESI-MS obsd 633.28529 [M + H]^+^, calcd 633.28602 (M = C_41_H_36_N_4_O_3_); λ_abs_ (toluene) 382, 425, 515, 548, 612, 666 nm; λ_em_ (λ_exc_ = 424 nm, toluene) 670 nm.

*13-(3-Ethoxy-3-oxopropanoyl)-10-mesityl-18,18-dimethylchlorin* (**H_2_C7**). Following an reported procedure [27], a mixture of **H_2_C4** (25.0 mg, 46.5 µmol), potassium monoethyl malonate (11.9 mg, 69.8 µmol), MgCl_2_ (6.6 mg, 69.8 µmol), imidazole (6.1 mg, 93 µmol), xantphos (13.4 mg, 23.2 µmol), Pd(OAc)_2_ (5.2 mg, 23 µmol), and Et_3_N (10 µL, 70 µmol) in fresh distilled THF (1.0 mL) was deaerated by three freeze-pump-thaw cycles, CO gas was plugged in, and then Co_2_CO_8_ (7.9 mg, 23 µmol) was added and the mixture was stirred at 65 °C. After 16 h, the reaction mixture was concentrated, and chromatographed [silica, hexanes/CH_2_Cl_2_ (1:2)] to afford a brown solid (16 mg, 62%): ^1^H-NMR (CDCl_3_, 300 MHz): δ 10.04 (s, 1H), 9.48 (s, 1H), 9.04 (d, *J* = 4.4 Hz, 1H), 8.85 (s, 1H), 8.80 (d, *J* = 4.4 Hz, 1H), 8.70 (d, *J* = 4.4 Hz, 1H), 8.68 (s, 1H), 8.35 (d, *J* = 4.4 Hz, 1H), 7.25 (s, 2H), 4.54 (s, 2H), 4.46 (s, 2H), 4.28 (q, *J* = 7.2 Hz, 2H), 2.61 (s, 3H), 1.99 (s, 6H), 1.85 (s, 6H), 1.28 (t, *J* = 7.2 Hz, 3H), −0.88 (br s, 1H), −1.24 (br s, 1H); ^13^C-NMR (CDCl_3_, 100 MHz): δ 191.2, 178.7, 168.2, 164.9, 154.5, 152.6, 143.3, 139.2, 138.2, 137.49, 137.35, 137.1, 133.0, 130.8, 129.9, 128.8, 128.1, 127.2, 125.8, 123.9, 106.3, 97.7, 94.8, 64.7, 51.8, 49.2, 47.0, 31.0, 21.7, 21.5, 14.4; ESI-MS obsd 573.28573 [M + H]^+^, calcd 573.28602 (M = C_36_H_36_N_4_O_3_); λ_abs_ (toluene) 417, 661 nm.

*Zn(II)-10-[2,4,6-Tris(propargyloxy)phenyl]-18,18-dimethylchlorin* (**ZnC8**). Following a reported procedure [20], a solution of **4** (412 mg, 0.997 mmol) in anhydrous THF (11.1 mL) was treated with NBS (151 mg, 0.85 mmol) at −78 °C. The mixture was stirred at −78 °C for 1 h and then allowed to warm to 0 °C, whereupon water was added. Then the mixture was extracted by ethyl acetate. The organic phase was washed with water, dried (Na_2_SO_4_) and concentrated. The resulting product (**4-Br**) was dissolved in CH_2_Cl_2_ (23 mL) and treated with a sample of **2** (161 mg, 0.85 mmol). The resulting mixture was treated slowly with a solution of *p*-TsOH·H_2_O (0.81 g, 4.3 mmol) in MeOH (5.7 mL) under argon. The mixture was stirred at room temperature under argon for 30 min, whereupon 2,2,6,6-tetramethylpiperidine (1.03 mL, 6.38 mmol) was added. The mixture was then concentrated and dissolved in CH_3_CN (86 mL). Then the mixture was treated in succession with 2,2,6,6-tetramethylpiperidine (2.85 mL, 17.0 mmol), Zn(OAc)_2_ (2.33 g, 12.7 mmol) and AgOTf (656 mg, 2.55 mmol). The mixture was heated to reflux with exposure to air for 3 h. Then the mixture was passed through a silica pad (eluting with CH_2_Cl_2_), concentrated and chromatographed [silica gel, hexanes/CH_2_Cl_2_ (1:1)] to afford a dark green solid (127 mg, 20% for 3 steps): ^1^H-NMR (CDCl_3_, 300 MHz) δ 9.60 (s, 1H), 9.06 (d, *J* = 4.4 Hz, 1H), 8.85 (d, *J* = 4.4 Hz, 1H), 8.74 (d, *J* = 4.3 Hz, 1H), 8.72 (d, *J* = 4.5 Hz, 1H), 8.68 (s, 1H), 8.62 (d, *J* = 4.5 Hz, 1H), 8.59 (s, 1H), 8.53 (d, *J* = 4.3 Hz, 1H), 6.86 (s, 2H), 4.96–4.95 (m, 2H), 4.52 (s, 2H), 4.31 (br, 4H), 2.70 (s, 1H), 2.33 (s, 2H), 2.02 (s, 6H); ^13^C-NMR (CDCl_3_, 75 MHz) δ 170.3, 159.03, 158.98, 158.6, 153.4, 153.2, 148.0, 146.7, 146.2, 145.5, 132.56, 132.51, 128.5, 128.1, 127.1, 126.8, 115.3, 113.9, 109.4, 96.7, 94.8, 94.2, 78.62, 78.54, 76.0, 75.4, 56.42, 56.37, 50.4, 45.3, 30.9, 29.7; MALDI-MS obsd 640.8; ESI-MS obsd 640.1433 [M]^+^, calcd 640.1447 (M = C_37_H_28_N_4_O_3_Zn); λ_abs_ (toluene) 406, 608 nm.

*10-[2,4,6-Tris(propargyloxy)phenyl]-18,18-dimethylchlorin* (**H_2_C8**). Following a standard procedure [29], a solution of **ZnC8** (78 mg, 0.12 mmol) in CH_2_Cl_2_ (3.3 mL) was treated with TFA (279 µL, 3.6 mmol). The mixture was stirred at room temperature for 2 h, whereupon triethylamine and water were slowly added to the mixture. The organic phase was washed with brine, dried (Na_2_SO_4_), concentrated and chromatographed [silica gel, hexanes/CH_2_Cl_2_ (1:2)] to afford a dark green solid (55 mg, 79%): ^1^H-NMR (CDCl_3_, 300 MHz) δ 9.84 (s, 1H), 9.22 (d, *J* = 4.9 Hz, 1H), 9.01 (s, 1H), 8.95–8.94 (m, 2H), 8.90 (s, 1H), 8.824–8.817 (m, 2H), 8.65 (d, *J* = 4.2 Hz, 1H), 6.91 (s, 2H), 5.01–5.00 (m, 2H), 4.64 (s, 2H), 4.45–4.22 (m, 4H), 2.74 (s, 1H), 2.36 (s, 2H), 2.06 (s, 6H), −1.99 (s, 1H), −2.28 (s, 1H); ^13^C-NMR (CDCl_3_, 100 MHz) δ 174.4, 162.6, 159.2, 158.8, 153.7, 150.7, 140.2, 139.5, 135.9, 133.8, 132.6, 131.3, 127.9, 127.6, 123.7, 122.5, 107.1, 96.5, 94.9, 94.2, 78.6, 76.1, 75.4, 56.4, 52.1, 46.3, 31.2, 29.7; MALDI-MS obsd 579.2; ESI-MS obsd 579.2372 [M + H]^+^, calcd 579.2391 (M = C_37_H_30_N_4_O_3_); λ_abs_ (toluene) 404, 499, 522, 588, 639 nm.

*15-Bromo-10-[2,4,6-tris(propargyloxy)phenyl]-18,18-dimethylchlorin* (**H_2_C9**). Following a reported procedure [30], a solution of **H_2_C8** (20 mg, 35 µmol) in anhydrous THF (17.5 mL) was treated with NBS (6.2 mg, 35 µmol). The mixture was stirred at room temperature under argon for 30 min, whereupon saturated aqueous NaHCO_3_ solution was slowly added. The mixture was extracted with CH_2_Cl_2_. The organic phase was washed with brine, dried (Na_2_SO_4_), concentrated and chromatographed [silica gel, hexanes/CH_2_Cl_2_ (1:2)] to afford a dark green solid (15 mg, 65%): ^1^H-NMR (CDCl_3_, 300 MHz) δ 9.72 (s, 1H), 9.22 (d, *J* = 4.6 Hz, 1H), 9.15 (d, *J* = 4.4 Hz, 1H), 8.91 (d, *J* = 4.5 Hz, 1H), 8.88 (d, *J* = 4.4 Hz, 1H), 8.84 (s, 1H), 8.82 (d, *J* = 5.0 Hz, 1H), 8.65 (d, *J* = 4.4, 1H), 6.91 (s, 2H), 5.01–5.00 (m, 2H), 4.71 (s, 2H), 4.36–4.33 (m, 4H), 2.74 (s, 1H), 2.37 (s, 2H), 2.06 (s, 6H), −2.05 (br, 1H), −2.12 (br, 1H); ^13^C-NMR (CDCl_3_, 125 MHz) δ 176.0, 162.2, 159.6, 158.9, 153.4, 152.8, 140.7, 138.3, 135.8, 135.6, 132.7, 128.4, 128.2, 124.4, 114.82, 114.73, 106.7, 96.4, 95.1, 94.8, 78.7, 76.4, 75.8, 56.65, 56.54, 55.4, 46.4, 31.8, 30.0, 29.6; MALDI-MS obsd 658.2; ESI-MS obsd 656.1400 [M]^+^, calcd 656.1418 (M = C_37_H_29_N_4_O_3_Br); λ_abs_ (toluene) 404, 505, 530, 593, 646 nm.

*Zn(II)-15-Bromo-10-[2,4,6-tris(propargyloxy)phenyl]-18,18-dimethylchlorin* (**ZnC9**). Following a standard procedure [31], a solution of **H_2_C9** (14 mg, 21 µmol) in CH_2_Cl_2_/MeOH (2.1 mL, 1:1) was treated with Zn(OAc)_2_ (20 mg, 0.11 mmol). The mixture was stirred at room temperature for 16 h. The mixture was concentrated and chromatographed [silica, hexanes/CH_2_Cl_2_ (1:2)] to afford a dark green solid (15 mg, 99%): ^1^H NMR (CDCl_3_, 300 MHz) δ 9.51 (s, 1H), 9.06 (d, *J* = 4.6 Hz, 1H), 9.00 (d, *J* = 4.4 Hz, 1H), 8.79 (d, *J* = 4.3 Hz, 1H), 8.69 (d, *J* = 4.3 Hz, 1H), 8.67 (d, *J* = 4.6 Hz, 1H), 8.52–8.51 (m, 2H), 6.85 (s, 2H), 4.96 (d, *J* = 2.5 Hz, 2H), 4.59 (s, 2H), 4.33 (d, *J* = 2.4 Hz, 4H), 2.71 (s, 1H), 2.34 (s, 2H), 2.01 (s, 6H); ^13^C NMR (CDCl_3_, 125 MHz) δ 172.0, 159.1, 158.5, 157.7, 154.0, 152.1, 147.62, 147.49, 146.62, 146.54, 133.2, 132.7, 129.2, 128.7, 127.6, 115.0, 109.0, 96.6, 94.73, 94.58, 78.5, 76.1, 75.5, 56.4, 54.0, 44.7, 31.5, 29.7; MALDI-MS obsd 721.8; ESI-MS obsd 718.0556 [M]^+^, calcd 718.0553 (M = C_37_H_27_N_4_O_3_BrZn); λ_abs_ (Toluene) 412, 613 nm.

*15-Bromo-10-[2,4,6-tris(2,5,8,11,14,17-hexaoxanonadecyl-1H-1,2,3-triazol-4-ylmethoxy)phenyl]-18,18-dimethylchlorin* (**H_2_C9-PEG_6_**). Following a reported procedure [32], a mixture of **ZnC9** (15 mg, 21 µmol), N_3_(C_2_H_4_O)_6_CH_3_ (98 mg, 0.31 mmol) and sodium ascorbate (8.2 mg, 41 µmol) in CH_2_Cl_2_ (7.6 mL) was treated with CuI (4.1 mg, 21 µmol) and *N,N*-diisopropylethylamine (183 µL, 1.1 mmol) under argon. The resulting mixture was stirred at room temperature under argon for 3 h. MALDI-MS analysis showed complete consumption of the starting material. The mixture was diluted with CH_2_Cl_2_ and washed with water. The organic phase was dried (Na_2_SO_4_), concentrated and dissolved in CH_2_Cl_2_ (25 mL). The resulting solution was treated with TFA (169 µL, 2.2 mmol) and stirred at room temperature for 5 min. Absorption analysis showed the demetalation was complete. Saturated aqueous NaHCO_3_ solution was slowly added to the mixture to quench the reaction. The organic phase was washed with brine, dried (Na_2_SO_4_), concentrated and chromatographed using a three-column strategy [33] [(1) silica, CH_2_Cl_2_/MeOH (95:5); (2) SEC, toluene; (3) silica, CH_2_Cl_2_/MeOH (95:5)] to afford a dark brown solid (30 mg, 88% for two steps): ^1^H-NMR (CDCl_3_, 400 MHz) δ 9.72 (s, 1H), 9.20 (d, *J* = 4.7 Hz, 1H), 9.14 (d, *J* = 4.9 Hz, 1H), 8.94 (d, *J* = 4.7 Hz, 1H), 8.85 (s, 1H), 8.81 (d, *J* = 4.3 Hz, 1H), 8.72 (d, *J* = 4.8 Hz, 1H), 8.54 (d, *J* = 4.3 Hz, 1H), 8.09 (s, 1H), 6.88 (s, 2H), 6.02 (s, 2H), 5.46 (s, 2H), 5.03 (d, *J* = 1.4, 3H), 4.70–4.66 (m, 3H), 3.99 (t, *J* = 5.1 Hz, 2H), 3.80 (t, *J* = 5.1 Hz, 3H), 3.73–3.60 (m, 21H), 3.57–3.51 (m, 3H), 3.49–3.33 (m, 22H), 3.30–3.23 (m, 10H), 3.14 (dd, *J* = 6.0, 4.1 Hz, 4H), 3.04 (p, *J* = 5.3 Hz, 4H), 2.93 (dd, *J* = 5.7, 3.9 Hz, 4H), 2.75–2.69 (m, 4H), 2.57 (p, *J* = 4.5 Hz, 4H), 2.06 (s, 6H), −2.08 (br, 2H); ^13^C-NMR (CDCl_3_, 125 MHz) δ 176.0, 161.9, 160.7, 159.4, 153.5, 152.3, 143.7, 143.5, 140.5, 137.8, 135.2, 132.9, 132.4, 130.9, 128.8, 128.28, 128.14, 124.50, 124.35, 124.2, 122.9, 116.0, 113.5, 94.2, 71.9, 71.81, 71.76, 70.62, 70.57, 70.55, 70.52, 70.47, 70.33, 70.30, 70.21, 70.13, 70.01, 69.82, 69.71, 69.57, 69.53, 68.51, 63.32, 59.03, 58.94, 55.1, 50.4, 49.5, 46.2, 31.6; MALDI-MS obsd 1622.6; ESI-MS obsd 1642.6952 [M + Na]^+^, calcd 1642.7015 (M = C_76_H_110_N_13_O_21_Br); λ_abs_ (MeOH) 399, 503, 529, 591, 642 nm.

*15-[4-(3-Carboxypropanamido)phenyl]-10-[2,4,6-tris(2,5,8,11,14,17-hexaoxanonadecyl-1H-1,2,3-triazol-4-ylmethoxy)phenyl]-18,18-dimethylchlorin* (**H_2_C10-PEG_6_**). Following a reported procedure [35,36], a mixture of **H_2_C9-PEG_6_** (6.5 mg, 4.0 µmol), **6** (1.9 mg, 6.0 µmol) and Cs_2_CO_3_ (6.5 mg, 20 µmol) in a Schlenk flask was degassed under high vacuum for 20 min. A sample of Pd(PPh_3_)_4_ (1.2 mg, 1.0 µmol) was added, whereupon the flask was further degassed for 20 min. Degassed toluene/DMF (1.0 mL, 1:3, *v*/*v*) was added to the flask. The resulting solution was degassed by three freeze-pump-thaw cycles. The resulting mixture was stirred at 90 °C for 3 h, whereupon water was added. The organic phase was washed with brine, dried (Na_2_SO_4_), concentrated and chromatographed [silica, CH_2_Cl_2_/MeOH/triethylamine (95:5:1)] to afford a dark green solid (5.1 mg, 74%): ^1^H-NMR (DMSO-*d*_6_, 400 MHz) δ 11.99 (br, 1H, –CO_2_H proton), 9.92 (s, 1H), 9.42 (d, *J* = 4.7 Hz, 1H), 9.16–9.15 (m, 2H), 8.92 (d, *J* = 4.3 Hz, 1H), 8.52 (d, *J* = 4.9 Hz, 1H), 8.41 (s, 1H), 8.39 (d, *J* = 4.3 Hz, 1H), 8.21 (d, *J* = 5.0 Hz, 1H), 7.91 (d, *J* = 8.5 Hz, 2H), 7.78 (d, *J* = 8.4 Hz, 2H), 7.54 (s, 1H), 7.04 (s, 2H), 6.76 (s, 2H), 5.43 (s, 2H), 4.97 (s, 4H), 4.63 (t, *J* = 5.1 Hz, 2 H), 4.15 (s, 2H), 3.96–3.88 (m, 7 H), 3.60–3.57 (m, 3H), 3.54–3.44 (m, 23H), 3.28–3.25 (m, 12H), 3.22–3.19 (m, 10H), 3.12–3.06 (m, 15H), 3.02–2.99 (m, 3H), 2.86–2.83 (m, 4H), 2.69–2.66 (m, 3H), 2.59–2.55 (m, 3H), 1.92 (s, 6H), −2.32 (br, 2H); ^13^C-NMR (CD_3_OD, 100 MHz) δ 176.3, 174.2, 163.9, 162.3, 161.0, 154.9, 152.9, 144.8, 144.5, 141.6, 141.1, 139.9, 139.4, 136.59, 136.47, 135.9, 133.9, 133.6, 128.7, 126.6, 125.1, 124.9, 124.6, 123.2, 120.7, 116.1, 115.9, 113.1, 95.9, 95.6, 72.7, 71.8, 71.25, 71.14, 71.0, 70.4, 70.2, 70.1, 69.9, 69.7, 69.51, 69.43, 69.1, 63.8, 62.9, 59.2, 58.6, 53.4, 51.4, 50.4, 47.1, 37.0, 34.2, 33.1, 31.6, 30.8, 30.6, 25.19, 25.03; ESI-MS obsd 1731.8541 [M − H]^–^, calcd 1731.8527 (M = C_86_H_120_N_14_O_24_); λ_abs_ (methanol) 409, 504, 530, 591, 641 nm.

*15-[4-(3-Succinimidylpropanamido)phenyl]-10-[2,4,6-tris(2,5,8,11,14,17-hexaoxanonadecyl-1H-1,2,3-triazol-4-ylmethoxy)phenyl]-18,18-dimethylchlorin* (**H_2_C10-PEG_6_-NHS**). A mixture of **H_2_C10-PEG_6_** (5.1 mg, 2.9 µmol) and DCC (6.0 mg, 29 µmol) in CH_2_Cl_2_ (290 µL) was treated with *N*-hydroxysuccinimide (3.3 mg, 29 µmol). The mixture was stirred at room temperature for 3 h. MALDI-MS analysis showed the completion of the reaction. Then the mixture was washed with water, dried (Na_2_SO_4_), concentrated and chromatographed [silica, CH_2_Cl_2_/MeOH (95:5)] to afford a dark green solid (5.0 mg, 94%): MALDI-MS obsd 1832.90, calcd 1829.88 (M = C_90_H_123_N_15_O_26_).

*15-[4-(2-(tert-Butyloxycarbonyl)ethyl)-phenyl]-10-[2,4,6-tris(2,5,8,11,14,17-hexaoxanonadecyl-1H-1,2,3-triazol-4-ylmethoxy)phenyl]-18,18-dimethylchlorin* (**H_2_C11-PEG_6_**). Following a reported procedure [35,36], a mixture of **H_2_C9-PEG_6_** (25 mg, 15 µmol), **7** (7.3 mg, 22 µmol) and Cs_2_CO_3_ (24 mg, 75 µmol) in a Schlenk flask was degassed under high vacuum for 20 min. A sample of Pd(PPh_3_)_4_ (4.4 mg, 3.8 µmol) was added, whereupon the flask was further degassed for 20 min. Degassed toluene/DMF (1.0 mL, 1:3, *v*/*v*) was added to the flask. The resulting solution was degassed by three freeze-pump-thaw cycles. The resulting mixture was stirred at 90 °C for 2 h, whereupon water was added. The organic phase was washed with brine, dried (Na_2_SO_4_), concentrated and chromatographed [silica, CH_2_Cl_2_/MeOH (95:5)] to afford a green solid (10 mg, 38%): ^1^H-NMR (CDCl_3_, 300 MHz): δ 9.77 (s, 1H), 9.22 (d, *J* = 4.6 Hz, 1H), 8.96 (d, *J* = 4.6 Hz, 1H), 8.92 (s, 1H), 8.86 (d, *J* = 4.3 Hz, 1H), 8.66 (d, *J* = 4.9 Hz, 1H), 8.54 (d, *J* = 4.3 Hz, 1H), 8.24 (d, *J* = 4.9 Hz, 1H), 8.08 (s, 1H), 7.79 (d, *J* = 7.8 Hz, 2H), 7.54 (d, *J* = 7.9 Hz, 2H), 6.87 (s, 2H), 5.89 (s, 2H), 5.46 (s, 2H), 5.02 (d, *J* = 7.2 Hz, 4H), 4.67 (t, *J* = 5.1 Hz, 2H), 4.18 (s, 2H), 3.98 (t, *J* = 5.1 Hz, 3H), 3.72–3.61 (m, 21H), 3.56–3.50 (m, 3H), 3.50–3.34 (m, 21H), 3.31–3.17 (m, 10H), 3.13 (dd, *J* = 6.0, 3.9 Hz, 4H), 2.96–2.93 (m, 3H), 2.92–2.88 (m, 4H), 2.82 (t, *J* = 7.6 Hz, 2 H), 2.68–2.61 (m, 4H), 2.47–2.40 (m, 3H), 2.35 (dd, *J* = 6.9, 3.7 Hz, 5H), 1.97 (s, 6H), 1.54 (s, 9H), −2.21 (br, 2H); ESI-MS: obsd, 1746.9202 [M + H]^+^; calcd 1746.9241 (M = C_89_H_127_N_13_O_23_).

*15-[4-(2-Carboxyethyl)-phenyl]-10-[2,4,6-tris(2,5,8,11,14,17-hexaoxanonadecyl-1H-1,2,3-triazol-4-yl-methoxy)phenyl]-18,18-dimethylchlorin* (**H_2_C12-PEG_6_**). A solution of **H_2_C11-PEG_6_** (10 mg, 5.7 µmol) in CH_2_Cl_2_ (3.3 mL) was treated with TFA (2.2 mL). The mixture was stirred at room temperature for 15 h, whereupon saturated sodium bicarbonate solution was added slowly to quench the reaction. The mixture was then washed with water, dried (Na_2_SO_4_), concentrated to afford a black solid (9.0 mg, 93%): ^1^H-NMR (CDCl_3_, 400 MHz, the CO_2_H proton peak was not observed): δ 9.78 (s, 1H), 9.22 (d, *J* = 4.6 Hz, 1H), 8.92 (s, 1H), 8.87 (d, *J* = 4.3 Hz, 1H), 8.60 (d, *J* = 4.8 Hz, 1H), 8.56 (d, *J* = 4.3 Hz, 1H), 8.21 (d, *J* = 4.8 Hz, 1H), 8.10 (s, 1H), 7.82–7.77 (m, 2H), 7.56 (d, *J* = 7.7 Hz, 2H), 6.87 (s, 2H), 5.97 (s, 2H), 5.47 (s, 2H), 5.02 (d, *J* = 6.4 Hz, 4H), 4.67 (t, *J* = 5.1 Hz, 2H), 4.21 (s, 2H), 3.98 (d, *J* = 5.1 Hz, 3H), 3.77–3.61 (m, 21H), 3.57–3.51 (m, 3H), 3.50–3.35 (m, 21H), 3.33–3.23 (m, 10H), 3.21 (dd, *J* = 5.7, 3.8 Hz, 4H), 3.06–3.02 (m, 3H), 3.02–2.98 (m, 4H), 2.93 (t, *J* = 7.6 Hz, 2 H), 2.79–2.75 (m, 4H), 2.61–2.54 (m, 3H), 2.53–2.47 (m, 6H), 1.98 (s, 6H), -2.09 (br, 2H); ESI-MS: obsd, 1712.8384 [M + Na]^+^; calcd, 1712.8334 (M = C_85_H_119_N_13_O_23_).

*15-[4-(2-(Succinimidyloxy)carboxyethyl)-phenyl]-10-[2,4,6-tris(2,5,8,11,14,17-hexaoxanonadecyl-1H-1,2,3-triazol-4-ylmethoxy)phenyl]-18,18-dimethylchlorin* (**H_2_C12-PEG_6_-NHS**). A solution of **H_2_C12-PEG_6_** (4.0 mg, 2.4 µmol) and **8** (7.2 mg, 24 µmol) in CH_2_Cl_2_ (0.5 mL) was treated with triethylamine (3.3 µL). The mixture was stirred at room temperature for 16 h. MALDI-MS showed all starting material was converted to the desired product. The mixture was treated with water. The organic phase was washed with water, dried (Na_2_SO_4_), and concentrated to afford a dark green solid.

*Zinc(II)-15-[4-(2-Carboxyethyl)-phenyl]-10-[2,4,6-tris(2,5,8,11,14,17-hexaoxanonadecyl-1H-1,2,3-triazol-4-ylmethoxy)phenyl]-18,18-dimethylchlorin* (**ZnC12-PEG_6_**). Following a standard procedure [31], a solution of **H_2_C12-PEG_6_** (10 mg, 5.3 µmol) in CH_2_Cl_2_/MeOH (0.5 mL, 1:1 *v*/*v*) was treated with zinc acetate (5.0 mg 27 µmol). The mixture was stirred at room temperature for 16 h, whereupon water was added to quench the reaction. The mixture was then washed with water, dried (Na_2_SO_4_), concentrated to afford a dark green solid (9.0 mg, 96%): ^1^H-NMR (THF-*d*_8_, 300 MHz, the CO_2_H proton peak was not observed): δ 9.52 (s, 1H), 9.01 (d, *J* = 4.3 Hz, 1H), 8.74 (d, *J* = 4.2 Hz, 1H), 8.69 (d, *J* = 4.2 Hz, 1H), 8.57 (s, 1H), 8.48 (d, *J* = 4.5 Hz, 1H), 8.40 (d, *J* = 4.2 Hz, 1H), 8.21 (s, 1H), 8.01 (d, *J* = 4.3 Hz, 1H), 7.75 (d, *J* = 7.6 Hz, 2H), 7.55 (d, *J* = 7.7 Hz, 2H), 6.94 (s, 2H), 6.46 (s, 2H), 5.40 (s, 2H), 4.95 (s, 4H), 4.59 (s, 2H), 4.14 (s, 2H), 3.55–3.52 (m, 21H), 3.42–3.30 (m, 28H), 3.24 (br, 10H), 3.20–3.16 (m, 10H), 3.07–3.04 (m, 4H), 2.95–2.90 (m, 2H), 2.80 (br, 8H), 1.94 (s, 6H); ESI-MS: obsd, 1752.7709 [M + H]^+^; calcd, 1752.7750 (M = C_85_H_117_N_13_O_23_Zn).

*13-Bromo-10-[2,4,6-tris(propargyloxy)phenyl]-18,18-dimethylchlorin* (**H_2_C13**). Following a reported procedure [20], a solution of **4** (457 mg, 1.11 mmol) in anhydrous THF (11.1 mL) was treated with NBS (395 mg, 2.22 mmol) at −78 °C. The mixture was stirred at −78 °C for 1 h and then allowed to warm to 0 °C, whereupon water was added. Then the mixture was extracted by ethyl acetate. The organic phase was washed with water, dried (Na_2_SO_4_) and concentrated. The resulting product (**4-Br_2_**) was dissolved in CH_2_Cl_2_ (30 mL) and treated with a sample of **2** (209 mg, 1.11 mmol). The resulting mixture was treated slowly with a solution of *p*-TsOH·H_2_O (1.06 g, 5.55 mmol) in MeOH (7.5 mL) under argon. The mixture was stirred at room temperature under argon for 30 min, whereupon 2,2,6,6-tetramethylpiperidine (1.3 mL, 8.3 mmol) was added. The mixture was then concentrated and dissolved in CH_3_CN (112 mL). Then the mixture was treated in succession with 2,2,6,6-tetramethylpiperidine (3.7 mL, 24 mmol), Zn(OAc)_2_ (3.0 g, 16 mmol) and AgOTf (857 mg, 3.34 mmol). The mixture was heated to reflux with exposure to air for 16 h. Then the mixture was passed through a silica pad (eluting with CH_2_Cl_2_), concentrated. The resulting product was dissolved in CH_2_Cl_2_ (30 mL) and treated with TFA (2.6 mL, 33 mmol). The mixture was stirred at room temperature for 30 min, whereupon saturated sodium bicarbonate solution was added slowly to quench the reaction. The mixture was then washed with water, dried (Na_2_SO_4_), concentrated and chromatographed [silica, hexanes/CH_2_Cl_2_ (1:1)] to afford a dark green solid (84 mg, 12% for four steps): ^1^H-NMR (CDCl_3_, 400 MHz): δ 9.79 (s, 1H), 9.20 (d, *J* = 4.7 Hz, 1H), 9.11 (s, 1H), 8.92 (d, *J* = 4.6 Hz, 1H), 8.91–8.89 (m, 1H), 8.89 (s, 1H), 8.84 (s, 1H), 8.62 (d, *J* = 4.3 Hz, 1H), 6.88 (s, 2H), 5.00 (d, *J* = 2.4 Hz, 2H), 4.66 (s, 2H), 4.42–4.25 (m, 4H), 2.74 (s, 1H), 2.38 (t, *J* = 2.3 Hz, 2H), 2.05 (s, 6H), −1.94 (br, 1H), −2.21 (br, 1H); ^13^C-NMR (CDCl_3_, 100 MHz): δ 175.0, 162.9, 159.4, 158.7, 151.4, 140.7, 136.1, 134.4, 132.8, 131.8, 128.15, 128.07, 123.2, 107.2, 94.8, 94.7, 78.5, 76.1, 75.5, 56.4, 56.1, 52.1, 46.4, 31.1 ; ESI-MS obsd, 657.1480 [M + H]^+^; calcd, 657.1496 (M = C_37_H_29_BrN_4_O_3_); λ_abs_ (toluene) 409, 501, 595, 647 nm.

*Zinc(II)-13-Bromo-10-[2,4,6-tris(propargyloxy)phenyl]-18,18-dimethylchlorin* (**ZnC13**). Following a standard procedure [31], a solution of **H_2_C13** (39 mg, 59 µmol) in CH_2_Cl_2_/MeOH (5.6 mL, 1:1 *v*/*v*) was treated with zinc acetate (33 mg 0.18 mmol). The mixture was stirred at room temperature for 20 h, whereupon water was added to quench the reaction. The mixture was then washed with water, dried (Na_2_SO_4_), concentrated to afford a violet solid (42 mg, 99%): ^1^H-NMR (CDCl_3_, 300 MHz): δ 9.55 (s, 1H), 9.03 (d, *J* = 4.3 Hz, 1H), 8.84 (s, 1H), 8.81 (d, *J* = 4.3 Hz, 1H), 8.73 (s, 1H), 8.71 (s, 1H), 8.58 (s, 1H), 8.51 (d, *J* = 4.1 Hz, 1H), 6.84 (s, 2H), 4.96 (d, *J* = 2.4 Hz, 2H), 4.54 (s, 2H), 4.34 (s, 4H), 2.72 (s, 1H), 2.36 (s, 2H), 2.01 (s, 6H); ^13^C-NMR (CDCl_3_, 75 MHz): δ 159.2, 158.6, 153.9, 146.1, 132.9, 132.4, 128.8, 128.7, 127.3, 115.7, 109.3, 94.74, 94.66, 94.1, 78.5, 76.0, 75.5, 56.4, 53.4, 50.5, 45.3, 31.6, 30.8, 25.3, 22.6, MALDI-MS obsd 721.2; ESI-MS: obsd, 718.0540 [M + H]^+^; calcd, 718.0553 (M = C_37_H_27_BrN_4_O_3_Zn).

*13-Bromo-10-[2,4,6-tris(2,5,8,11,14,17-hexaoxanonadecyl-1H-1,2,3-triazol-4-ylmethoxy)phenyl]-18,18-di-methylchlorin* (**H_2_C14-PEG_6_**). Following a reported procedure [32], a mixture of **ZnC13** (20 mg, 28 µmol), N_3_(C_2_H_4_O)_6_CH_3_ (131 mg, 0.41 mmol) and sodium ascorbate (11 mg, 56 µmol) in CH_2_Cl_2_ (10 mL) was treated with CuI (5.3 mg, 28 µmol) and *N,N*-diisopropylethylamine (244 µL, 1.4 mmol) under argon. The resulting mixture was stirred at room temperature under argon for 2 h. MALDI-MS analysis showed complete consumption of the starting material. The mixture was diluted with CH_2_Cl_2_ and washed with water. The organic phase was dried (Na_2_SO_4_), concentrated and dissolved in CH_2_Cl_2_ (90 mL). The resulting solution was treated with TFA (225 µL, 2.93 mmol) and stirred at room temperature for 5 min. Absorption analysis showed the demetalation was complete. Saturated aqueous NaHCO_3_ solution was slowly added to the mixture to quench the reaction. The organic phase was washed with brine, dried (Na_2_SO_4_), concentrated and chromatographed [silica, CH_2_Cl_2_/MeOH (95:5)] to afford a dark yellow solid (42 mg, 93% for two steps): ^1^H-NMR (CDCl_3_, 400 MHz): δ 9.78 (s, 1H), 9.22 (d, *J* = 4.2 Hz, 1H), 9.10 (s, 1H), 8.94 (d, *J* = 4.4 Hz, 1H), 8.90 (s, 1H), 8.85 (d, *J* = 4.3 Hz, 1H), 8.75 (s, 1H), 8.53 (d, *J* = 4.2 Hz, 1H), 8.10 (s, 1H), 6.88 (s, 2H), 6.11 (s, 2H), 5.46 (s, 2H), 5.07–4.98 (s, 4H), 4.66 (s, 2H), 3.99 (t, *J* = 5.3 Hz, 2H), 3.82 (t, *J* = 5.3 Hz, 3H), 3.72–3.60 (m, 21H), 3.54 (t, *J* = 4.5 Hz, 3H), 3.50–3.31 (m, 21H), 3.30–3.25 (m, 10H), 3.18–3.12 (m, 4H), 3.10–3.01 (m, 3H), 2.95 (dd, *J* = 5.7, 3.8 Hz, 4H), 2.75 (t, *J* = 4.8 Hz, 4 H), 2.62–2.53 (m, 3H), 2.52–2.48 (m, 3H), 2.06 (s, 6H), −1.93 (s, 1H), −2.21 (s, 1H); ^13^C-NMR (CDCl_3_, 100 MHz): δ 175.2, 162.9, 160.8, 159.3, 143.7, 143.5, 140.7, 136.0, 134.2, 132.7, 132.1, 128.2, 124.5, 122.9, 71.91, 71.88, 71.80, 71.74, 70.65, 70.64, 70.63, 70.59, 70.55, 70.48, 70.45, 70.38, 70.34, 70.27, 70.18, 70.14, 70.07, 69.88, 69.78, 69.63, 69.61, 69.52, 69.48, 68.5, 63.2, 58.99, 58.91, 58.85, 58.77, 50.42, 50.36, 49.5, 46.4, 31.1; ESI-MS obsd, 1642.6987 [M + Na]^+^; calcd, 1642.7015 (M = C_76_H_110_N_13_O_21_Br); λ_abs_ (CH_2_Cl_2_) 409, 501, 593, 645 nm.

*13-[6-Carboxylhex-1-ynyl]-10-[2,4,6-tris(2,5,8,11,14,17-hexaoxanonadecyl-1H-1,2,3-triazol-4-ylmethoxy)-phenyl]-18,18-dimethylchlorin* (**H_2_C15-PEG_6_**). Following a reported procedure [24], a mixture of DMF/triethylamine (3.1 mL, 5:1, *v*/*v*) was deaerated with a continuous stream of argon for 1 h. Samples of **H_2_C14-PEG_6_** (25 mg, 15 µmol), **9** (10 mg, 76 µmol), P(*o*-tol)_3_ (5.8 mg, 2.3 µmol) and Pd_2_(dba)_3_ (2.1 mg, 2.3 µmol) were placed into a Schlenk flask, the contents of which were then degassed under high vacuum for 20 min. A solution of the deaerated DMF/triethylamine solution (5.3 mL) was added to the flask. The mixture was degassed by three freeze-pump-thaw cycles. The resulting mixture was stirred at 80 °C for 18 h. The mixture was allowed to cool to room temperature. The cooled mixture was diluted with CH_2_Cl_2_, washed with water, dried (Na_2_SO_4_), concentrated. The resulting solid was dissolved in CH_2_Cl_2_ (1.0 mL) and treated with TFA (1.0 mL). The reaction mixture was stirred at room temperature for 16 h, whereupon saturated sodium bicarbonate solution was added slowly to quench the reaction. The mixture was then washed with water, dried (Na_2_SO_4_), concentrated and chromatographed [reverse phase silica TLC plate (C_18_), methanol] to afford a green solid as the first band (19 mg, 76% for two steps): ^1^H-NMR (DMSO-*d*_6_, 300 MHz, the CO_2_H proton peak was not observed): δ 9.93 (s, 1H), 9.43 (s, 1H), 9.13 (br, 2H), 8.91 (d, *J* = 4.4 Hz, 1H), 8.50 (s, 1H), 8.44 (s, 1H), 8.38 (d, *J* = 4.3 Hz, 1H), 7.65 (s, 1H), 7.07 (s, 2H), 6.87 (s, 2H), 5.45 (d, *J* = 7.2 Hz, 2H), 5.00 (d, *J* = 5.5 Hz, 4H), 4.68–4.56 (m, 2H), 4.04–3.94 (m, 2H), 3.92–3.85 (m,2H), 3.68 (s, 3H), 3.60–3.45 (m, 21H), 3.23–3.17 (m, 31H), 3.12–3.10 (m, 4H), 3.05–3.02 (m, 4H), 2.90–2.84 (m, 6H), 2.74–2.72 (m, 6H), 2.70–2.67 (m, 3H), 2.60–2.58 (m, 3H), 2.06 (s, 6H), 2.00–1.97 (m, 4H), −2.07 (s, 1H), −2.32 (s, 1H); ESI-MS obsd, 1666.8591 [M + H]^+^; calcd, 1666.8615 (M = C_83_H_119_N_13_O_23_); λ_abs_ (CH_2_Cl_2_) 413, 503, 599, 649 nm.

*Zinc(II)-13-(6Carboxylhex-1-ynyl)-10-[2,4,6-tris(2,5,8,11,14,17-hexaoxanonadecyl-1H-1,2,3-triazol-4-yl-methoxy)phenyl]-18,18-dimethylchlorin* (**ZnC15-PEG_6_**). Following a standard procedure [31], a solution of **H_2_C15-PEG_6_** (3.0 mg, 1.8 µmol) in CH_2_Cl_2_/MeOH (1.0 mL, 1:1 *v*/*v*) was treated with zinc acetate (1.7 mg 9.3 µmol). The mixture was stirred at room temperature for 16 h, whereupon water was added to quench the reaction. The mixture was then washed with water, dried (Na_2_SO_4_), concentrated to afford a blue solid (3.0 mg, 96%): ^1^H-NMR (pyridine-*d*_5_, 300 MHz, the CO_2_H proton peak was not observed): δ 9.71 (s, 1H), 9.24 (br, 2H), 9.14 (s, 1H), 8.99 (br, 2H), 8.84–8.82 (m, 2H), 8.59 (s, 1H), 7.43 (s, 2H), 6.90 (d, *J* = 7.2 Hz, 2H), 5.79 (s, 2H), 5.34 (d, *J* = 5.8 Hz, 4H), 4.76 (s, 2H), 4.40 (d, *J* = 7.6 Hz, 2H), 4.02–3.91 (m, 3H), 3.78–3.36 (m, 50 H), 3.32–3.20 (m, 14 H), 3.09–3.05 (m, 8H), 2.64 (br, 4H), 2.24 (br, 4H), 1.97–1.95 (m, 10H); ESI-MS: obsd, 864.8911 [M + 2H]^2+^; calcd, 864.8895 (M = C_83_H_117_N_13_O_23_Zn).

### 3.3. Measuring Absorption and Emission of Chlorins in Micellar Solution

The cmc of CTAB at 25 °C is ~1 mM [52]. Each chlorin was dissolved in 1.5 µL of CH_2_Cl_2_ and diluted with 3.0 mL aqueous CTAB (10 mM) solution in a glass cuvette. The cuvette was vigorously shaken before the absorption and emission experiments.

### 3.4. Experimental Method for Chlorin–Avidin Conjugation

Stock solutions of **H_2_C12-PEG_6_-NHS** and avidin were prepared separately in 0.1 M sodium phosphate buffer (pH 7.6). A solution of **H_2_C12-PEG_6_-NHS** (corresponding to 0.42 mg, 0.84 mg, or 1.26 mg for 30, 60 or 90 equiv.) in 0.1 M sodium phosphate buffer (50 µL, pH 7.6) was added to a solution of avidin (corresponding to 0.5 mg, 7.9 nmol) in 0.1 M sodium phosphate buffer (50 µL, pH 7.6) in a conical vial. The vial was placed on a rocker to gently mix the contents for 16 h at room temperature. To purify the conjugate, the reaction mixture was diluted with deionized water to a final volume of 1.0 mL; the entire resulting 1-mL volume was added to a gel permeation chromatography column (GE-25 midiTrap, molecular weight cut-off at 5000 Da, Thermo Fisher Scientific Inc., Waltham, MA, USA) that was pre-washed with water. The reaction mixture was allowed to enter the column bed completely and the eluent (colorless) was discarded. Then 1.5 mL of deionized water was added to the column whereupon the eluent (faint green) was collected in a 20-mL vial. The eluent was placed in a centrifugal filter (Amicon Ultra-4, molecular weight cut-off at 30,000 Da, MilliporeSigma, Billerica, MA, USA) and centrifuged at 4000 rpm for 5 min. The residual solution inside the membrane, which contains the chlorin–avidin conjugate, was collected by pipette. The excess chlorin (detectable by red fluorescence upon UV illumination) remained bound near the top of the gel permeation chromatography column. The conjugate was then analyzed by absorption and fluorescence spectroscopy. An estimate of loading was carried out by (1) measuring the relative absorption at 280 nm for the chlorin–avidin conjugate versus that of **H_2_C12-PEG_6_-NHS** at the Soret band maximum, and (2) using the molar absorption coefficient at 280 nm of avidin (ε_280 nm_ = 96,000 M^−1^cm^−1^) [70] and of the synthetic chlorin **H_2_C12-PEG_6_-NHS** (ε_280nm_ ~ 19,500 M^−1^cm^−1^); the latter is assumed on the basis of the value for the ε(Q_y_) band of structurally similar 10,15-diarylchlorins [8,9] and the known spectrum of **H_2_C12-PEG_6_-NHS**. The baseline for each absorption spectrum was established at the long wavelength region (700–900 nm) without any correction for any other putative absorbers. The ratio of the intensity of absorption of the Soret band versus that at 280 nm increases as the number of equivalents of **H_2_C12-PEG_6_-NHS** increases (see Figure 9), as expected for increased loading.

### 3.5. Photophysical Properties

Photophysical studies were carried out on dilute (μM) argon-purged samples at room temperature, typically on samples containing ambient O_2_. Static emission spectra were acquired using a Nanolog (Spex-Horiba, Edison NJ, USA) or QuantaMaster (Photon Technology International-Horiba, Edison, NJ, USA) spectrofluorimeter with 2–4 nm excitation and detection bandwidths and corrected for instrument spectral response. Fluorescence quantum yields were obtained relative to *meso*-tetraphenylporphyrin (Φ_f_ = 0.070 in nondegassed toluene) [74] or 18,18-dimethyl-5-*p*-tolyl-10-mesitylchlorin in toluene (Φ_f_ = 0.22 in nondegassed toluene) [35]. Singlet excited-state lifetimes were determined by using transient absorption spectroscopy employing ~100 fs excitation flashes from an ultrafast laser system (Spectra Physics, Santa Clara, CA, USA) and acquisition of difference spectra (360–900 nm) using a white-light pulsed laser (~1 ns rise time) in 100-ps time bins with variable pump-probe spacing up to 0.5 ms (EOS, Ultrafast Systems, Sarasota, FL, USA). The same transient absorption studies provided the yield of S_1_→ T_1_ intersystem crossing by comparing the extent of bleaching of the ground-state absorption bands due to T_1_ at the asymptote of the S_1_ decay versus the extent due to S_1_ immediately after the excitation flash. Measurements in the Q_y_ region account for the contribution of both S_0_ bleaching and S_1_ stimulated emission to the S_1_ feature.

## 4. Conclusions

The present work illustrates the design and synthesis of stable chlorins spanning a range of polarity and bearing diverse substituents. Eight target chlorins (19 new chlorins in total) have been synthesized and characterized with regards to photophysical properties. Five PEGylated chlorins were designed for aqueous solubility, wherein the narrow emission bands (fwhm ≤ 20 nm) and moderate Φ_f_ and τ_S_ values characteristic for analogues in organic solvents also were observed. Two amphiphilic chlorins bearing amino- or carboxylic acid-substitution have been examined in aqueous micellar media, where again spectroscopic features resembling those in organic solvents were observed. Loading of avidin with an average of 2–12 bioconjugatable chlorins afforded an approximate ~2-fold increase in Φ_f_ value across this span. One attraction of the de novo synthesis approach is the ability to achieve wavelength tunability across a family of chlorins, but doing so requires considerable synthetic effort. A less synthetically demanding route to a PEGylated bioconjugatable chlorin (without wavelength tunability) entails derivatization of a porphyrin [75]. While the design features for a palette of wavelength-tuned chlorins are now known to some extent, and approaches toward a number of desired architectural embodiments also have been established, an unsolved challenge entails how to create diverse chlorins in a concise and facile synthesis amenable to non-specialists. Accomplishing this objective is desirable for broad practical utilization of Nature’s chosen chromophores.

## Figures and Tables

**Table 1:** Spectral properties of PEGylated chlorins. *^a^*

Chlorin	Solvent	B_max_ Abs (nm)	Q_y_ Abs (fwhm) in nm	Q_y_ Em (fwhm) in nm	τ_S_ (ns)	Φ_f_	Φ_isc_	Φ_ic_	k_f_^−1^ (ns)	k_isc_^−1^ (ns)	k_ic_^−1^ (ns)
**H_2_C10-PEG_6_**	toluene *^b^*	413	644 (14)	647 (15)	9.3	0.25	0.61	0.14	37	15	66
**H_2_C10-PEG_6_**	DMF	412	642 (14)	645 (16)	9.3	0.26	0.62	0.12	36	15	78
**H_2_C10-PEG_6_**	water	409	640 (16)	644 (18)	7.4 *^c^*	0.20 *^d^*	0.52	0.28	37	14	26
**H_2_C12-PEG_6_**	water	409	640 (17)	644 (18)	7.6 *^b^*	0.23	0.70	0.07	33	11	110
**ZnC12-PEG_6_**	toluene *^b^*	415	612 (18)	616 (20)	2.4	0.092	0.87	0.04	22	2.8	63
**ZnC12-PEG_6_**	DMF	415	613 (20)	617 (21)	1.9	0.065	0.84	0.10	29	2.3	20
**ZnC12-PEG_6_**	water	413	613 (20)	619 (20)	2.0 *^c^*	0.060	0.80	0.14	33	2.5	14
**H_2_C15-PEG_6_**	water	411	650 (31)	654 (20)	6.2 *^c^*	0.20	0.67	0.13	31	9.3	48
**ZnC15-PEG_6_**	toluene *^b^*	416	625 (26)	627 (22)	2.8	0.11	0.74	0.15	25	3.8	19
**ZnC15-PEG_6_**	DMF	415	625 (18)	628 (22)	2.4	0.090	0.72	0.18	27	3.3	13
**ZnC15-PEG_6_**	water	413	625 (22)	630 (19)	2.8 *^c^*	0.11	0.67	0.22	25	4.2	13

*^a^* All measurements at room temperature in the presence of ambient O_2_. The typical errors (percent of value) are τ_S_ (±5%), Φ_f_ (±5%), Φ_isc_ (±10%), Φ_ic_ (±15), k_f_ (±10%), k_isc_ (±15%), k_ic_ (±20%). *^b^* Pre-solubilized with a small amount of DMF. *^c^* Measured in standard aqueous phosphate-buffered saline (PBS) solution. *^d^* Average of values in deionized water and PBS.

**Table 2:** Spectral properties of amphiphilic chlorins. *^a^*

Chlorin	Solvent	B_max_ Abs (nm)	Q_y_ Abs (fwhm) in nm	Q_y_ Em (fwhm) in nm	τ_S_ (ns)	Φ_f_	Φ_isc_	Φ_ic_	k_f_^−1^ (ns)	k_isc_^−1^ (ns)	k_ic_^−1^ (ns)
**H_2_C2**	toluene	415	641 (10)	643 (13)	9.3	0.23	0.70	0.07	40	13	130
**H_2_C2**	DMF	413	638 (11)	641 (16)	9.8	0.18	0.74	0.08	54	13	120
**H_2_C3**	toluene	414	641 (10)	642 (12)	7.1	0.18	0.72	0.10	39	10	71
**H_2_C3**	DMF	412	639 (12)	640 (13)	8.4	0.18	0.71	0.11	47	12	76
**H_2_C3**	CTAB *^b^*	413	641 (12)	641 (20)	7.9	0.16	0.73	0.11	49	11	72
**H_2_C6**	toluene	427	667 (15)	672 (18)	6.4	0.29	0.62	0.090	22	10	71
**H_2_C6**	DMF	424	662 (18)	667 (20)	6.5	0.26	0.63	0.11	25	10	59
**H_2_C6**	CTAB	426	666 (19)	669 (22)	6.4	0.24	0.44	0.32	27	15	20

*^a^* All measurements at room temperature in the presence of ambient O_2_. The typical errors (percent of value) are τ_S_ (±5%), Φ_f_ (±5%), Φ_isc_ (±10%), Φ_ic_ (±15), k_f_ (±10%), k_isc_ (±15%), k_ic_ (±20%). *^b^* 10 mM CTAB micellar solution in 0.1 M sodium phosphate buffer (pH = 7.0).

**Table 3:** Loading of **H_2_C12-PEG_6_-NHS** on avidin.

Entry	[Chlorin]	[Avidin]	Ratio *^a^*	Loading *^b^*	Φ_f_ of Conjugate *^c^*
1	2.4 mM	79 µM	30	2.3	0.075
2	4.8 mM	79 µM	60	6.2	0.13
3	7.2 mM	79 µM	90	12	0.17

*^a^* Ratio of chlorin/avidin reactants. *^b^* Observed average loading of chlorins/avidin. *^c^* Determined using λ_exc_ = 505 nm and integration of the corrected emission (530–800 nm).

## Supplementary Materials

^1^H- and ^13^C-NMR spectra, MALDI-MS and ESI-MS data for new chlorins.
