# Supplementary material for: Chlorophyll-Inspired Red-Region Fluorophores: Building Block Synthesis and Studies in Aqueous Media
Source: Molecules. 2018 Jan 10;23(1):130. doi: 10.3390/molecules23010130 (PMC6017558; doi:10.3390/molecules23010130)

Supporting Information for:

Chlorophyll-Inspired Red-Region Fluorophores: Building Block Synthesis and Studies in Aqueous Media

Rui Liu, Mengran Liu, Don Hood, Chih-Yuan Chen, Christopher J. MacNevin,

Dewey Holten and Jonathan S. Lindsey

Table of Contents

**Topic**	**Pages**
Cyclization data for **H_2C10-PEG$_6$-NHS**	S2, S3
Spectral data for new compounds	S4–S70

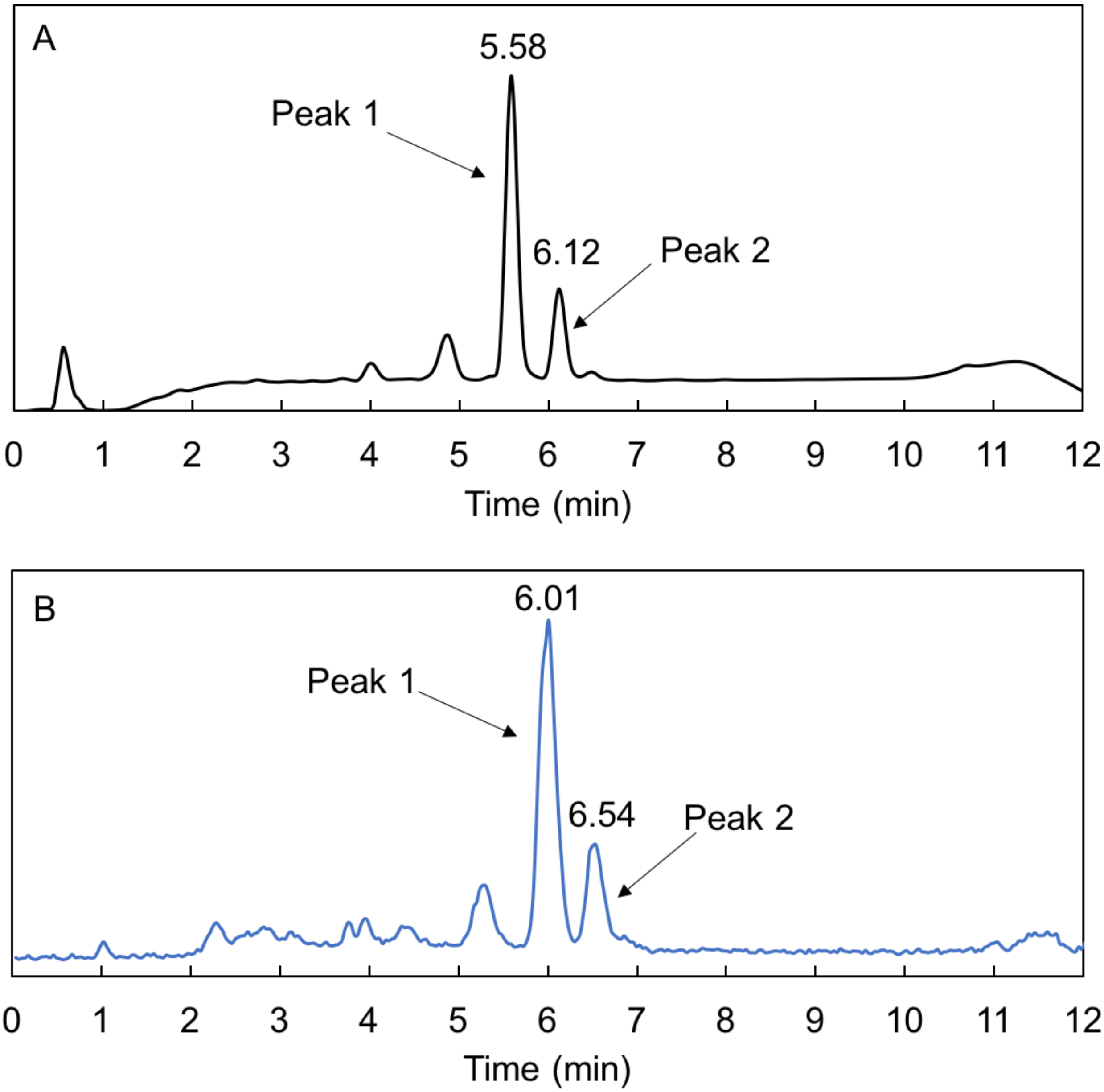
A
5.58
Peak 1
6.12
Peak 2
0 1 2 3 4 5 6 7 8 9 10 11 12
Time (min)
B
6.01
Peak 1
6.54
Peak 2
0 1 2 3 4 5 6 7 8 9 10 11 12
Time (min)

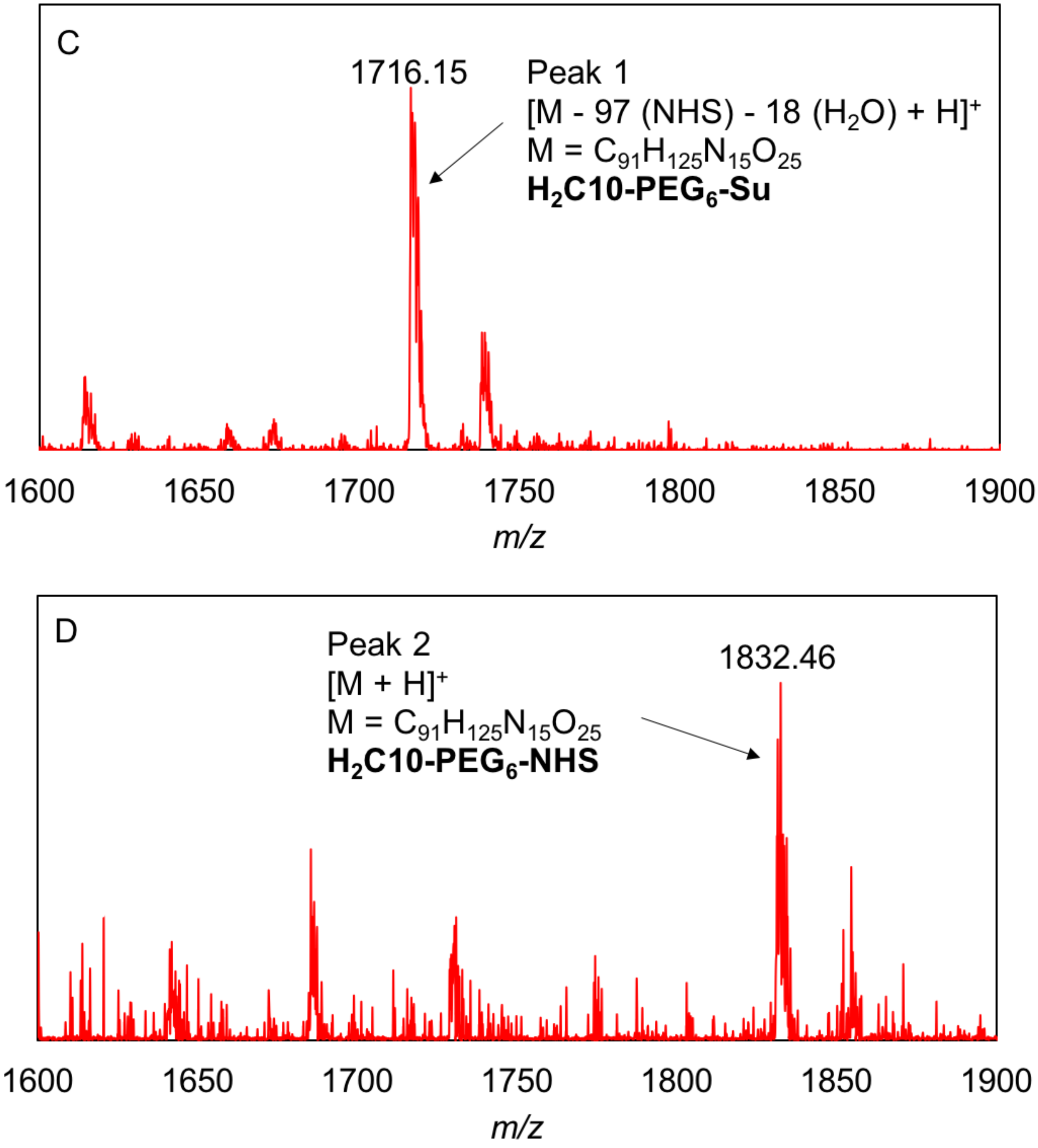

Figure S1. Chromatogram of **H_2C10-PEG$_6$-NHS** with detection by absorption spectroscopy (200 – 800 nm) (panel A) followed by ESI-MS with total ion current (panel B). ESI-MS data of peak 1 is shown in panel C, whereas peak 2 (panel D) is consistent with **H_2C10-PEG$_6$-NHS**.

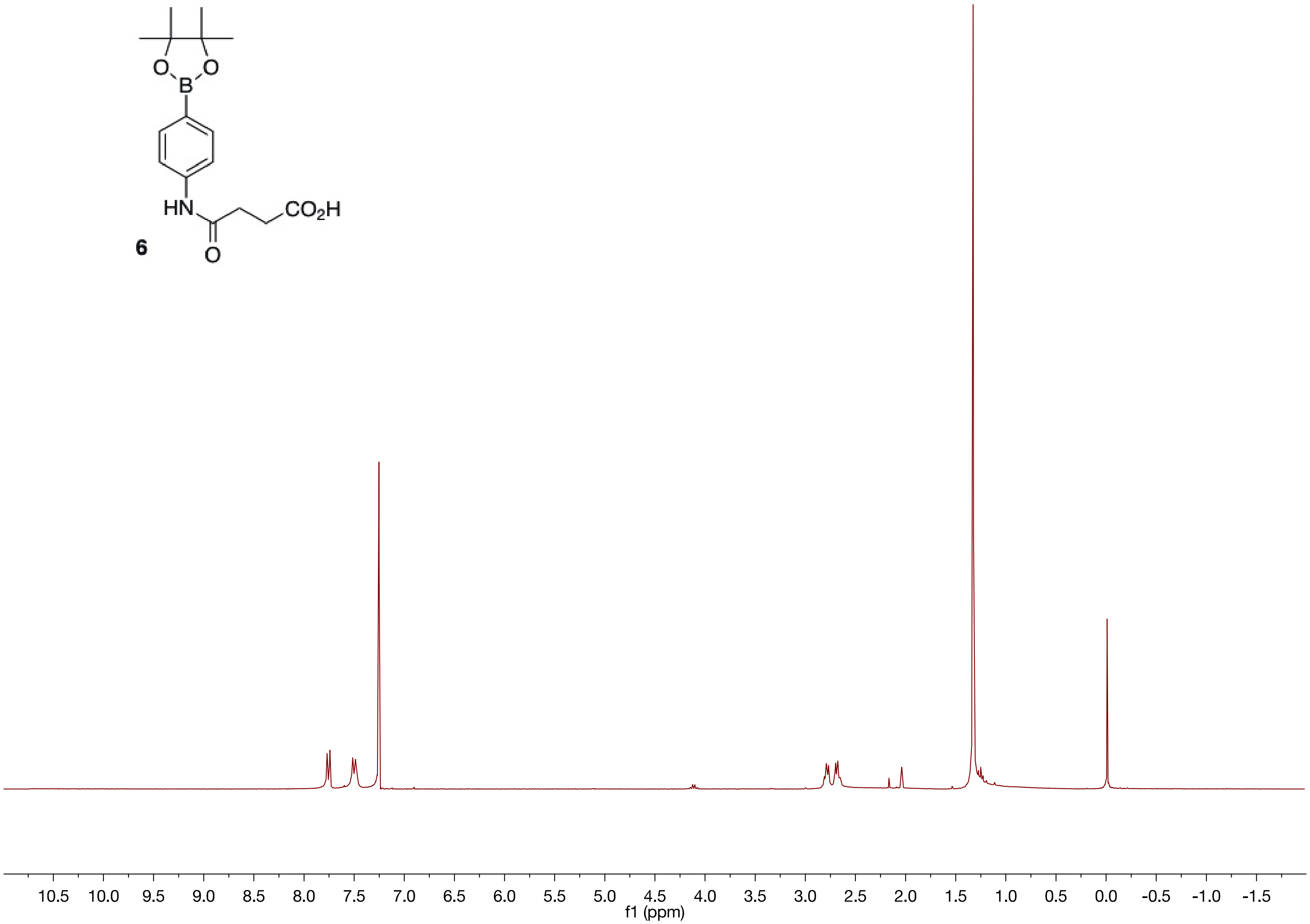
O
B
O
HN
CO2H
O
6
10.5
10.0
9.5
9.0
8.5
8.0
7.5
7.0
6.5
6.0
5.5
5.0
4.5
4.0
3.5
3.0
2.5
2.0
1.5
1.0
0.5
0.0
-0.5
-1.0
-1.5
f1 (ppm)

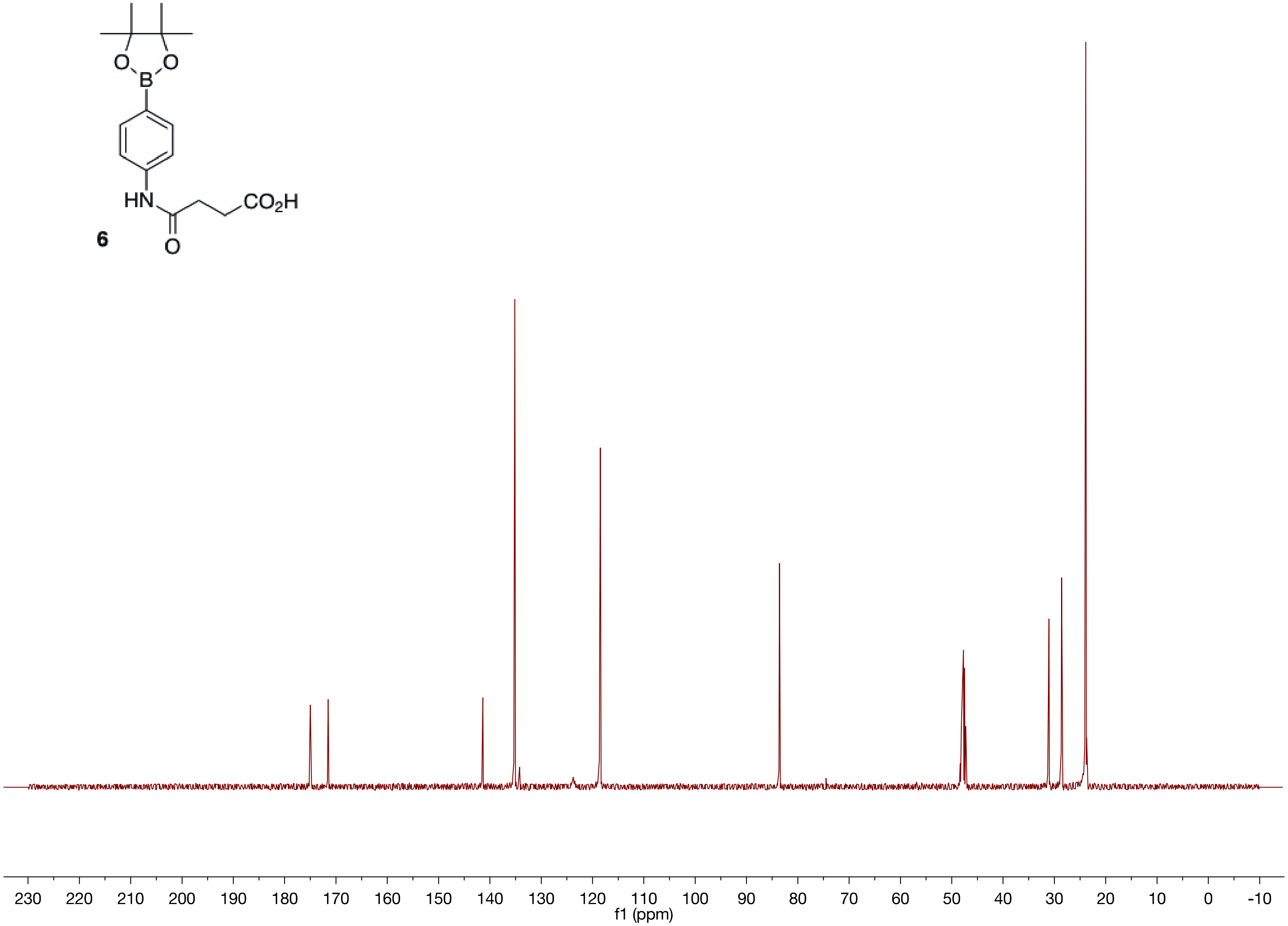
O
B
O
HN
CO2H
6
O
230 220 210 200 190 180 170 160 150 140 130 120 110 100 90 80 70 60 50 40 30 20 10 0 -10
f1 (ppm)

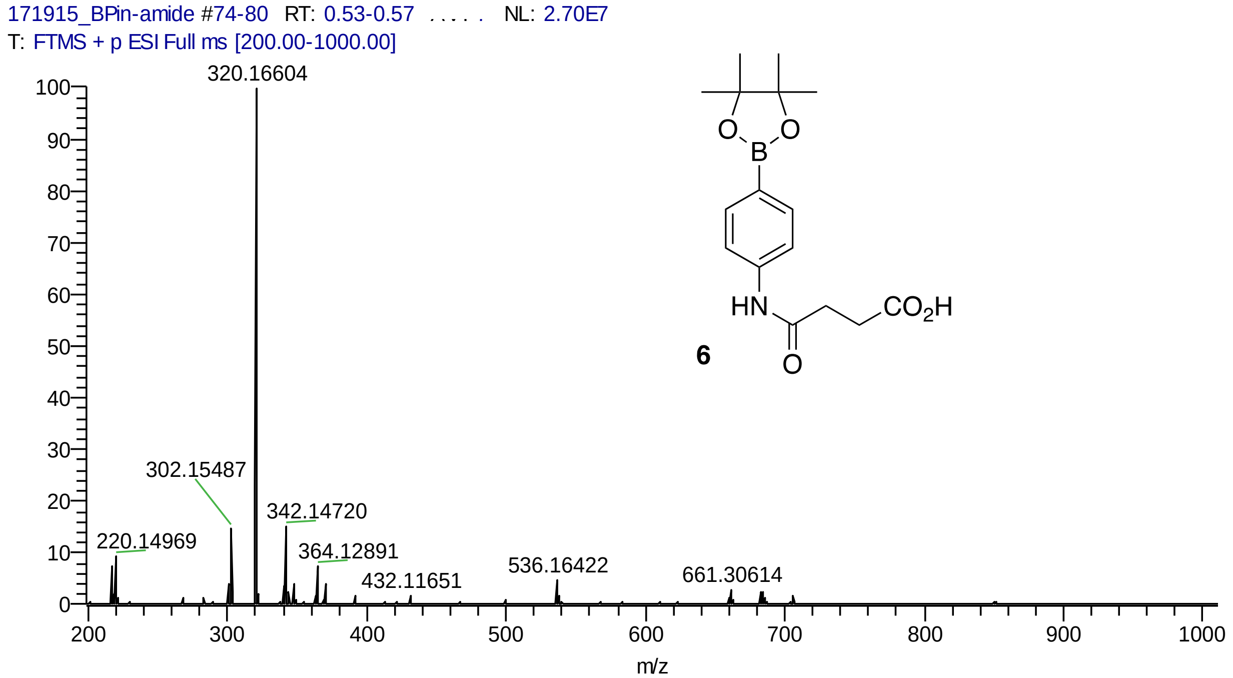
171915_BPin-amide #74-80 RT: 0.53-0.57 . . . . . NL: 2.70E7
T: FTMS + p ESI Full ms [200.00-1000.00]
320.16604
302.15487
342.14720
220.14969
364.12891
432.11651
536.16422
661.30614
m/z
HN
CO_2H
O
B
6

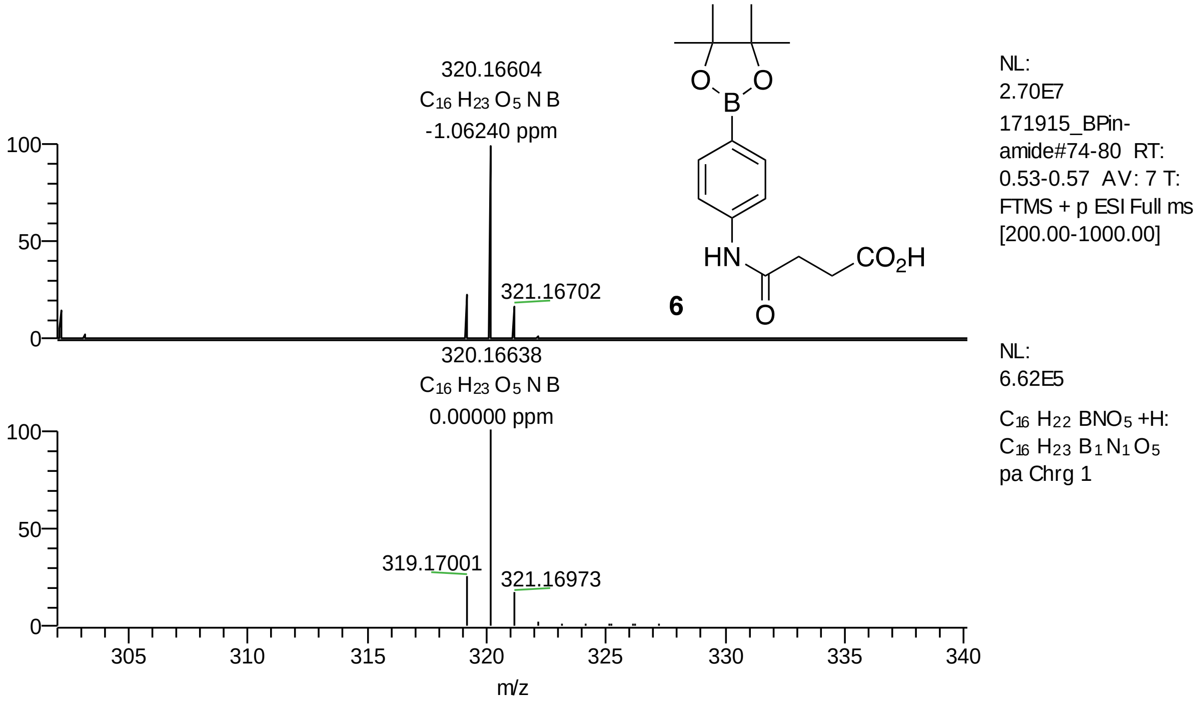
320.16604
C16 H23 O5 N B
-1.06240 ppm
321.16702
320.16638
C16 H23 O5 N B
0.00000 ppm
319.17001
321.16973
m/z
NL:
2.70E7
171915_BPin-amide#74-80 RT: 0.53-0.57 AV: 7 T: FTMS + p ESI Full ms [200.00-1000.00]
NL:
6.62E5
C16 H22 BNO5 +H:
C16 H23 B1 N1 O5
pa Chrg 1
HN
CO_2H
6

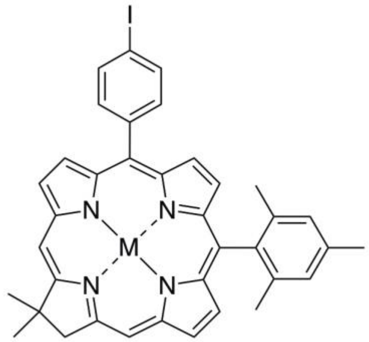

ZnC1, M = Zn

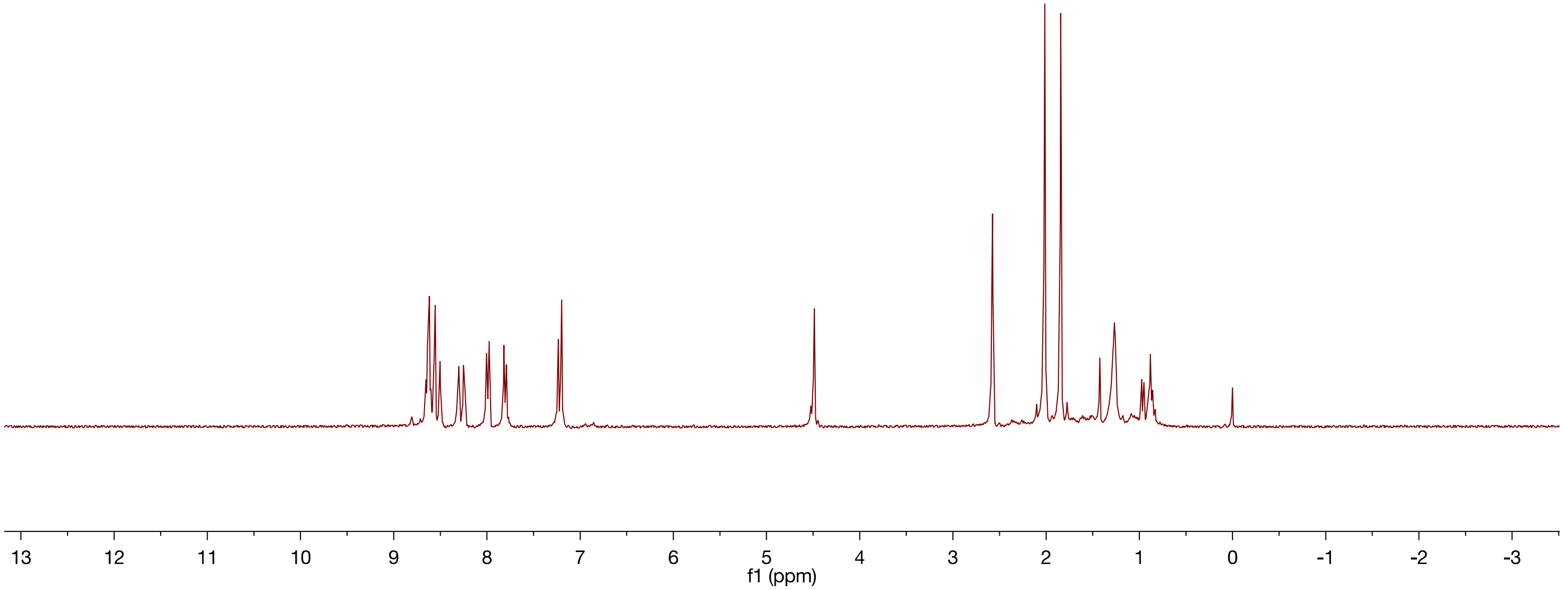

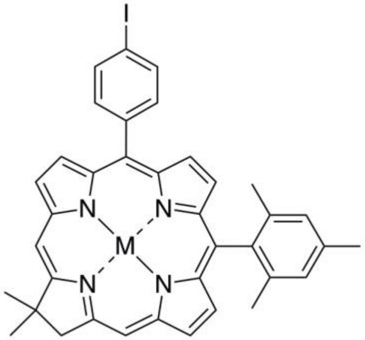

ZnC1, M = Zn

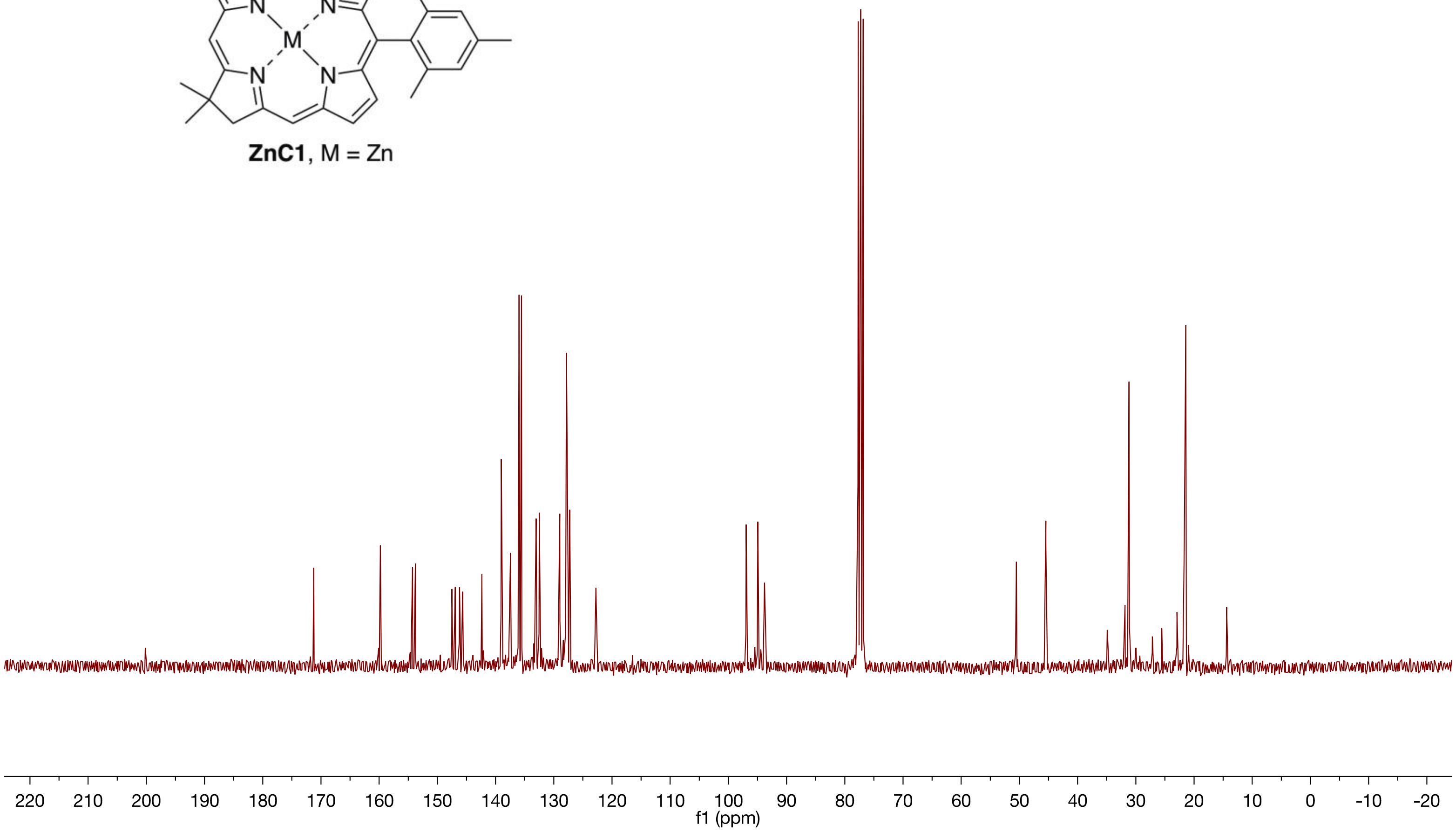
220 210 200 190 180 170 160 150 140 130 120 110 100 90 80 70 60 50 40 30 20 10 0 -10 -20
f1 (ppm)

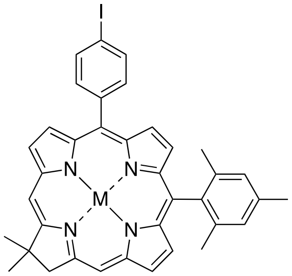

ZnC1
M = Zn

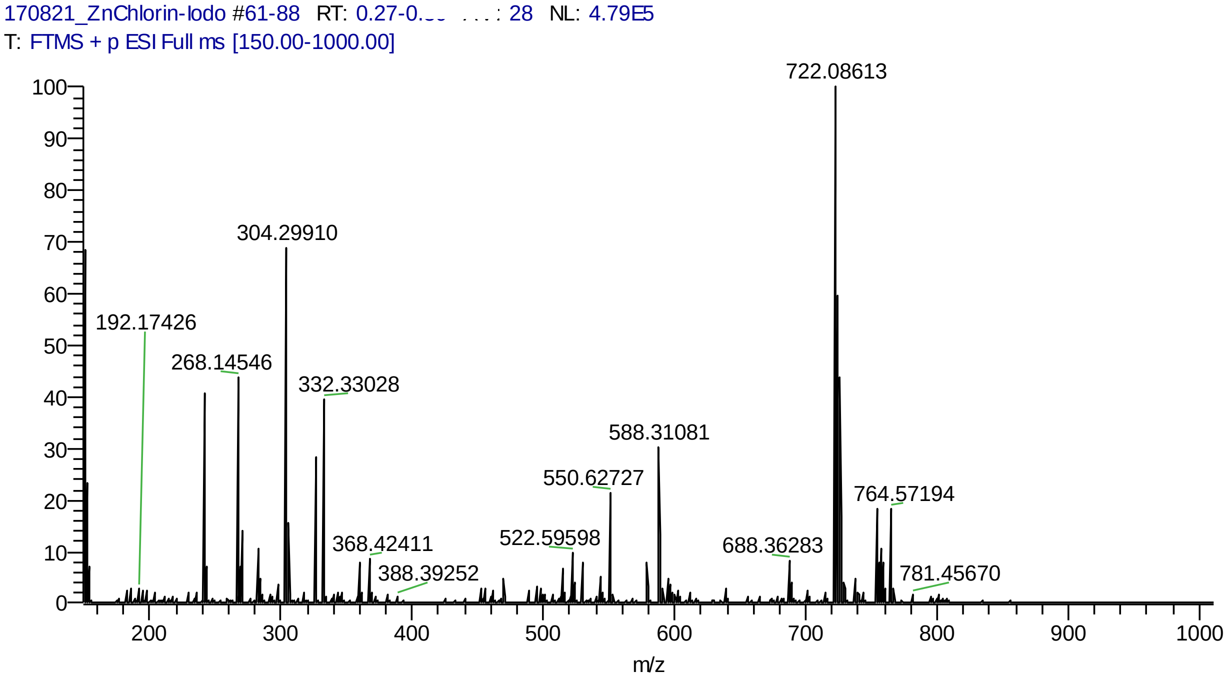

170821_ZnChlorin-Iodo #61-88 RT: 0.27-0. : 28 NL: 4.79E5
T: FTMS + p ESI Full ms [150.00-1000.00]
722.08613
304.29910
192.17426
268.14546
332.33028
588.31081
550.62727
764.57194
368.42411
522.59598
688.36283
388.39252
781.45670
m/z

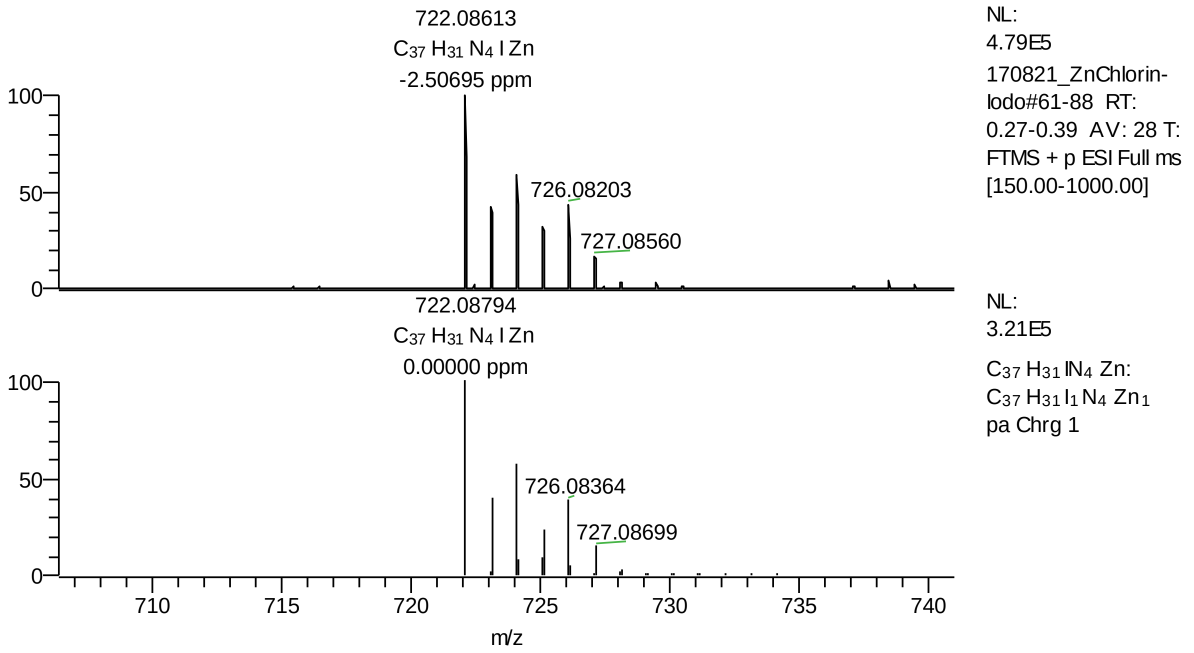

722.08613
C37 H31 N4 I Zn
-2.50695 ppm
726.08203
727.08560
NL:
4.79E5
170821_ZnChlorin-Iodo#61-88 RT: 0.27-0.39 AV: 28 T: FTMS + p ESI Full ms [150.00-1000.00]
722.08794
C37 H31 N4 I Zn
0.00000 ppm
726.08364
727.08699
NL:
3.21E5
C37 H31 IN4 Zn:
C37 H31 I1 N4 Zn1
pa Chrg 1
m/z

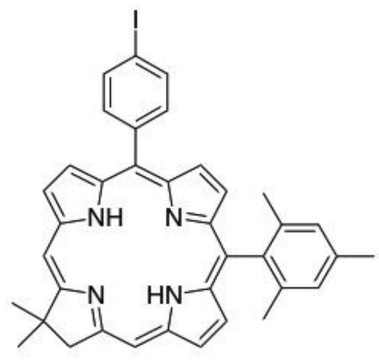

H_2C1

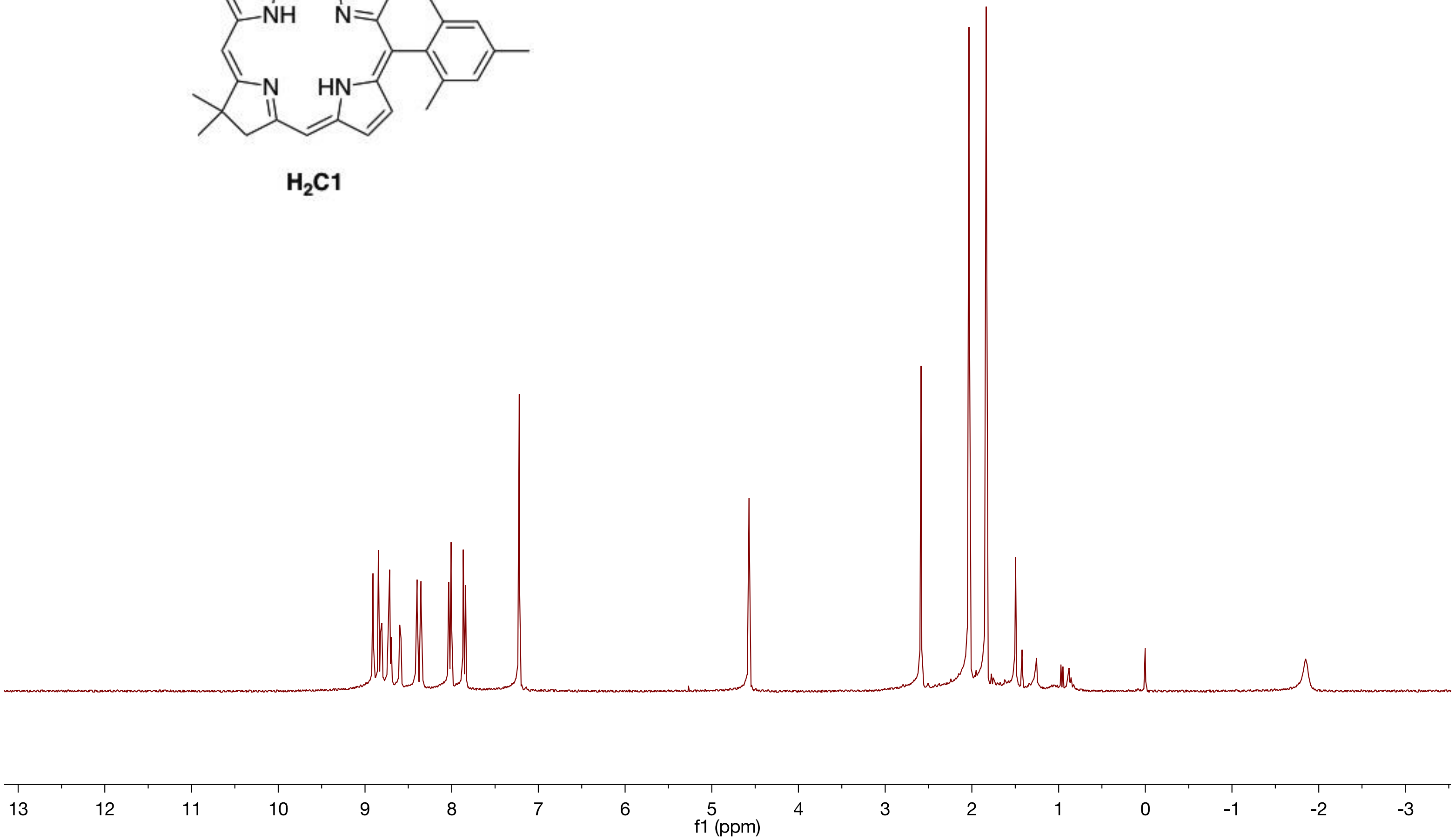

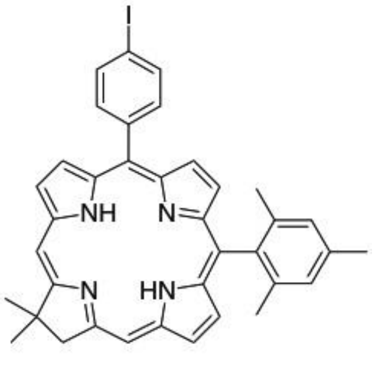
I
NH
N
N
HN

H_2C1

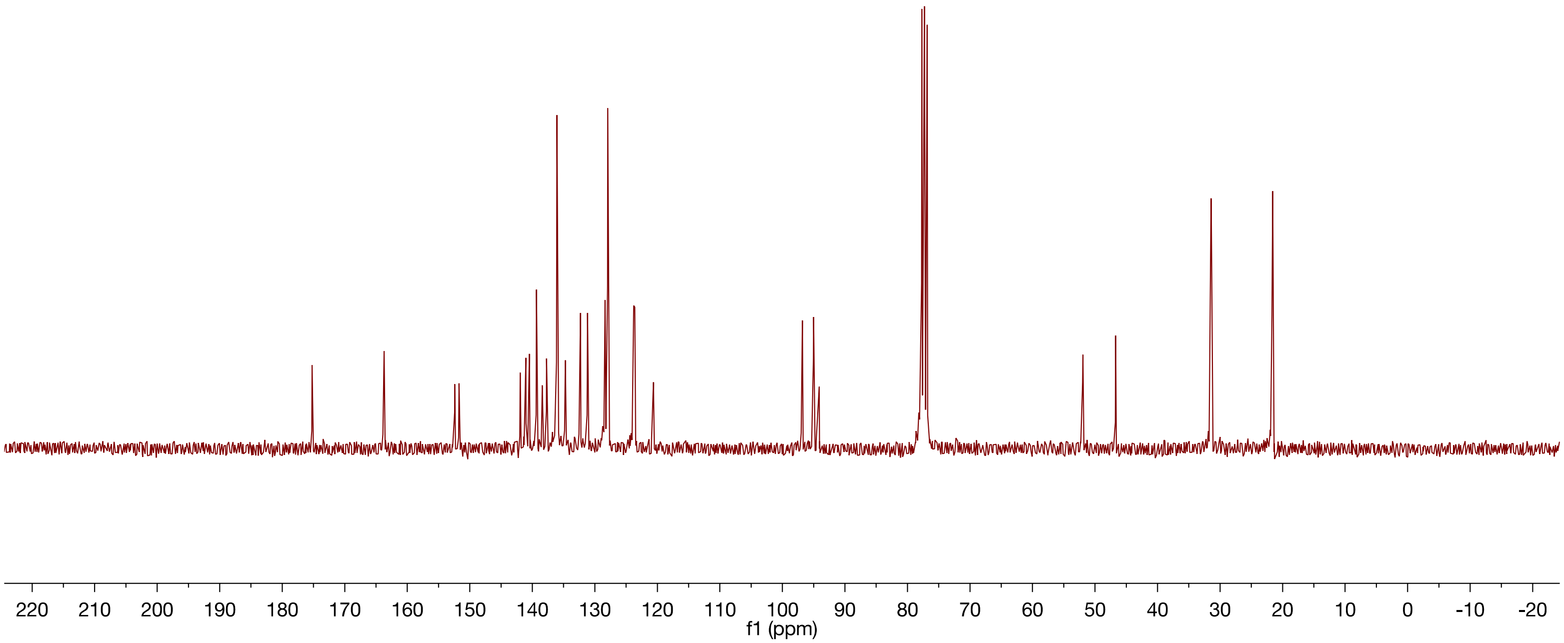
220 210 200 190 180 170 160 150 140 130 120 110 100 90 80 70 60 50 40 30 20 10 0 -10 -20
f1 (ppm)

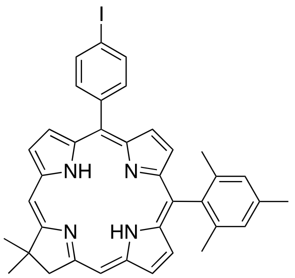
I
NH
N
N
HN

H_2C1

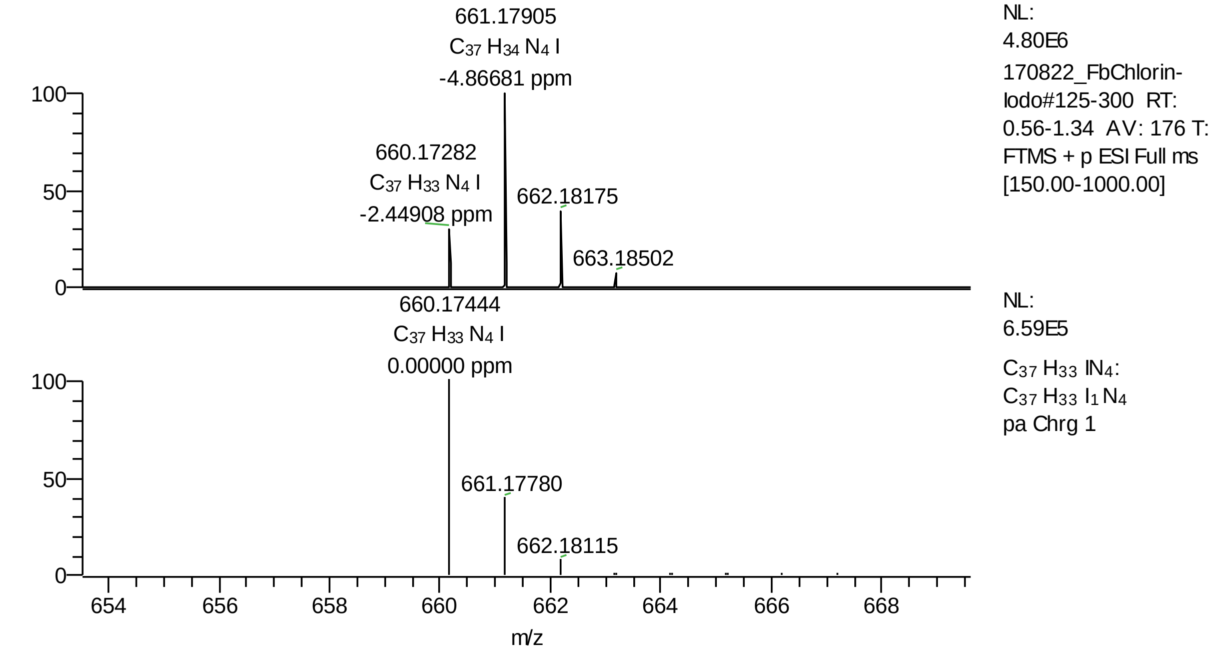
661.17905
C37 H34 N4 I
-4.86681 ppm
660.17282
C37 H33 N4 I
-2.44908 ppm
662.18175
663.18502
NL:
4.80E6
170822_FbChlorin-Iodo#125-300 RT: 0.56-1.34 AV: 176 T: FTMS + p ESI Full ms [150.00-1000.00]
660.17444
C37 H33 N4 I
0.00000 ppm
661.17780
662.18115
NL:
6.59E5
C37 H33 IN4:
C37 H33 I1 N4
pa Chrg 1
100
50
0
654
656
658
660
662
664
666
668
m/z

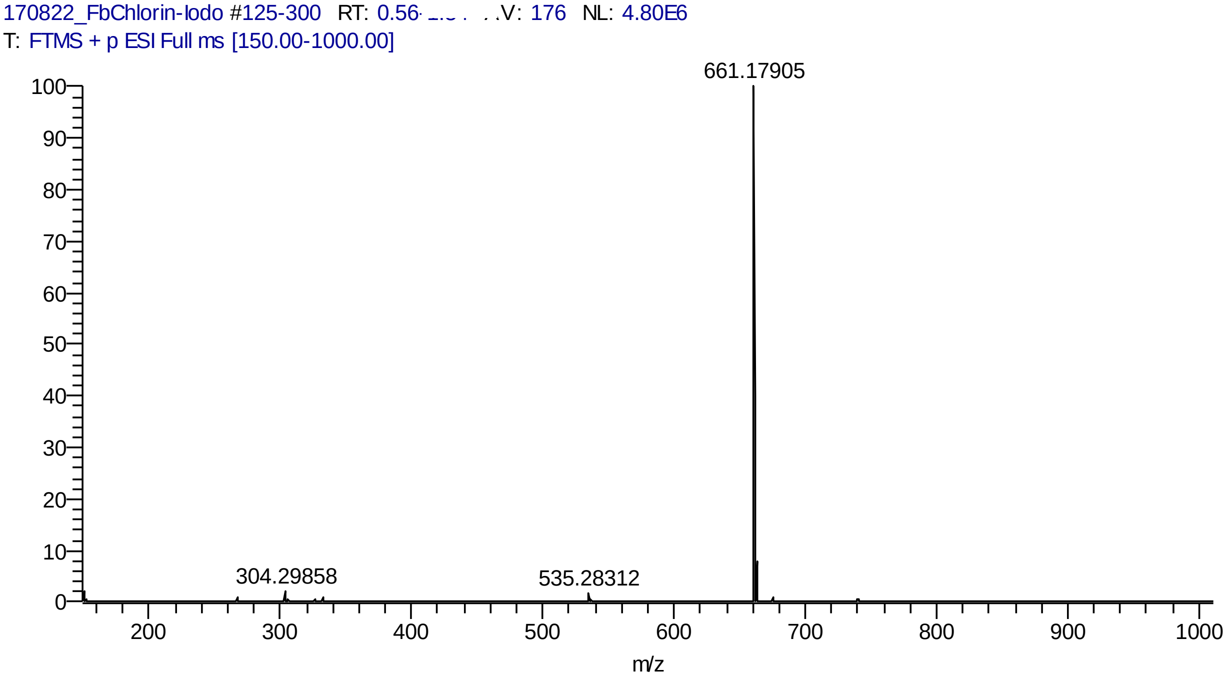
170822_FbChlorin-Iodo #125-300 RT: 0.56
V: 176 NL: 4.80E6
T: FTMS + p ESI Full ms [150.00-1000.00]
661.17905
304.29858
535.28312
100
90
80
70
60
50
40
30
20
10
0
200
300
400
500
600
700
800
900
1000
m/z

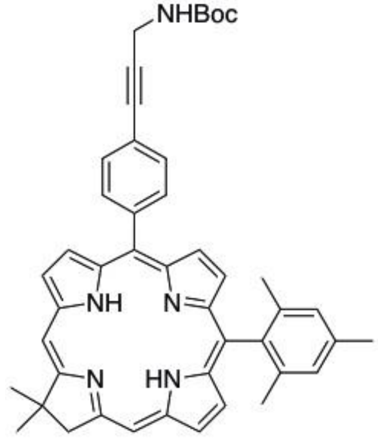

H_2C2

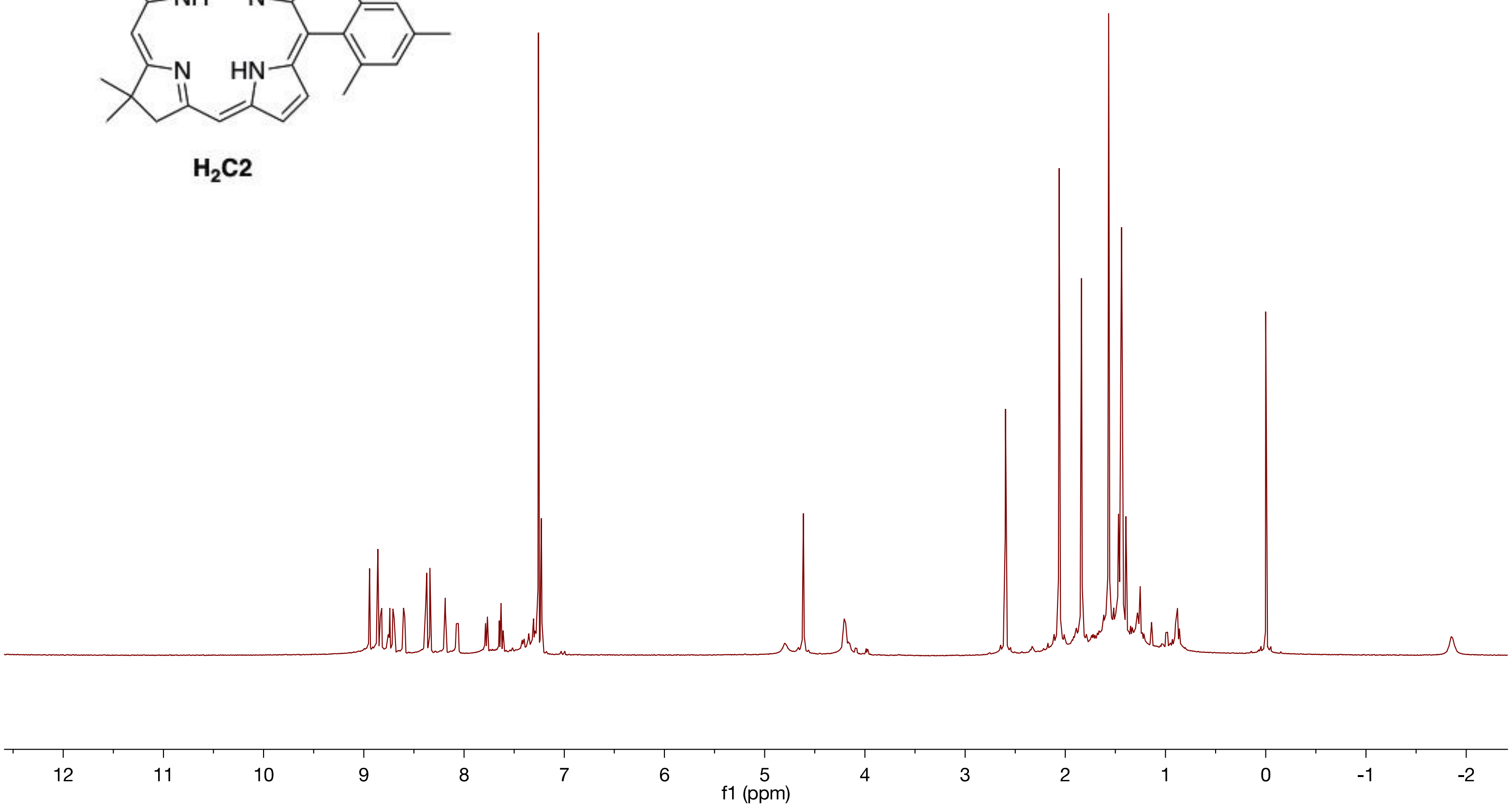

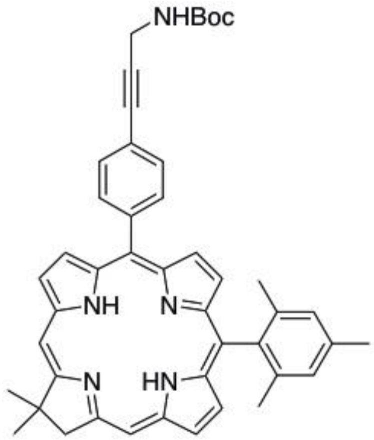
NHBoc
NH
N
N
HN

H_2C2

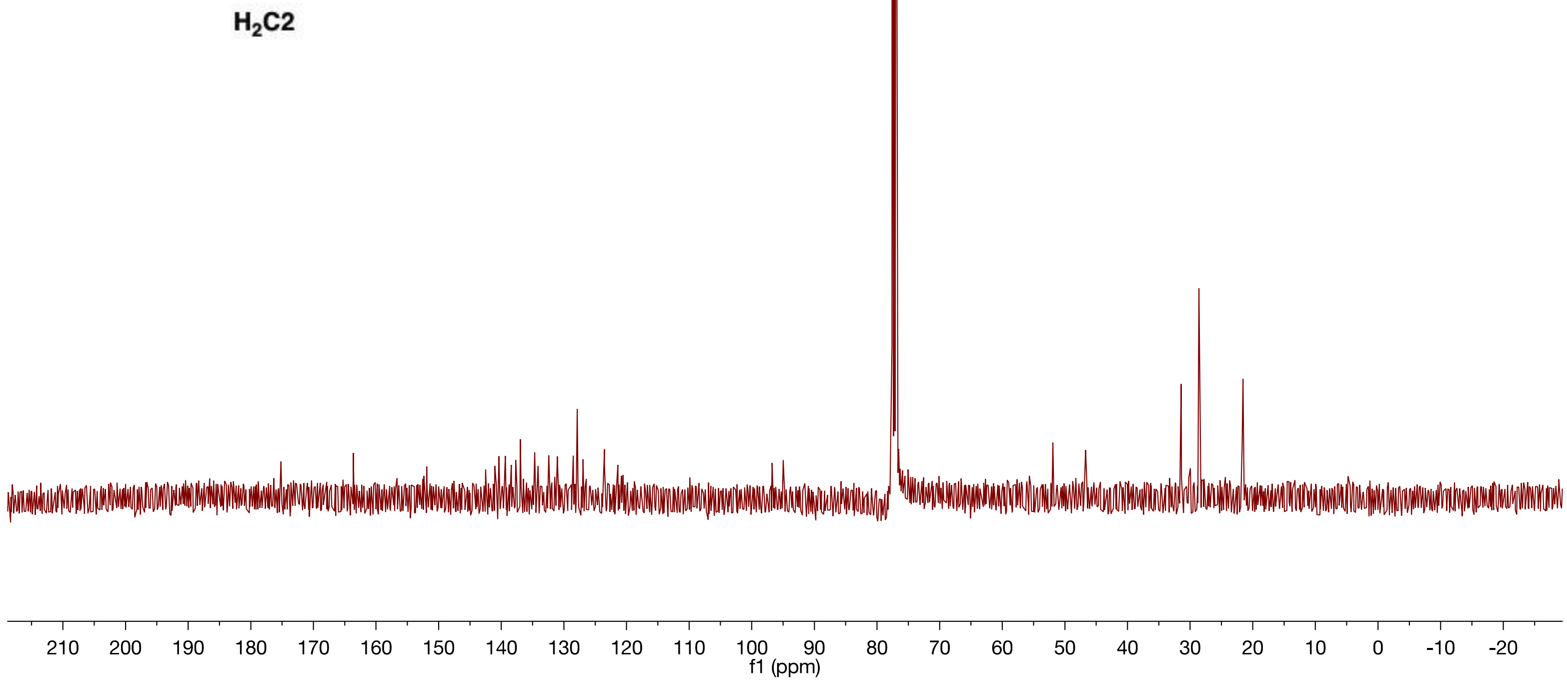
210
200
190
180
170
160
150
140
130
120
110
100
90
80
70
60
50
40
30
20
10
0
-10
-20
f1 (ppm)

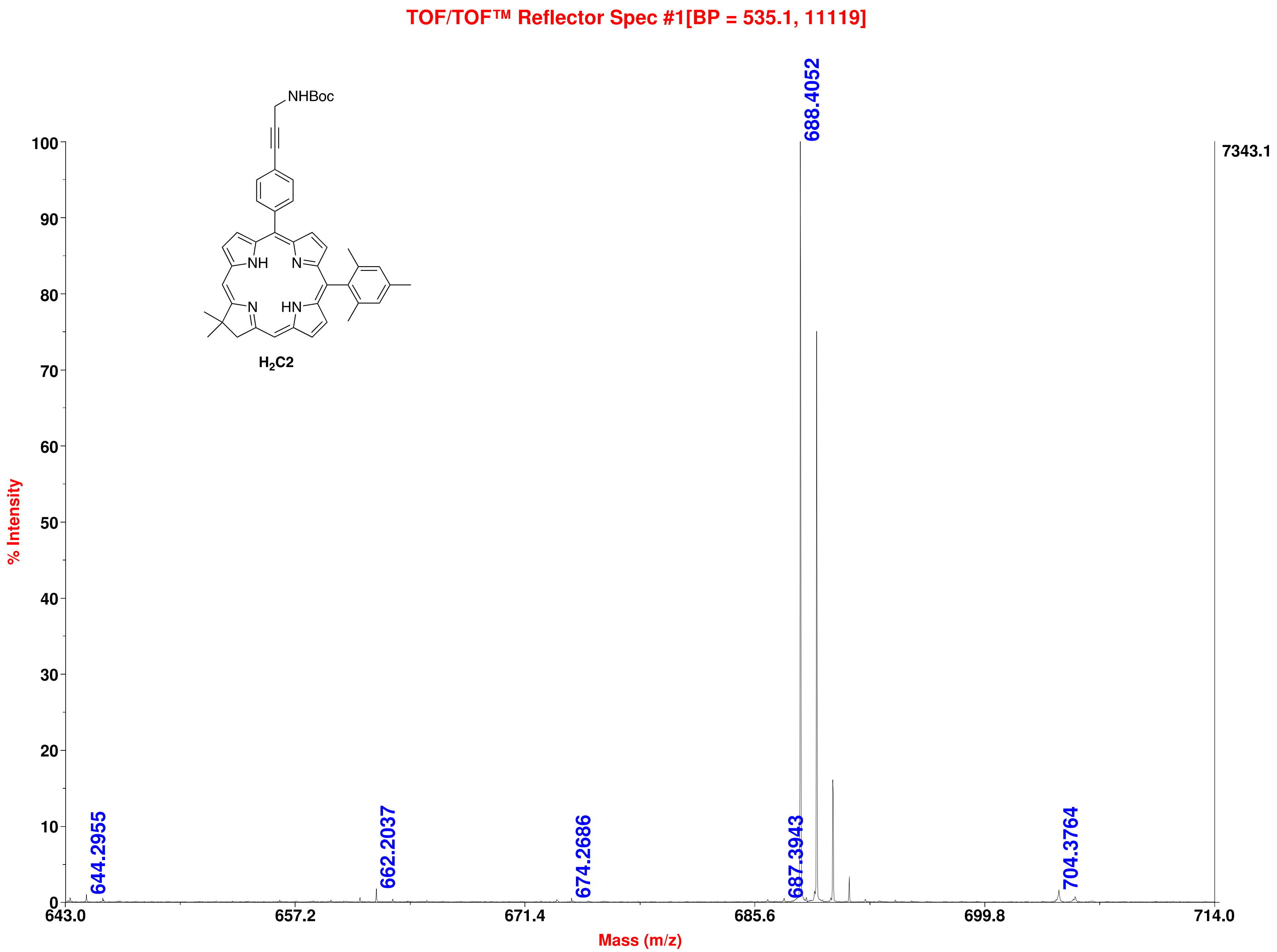

F:\...\BocChlorin.T2D
Acquired:

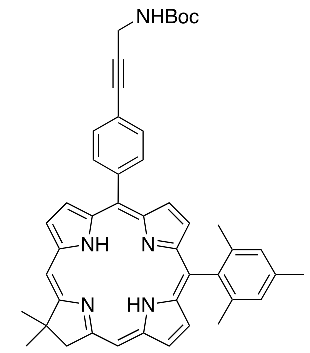

H_2C2

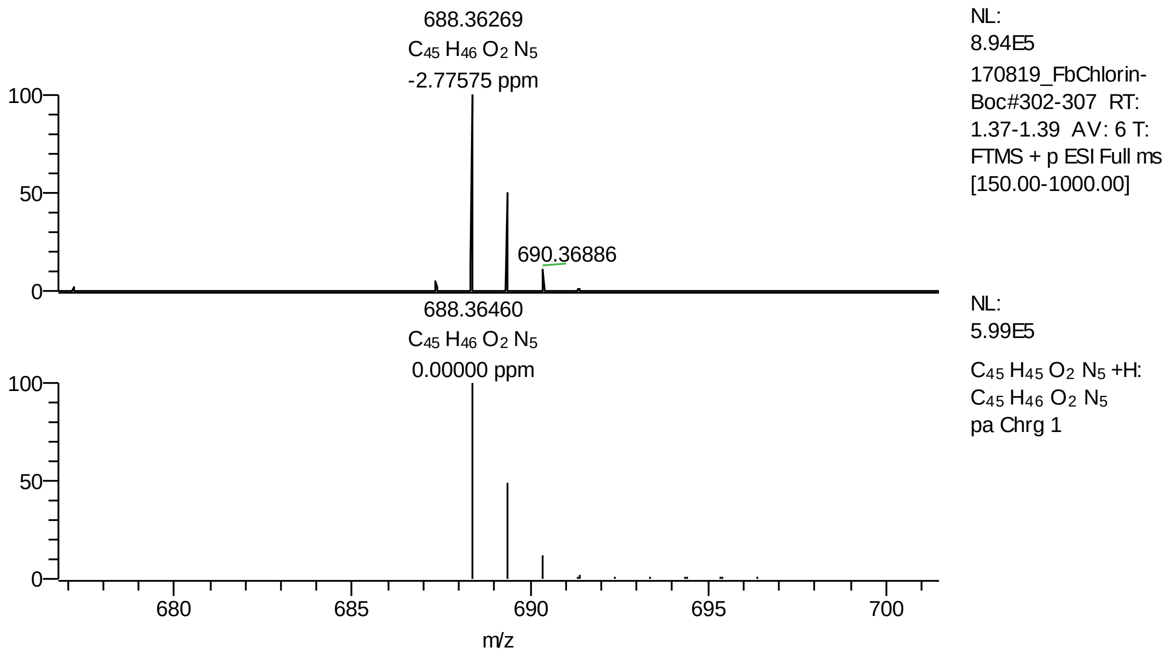

688.36269
C45 H46 O2 N5
-2.77575 ppm
690.36886
688.36460
C45 H46 O2 N5
0.00000 ppm
m/z
NL:
8.94E5
170819_FbChlorin-Boc#302-307 RT: 1.37-1.39 AV: 6 T: FTMS + p ESI Full ms [150.00-1000.00]
NL:
5.99E5
C45 H45 O2 N5 +H:
C45 H46 O2 N5
pa Chrg 1

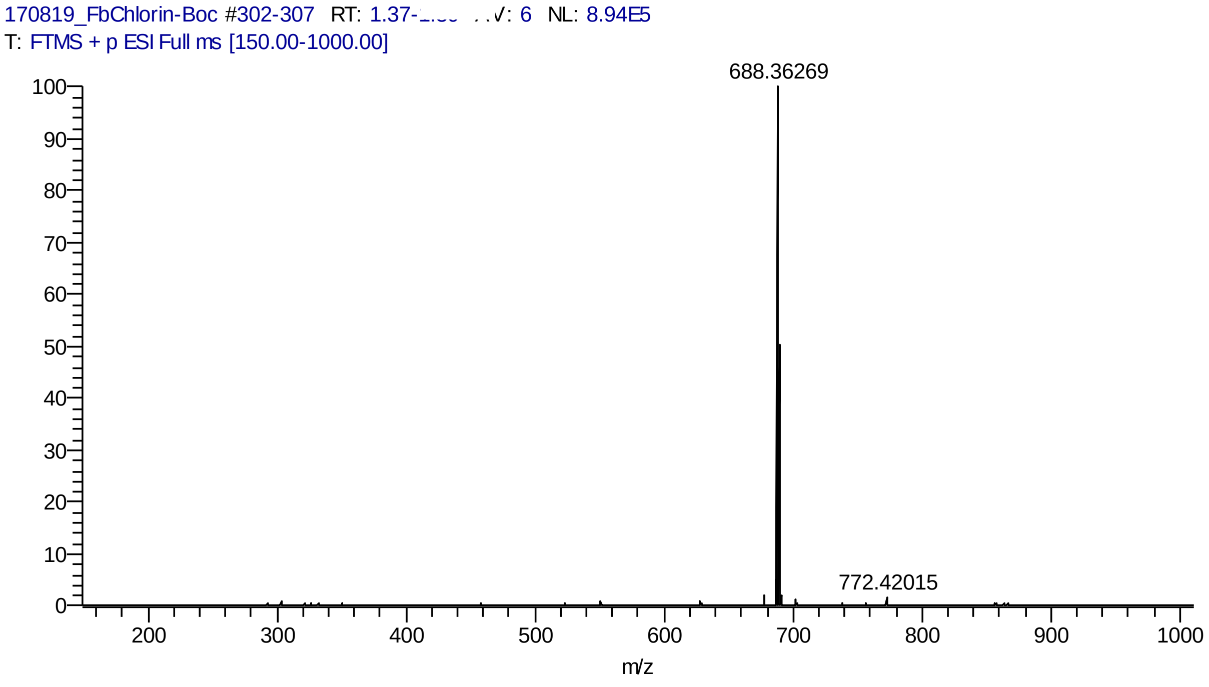

170819_FbChlorin-Boc #302-307 RT: 1.37-
V: 6 NL: 8.94E5
T: FTMS + p ESI Full ms [150.00-1000.00]
688.36269
772.42015
m/z

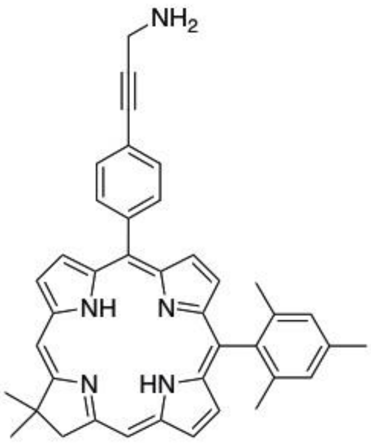

H_2C3

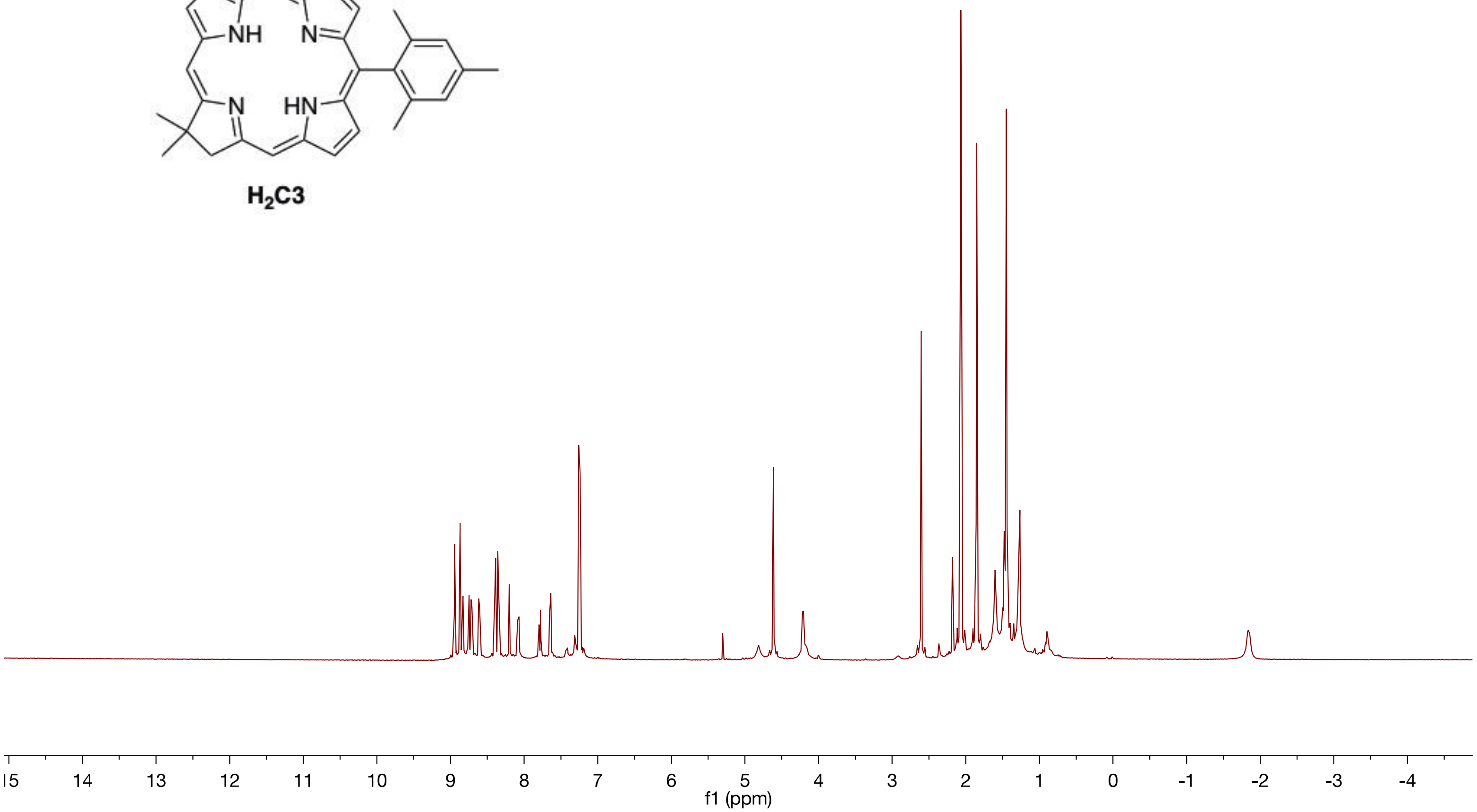

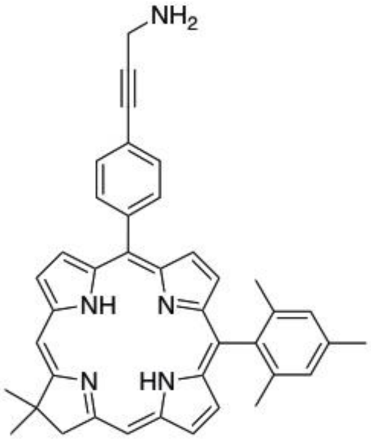
NH2
NH
N
N
HN

H_2C3

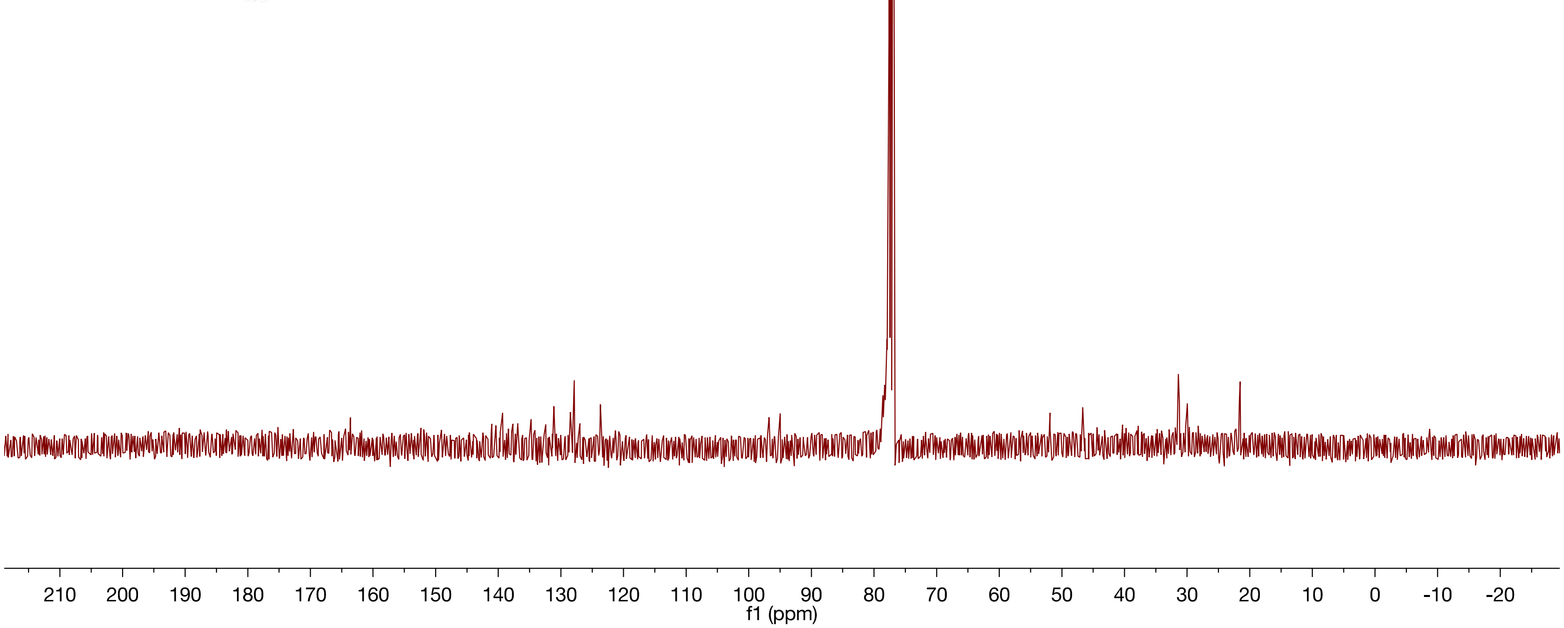
210
200
190
180
170
160
150
140
130
120
110
100
90
80
70
60
50
40
30
20
10
0
-10
-20
f1 (ppm)

AB Sciex TOF/TOF™ Series Explorer™ 72098

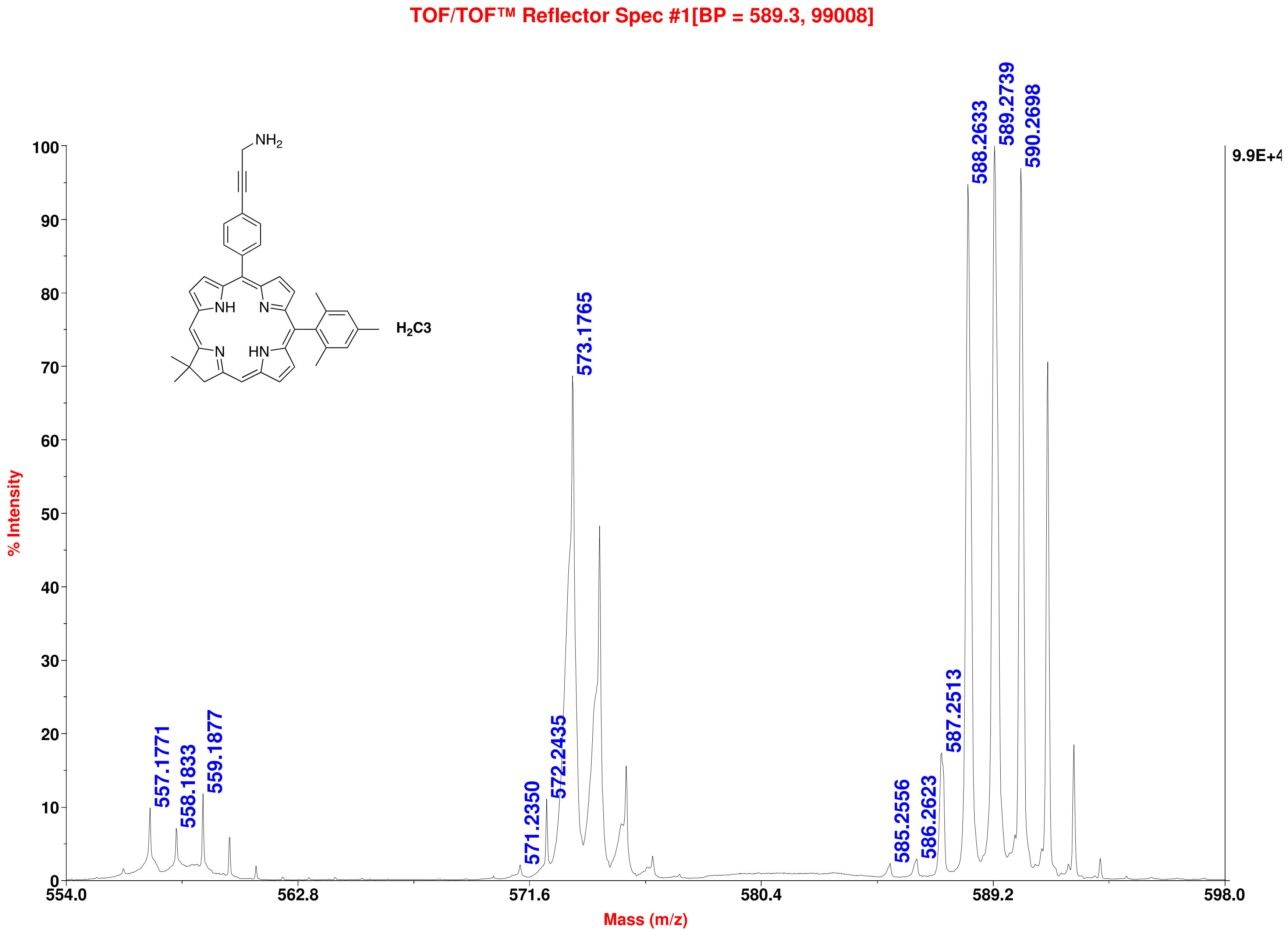

F:\User Project 1\Lindsey\RLiu\2016\051216\Chlorin-amine crude.T2D

Printed: 11:20, February 22, 2017

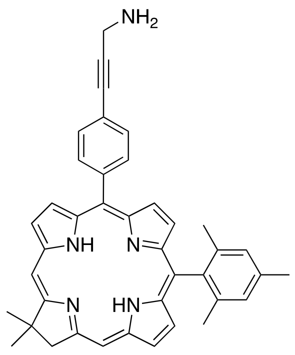

H_2C3

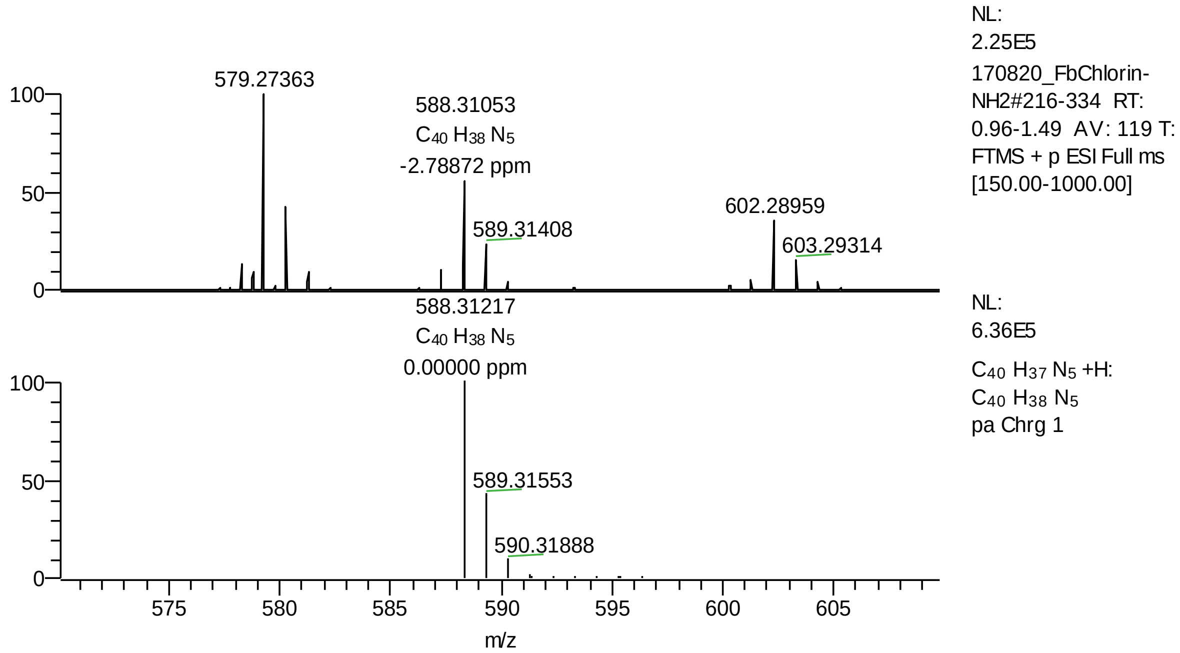

NL:
2.25E5
170820_FbChlorin-NH2#216-334 RT: 0.96-1.49 AV: 119 T: FTMS + p ESI Full ms [150.00-1000.00]
579.27363
588.31053
C40 H38 N5
-2.78872 ppm
589.31408
602.28959
603.29314
NL:
6.36E5
C40 H37 N5 +H:
C40 H38 N5
pa Chrg 1
588.31217
C40 H38 N5
0.00000 ppm
589.31553
590.31888
m/z

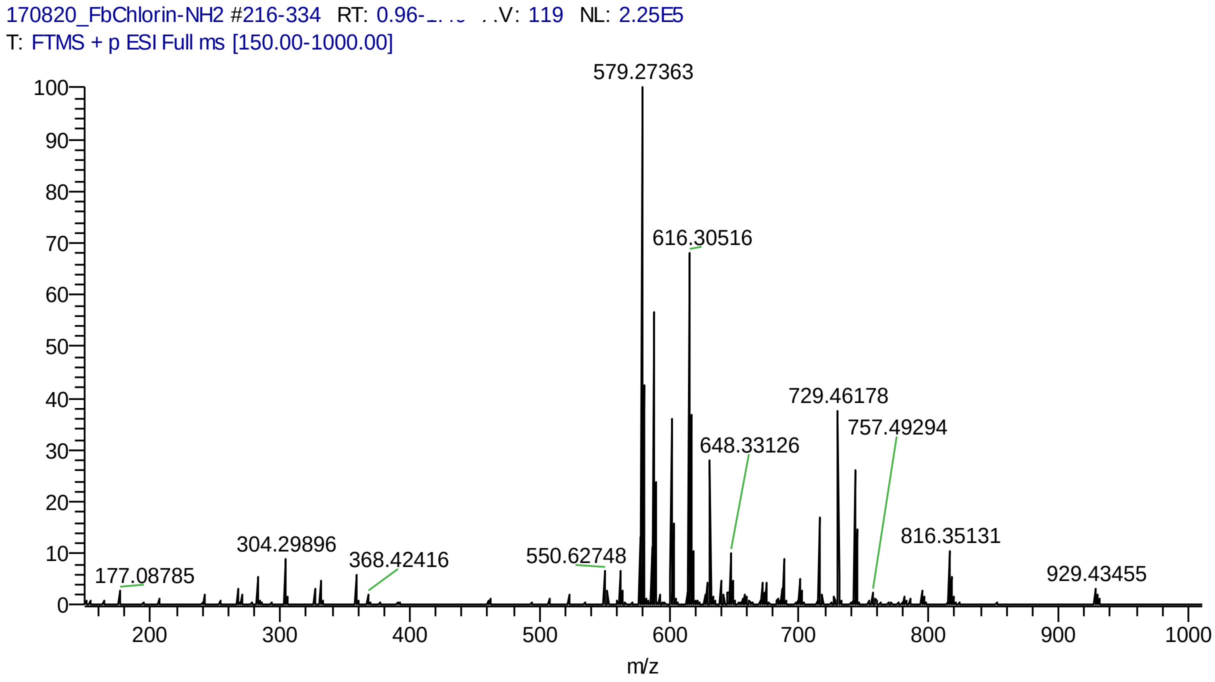

170820_FbChlorin-NH2 #216-334 RT: 0.96-
V: 119 NL: 2.25E5
T: FTMS + p ESI Full ms [150.00-1000.00]
579.27363
616.30516
729.46178
757.49294
648.33126
816.35131
304.29896
368.42416
550.62748
177.08785
929.43455
m/z

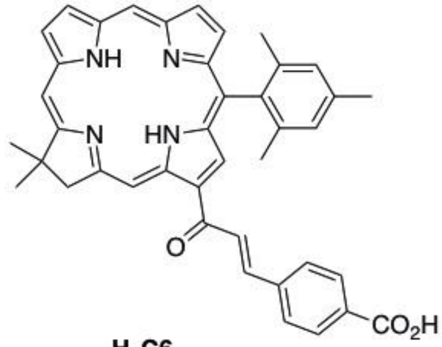

H_2C6

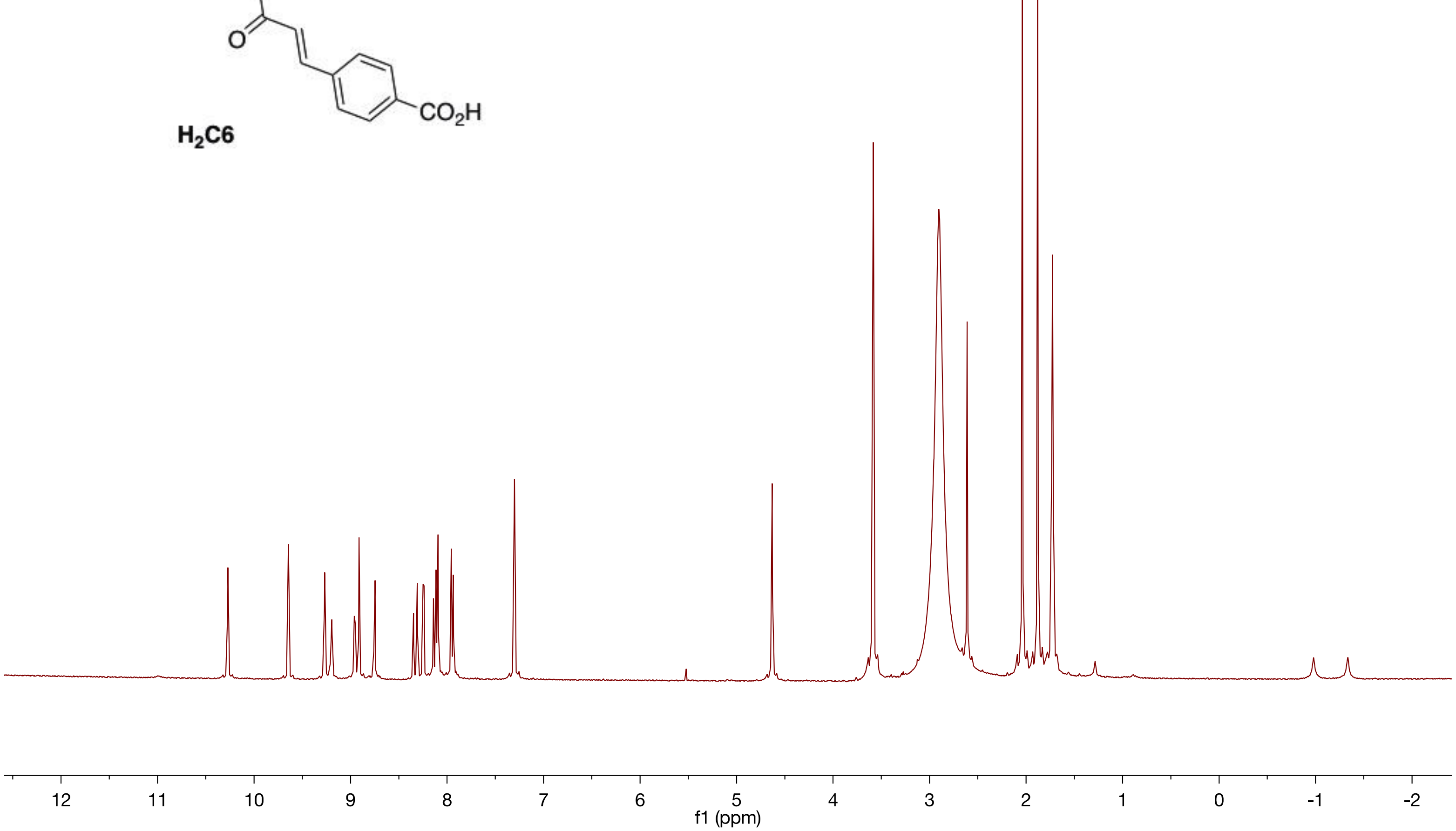

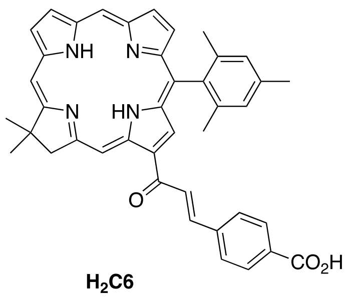

H_2C6

141760_Chalcone #459-501 RT: 2.75-3.01 AV: 43 NL: 3.22E6
T: FTMS + p ESI Full ms [200.00-1000.00]

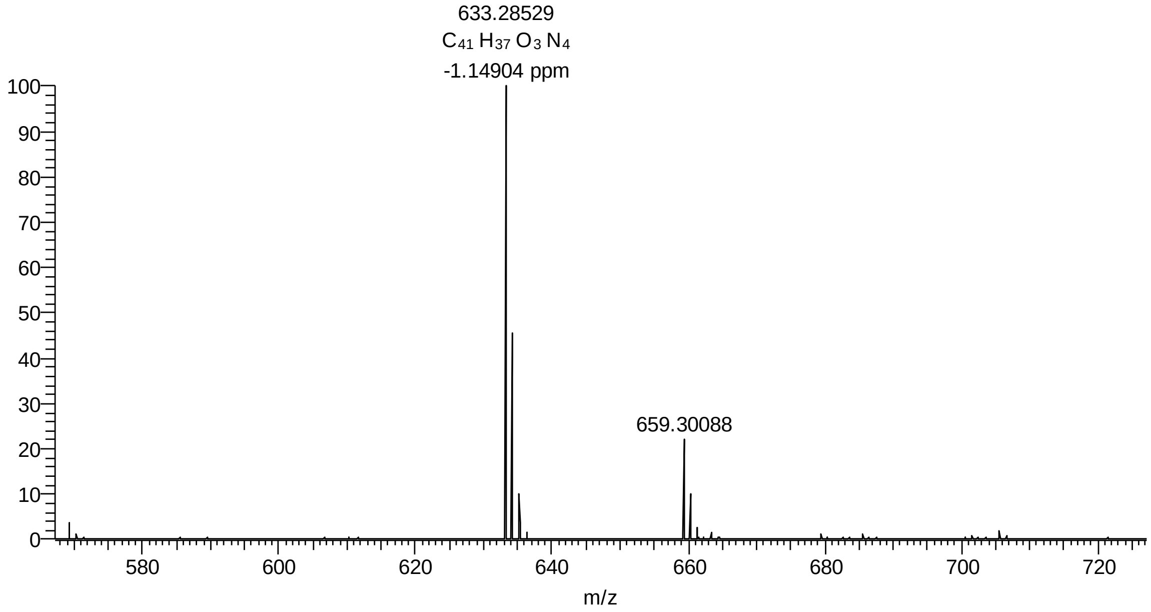

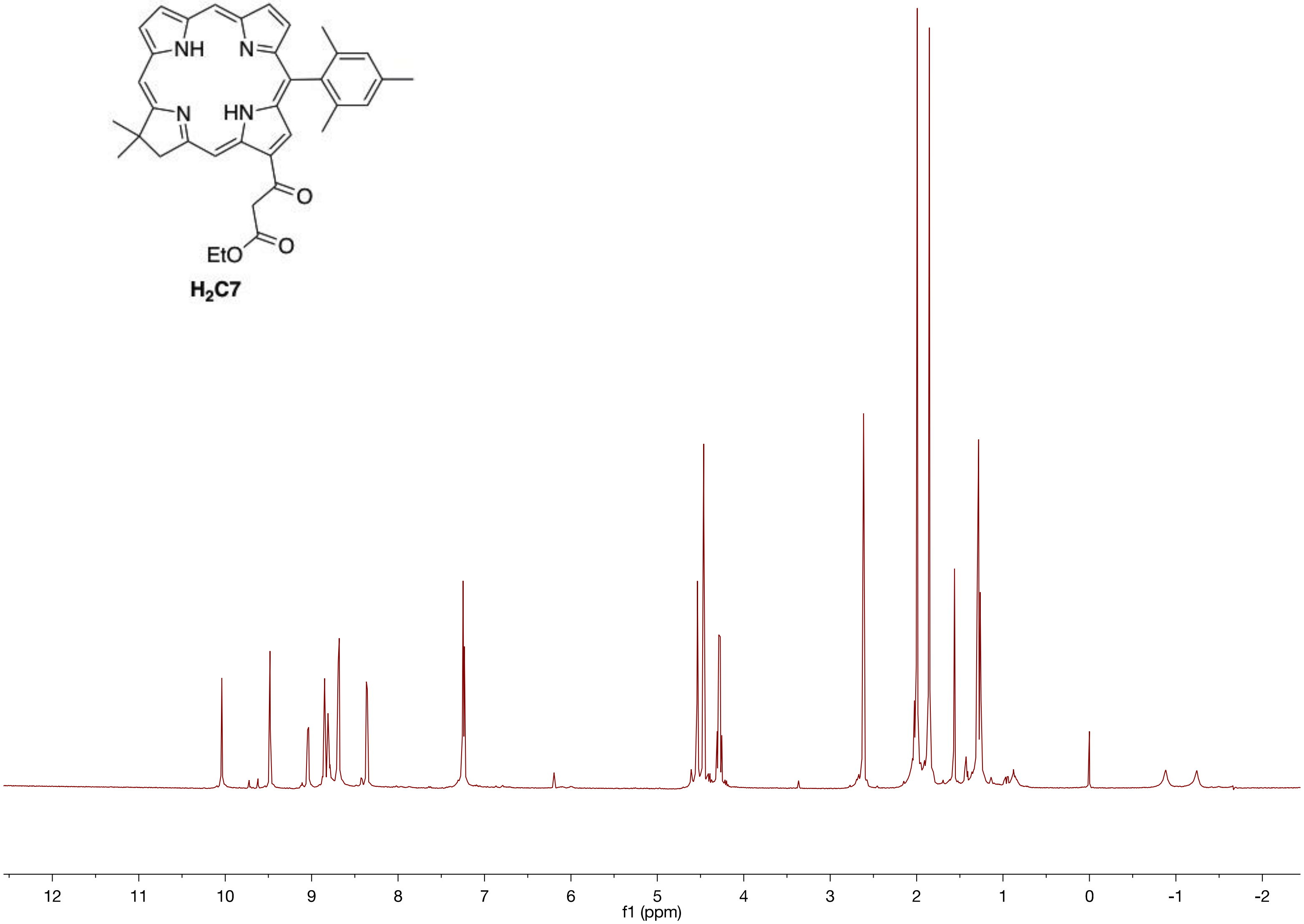
NH
N
N
HN
EtO
O
O
H2C7
12
11
10
9
8
7
6
5
4
3
2
1
0
-1
-2
f1 (ppm)

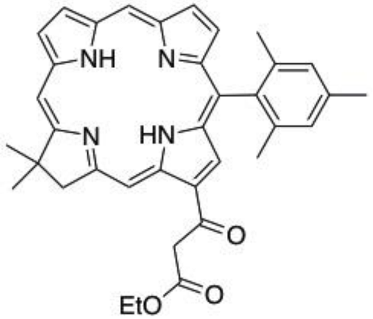

NH
N
N
HN
O
EtO
O

H_2C7

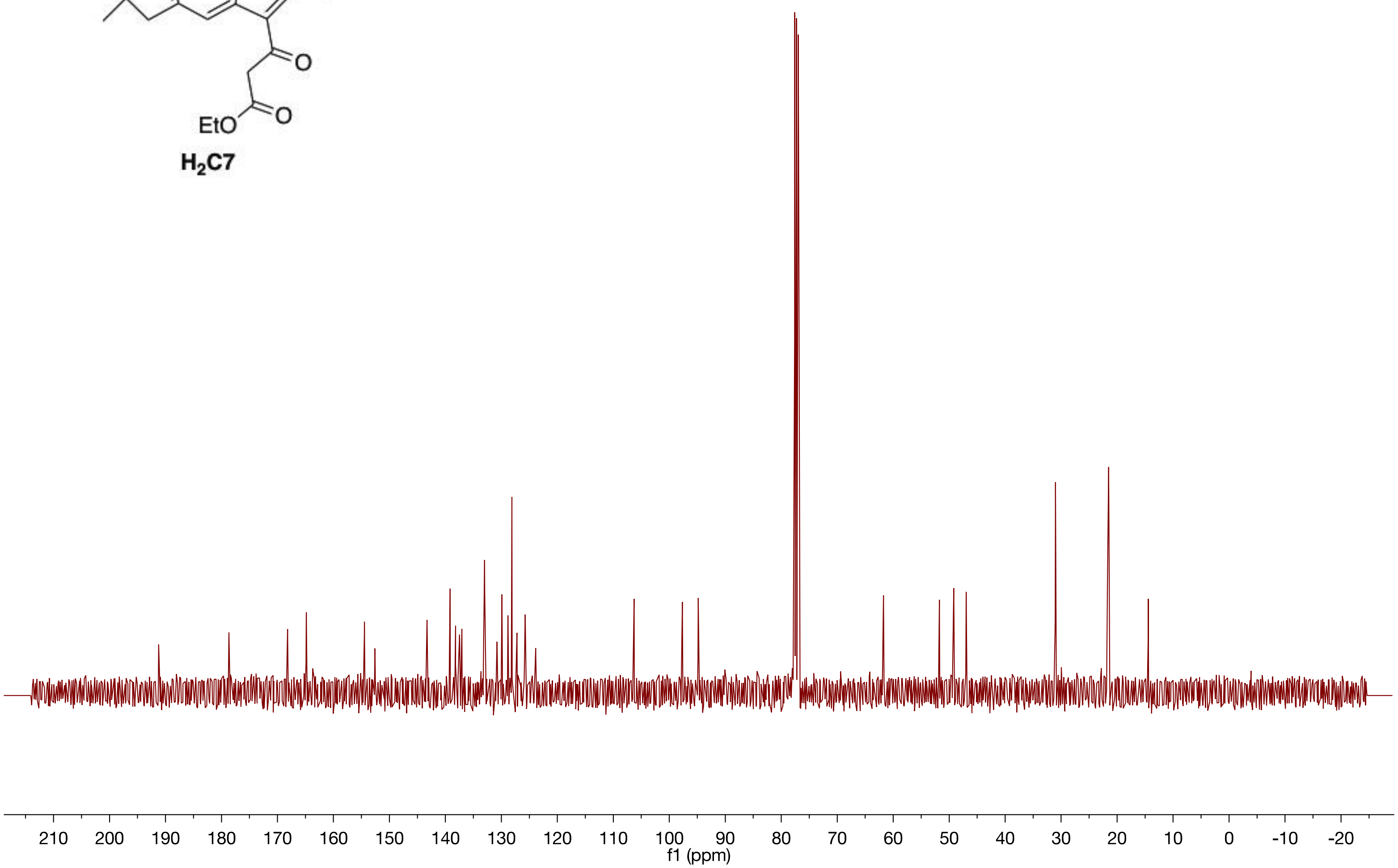

210
200
190
180
170
160
150
140
130
120
110
100
90
80
70
60
50
40
30
20
10
0
-10
-20
f1 (ppm)

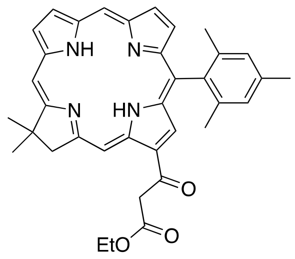
NH
N
N
HN
O
EtO
O

H_2C7

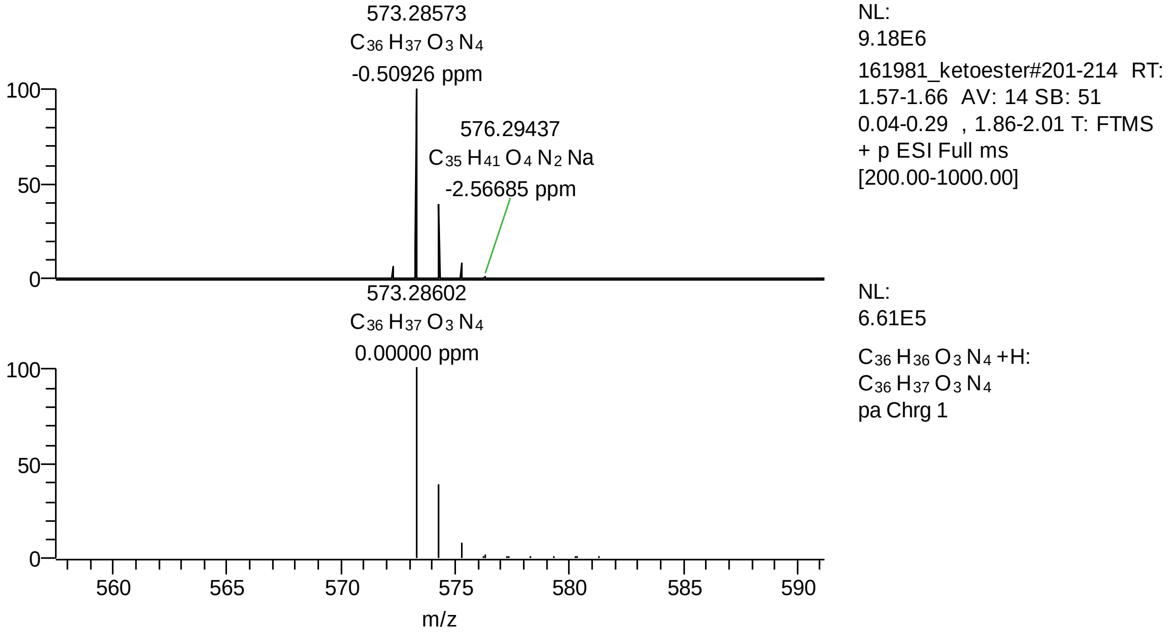
573.28573
C36 H37 O3 N4
-0.50926 ppm
576.29437
C35 H41 O4 N2 Na
-2.56685 ppm
NL:
9.18E6
161981_ketoester#201-214 RT: 1.57-1.66 AV: 14 SB: 51 0.04-0.29 , 1.86-2.01 T: FTMS + p ESI Full ms [200.00-1000.00]
573.28602
C36 H37 O3 N4
0.00000 ppm
NL:
6.61E5
C36 H36 O3 N4 +H:
C36 H37 O3 N4
pa Chrg 1
m/z

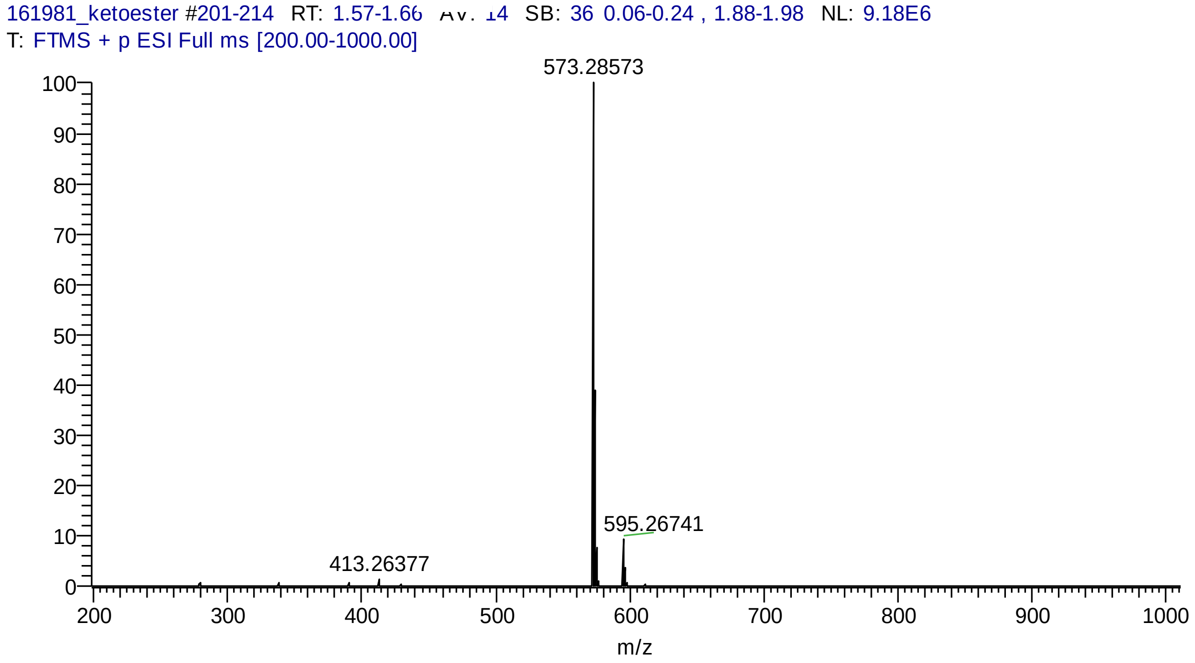
161981_ketoester #201-214 RT: 1.57-1.66 AV: 14 SB: 36 0.06-0.24 , 1.88-1.98 NL: 9.18E6
T: FTMS + p ESI Full ms [200.00-1000.00]
573.28573
595.26741
413.26377
m/z

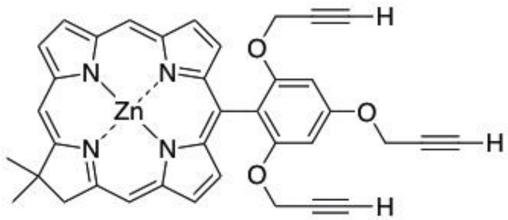

ZnC8

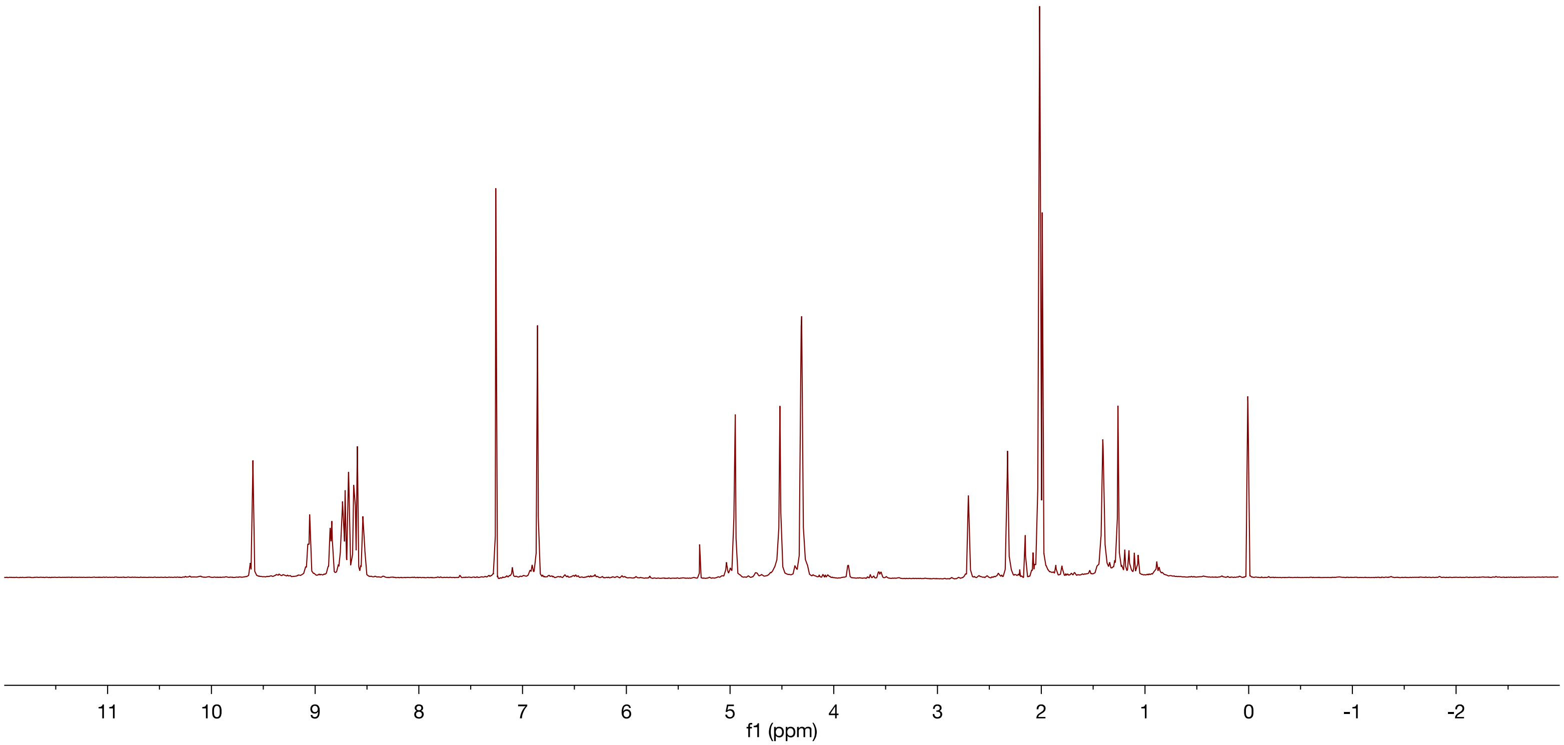

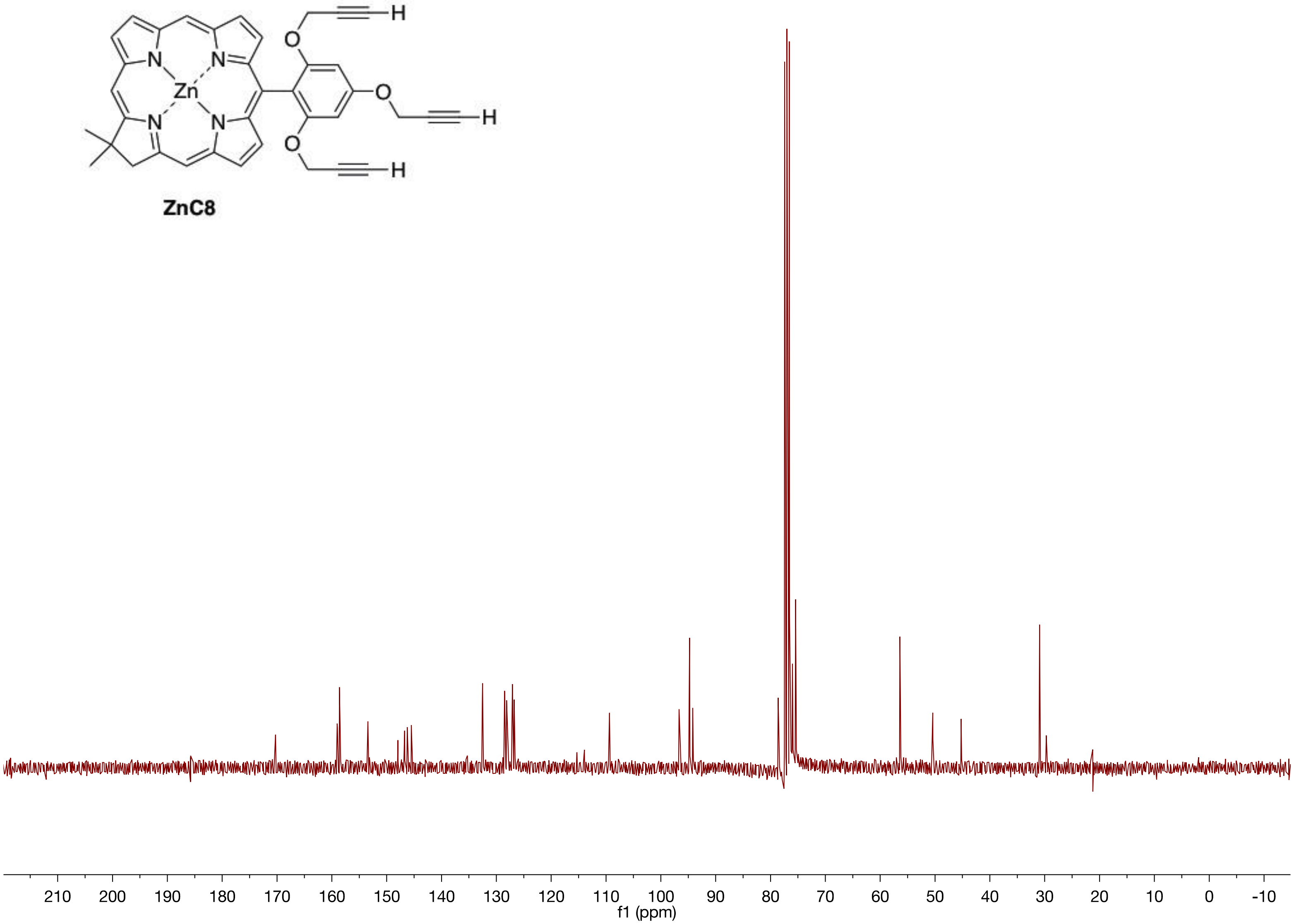
ZnC8
210 200 190 180 170 160 150 140 130 120 110 100 90 80 70 60 50 40 30 20 10 0 -10
f1 (ppm)

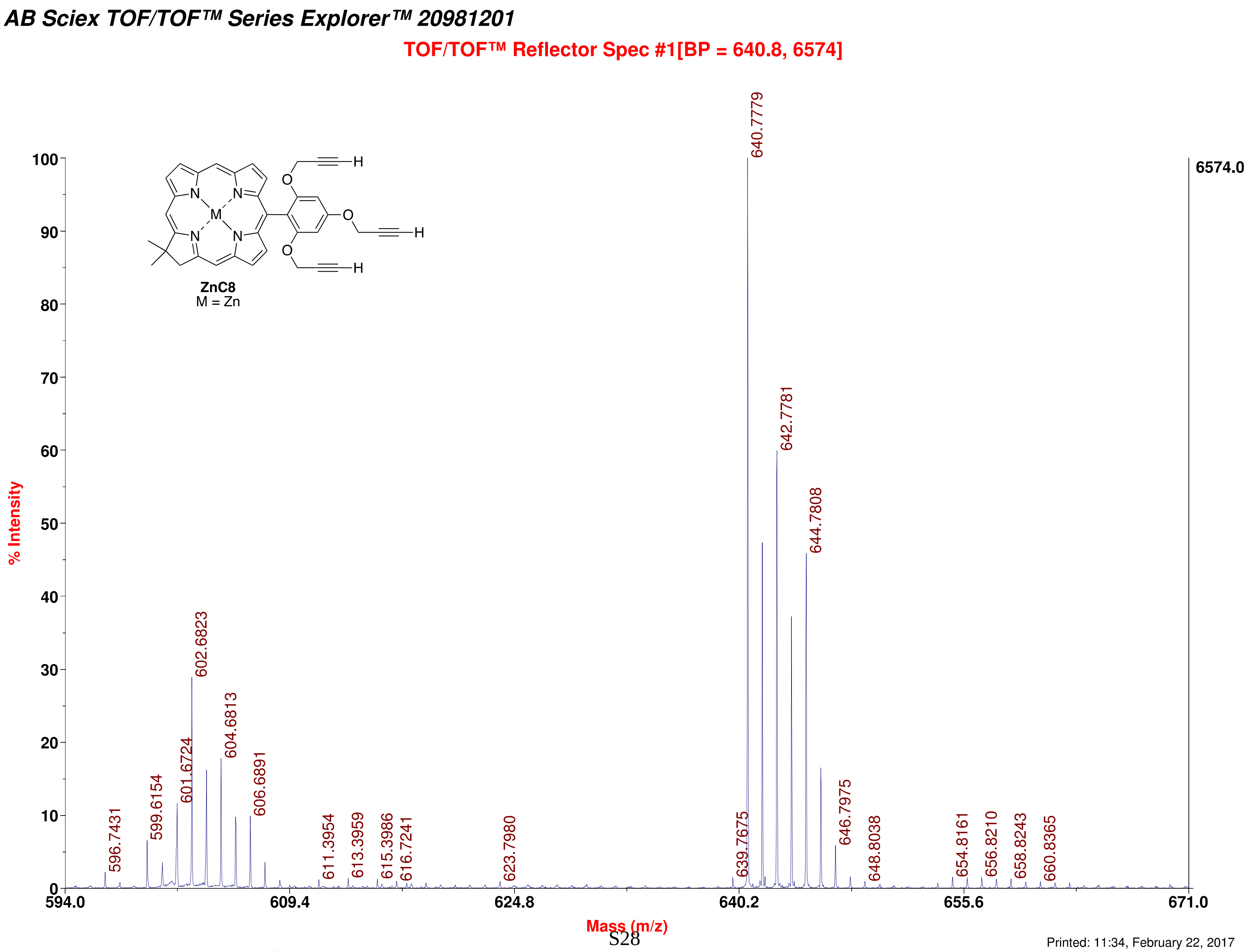

Printed: 11:34, February 22, 2017

F:\User Project 1\Lindsey\RLiu\2017\020817\Zn Chlorin.T2D

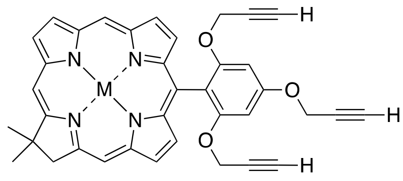

O
H
M
N

ZnC8
M = Zn

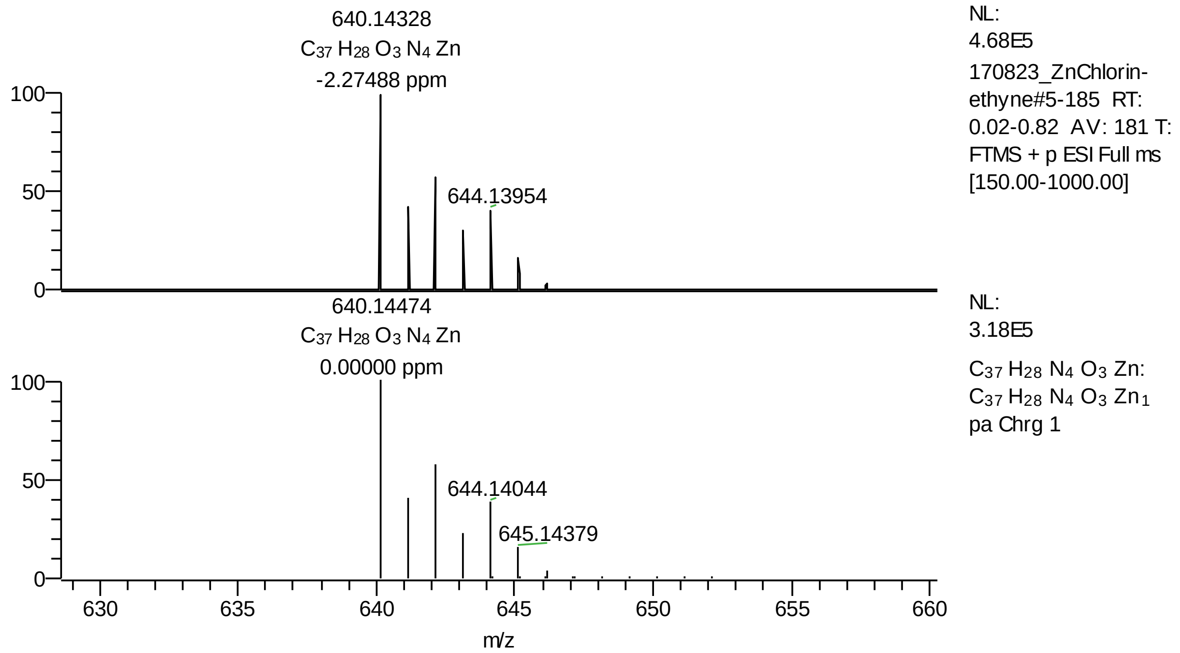

640.14328
C37 H28 O3 N4 Zn
-2.27488 ppm
644.13954
NL:
4.68E5
170823_ZnChlorin-ethyne#5-185 RT: 0.02-0.82 AV: 181 T: FTMS + p ESI Full ms [150.00-1000.00]
640.14474
C37 H28 O3 N4 Zn
0.00000 ppm
644.14044
645.14379
NL:
3.18E5
C37 H28 N4 O3 Zn: C37 H28 N4 O3 Zn1 pa Chrg 1
m/z

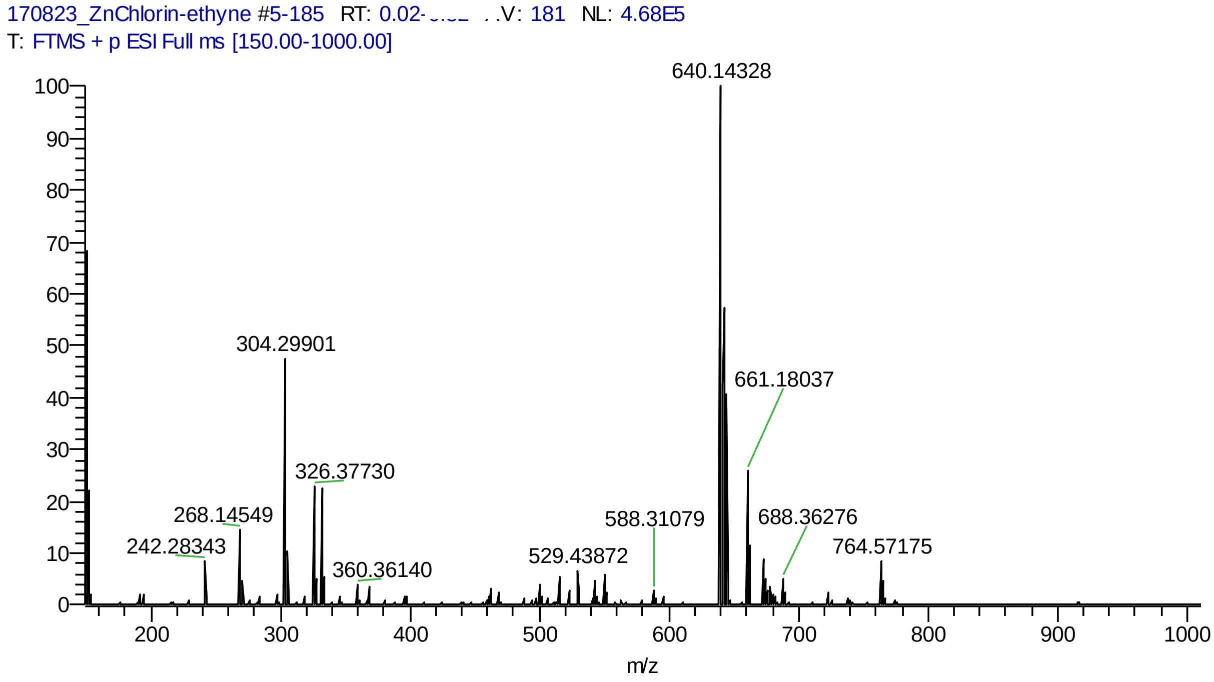

170823_ZnChlorin-ethyne #5-185 RT: 0.02- V: 181 NL: 4.68E5
T: FTMS + p ESI Full ms [150.00-1000.00]
640.14328
304.29901
661.18037
326.37730
268.14549
242.28343
360.36140
588.31079
688.36276
529.43872
764.57175
m/z

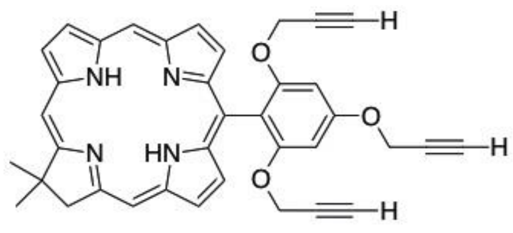
NH
N
N
HN
O
O
O
H
H
H

H_2C8

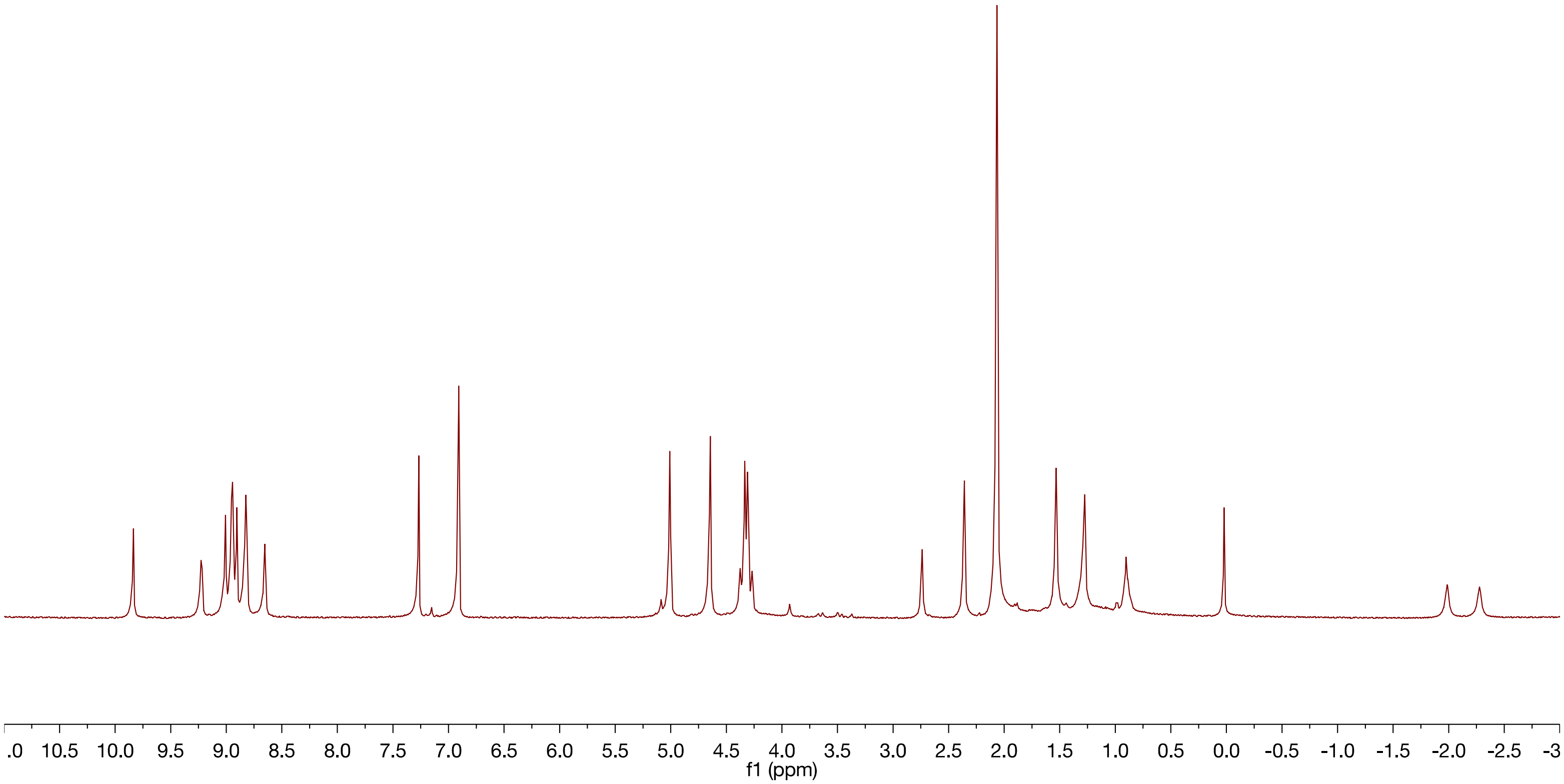
.0 10.5 10.0 9.5 9.0 8.5 8.0 7.5 7.0 6.5 6.0 5.5 5.0 4.5 4.0 3.5 3.0 2.5 2.0 1.5 1.0 0.5 0.0 -0.5 -1.0 -1.5 -2.0 -2.5 -3
f1 (ppm)

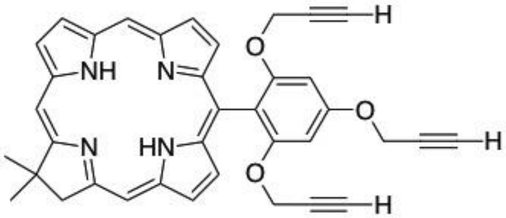
NH
N
N
HN
O
O
O
H
H
H

H_2C8

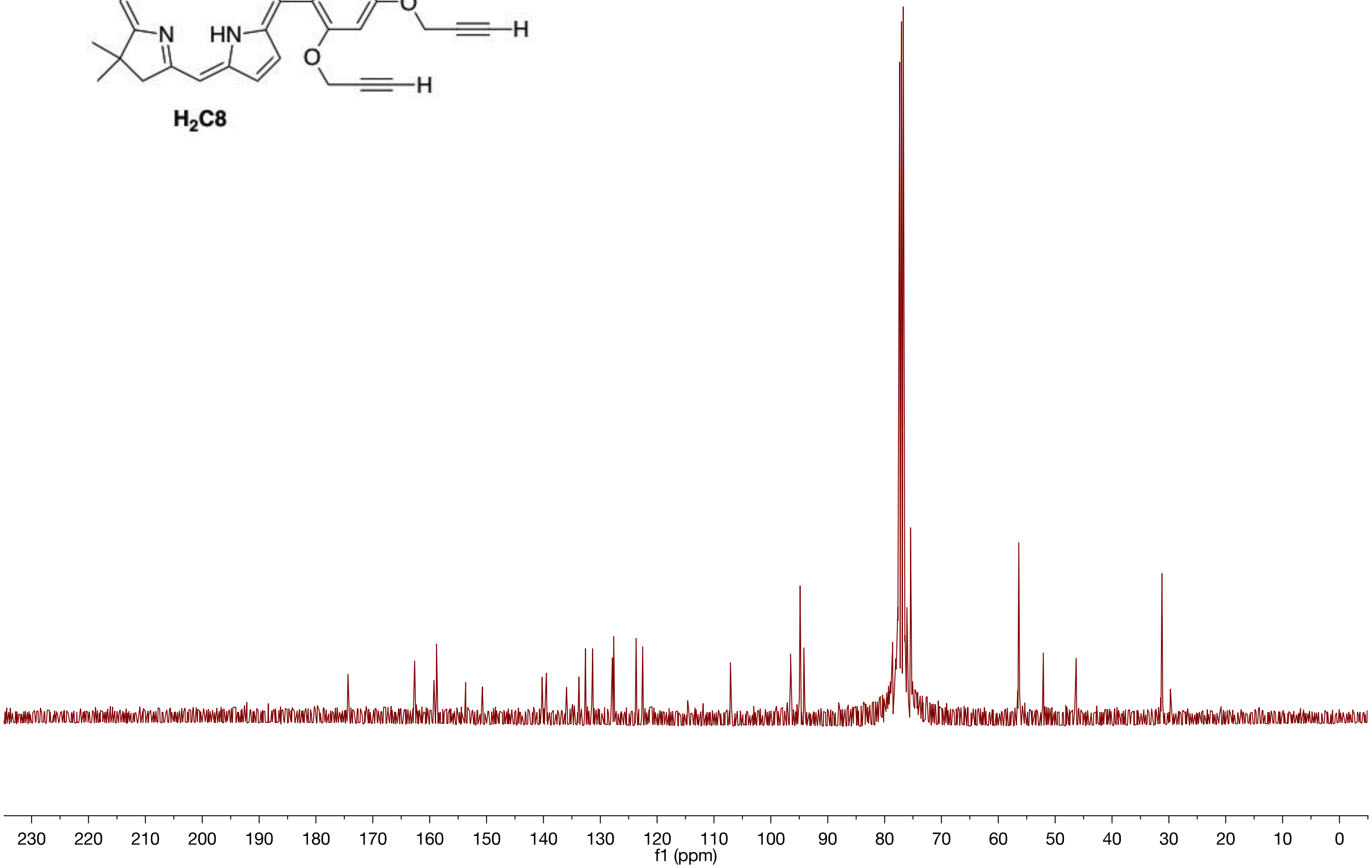
230 220 210 200 190 180 170 160 150 140 130 120 110 100 90 80 70 60 50 40 30 20 10 0
f1 (ppm)

AB Sciex TOF/TOF™ Series Explorer™ 20981201

TOF/TOF™ Reflector Spec #1[BP = 579.2, 21493]

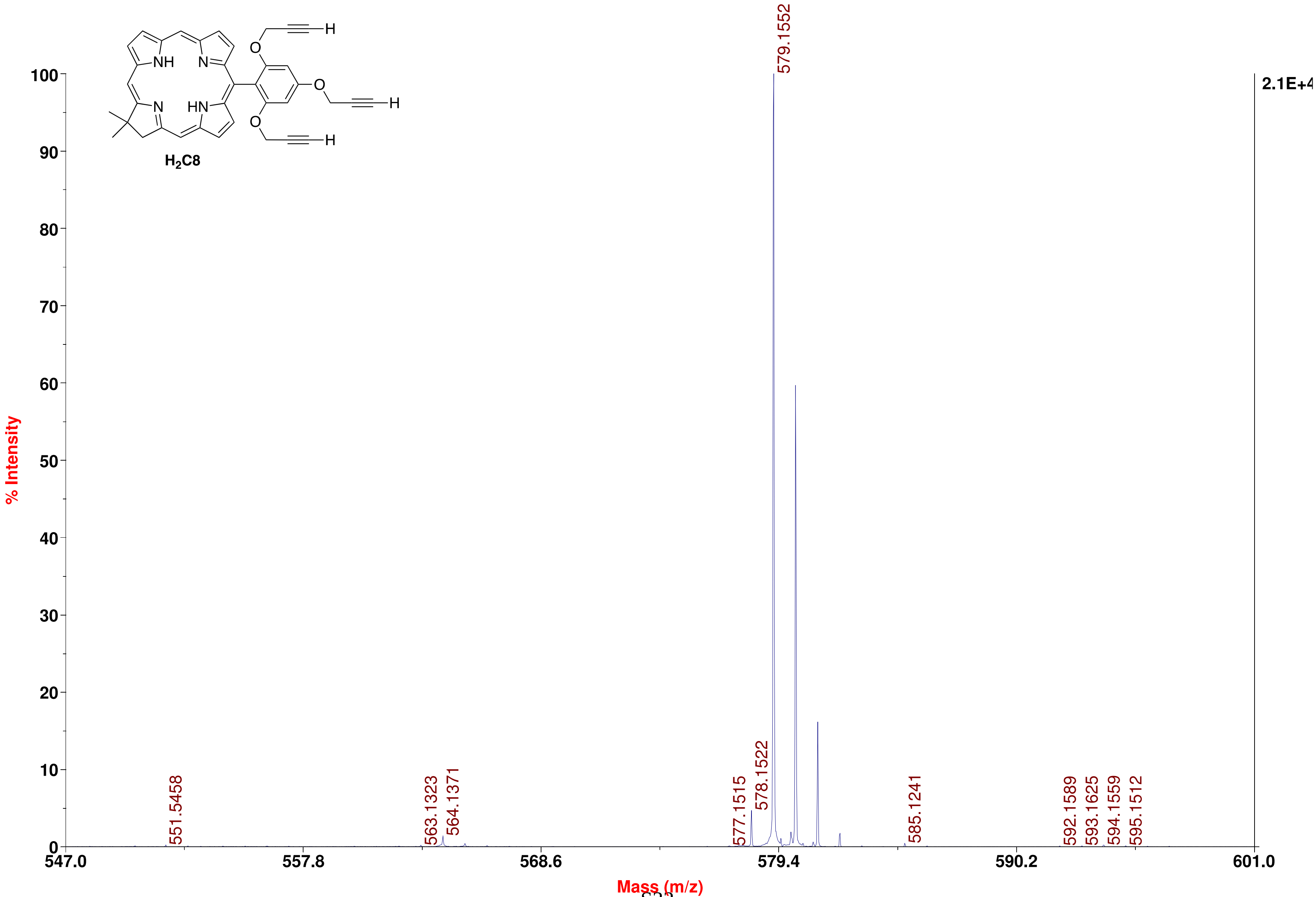

Printed: 11:35, February 22, 2017

F:\User Project 1\Lindsey\RLiu\2017\022217\FbC-ethyne.T2D

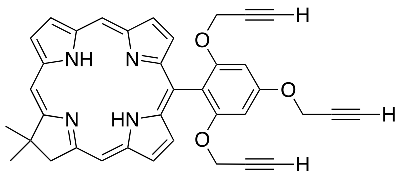
NH
N
N
HN
O
O
O
H
H
H

H_2C8

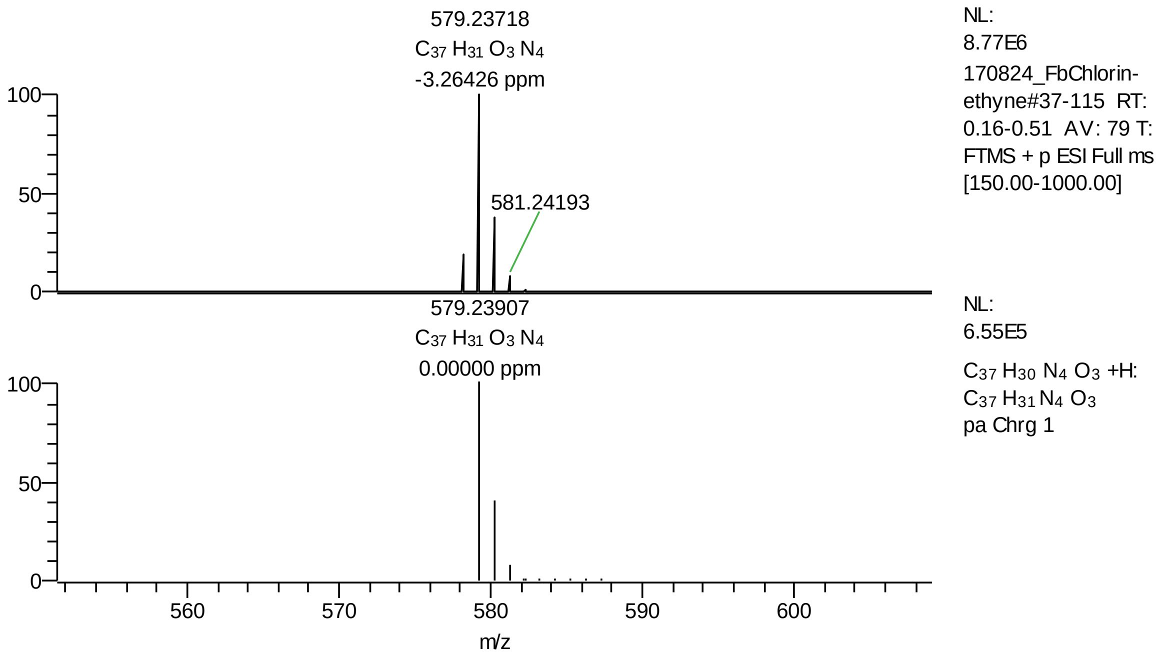
579.23718
C37 H31 O3 N4
-3.26426 ppm
581.24193
NL:
8.77E6
170824_FbChlorin-ethyne#37-115 RT: 0.16-0.51 AV: 79 T: FTMS + p ESI Full ms [150.00-1000.00]
579.23907
C37 H31 O3 N4
0.00000 ppm
NL:
6.55E5
C37 H30 N4 O3 +H: C37 H31 N4 O3
pa Chrg 1
560
570
580
590
600
m/z

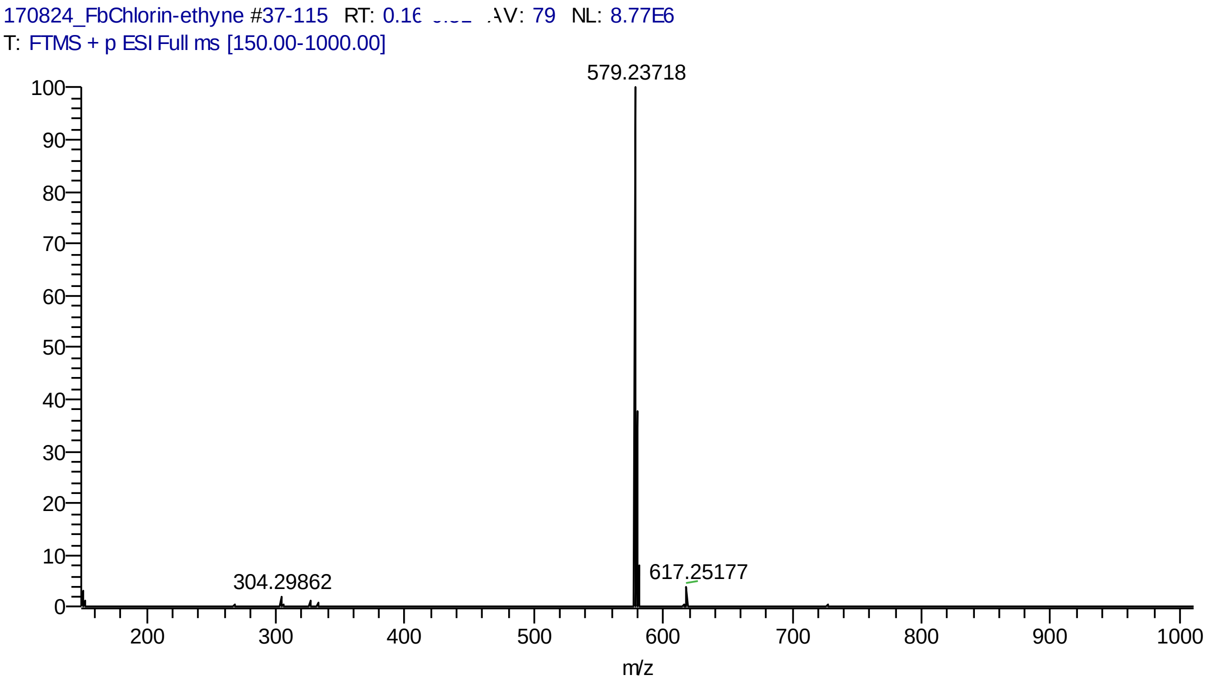
170824_FbChlorin-ethyne #37-115 RT: 0.16 AV: 79 NL: 8.77E6
T: FTMS + p ESI Full ms [150.00-1000.00]
579.23718
304.29862
617.25177
m/z

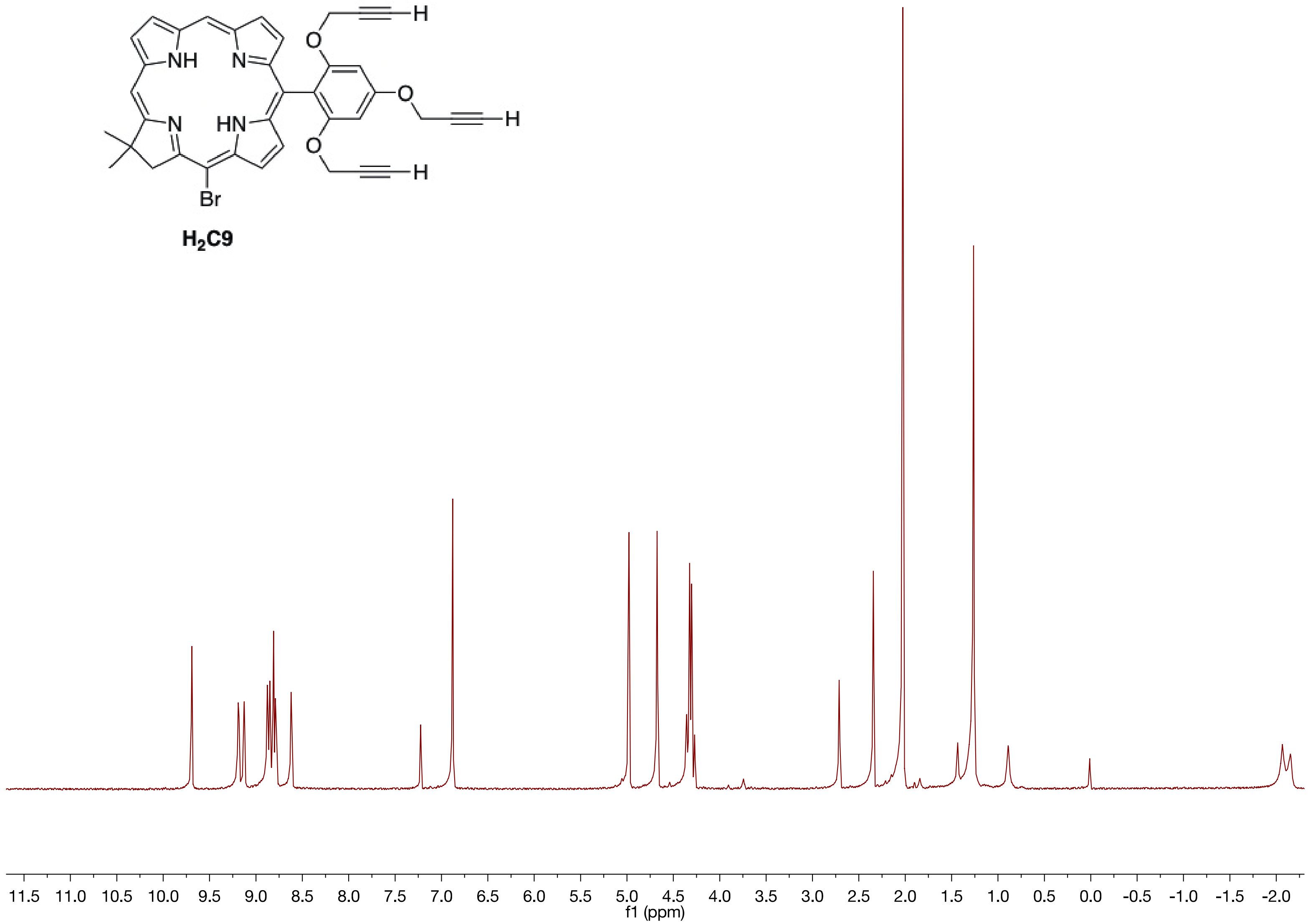
NH
N
N
HN
Br
O
O
O
H
H
H
H2C9
11.5 11.0 10.5 10.0 9.5 9.0 8.5 8.0 7.5 7.0 6.5 6.0 5.5 5.0 4.5 4.0 3.5 3.0 2.5 2.0 1.5 1.0 0.5 0.0 -0.5 -1.0 -1.5 -2.0
f1 (ppm)

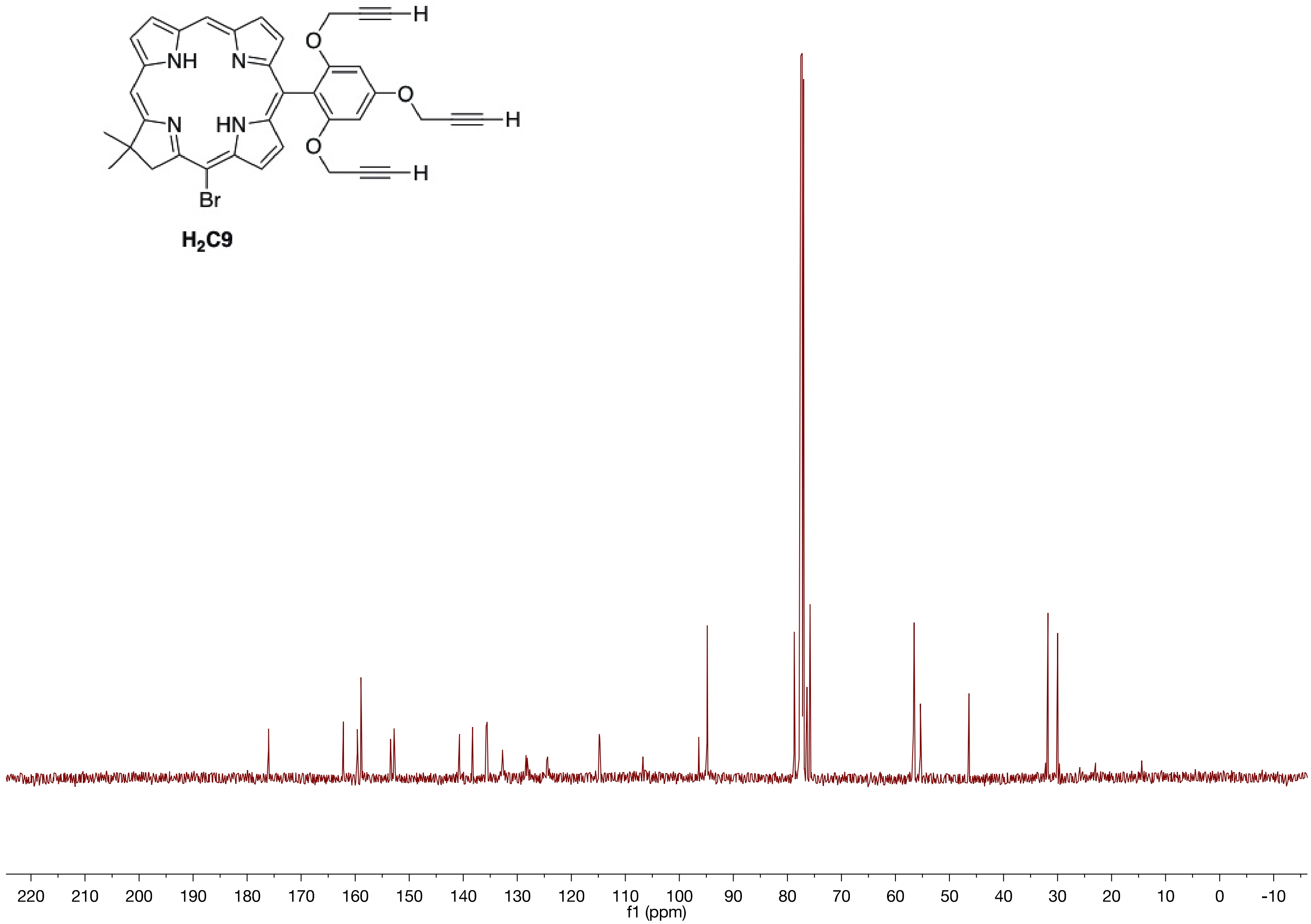

H_2C9
220 210 200 190 180 170 160 150 140 130 120 110 100 90 80 70 60 50 40 30 20 10 0 -10
f1 (ppm)

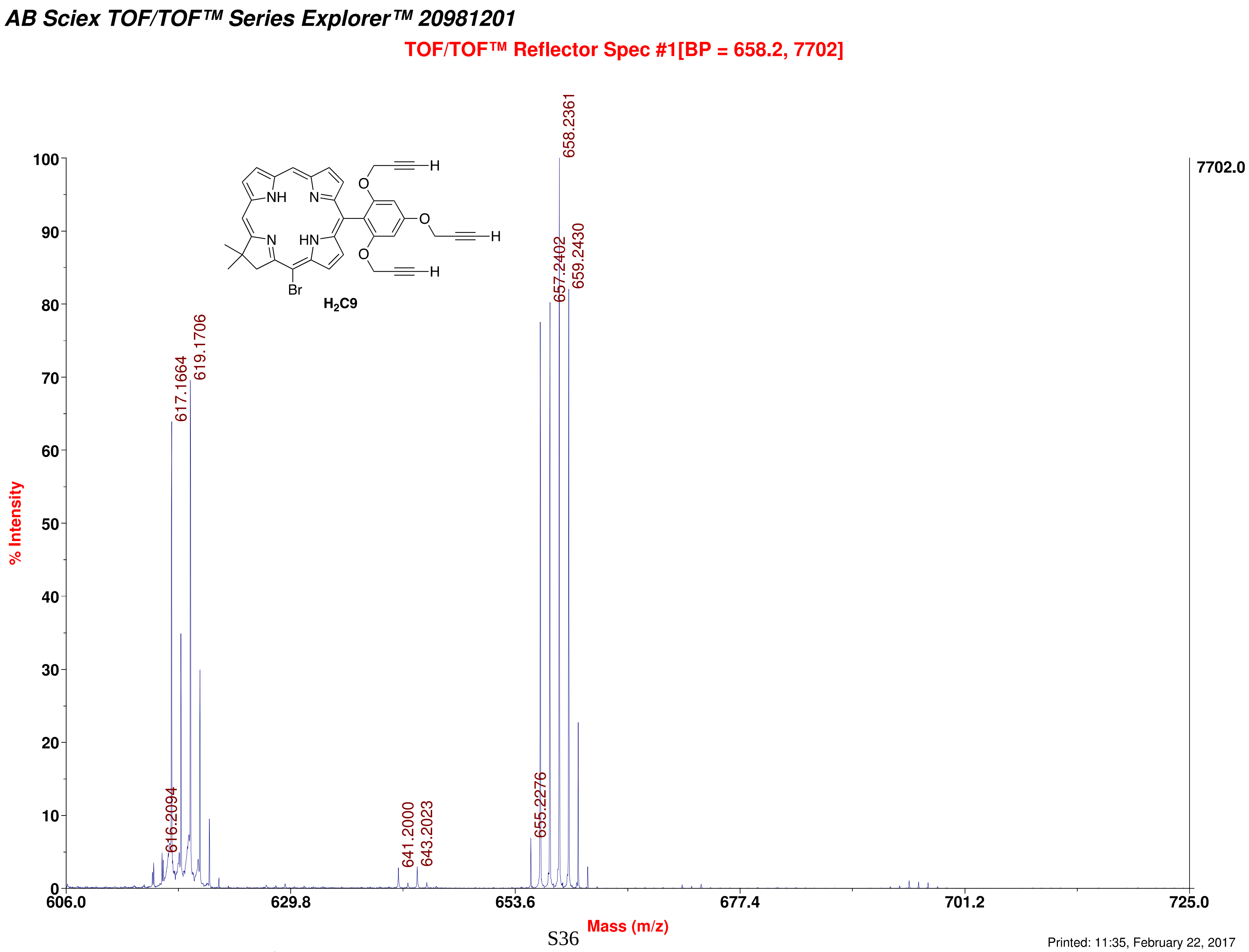

Printed: 11:35, February 22, 2017

F:\User Project 1\Lindsey\RLiu\2017\021317\FbC-15Br.T2D

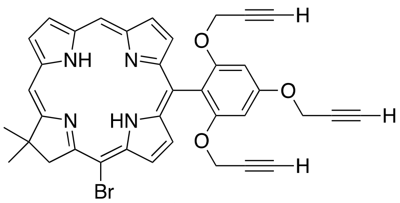
NH
N
N
HN
O
O
O
H
H
H
Br

H_2C9

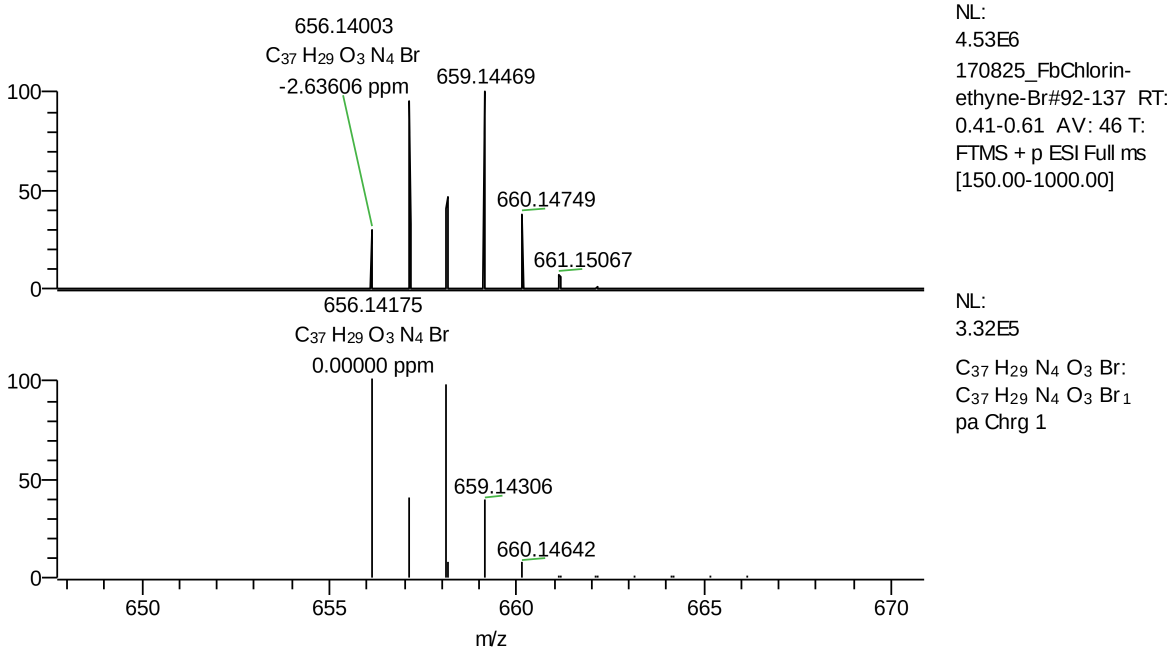
656.14003
C37 H29 O3 N4 Br
-2.63606 ppm
659.14469
660.14749
661.15067
NL:
4.53E6
170825_FbChlorin-ethyne-Br#92-137 RT: 0.41-0.61 AV: 46 T: FTMS + p ESI Full ms [150.00-1000.00]
656.14175
C37 H29 O3 N4 Br
0.00000 ppm
659.14306
660.14642
NL:
3.32E5
C37 H29 N4 O3 Br:
C37 H29 N4 O3 Br1
pa Chrg 1
m/z

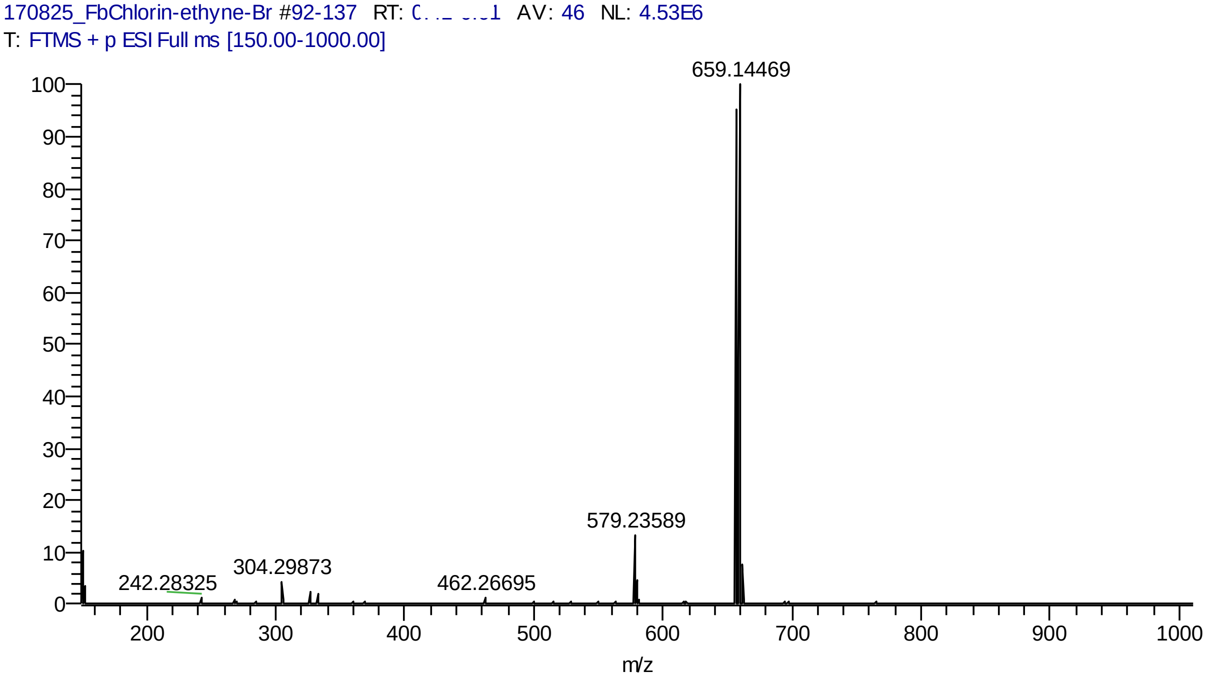
170825_FbChlorin-ethyne-Br #92-137 RT: AV: 46 NL: 4.53E6
T: FTMS + p ESI Full ms [150.00-1000.00]
659.14469
579.23589
242.28325
304.29873
462.26695
m/z

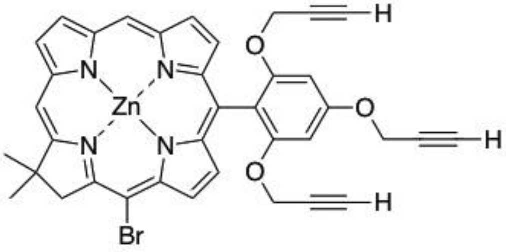

N
Zn
Br
O
H

ZnC9

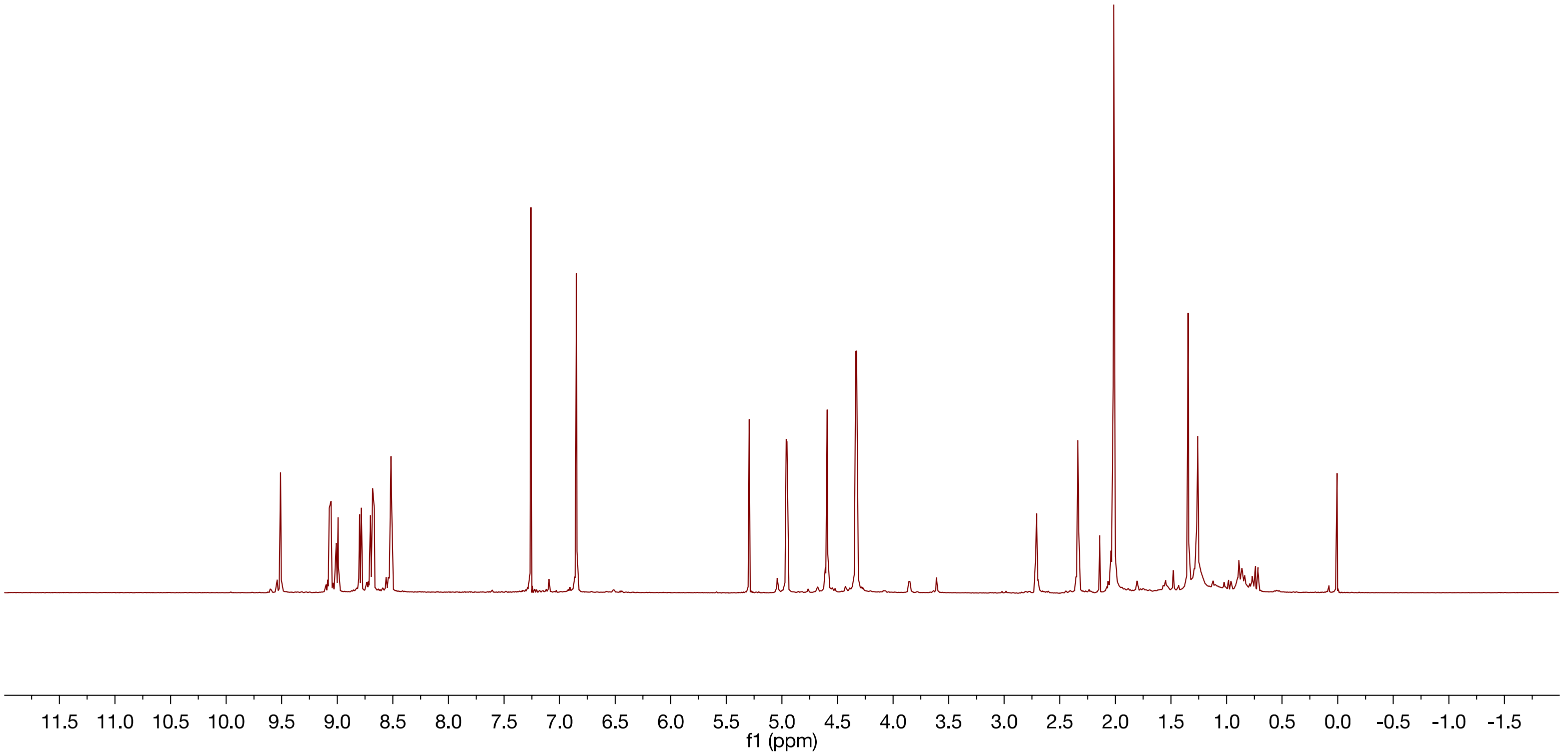

11.5 11.0 10.5 10.0 9.5 9.0 8.5 8.0 7.5 7.0 6.5 6.0 5.5 5.0 4.5 4.0 3.5 3.0 2.5 2.0 1.5 1.0 0.5 0.0 -0.5 -1.0 -1.5
f1 (ppm)

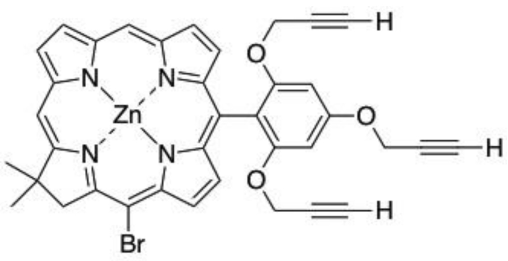
N
N
N
N
Zn
O
O
O
H
H
H
Br

ZnC9

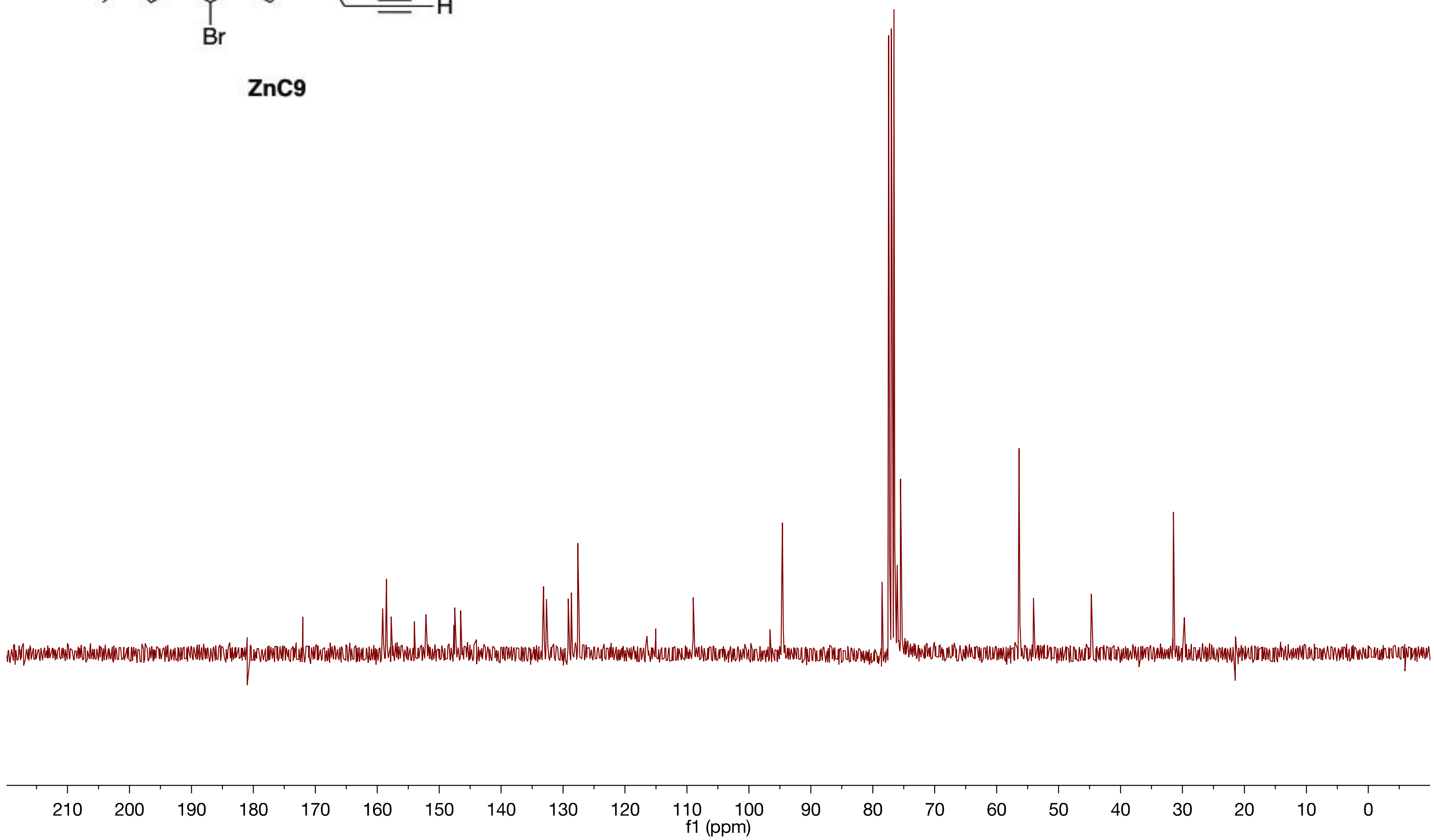
210
200
190
180
170
160
150
140
130
120
110
100
90
80
70
60
50
40
30
20
10
0
f1 (ppm)

AB Sciex TOF/TOF™ Series Explorer™ 20981201

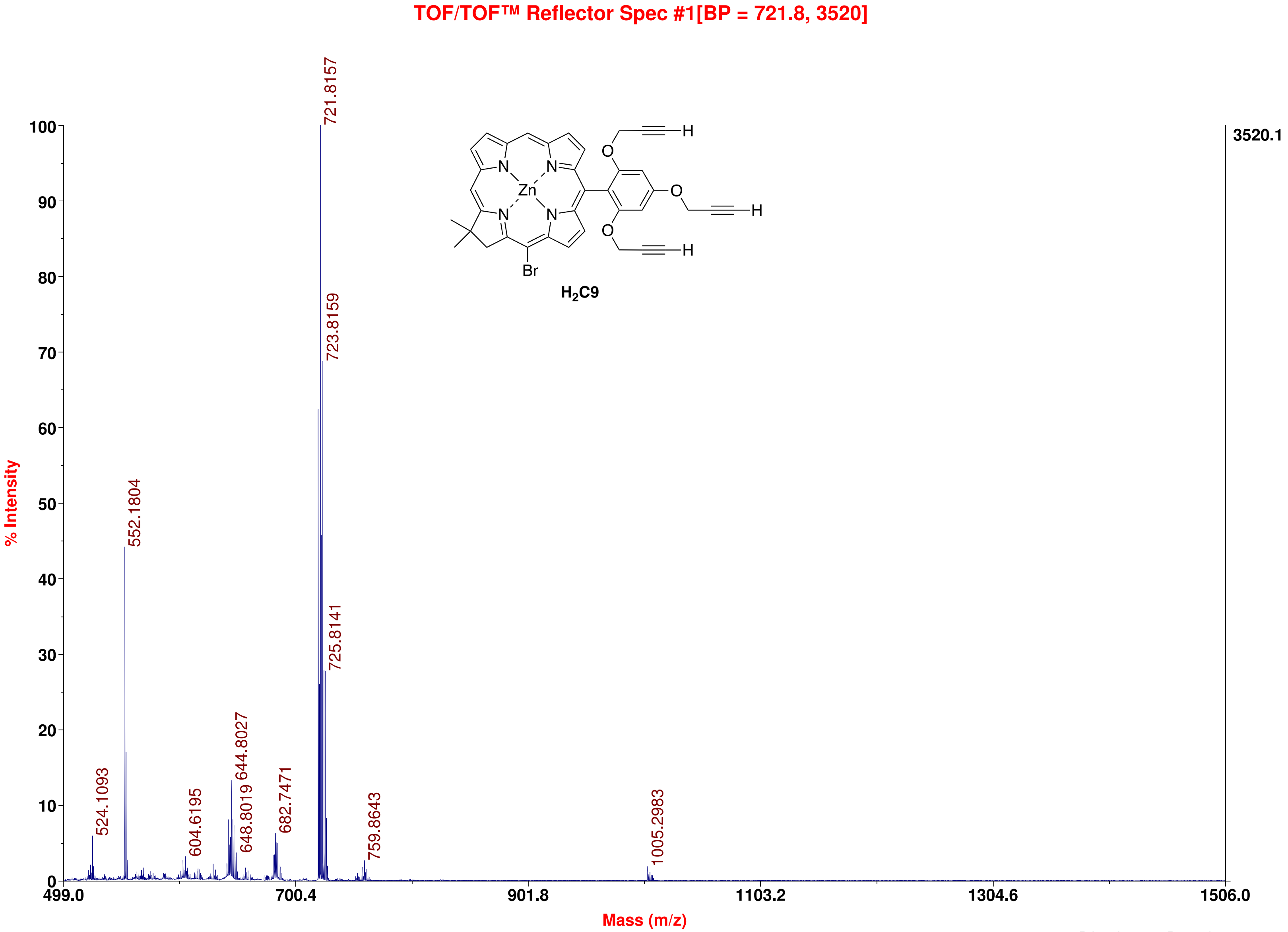

Printed: 16:03, December 06, 2017

F:\User Project 1\Lindsey\Gongfang\2017\030917\Rui.T2D

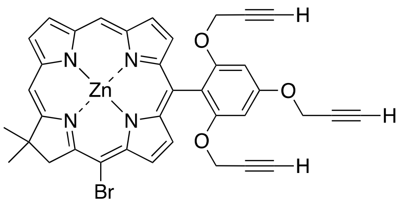

ZnC9

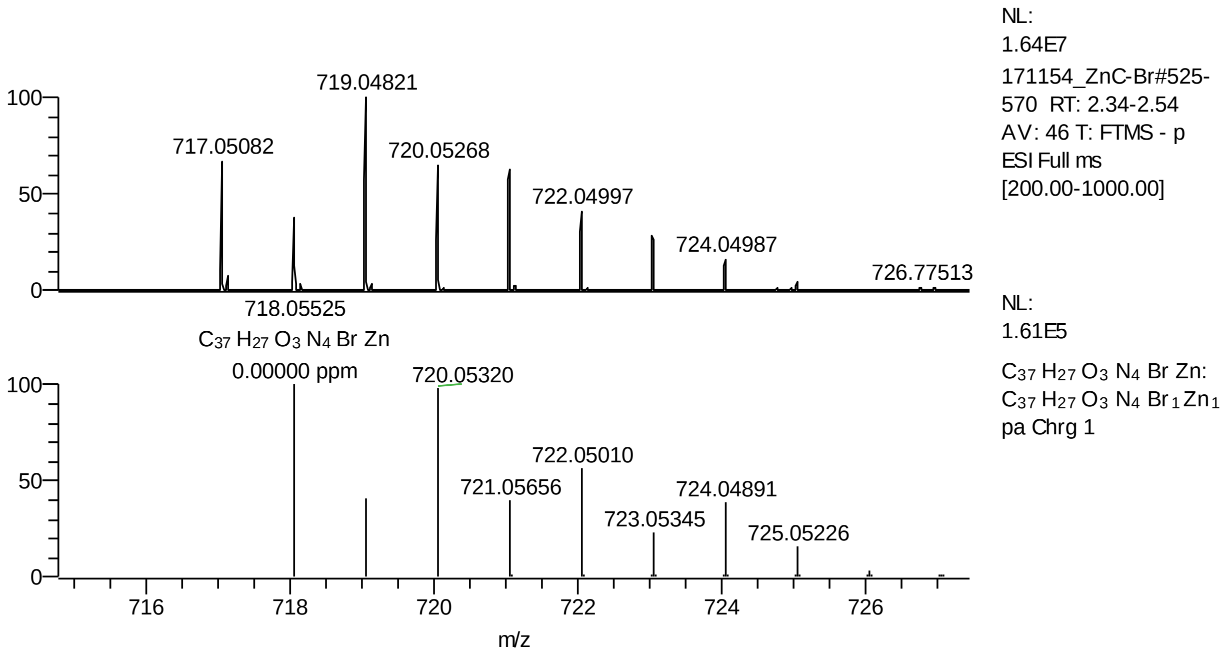

NL:
1.64E7
171154_ZnC-Br#525-570 RT: 2.34-2.54
AV: 46 T: FTMS - p
ESI Full ms
[200.00-1000.00]
719.04821
717.05082
720.05268
722.04997
724.04987
726.77513
718.05525
C37 H27 O3 N4 Br Zn
0.00000 ppm
NL:
1.61E5
C37 H27 O3 N4 Br Zn:
C37 H27 O3 N4 Br1 Zn1
pa Chrg 1
720.05320
722.05010
721.05656
724.04891
723.05345
725.05226
m/z

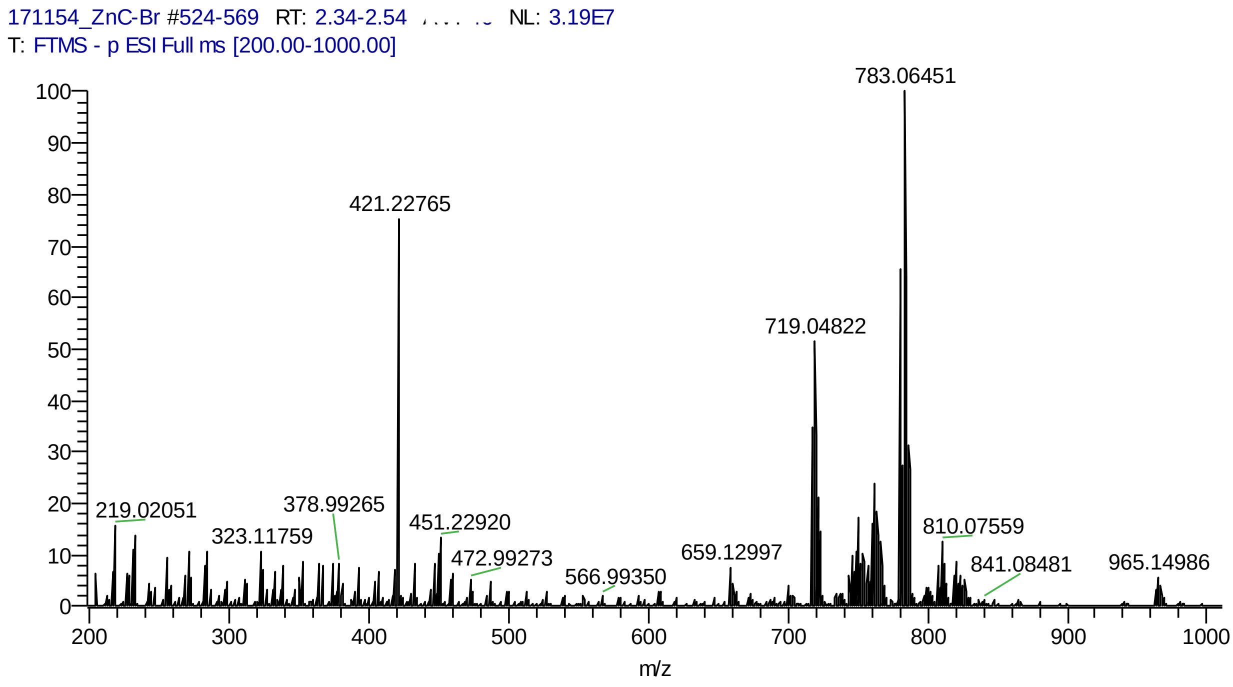

171154_ZnC-Br #524-569 RT: 2.34-2.54
NL: 3.19E7
T: FTMS - p ESI Full ms [200.00-1000.00]
783.06451
421.22765
719.04822
219.02051
378.99265
323.11759
451.22920
472.99273
566.99350
659.12997
810.07559
841.08481
965.14986
m/z

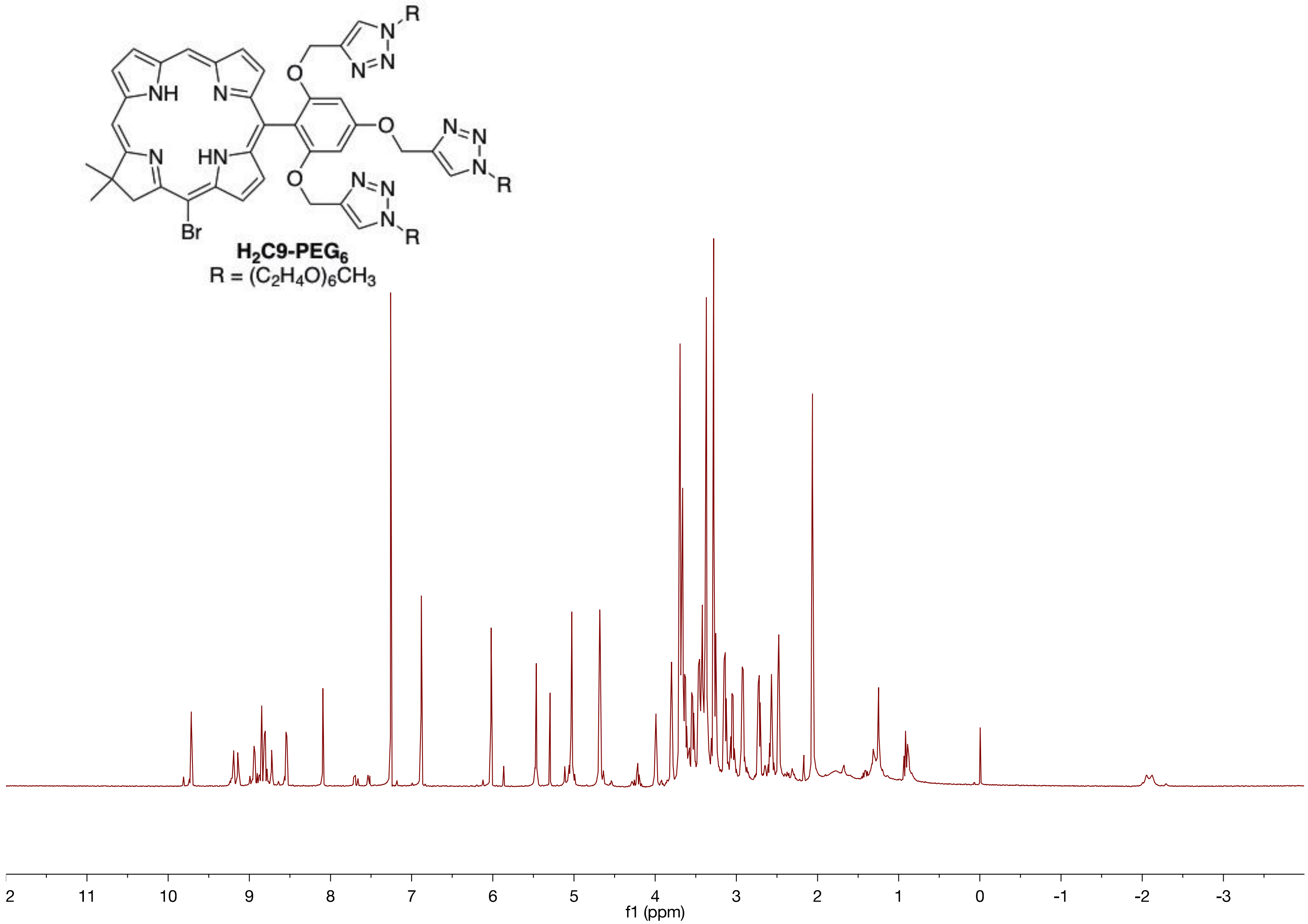
H2C9-PEG6
R = (C2H4O)6CH3
Br
NH
HN
f1 (ppm)

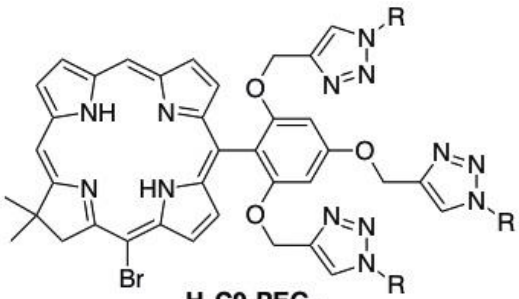

H_2C9-PEG_6
$R = (C_2H_4O)_6CH_3$

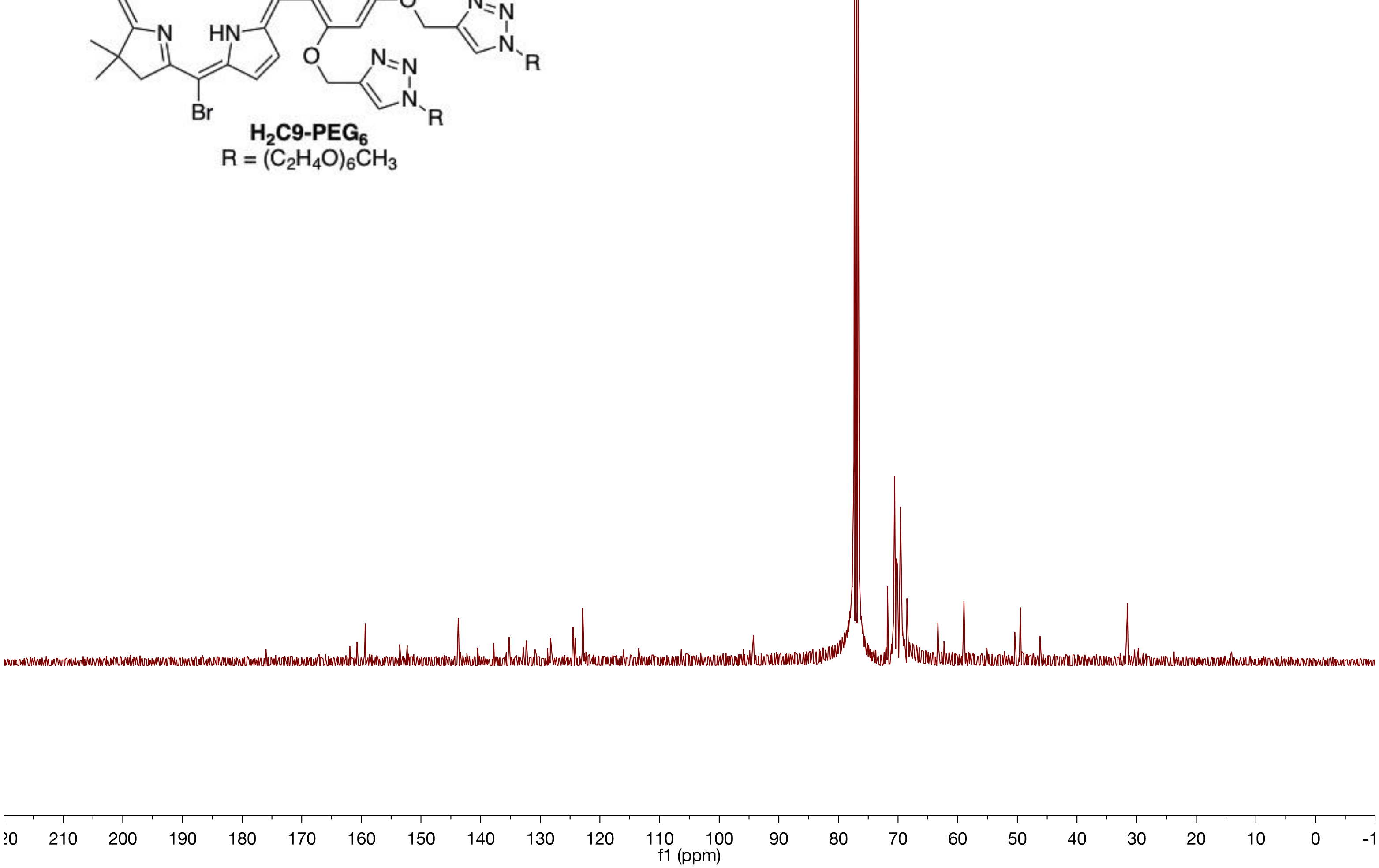

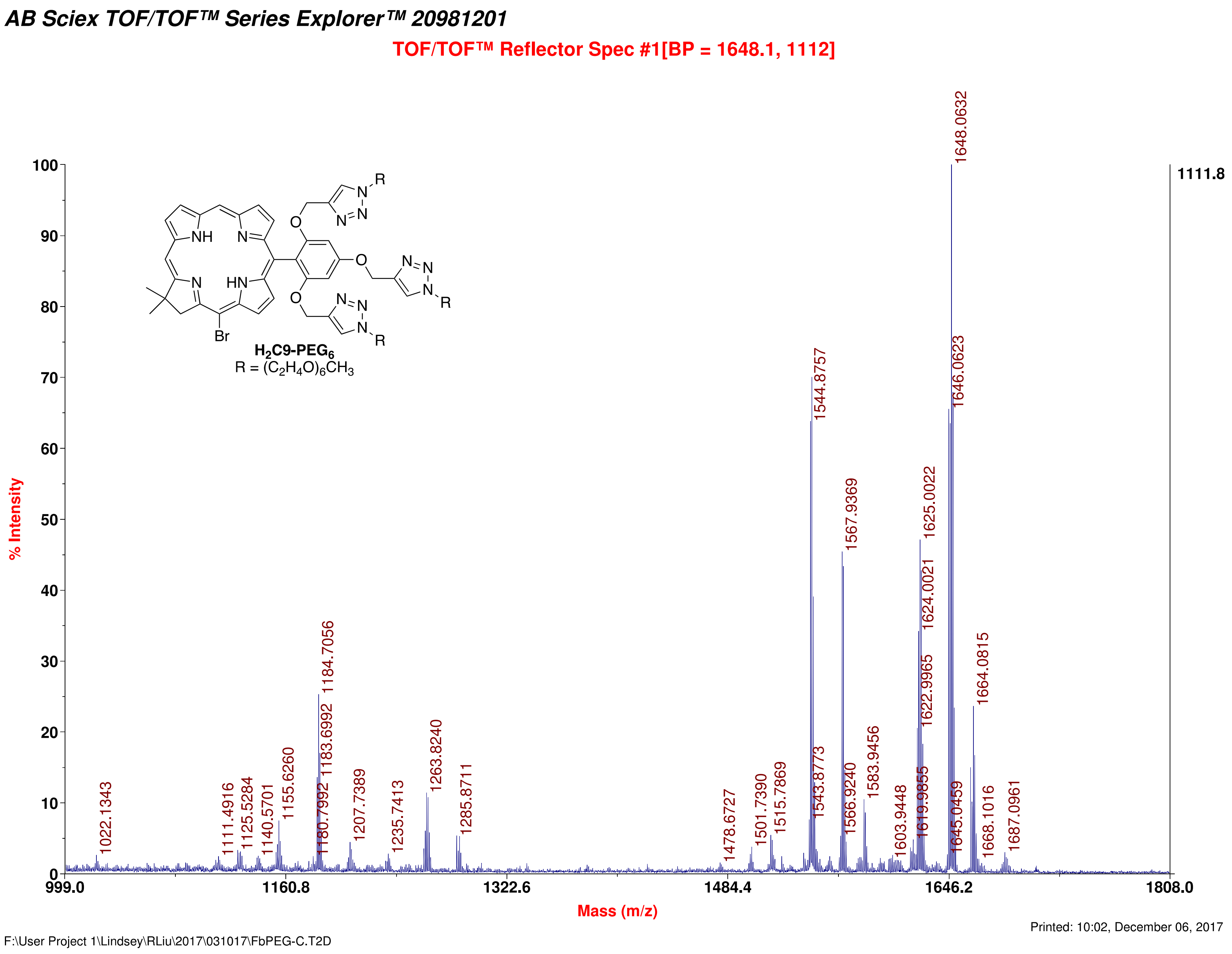
AB Sciex TOF/TOF™ Series Explorer™ 20981201
TOF/TOF™ Reflector Spec #1[BP = 1648.1, 1112]
H2C9-PEG6
R = (C2H4O)6CH3
Br
NH
N
HN
O
N
R
1648.0632
1646.0623
1544.8757
1567.9369
1625.0022
1624.0021
1664.0815
1622.9965
1583.9456
1184.7056
1183.6992
1263.8240
1543.8773
1566.9240
1603.9448
1619.9855
1645.0459
1668.1016
1687.0961
1478.6727
1501.7390
1515.7869
1285.8711
1235.7413
1207.7389
1180.7992
1155.6260
1140.5701
1125.5284
1111.4916
1022.1343
1111.8
% Intensity
Mass (m/z)
999.0
1160.8
1322.6
1484.4
1646.2
1808.0
Printed: 10:02, December 06, 2017
F:\User Project 1\Lindsey\RLiu\2017\031017\FbPEG-C.T2D

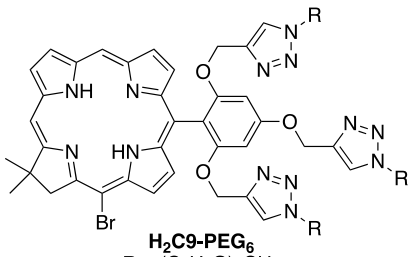

H_2C9-PEG$_6$
$R = (C_2H_4O)_6CH_3$

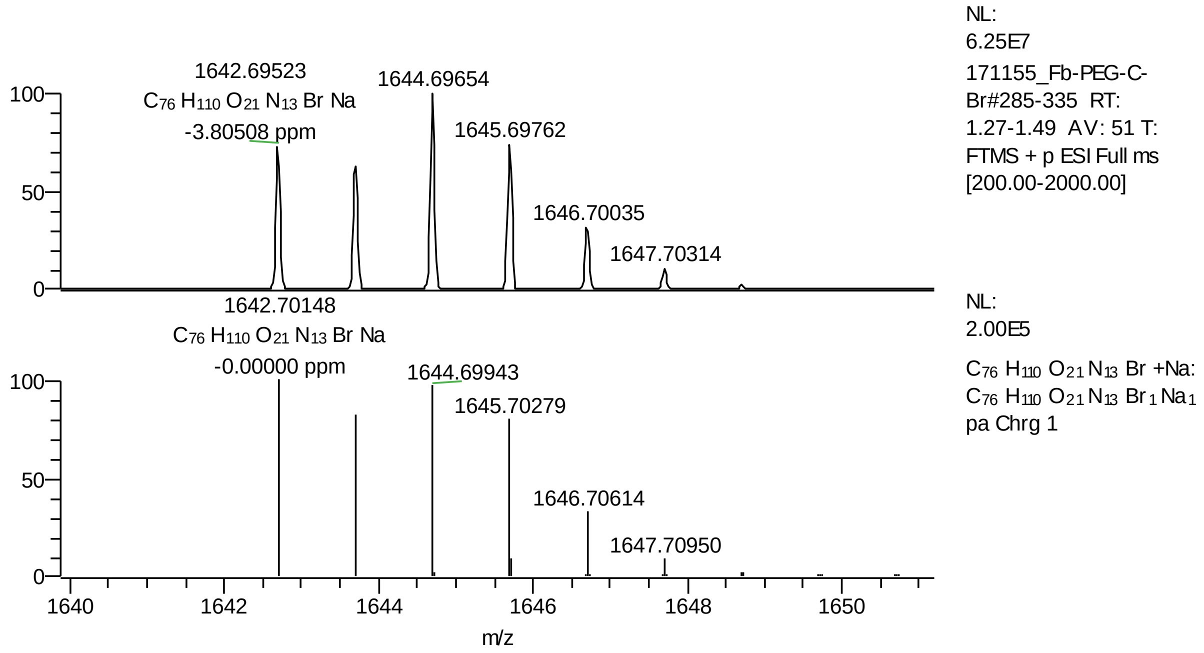

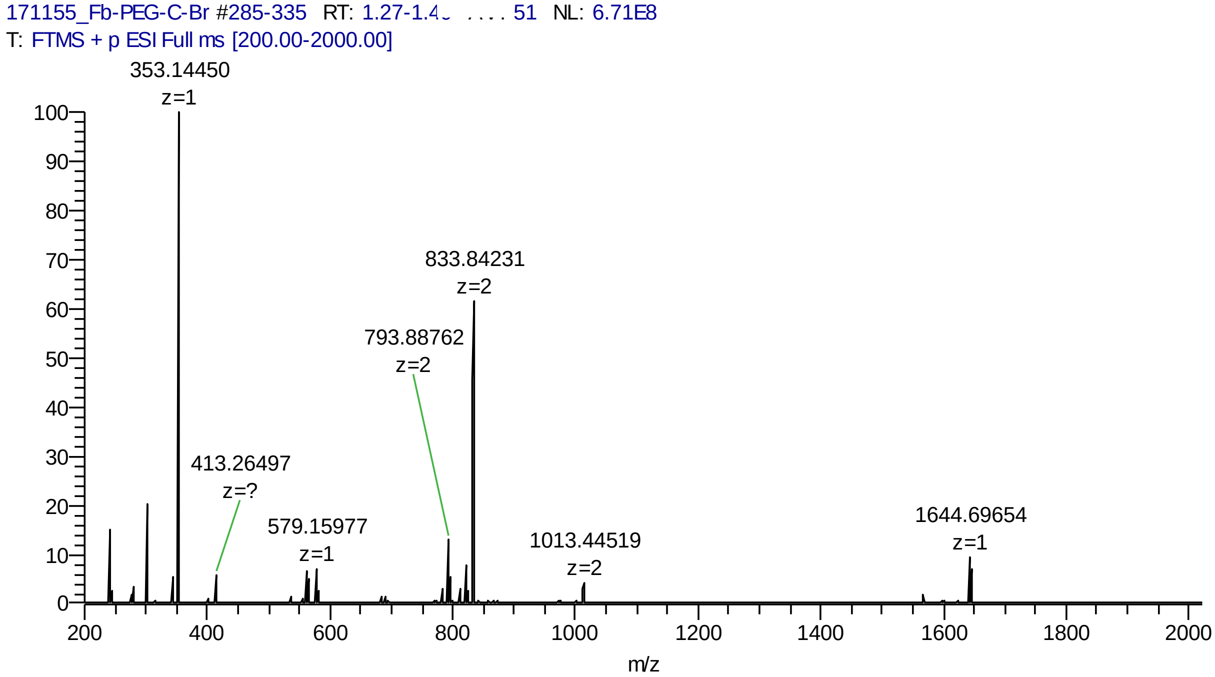

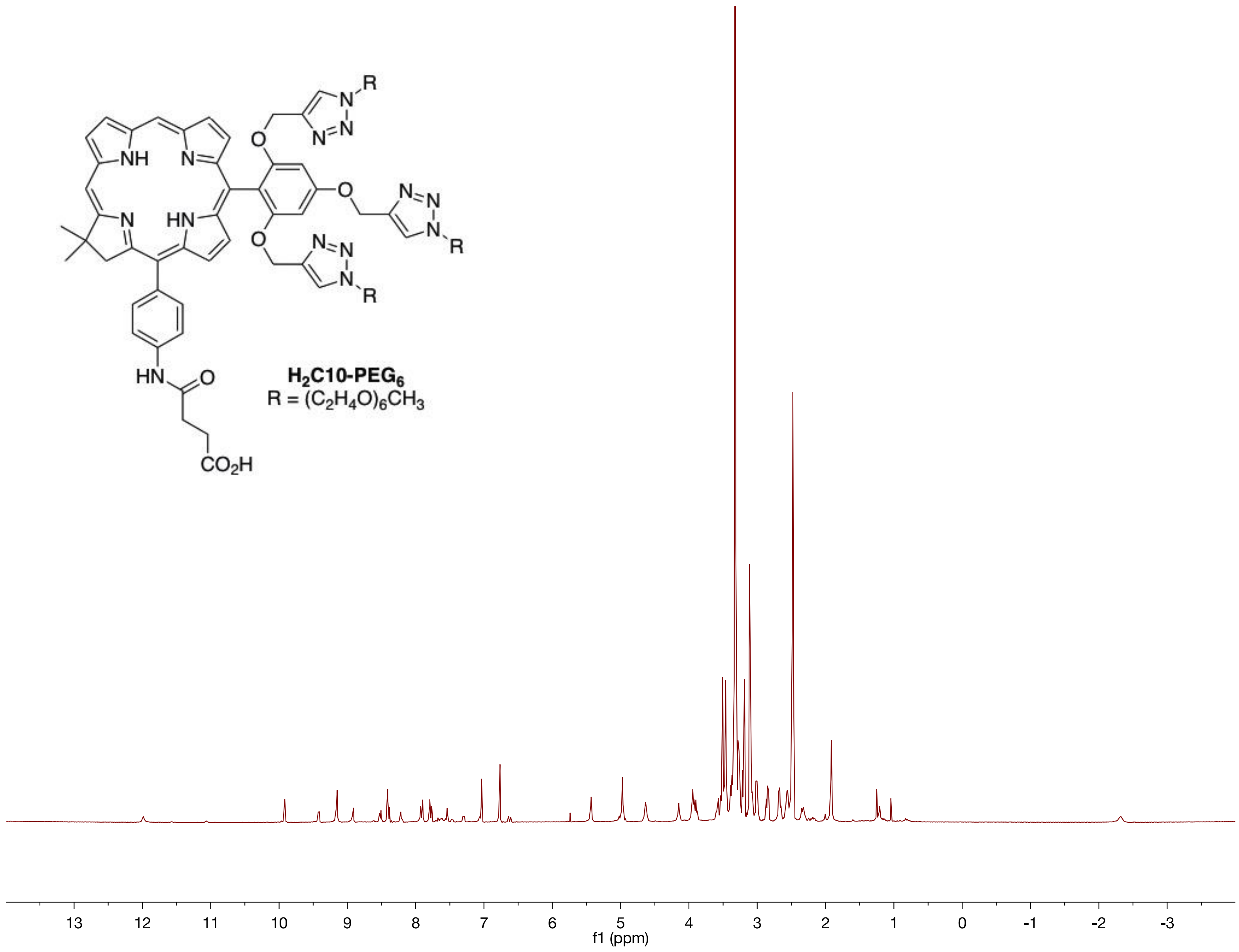

H2C10-PEG6
R = (C2H4O)6CH3
13
12
11
10
9
8
7
6
5
4
3
2
1
0
-1
-2
-3
f1 (ppm)

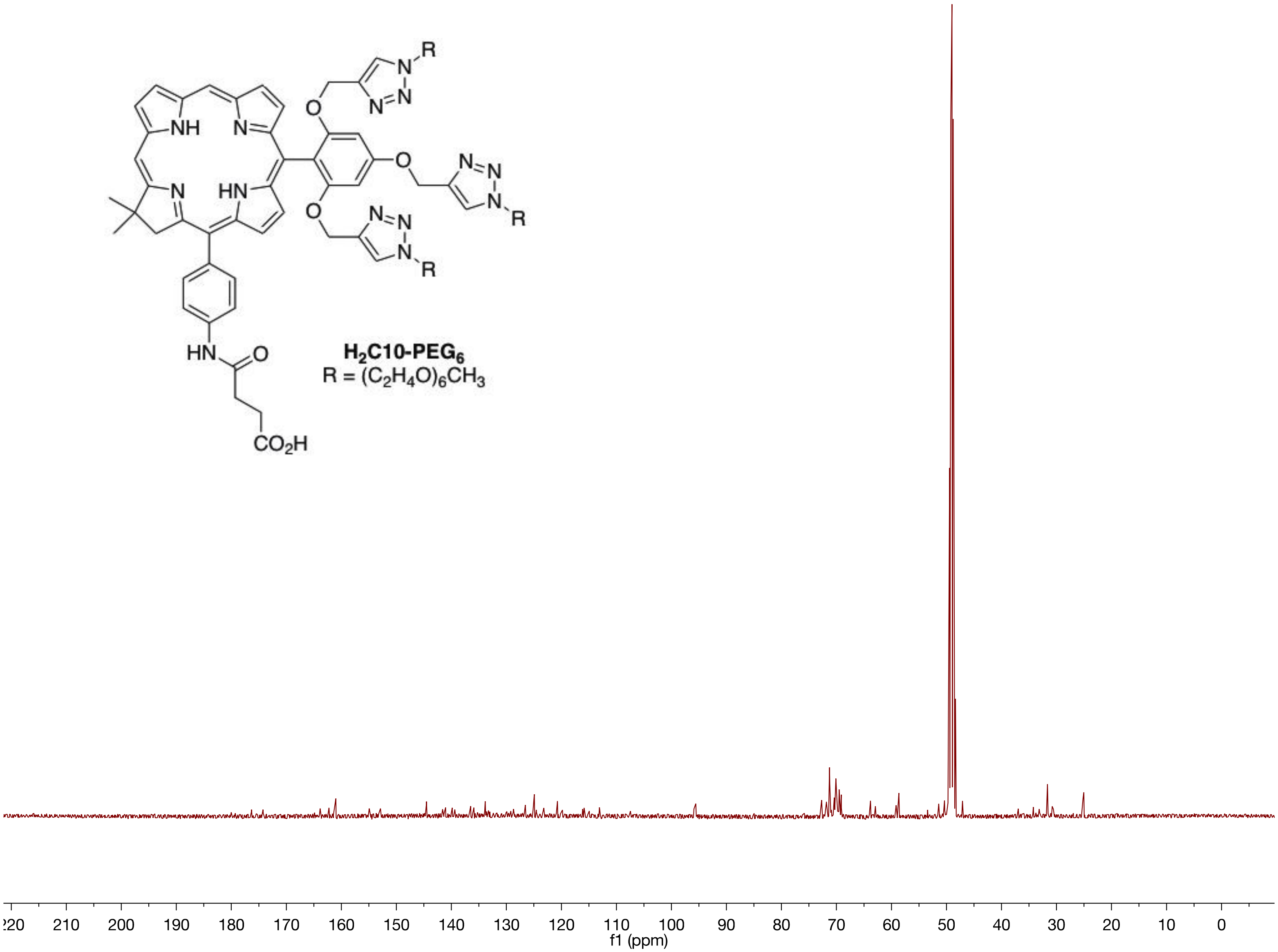

NH
N
N
HN
O
O
O
N
N
N
R
HN
O
CO2H
H2C10-PEG6
R = (C2H4O)6CH3
220 210 200 190 180 170 160 150 140 130 120 110 100 90 80 70 60 50 40 30 20 10 0
f1 (ppm)

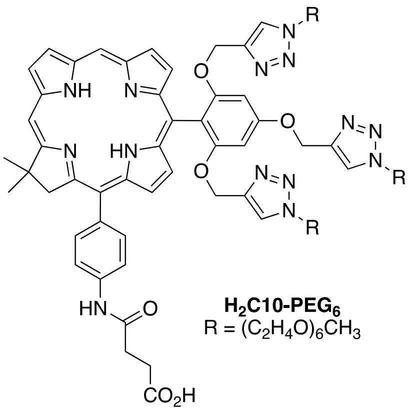

H_2C10-PEG$_6$
R = $(C_2H_4O)_6CH_3$

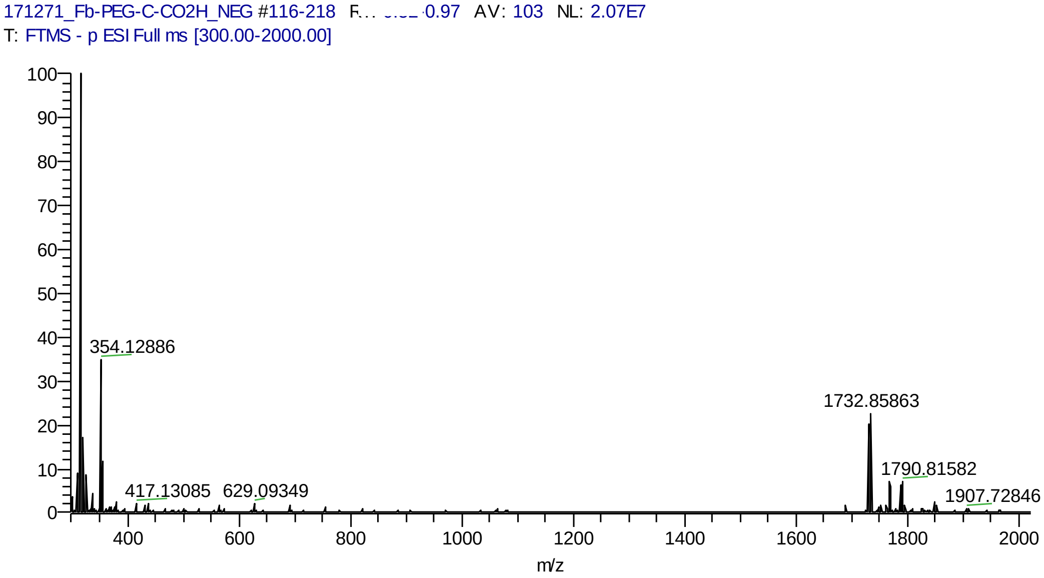

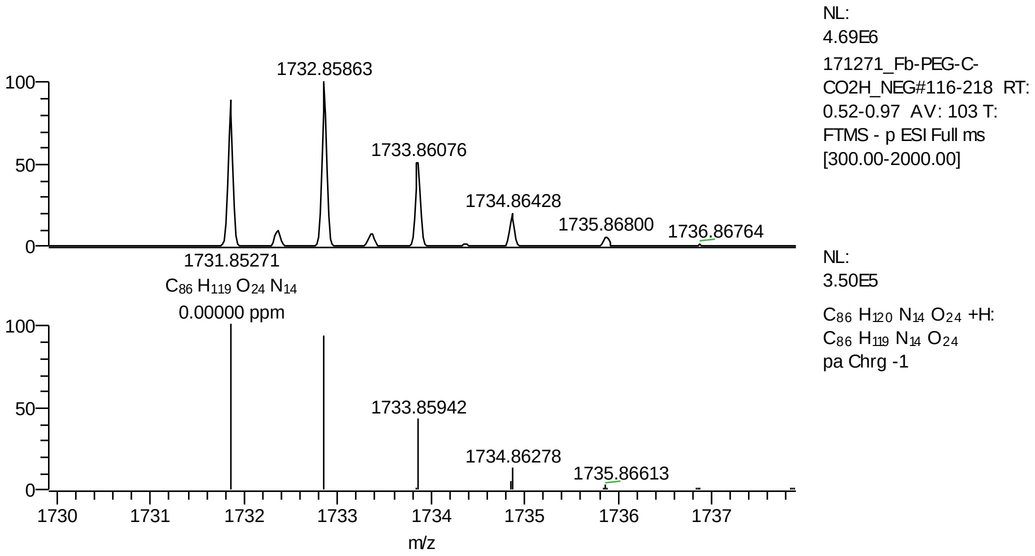

AB Sciex TOF/TOF™ Series Explorer™ 20981201

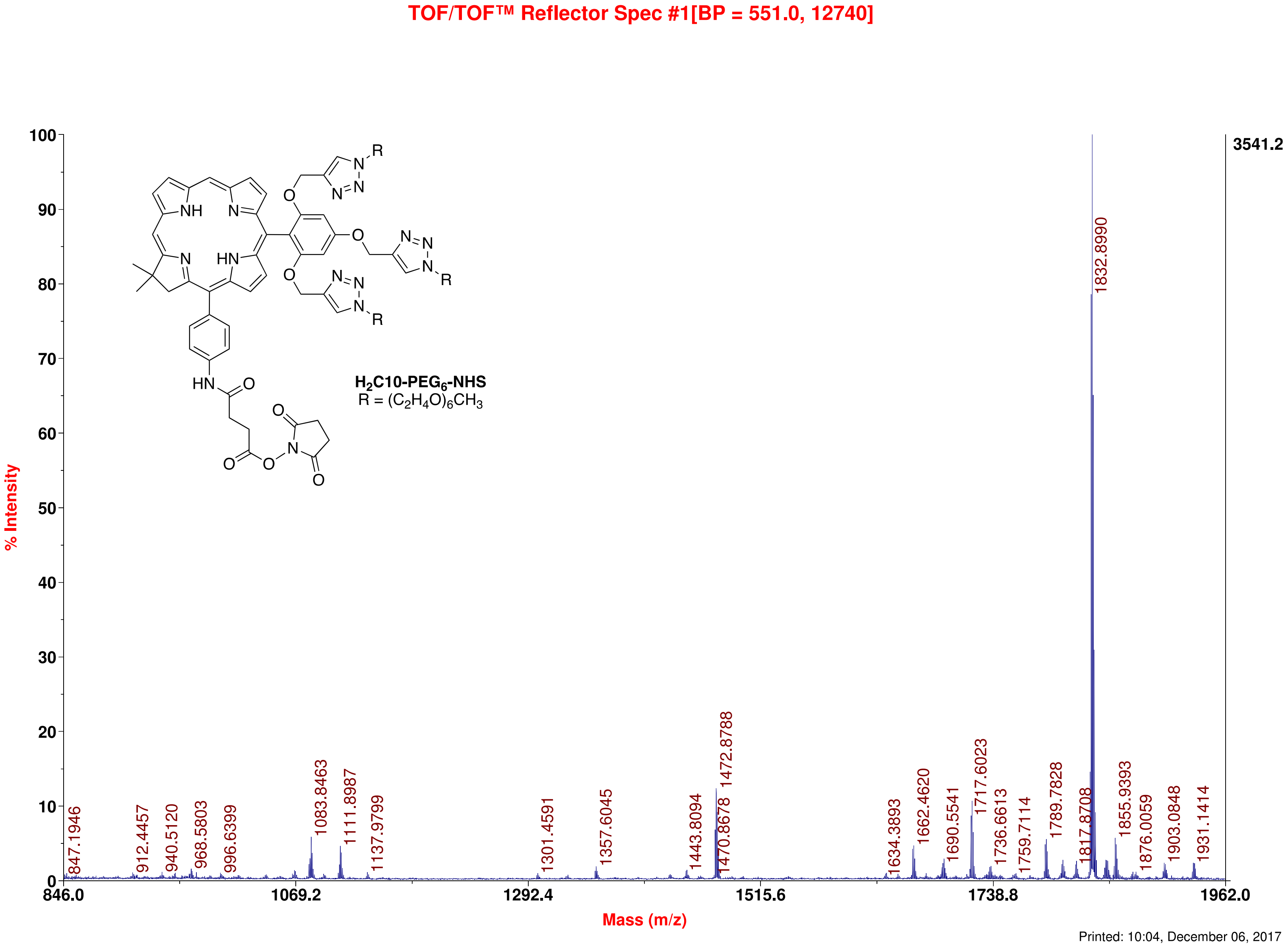

F:\User Project 1\Lindsey\RLiu\2017\041117\FbC-PEG-NHS.T2D

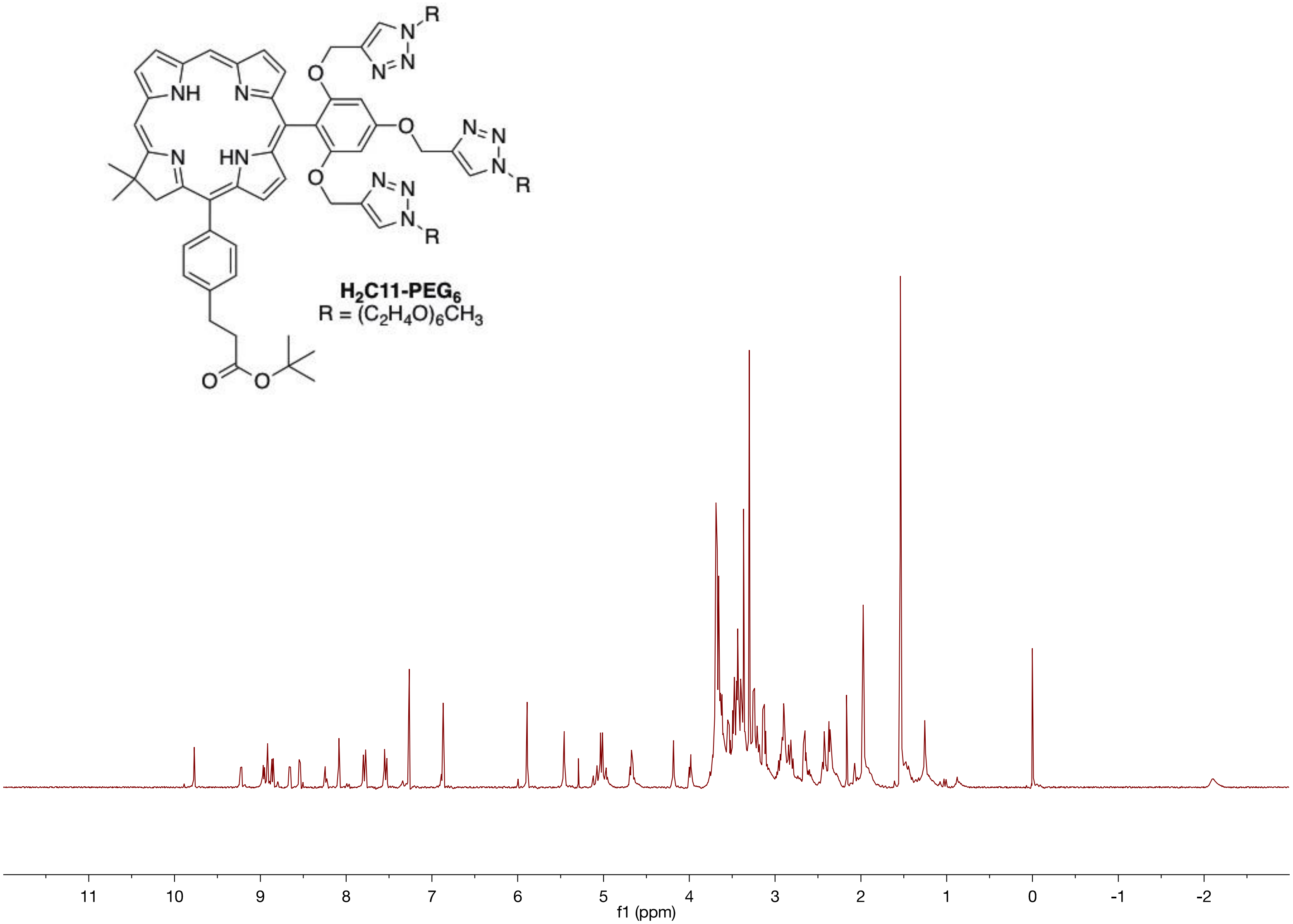
H2C11-PEG6
R = (C2H4O)6CH3
11 10 9 8 7 6 5 4 3 2 1 0 -1 -2
f1 (ppm)

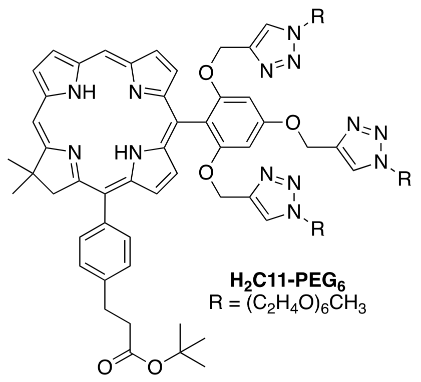

H_2C11-PEG$_6$
R = $(C_2H_4O)_6CH_3$

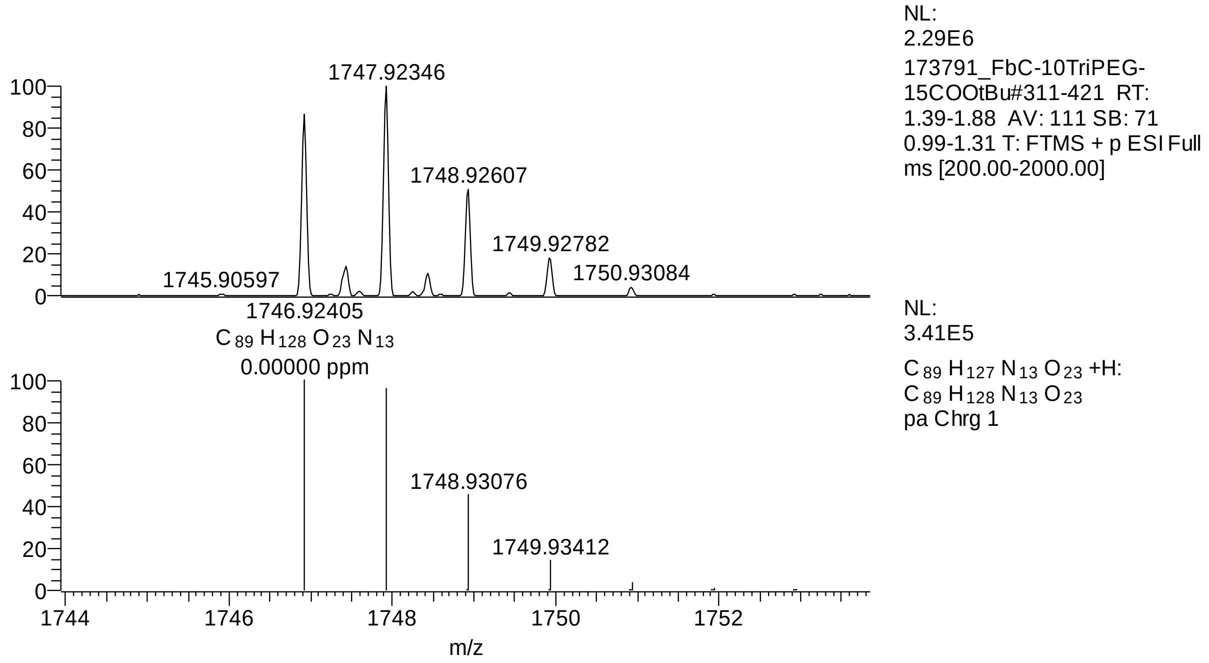

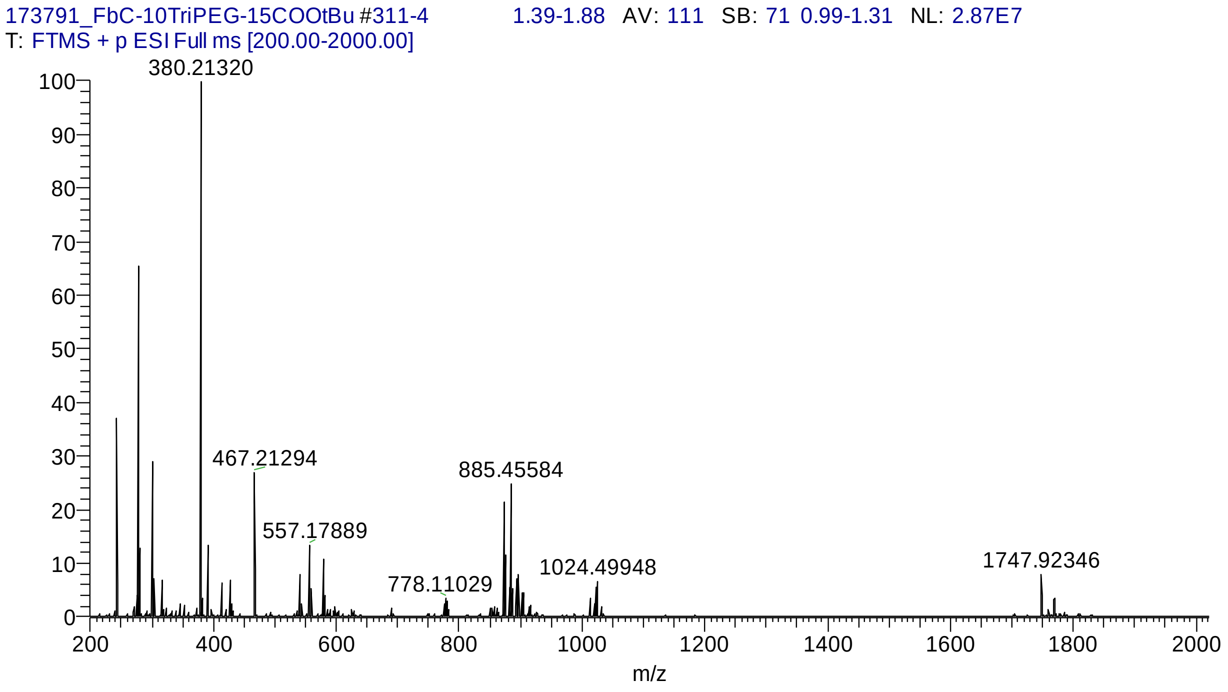

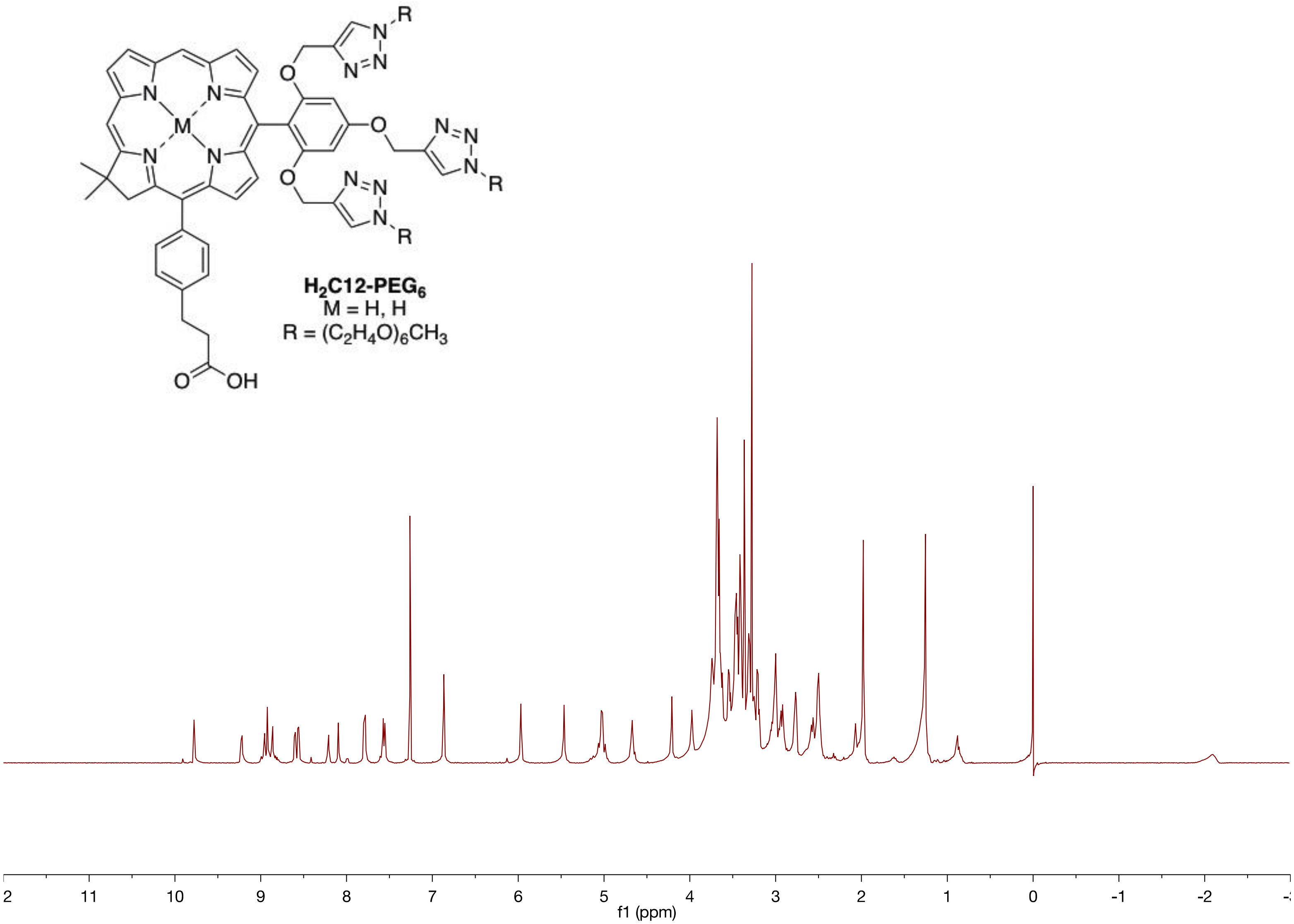
H$_2$C12-PEG$_6$
M = H, H
R = (C$_2$H$_4$O)$_6$CH$_3$
f1 (ppm)

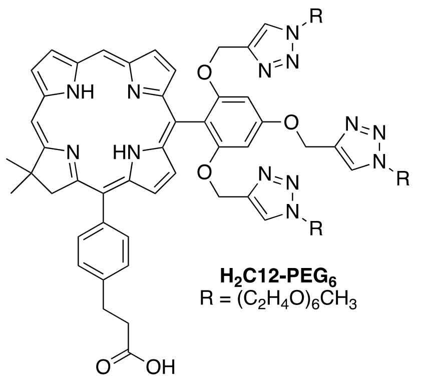

H_2C12-PEG$_6$
R = $(C_2H_4O)_6CH_3$

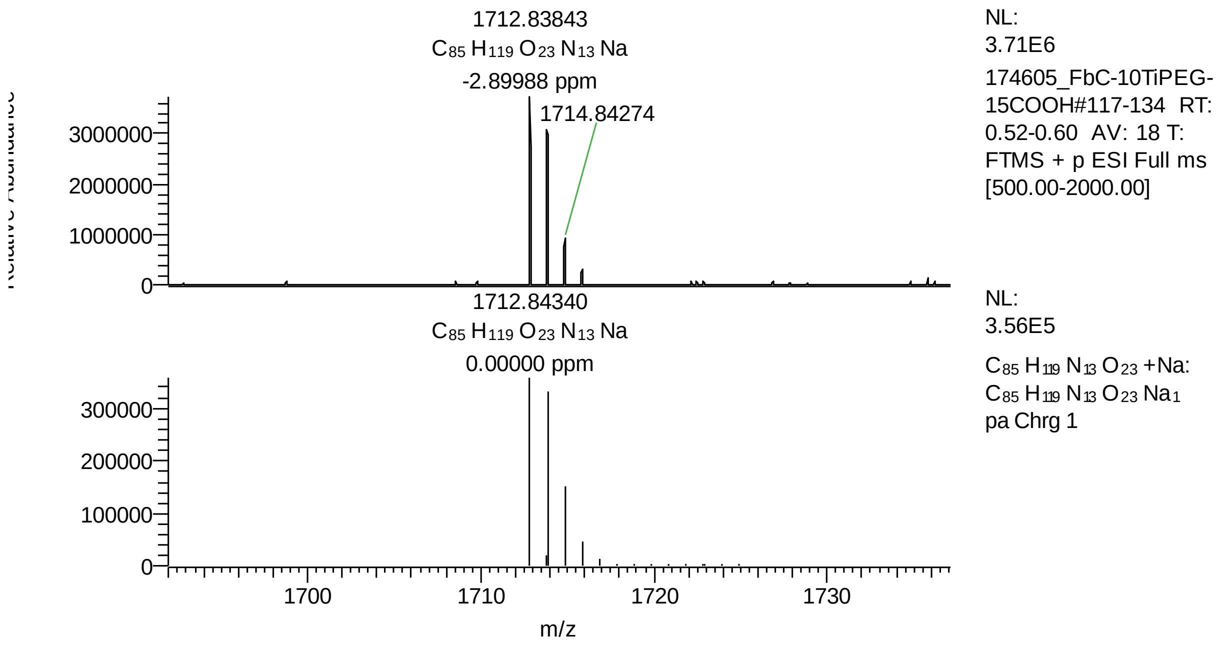

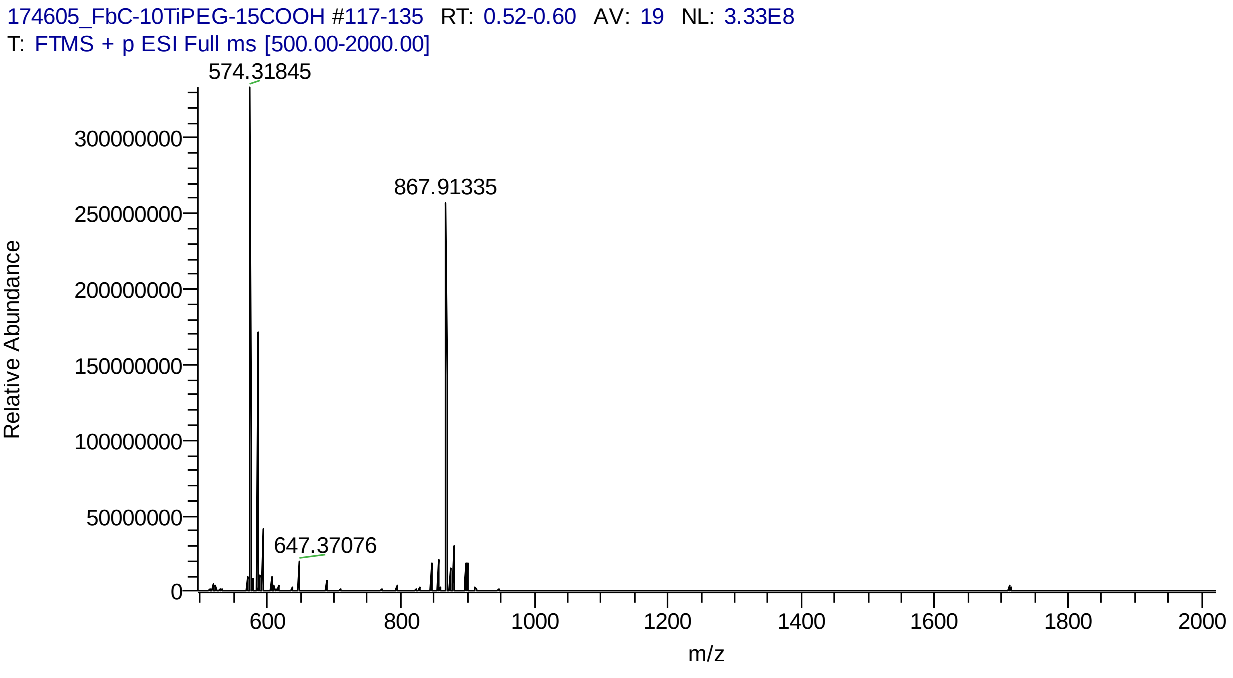

AB Sciex TOF/TOF™ Series Explorer™ 20981201

TOF/TOF™ Reflector Spec #1[BP = 1792.3, 651]

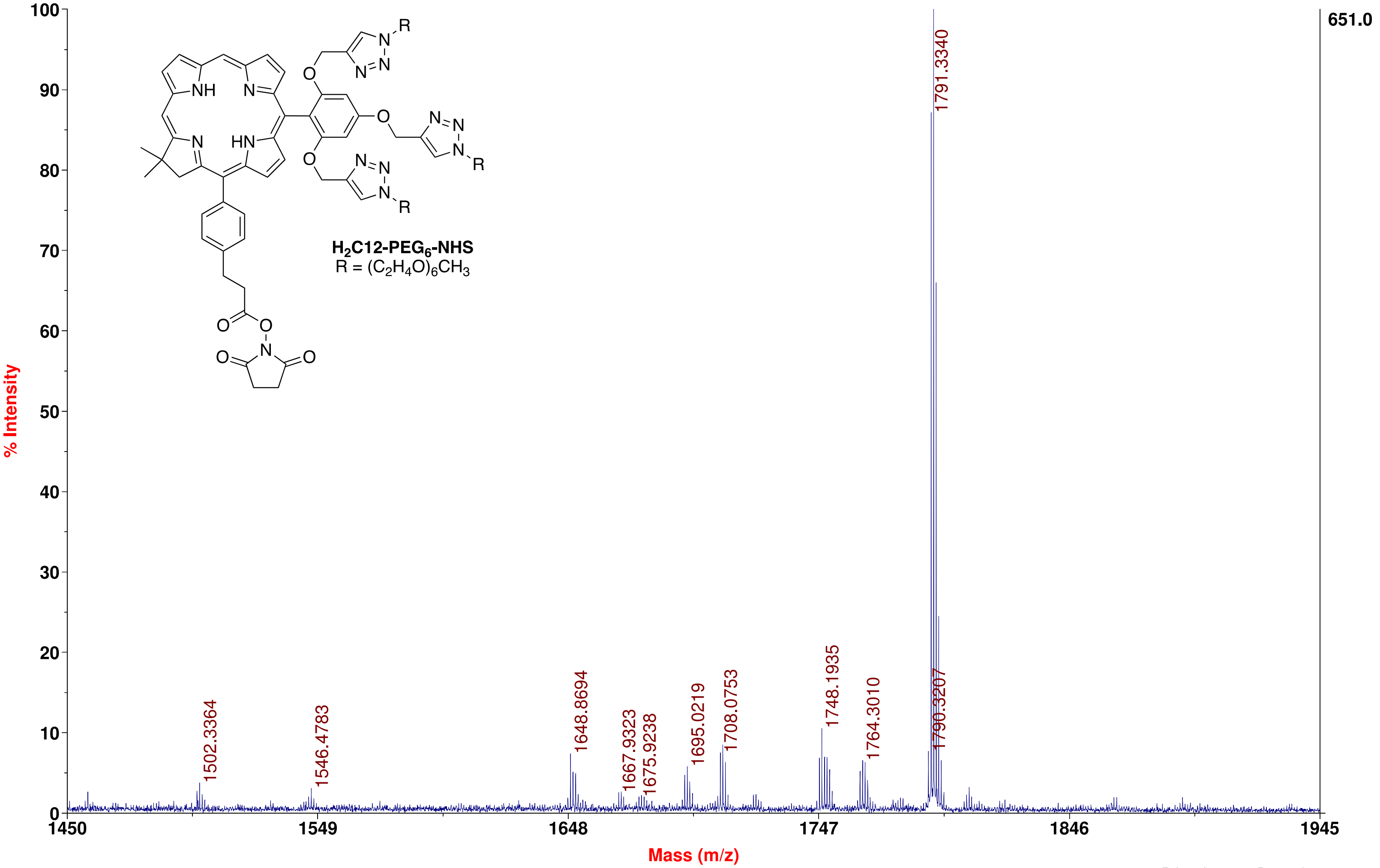

Printed: 10:05, December 06, 2017

F:\User Project 1\Lindsey\RLiu\2017\071517\FbC-10TriPEG-15NHS crude 2.T2D

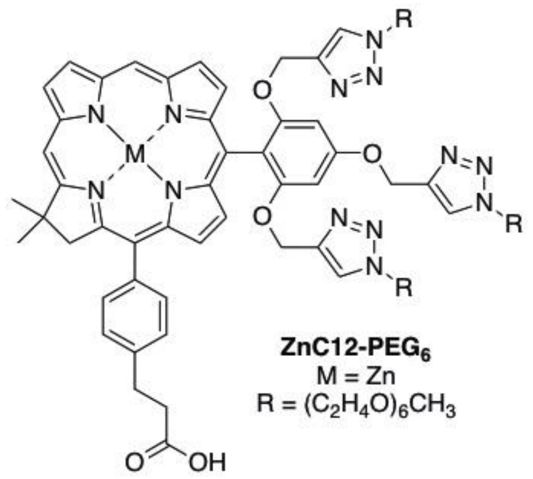
N
M
O
N=N
R
OH
ZnC12-PEG6
M = Zn
R = (C2H4O)6CH3

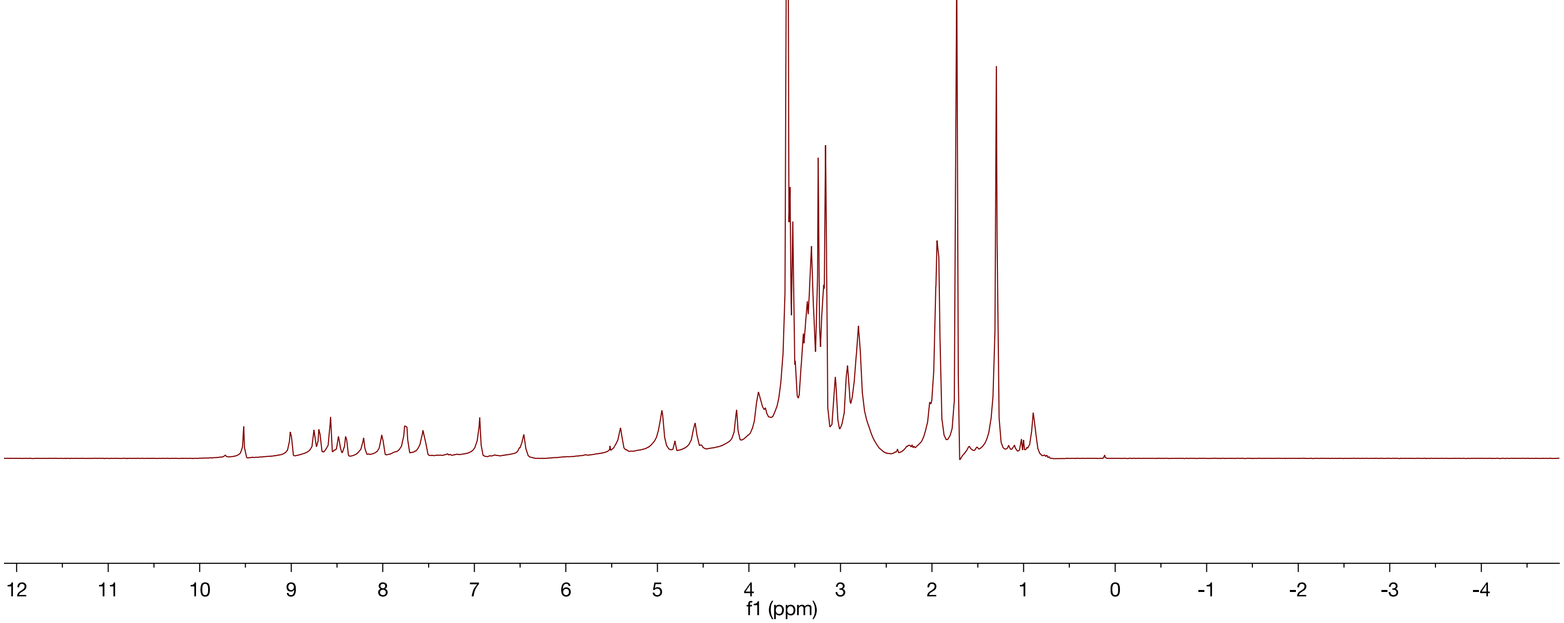
12
11
10
9
8
7
6
5
4
3
2
1
0
-1
-2
-3
-4
f1 (ppm)

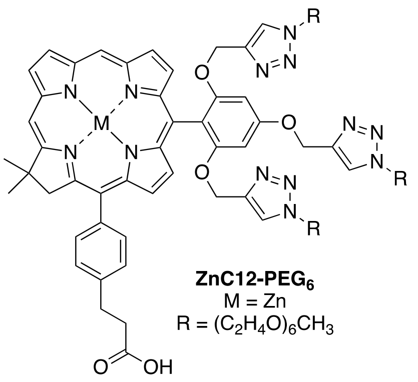

ZnC12-PEG$_6$
M = Zn
R = $(C_2H_4O)_6CH_3$

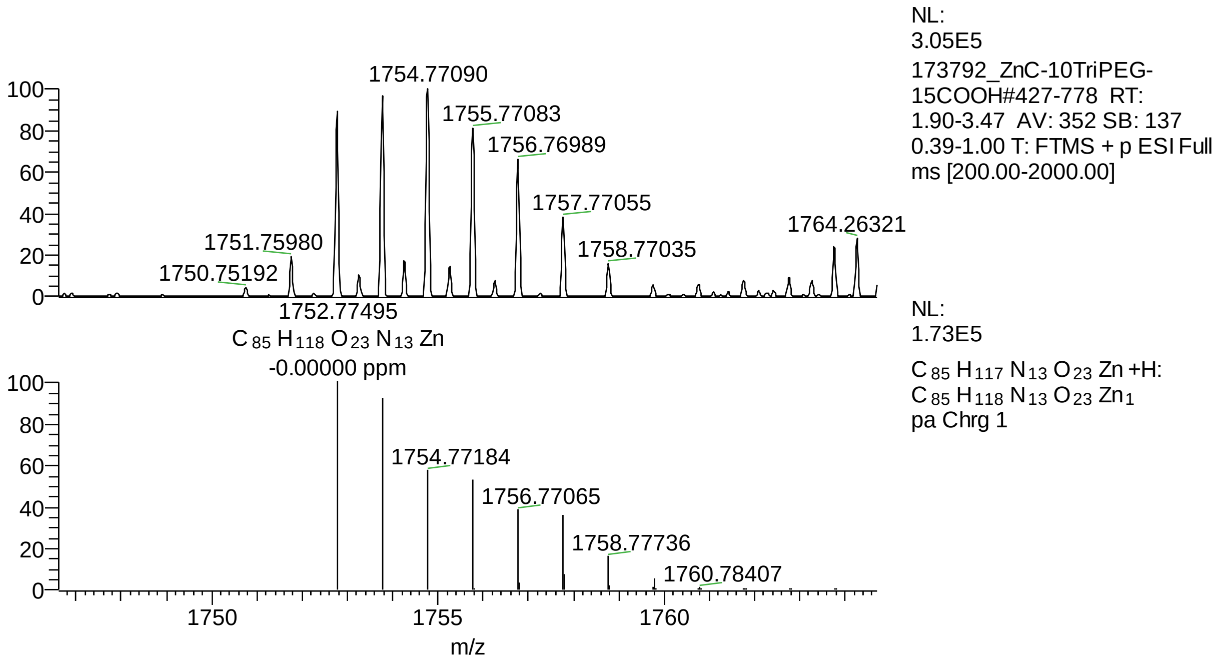

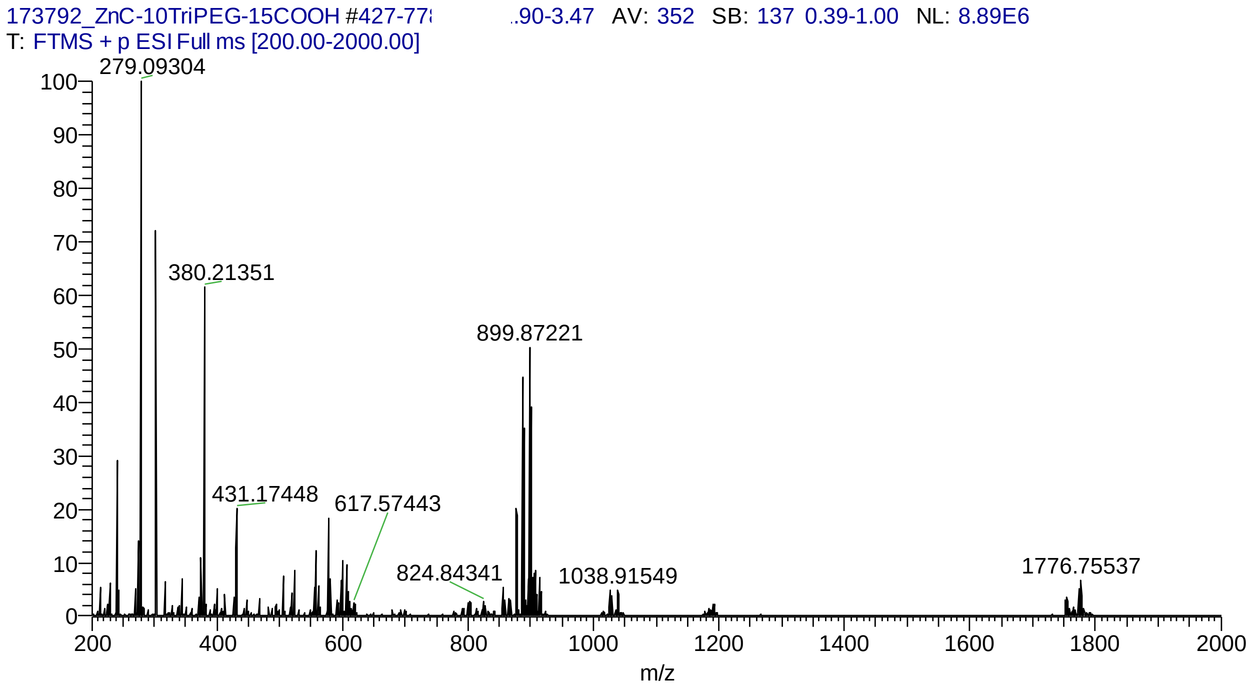

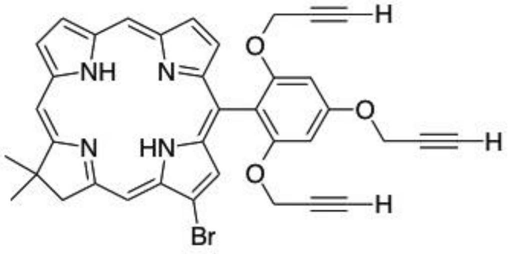

H_2C13

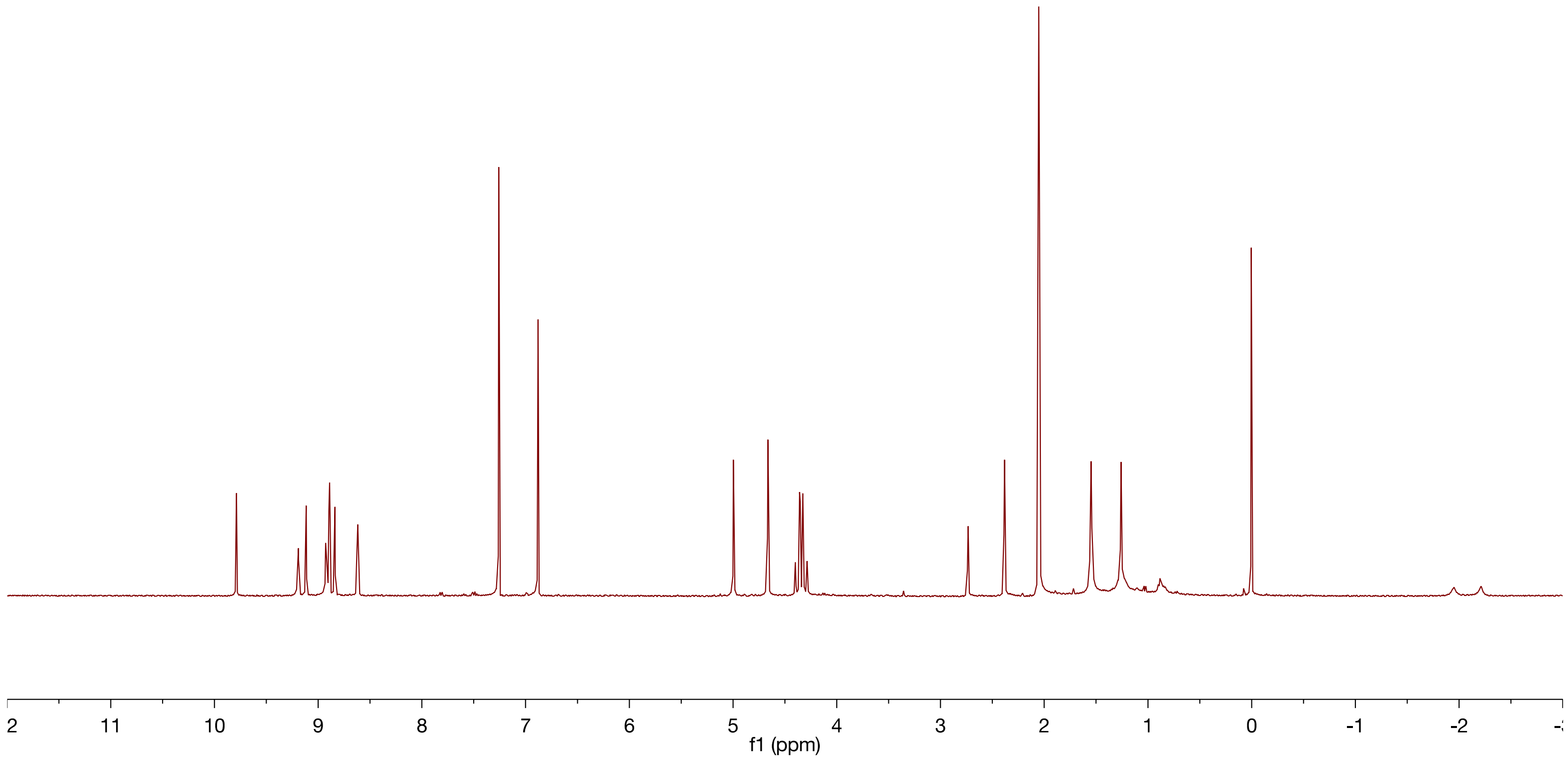

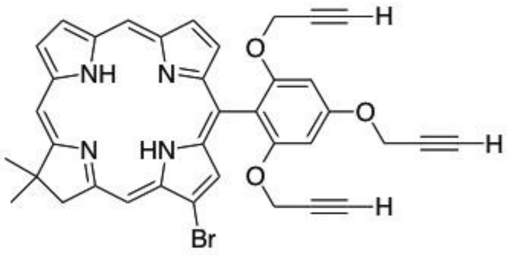
NH
N
N
HN
Br
O
O
O
H
H
H

H_2C13

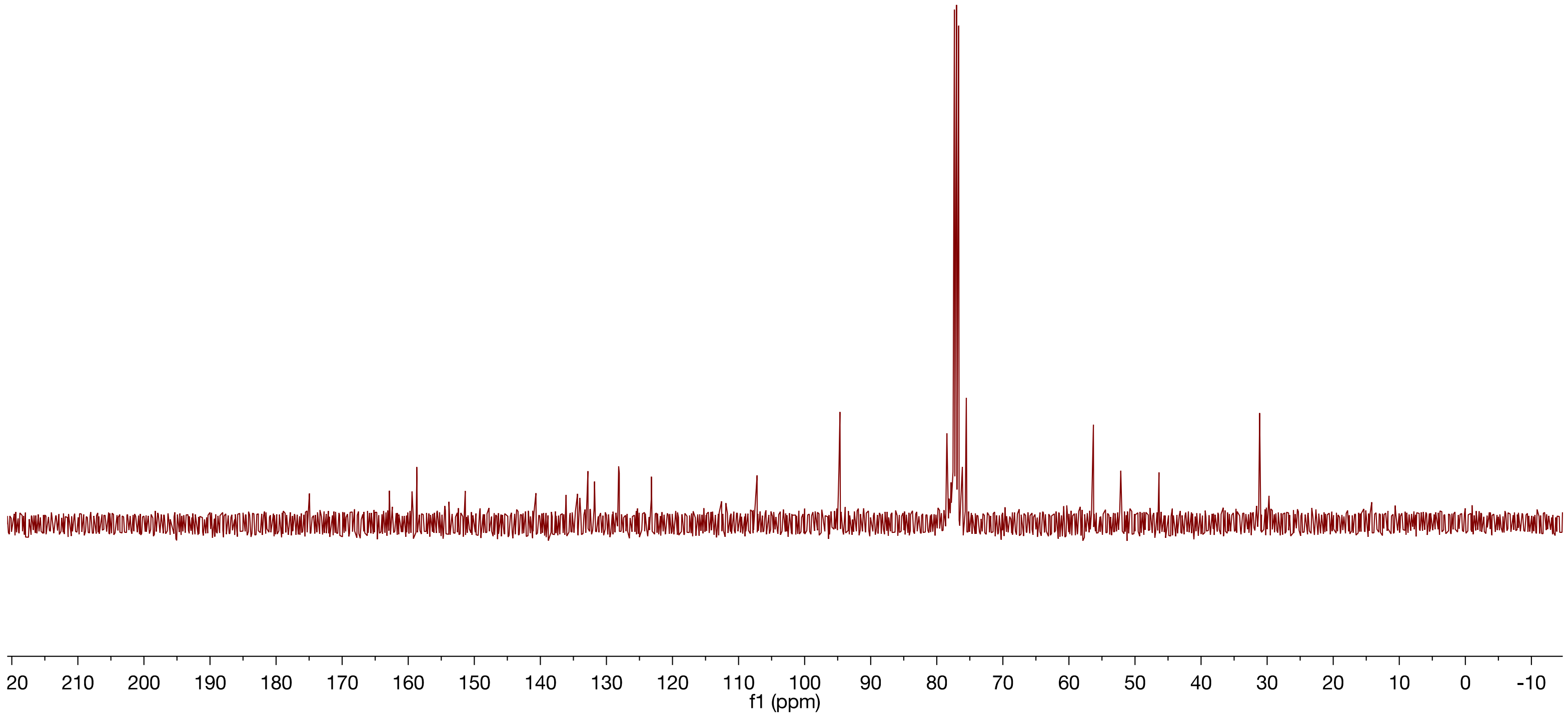
20
210
200
190
180
170
160
150
140
130
120
110
100
90
80
70
60
50
40
30
20
10
0
-10
f1 (ppm)

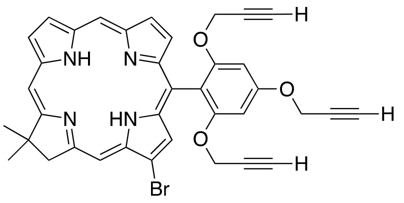
NH
N
N
HN
Br
O
O
O
H
H
H

H_2C13

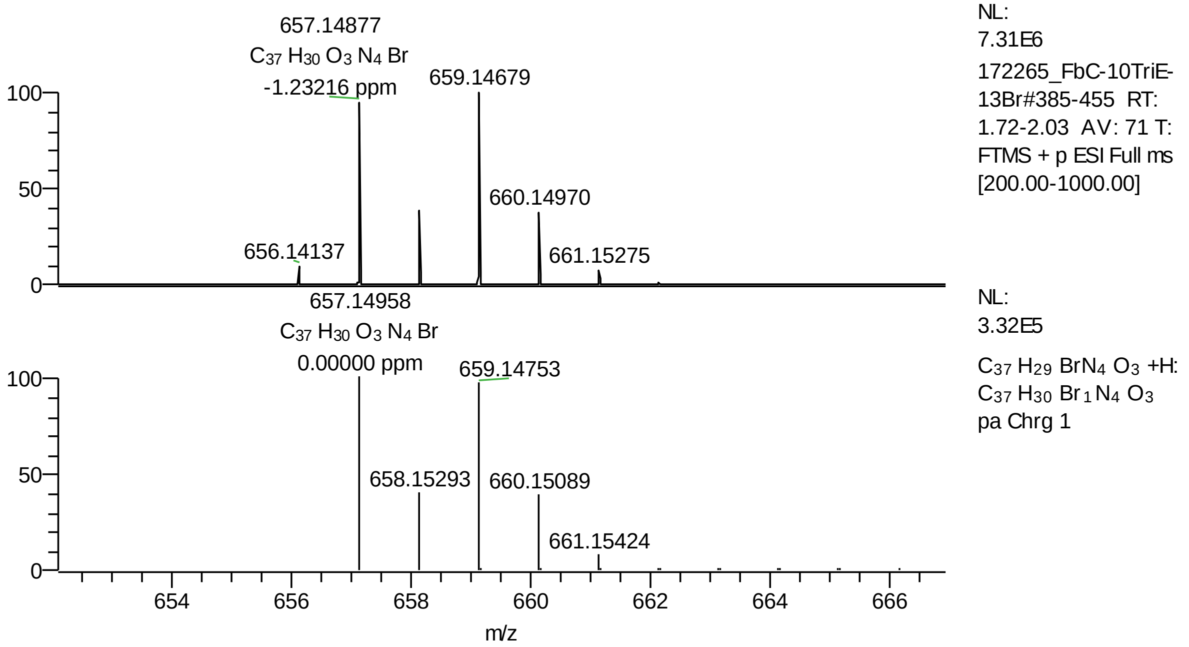
657.14877
C37 H30 O3 N4 Br
-1.23216 ppm
659.14679
660.14970
656.14137
661.15275
NL:
7.31E6
172265_FbC-10TriE-13Br#385-455 RT: 1.72-2.03 AV: 71 T: FTMS + p ESI Full ms [200.00-1000.00]
657.14958
C37 H30 O3 N4 Br
0.00000 ppm
659.14753
658.15293
660.15089
661.15424
NL:
3.32E5
C37 H29 BrN4 O3 +H:
C37 H30 Br 1 N4 O3
pa Chrg 1
100
50
0
654
656
658
660
662
664
666
m/z

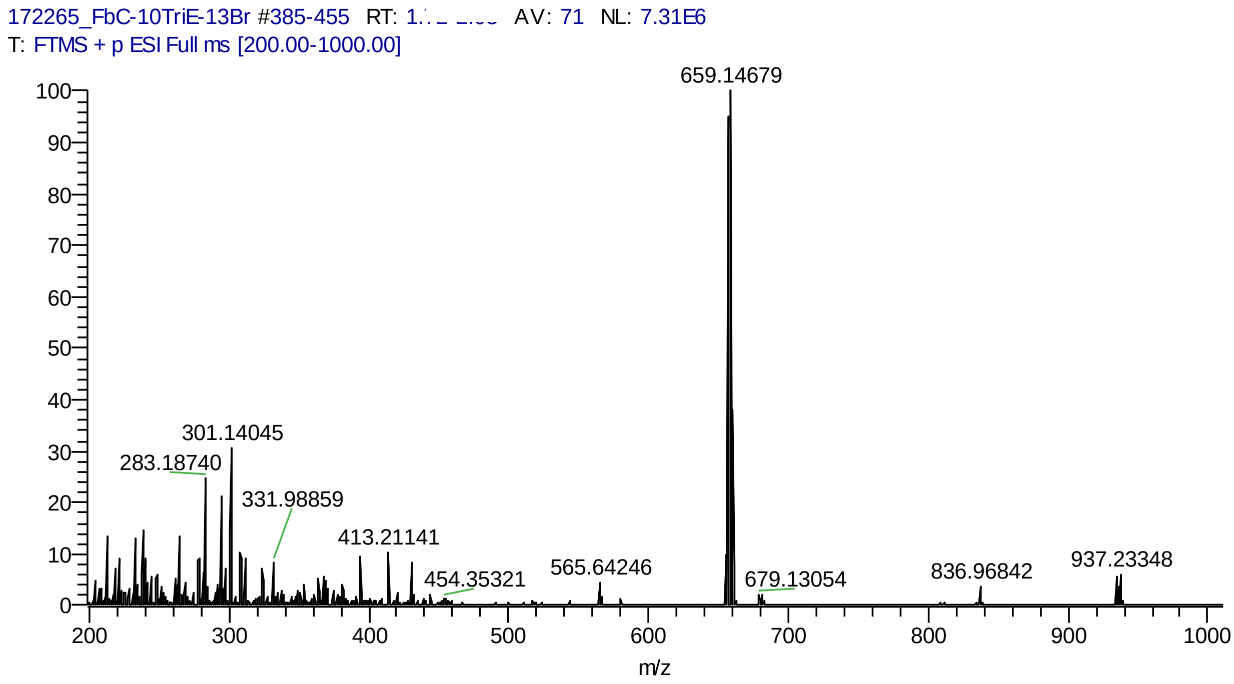
172265_FbC-10TriE-13Br #385-455 RT:
AV: 71 NL: 7.31E6
T: FTMS + p ESI Full ms [200.00-1000.00]
659.14679
301.14045
283.18740
331.98859
413.21141
454.35321
565.64246
679.13054
836.96842
937.23348
100
90
80
70
60
50
40
30
20
10
0
200
300
400
500
600
700
800
900
1000
m/z

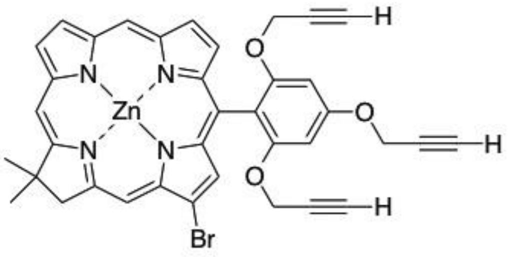

ZnC13

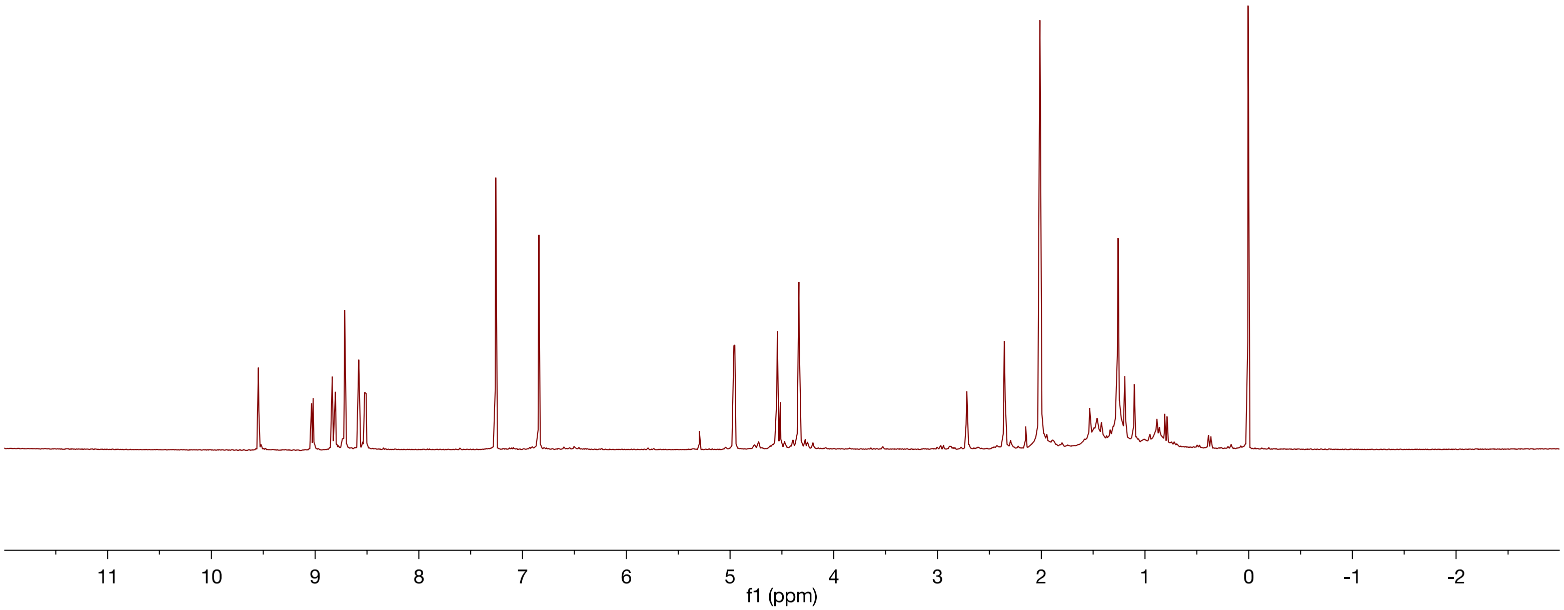

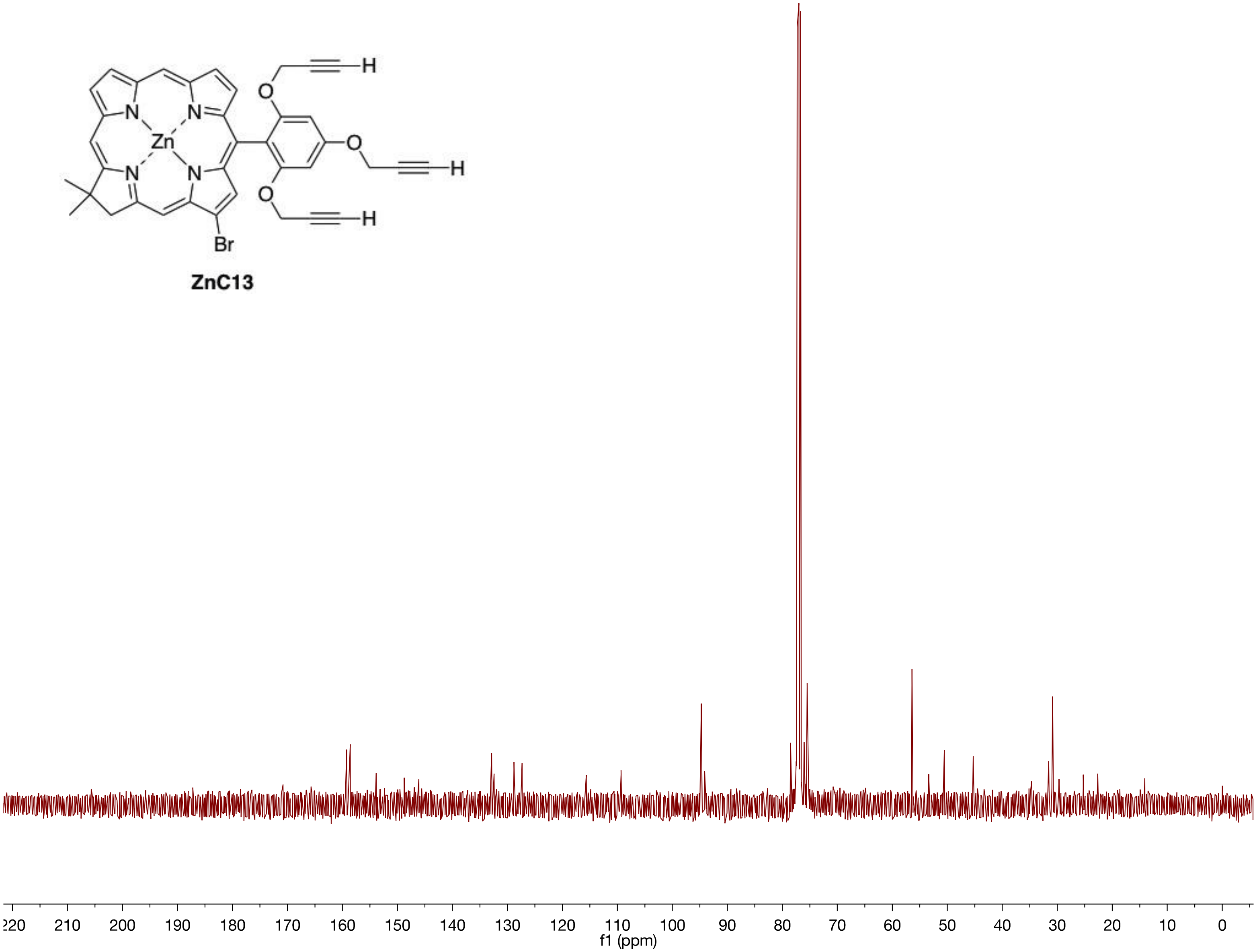
N
N
N
N
Zn
O
O
O
H
H
H
Br
220
210
200
190
180
170
160
150
140
130
120
110
100
90
80
70
60
50
40
30
20
10
0
f1 (ppm)

ZnC13

AB Sciex TOF/TOF™ Series Explorer™ 20981201

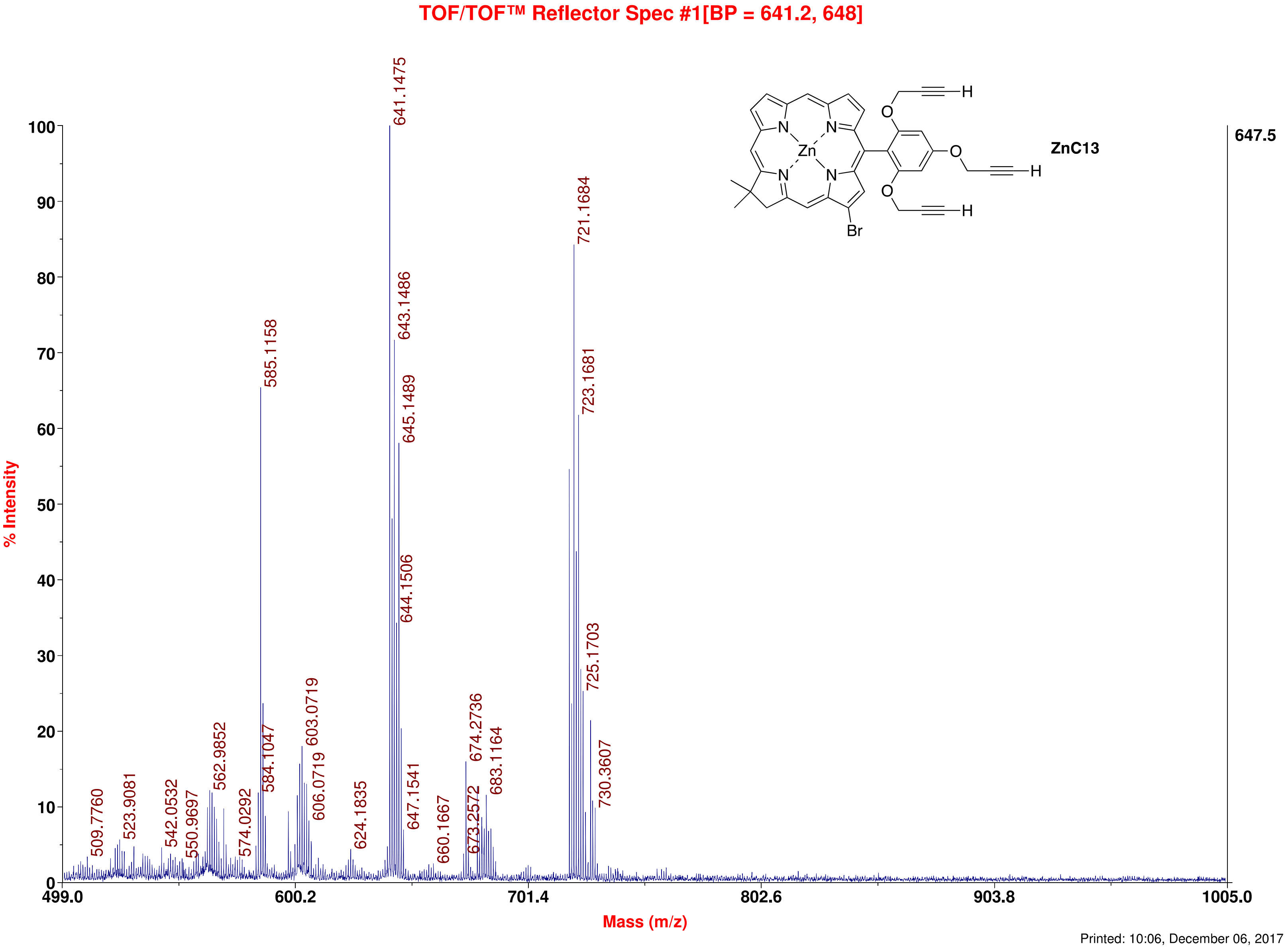

Printed: 10:06, December 06, 2017

F:\User Project 1\Lindsey\RLiu\2017\041917\ZnC-13Br.T2D

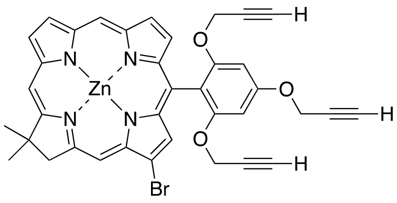
N
N
N
N
Zn
O
O
O
H
H
H
Br

ZnC13

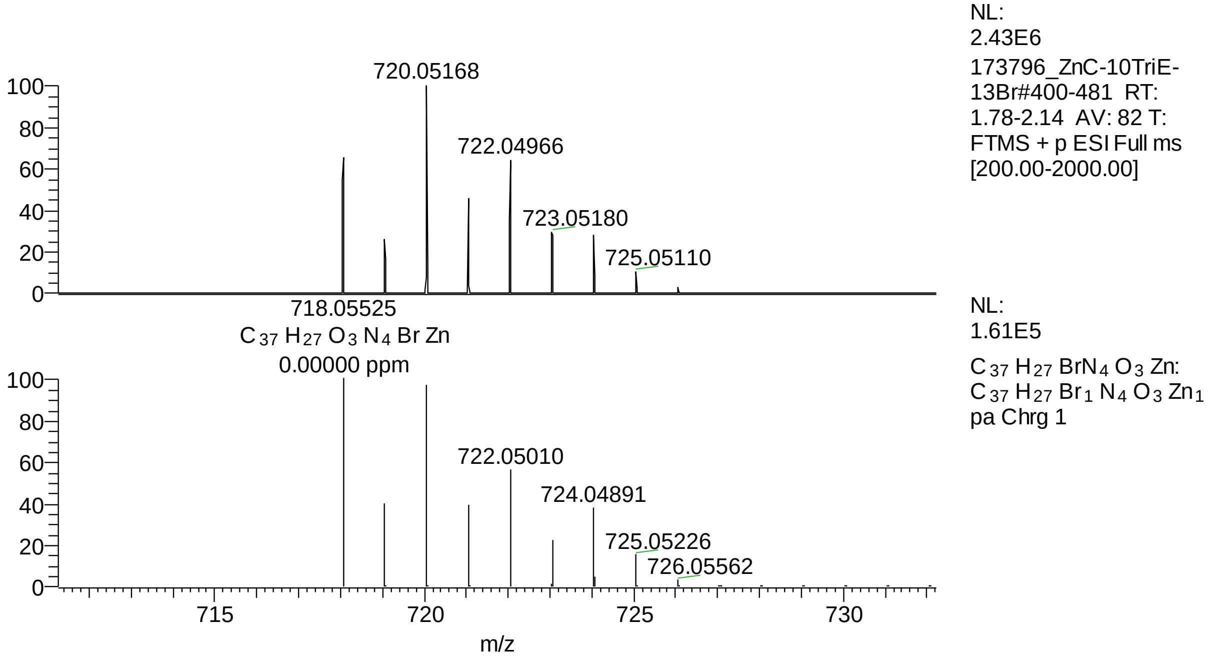
NL:
2.43E6
173796_ZnC-10TriE-13Br#400-481 RT: 1.78-2.14 AV: 82 T: FTMS + p ESI Full ms [200.00-2000.00]
720.05168
722.04966
723.05180
725.05110
718.05525
$C_{37}H_{27}O_3N_4BrZn$
0.00000 ppm
NL:
1.61E5
$C_{37}H_{27}BrN_4O_3Zn$:
$C_{37}H_{27}Br_1N_4O_3Zn_1$
pa Chrg 1
722.05010
724.04891
725.05226
726.05562
m/z

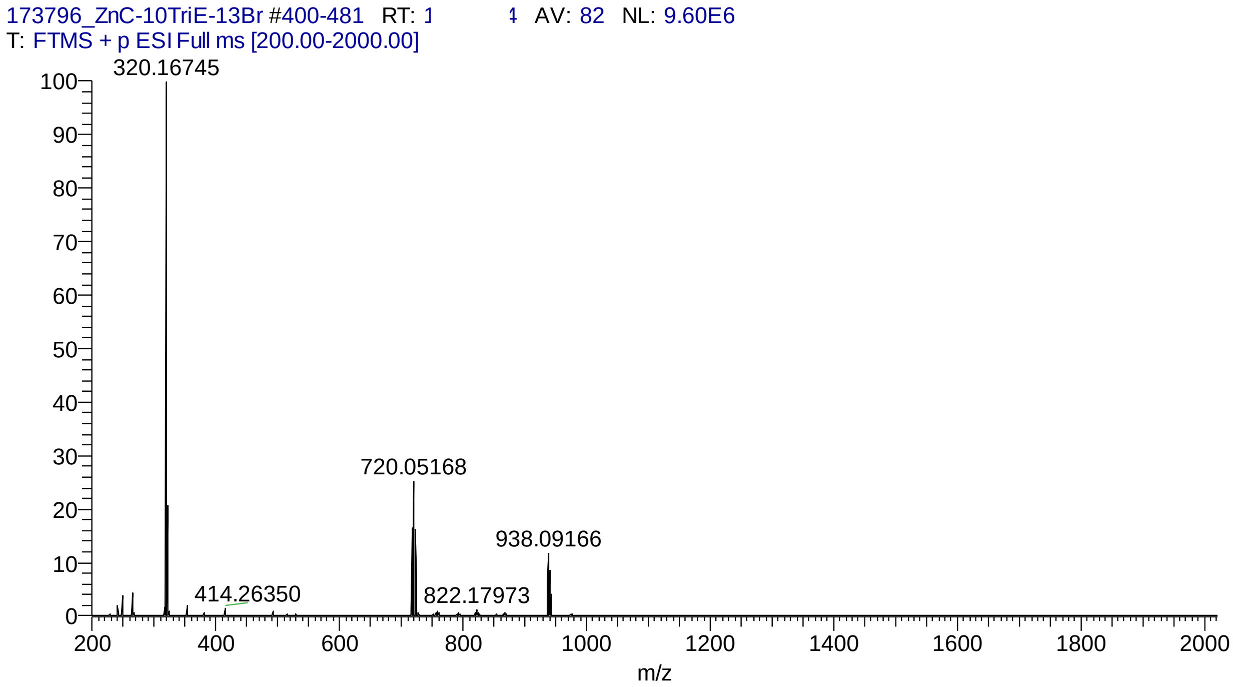
173796_ZnC-10TriE-13Br #400-481 RT: 1 4 AV: 82 NL: 9.60E6
T: FTMS + p ESI Full ms [200.00-2000.00]
320.16745
414.26350
720.05168
822.17973
938.09166
m/z

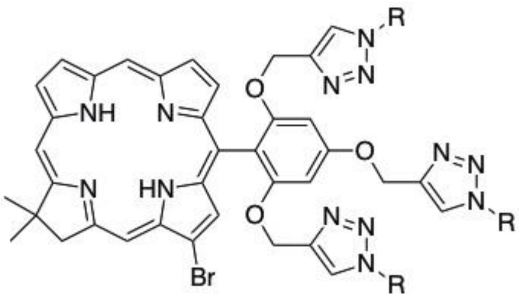

H_2C14-PEG$_6$
$R = (C_2H_4O)_6CH_3$

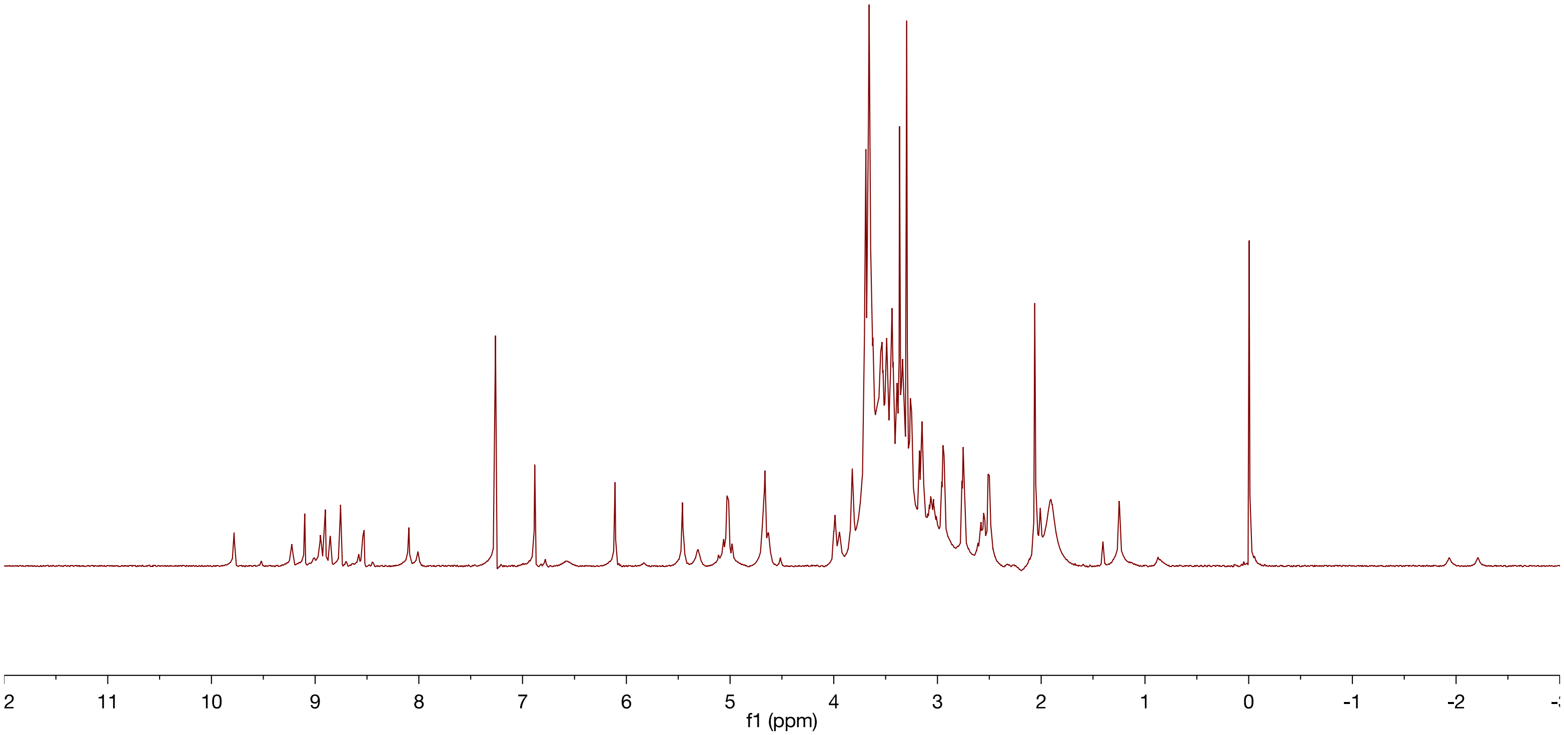

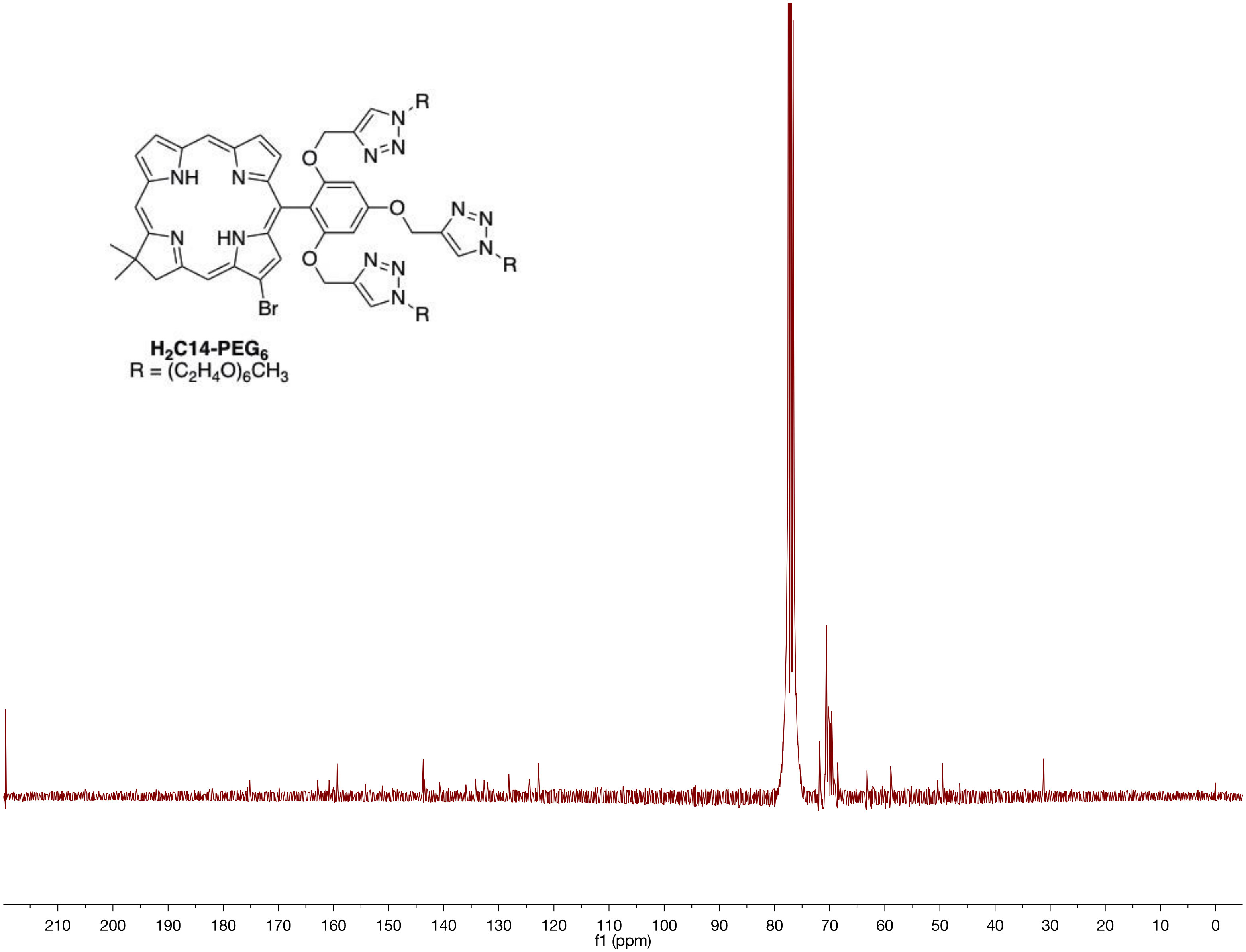

H_2C14-PEG$_6$
R = $(C_2H_4O)_6CH_3$

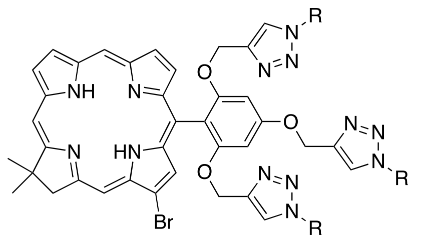

H_2C14-PEG$_6$
R = $(C_2H_4O)_6CH_3$

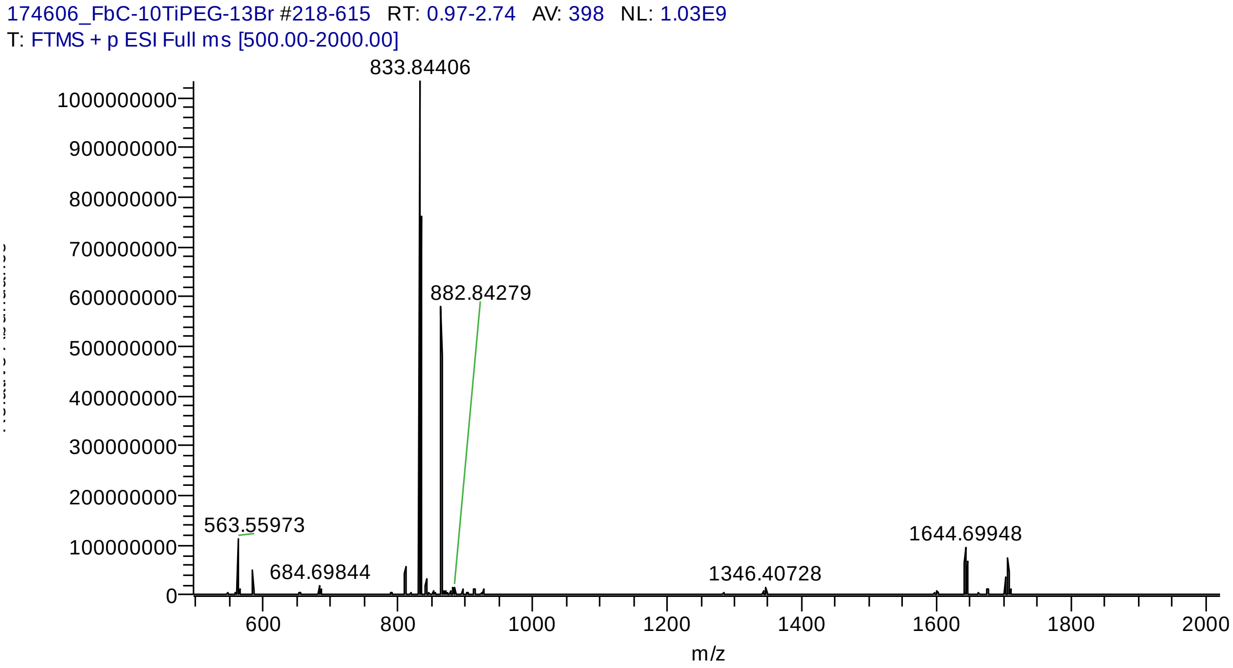

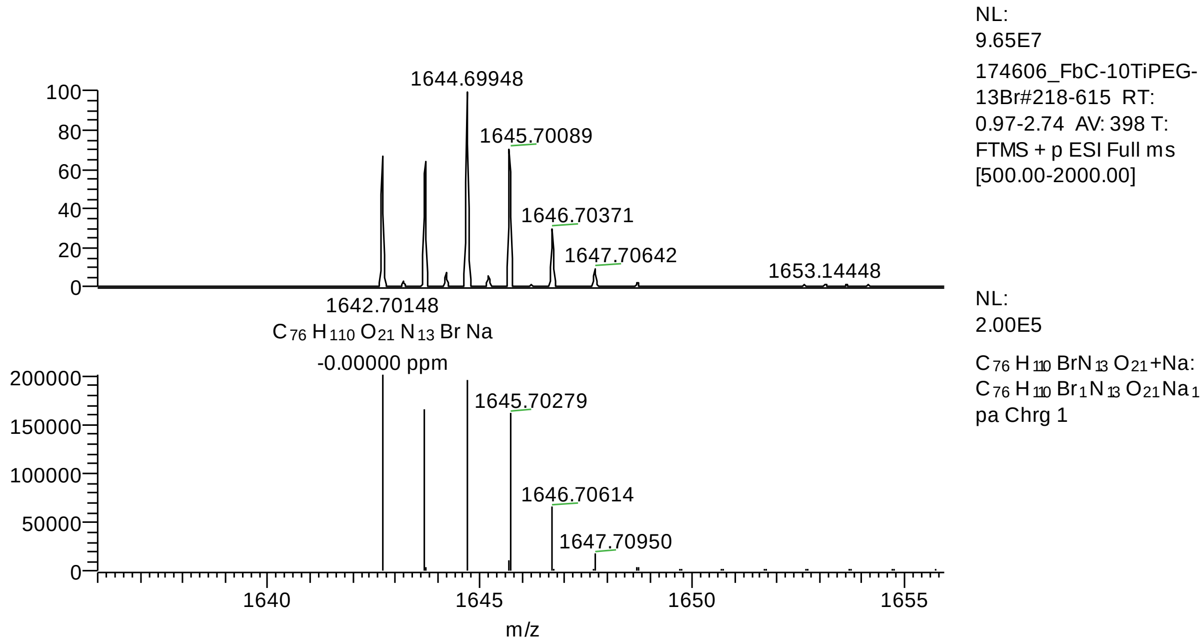

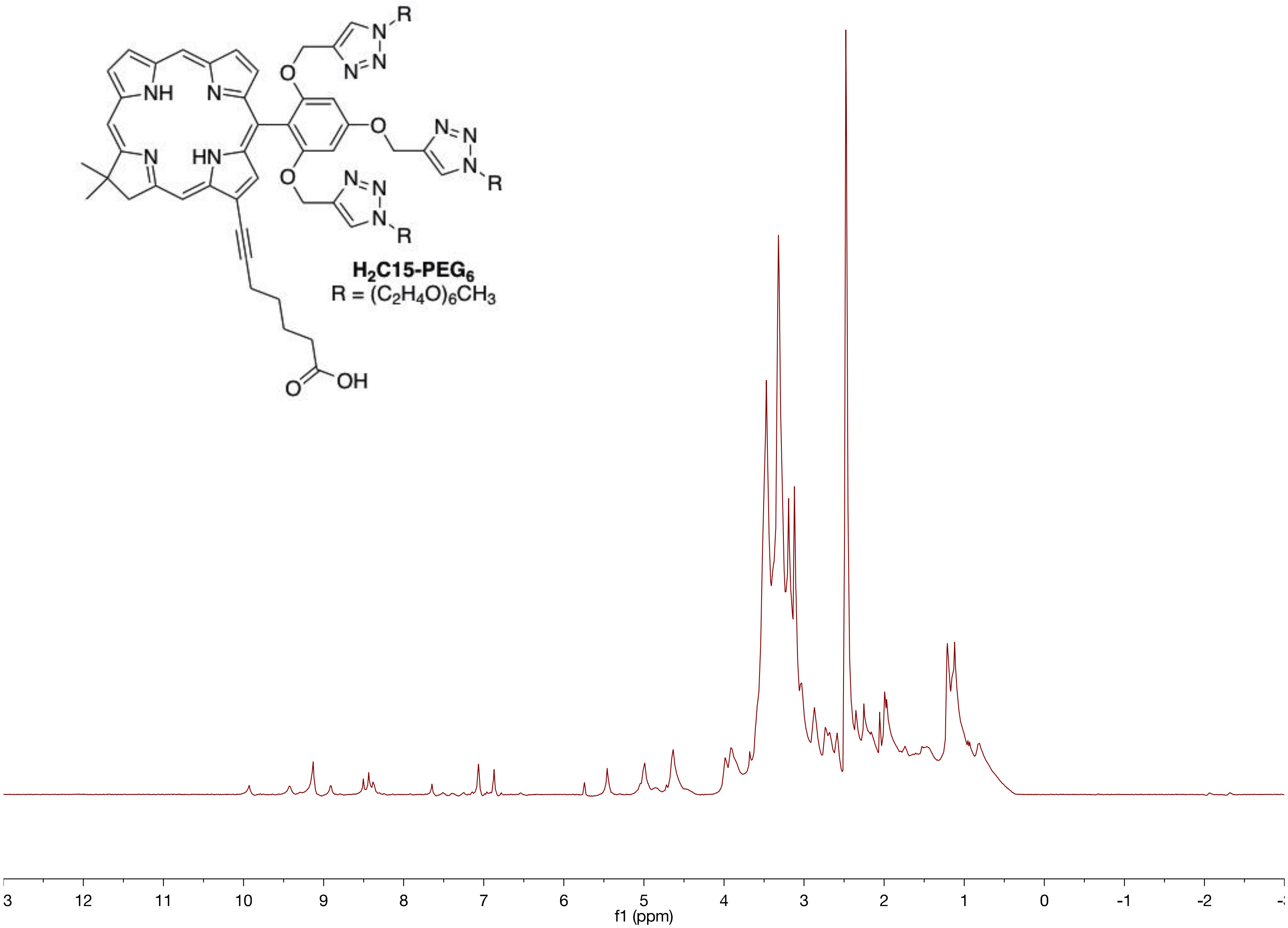
NH
N
N
HN
O
O
O
N
N
N
N
N
N
N
N
N
R
R
R
OH
O
H2C15-PEG6
R = (C2H4O)6CH3
12
11
10
9
8
7
6
5
4
3
2
1
0
-1
-2
f1 (ppm)

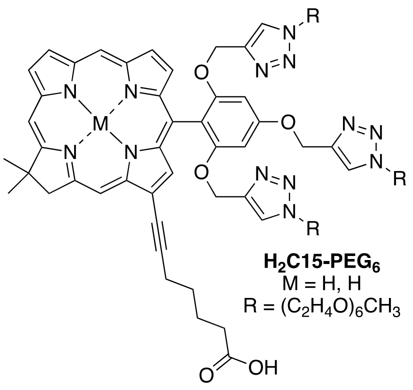
N
N
N
R
O
O
O
M
H2C15-PEG6
M = H, H
R = (C2H4O)6CH3
OH
O

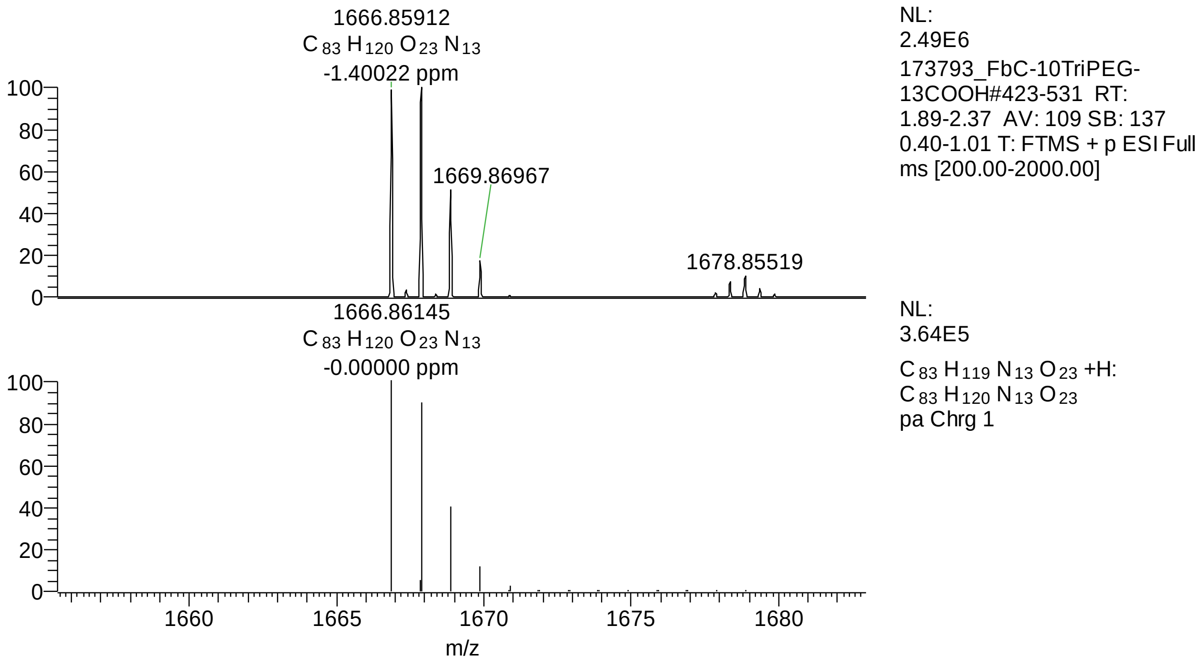
1666.85912
C 83 H 120 O 23 N 13
-1.40022 ppm
1669.86967
1678.85519
NL:
2.49E6
173793_FbC-10TriPEG-13COOH#423-531 RT: 1.89-2.37 AV: 109 SB: 137 0.40-1.01 T: FTMS + p ESI Full ms [200.00-2000.00]
1666.86145
C 83 H 120 O 23 N 13
-0.00000 ppm
NL:
3.64E5
C 83 H 119 N 13 O 23 +H:
C 83 H 120 N 13 O 23
pa Chrg 1
100
80
60
40
20
0
1660
1665
1670
1675
1680
m/z

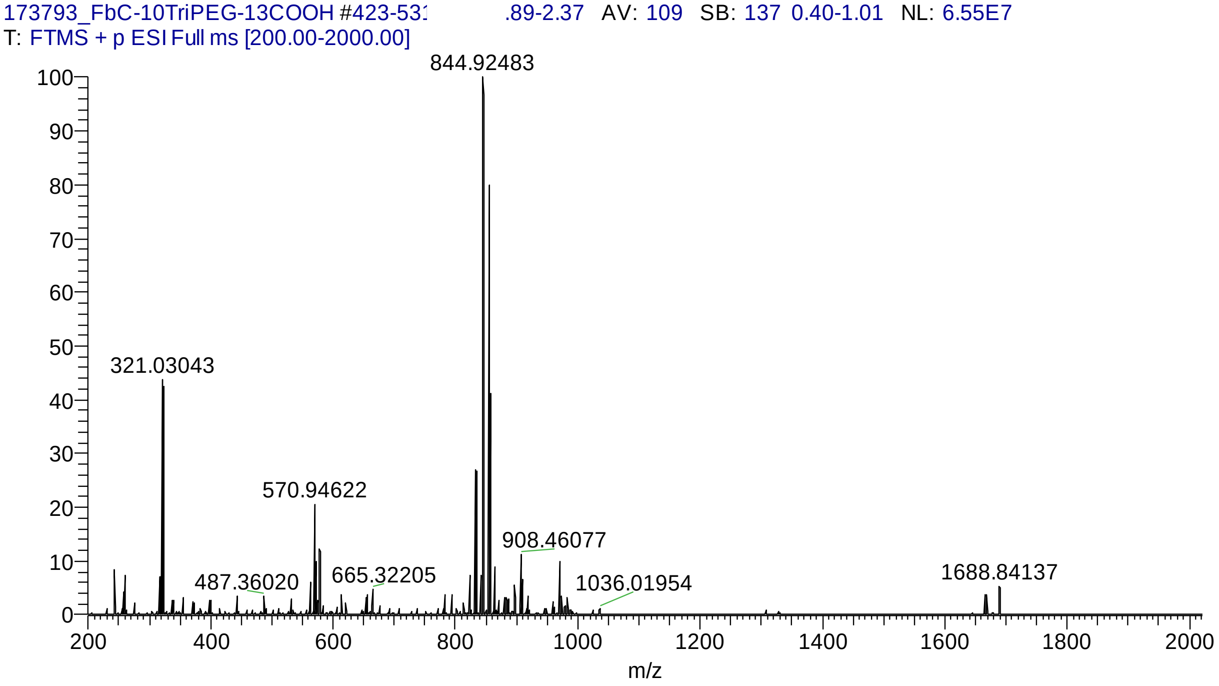
173793_FbC-10TriPEG-13COOH #423-53 .89-2.37 AV: 109 SB: 137 0.40-1.01 NL: 6.55E7
T: FTMS + p ESI Full ms [200.00-2000.00]
844.92483
321.03043
570.94622
487.36020
665.32205
908.46077
1036.01954
1688.84137
100
90
80
70
60
50
40
30
20
10
0
200
400
600
800
1000
1200
1400
1600
1800
2000
m/z

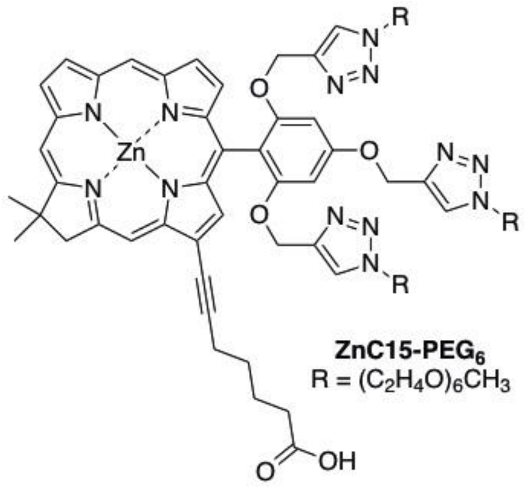
N
Zn
O
R
ZnC15-PEG$_6$
R = $(C_2H_4O)_6CH_3$
OH

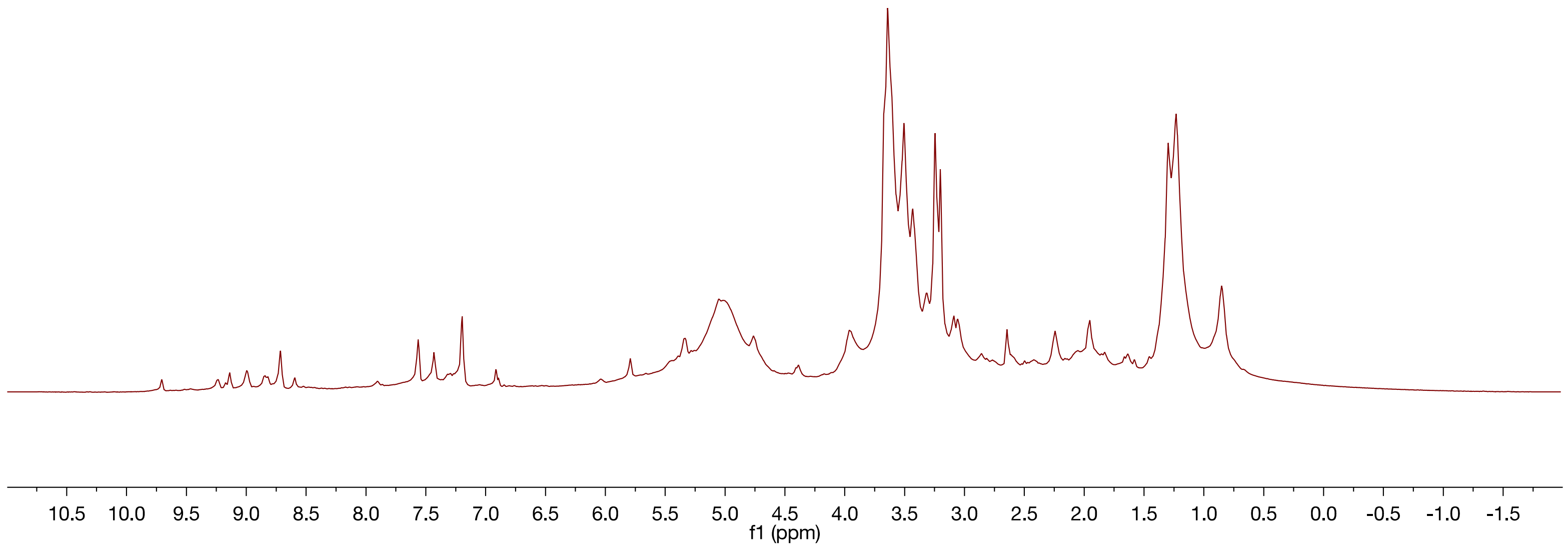
10.5 10.0 9.5 9.0 8.5 8.0 7.5 7.0 6.5 6.0 5.5 5.0 4.5 4.0 3.5 3.0 2.5 2.0 1.5 1.0 0.5 0.0 -0.5 -1.0 -1.5
f1 (ppm)

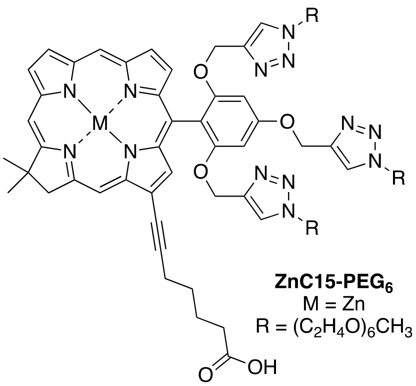

ZnC15-PEG$_6$
M = Zn
R = $(C_2H_4O)_6CH_3$

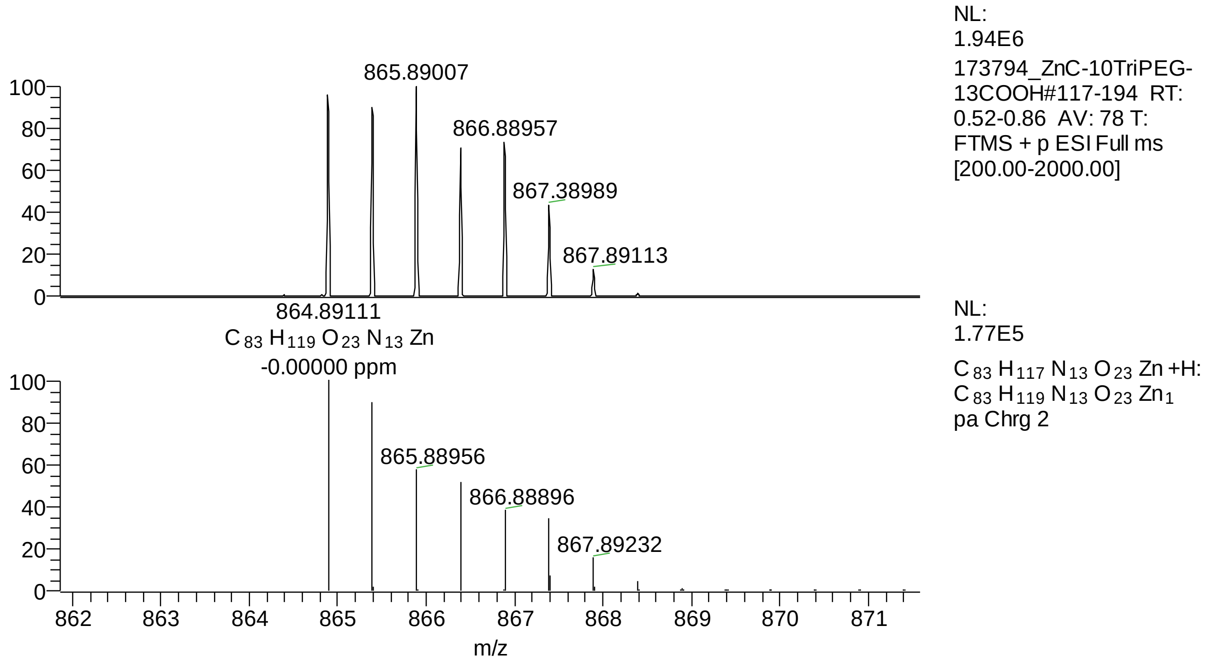

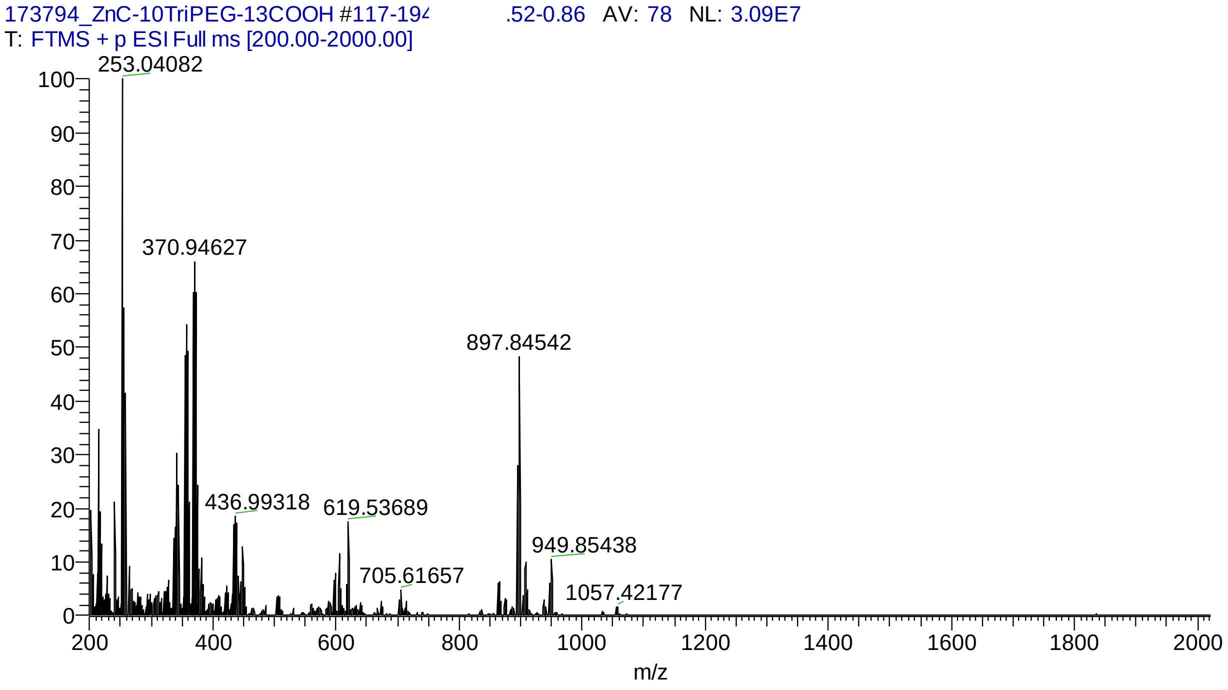

Supplement: Supplementary file 1 [file molecules-23-00130-s001.pdf]
